# RNAVirHost: a machine learning–based method for predicting hosts of RNA viruses through viral genomes

**DOI:** 10.1093/gigascience/giae059

**Published:** 2024-08-22

**Authors:** Guowei Chen, Jingzhe Jiang, Yanni Sun

**Affiliations:** Department of Electrical Engineering, City University of Hong Kong, Kowloon, Hong Kong (SAR), China; Key Laboratory of South China Sea Fishery Resources Exploitation & Utilization, Ministry of Agriculture and Rural Affairs, South China Sea Fisheries Research Institute, Chinese Academy of Fishery Sciences, Guangzhou 510300, China; Department of Electrical Engineering, City University of Hong Kong, Kowloon, Hong Kong (SAR), China

**Keywords:** RNA virus, host prediction, machine learning, metagenomics

## Abstract

**Background:**

The high-throughput sequencing technologies have revolutionized the identification of novel RNA viruses. Given that viruses are infectious agents, identifying hosts of these new viruses carries significant implications for public health and provides valuable insights into the dynamics of the microbiome. However, determining the hosts of these newly discovered viruses is not always straightforward, especially in the case of viruses detected in environmental samples. Even for host-associated samples, it is not always correct to assign the sample origin as the host of the identified viruses. The process of assigning hosts to RNA viruses remains challenging due to their high mutation rates and vast diversity.

**Results:**

In this study, we introduce RNAVirHost, a machine learning–based tool that predicts the hosts of RNA viruses solely based on viral genomes. RNAVirHost is a hierarchical classification framework that predicts hosts at different taxonomic levels. We demonstrate the superior accuracy of RNAVirHost in predicting hosts of RNA viruses through comprehensive comparisons with various state-of-the-art techniques. When applying to viruses from novel genera, RNAVirHost achieved the highest accuracy of 84.3%, outperforming the alignment-based strategy by 12.1%.

**Conclusions:**

The application of machine learning models has proven beneficial in predicting hosts of RNA viruses. By integrating genomic traits and sequence homologies, RNAVirHost provides a cost-effective and efficient strategy for host prediction. We believe that RNAVirHost can greatly assist in RNA virus analyses and contribute to pandemic surveillance.

## Introduction

Viruses are obligate intracellular parasites that depend on living organisms for their replication and survival. RNA viruses, possessing RNA as their genetic material, have the capability to infect a diverse array of organisms. For example, several types of RNA viruses are causal agents of the most disastrous pandemics in human history, including COVID-19, severe acute respiratory syndrome, the annual influenza, and so on. Furthermore, certain plant and animal RNA viruses pose a threat to agricultural and animal sectors, jeopardizing crop growth and livestock health and subsequently leading to substantial economic losses in agriculture and animal husbandry. Besides eukaryotic hosts, some RNA viruses can also infect bacteria and thus directly affect the dynamics of microbiome [1].

Besides its profound influence, viruses are believed to be the most diverse and abundant biological entities in the world [2]. Currently, metagenomic and metatranscriptomic sequencing has emerged as the primary approach for the discovery of novel viruses, as it eliminates the need for virus isolation and cultivation in laboratory settings. This method involves directly sequencing genetic material from host-associated or environmental samples, allowing for the identification of viruses present within these complex ecosystems. While the application of metagenomic/metatranscriptomic sequencing technologies has facilitated the discovery of the viral dark matter [3–6], how to determine the hosts of the newly identified viruses remains challenging owing to the complex composition of the metagenomic sequencing samples.

Understanding the interaction between viruses and their hosts is a fundamental step in characterizing the role of viruses in public health, animal husbandry, agriculture, and so on. While the concept of a virus’s host has multiple subconcepts related to the differences in replication, transmission, and pathogenicity [7–9], we follow the definition of host in the commonly used database, Virus-Host Database [10], which defines hosts as cellular organisms that viruses can infect and replicate within their cells. Besides their hosts, viruses may be detected in nonhost organisms due to the symbiotic relationship, the dietary interaction, or the physical contact, like the bacteria-infecting viruses and the plant-associated viruses found in birds’ digestive tract [11] and the plant-infecting viruses found in insect vectors [12]. These carriers are not the primary focus of our study.

### Related work

Traditionally, host verification requires stringent experimental contribution, including isolating viral particles from hosts of interest, serological tests, epidemiological investigation, and virus phylogenetic analyses. These processes are time-consuming and labor-intensive, and they often require specialized equipment and expertise. While metagenomic sequencing is becoming the main source of novel viruses, the heterogeneous composition made it harder to determine the target hosts. Therefore, when novel viruses rapidly emerge, predicting the hosts from the virus genome sequences, avoiding the tedious laboratory steps, shows its attractive advantage in terms of economy and efficiency.

By far, a number of computational works have been conducted to explore the association between viruses and the potential hosts. Many of these tools focus on prokaryotic viruses. For example, to predict the hosts of phages (viruses infecting prokaryotes), VPF-Class classified a set of viral protein families and aligned the query virus to the categorized references [13]. RaFAH generated protein clusters, constructed profile hidden Markov models (pHMMs), and trained a random forest classification model using the pHMM alignment score [14]. DeepHost encoded the spaced *k*-mer feature by a 3-dimensional matrix and trained a convolutional neural network to predict the hosts [15]. CHERRY integrated various signals, including gene organization, CRISPR, sequence similarity, and *k*-mer usage, and predicted the virus–host association by a graph convolutional encoder and decoder [16]. Currently, these tools allow host prediction at different ranks, and the accuracy decreases more with a refined host range (e.g., from class to species). Nevertheless, these tools are limited to host prediction for prokaryotic viruses. Neither their tools nor the methodology can be applied to eukaryotic viruses.

Compared to the extensive studies on phages, the host prediction of RNA viruses remains challenging and arduous. The typical genomes of RNA viruses, ranging from 3 to 41 kbp [17], are smaller than that of DNA viruses (5–600 kbp) and have limited capacity to carry host tropism signals [18]. While the sequence matches between phages and the prokaryotic genomes facilitate the host prediction of phages, they are less common in RNA viruses [1, 19]. Furthermore, while many bacterial genomes have been sequenced with metagenomic sequencing, the extensive host range of RNA viruses, limited availability of the potential hosts, and the large sizes of the potential host genomes make adding host genome features very difficult. Finally, the high mutation rate of RNA viruses makes the genomes less conserved, so the existing achievement cannot be extended to the novel viruses.

With these challenges, the computational frameworks still show outstanding performance in 2 host prediction scenarios. The first is to predict the host for a specific group of RNA viruses. Raj et al. [20] counted the spaced amino acid *k*-mer frequency and trained an Alternating Decision Tree classifier for 2 families, Picornaviridae and Rhabdoviridae. Eng et al. [21] encoded the protein sequences by the physical and chemical properties of the amino acid and trained a random forest for the influenza A virus. Mock et al. [22] developed 2 deep neural network models for the host classification of 3 viruses, respectively (influenza A virus, rabies lyssavirus, and rotavirus A). These viruses are associated closely with human activity and thus attract attention.

In another scenario, the researchers discuss whether the query viruses will infect the targeted host group, particularly humans and mammals. Zhang et al. [23] leveraged the *k*-mer frequency and designed a k-nearest neighbor model to discriminate the human-infecting viruses from other viruses. Bartoszewicz et al. [24] applied the reverse-complement neural networks to do read-based prediction of the viral host (human or nonhuman). Pandit et al. [25] investigated the host-sharing network of mammalian viruses and trained gradient boosting models to predict the host-sharing situation of 2 viruses. Zhang et al. [26] generated a set of protein families that are commonly shared by mammals, studied the correlation between these proteins and cross-species transmission, and trained a random forest model to predict the transmission of viruses.

However, these 2 categories of works are hard to extend to the host prediction of metagenome-assembled RNA viruses. They overlooked the broader host range of RNA viruses, including additional host candidates, like plants, invertebrates, and fungi. To predict hosts of the increasing novel RNA viruses, some primary explorations have been made. Babayan et al. [27] investigated the genomic traits and the sequences homologs of viruses and developed a classification model considering viruses from 12 taxonomic groups and 11 host groups. Building upon the study by Babayan et al., Lee et al. [28] further evaluated the application of machine learning with digital signal processing-based structural patterns (M-SP) of viruses in host prediction. Young et al. [29] assessed the gene content and the frequency of short sequences and developed a hierarchical host classification framework based on a support vector machine. Guo et al. [30] trained a 2-branch convolutional neural network to capture the informative motifs and classified the viruses into 5 host groups. Despite the promising results obtained from these validations, these studies still face the challenge of limited viruses and host ranges.

In this work, we concentrate on predicting hosts of emergent novel viruses and thus developed a hierarchical host classification framework, RNAVirHost. Combining virus taxonomy, genomic traits, and sequence homologies, RNAVirHost allows predicting the hosts using only viral genomes. To cover as many viruses and hosts as we can, RNAVirHost accepts queries from over 30 virus orders and includes 5 host types in its first layer, including Chordata (Vertebrate), Invertebrate, Plant, Fungi, and Bacteria. After obtaining the prediction results in the first layer, RNAVirHost will perform additional predictions in the second layer to obtain more precise host classification information. By evaluating various features and learning architectures in a more comprehensive database, we demonstrated the outstanding performance of RNAVirHost in host prediction of RNA viruses. We also evaluated RNAVirHost’s performance on novel viruses by conducting leave-one-taxon-out experiments. The results of these experiments demonstrated that RNAVirHost can be effectively applied across the diverse landscape of viruses without being limited to specific viral types.

## Method

### Overview of the method

**Figure 1:**
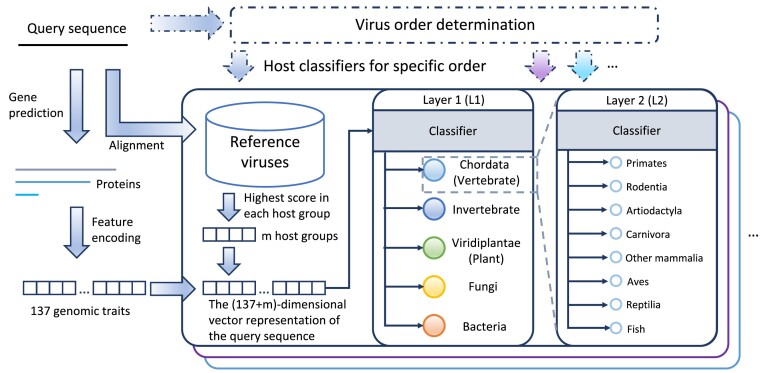
The framework of RNAVirHost. The hierarchical host prediction consists of 2 classification layers, layer 1 (L1) and layer 2 (L2). In layer 1, we predict hosts at kingdom and phylum level, including Chordata, Invertebrate, Viridiplantae, Fungi, and Bacteria. In layer 2, we further predict the specific host groups under Chordata at class and order level. The genomic traits consist of the usage preference of nucleotide, dinucleotide, codon, and amino acid. We categorized the reference viruses based on their hosts, and m denotes the number of host groups (output labels) in the corresponding classifiers.

The framework of RNAVirHost is depicted in Fig. 1. Initially, we categorize the queries based on their taxonomic information to narrow down the potential host range. Then, we extract 2 types of features, genomic traits and sequence homology, for host prediction using a learning-based model. This 2-step approach incorporates both the taxonomic information and the potential host signal in the sequences and is anticipated to enhance the accuracy of our predictions with greater confidence.

The origin and evolution of RNA viruses remain complicated and puzzling [5]. Different groups of viruses have distinct infection mechanisms and divergent host ranges. To benefit the host prediction, we incorporated prior virus taxonomic knowledge in RNAVirHost to mitigate potential interference among viruses originating from different sources. While the lower taxonomic rank may provide a precise host range, the emergent novel sequences may not fit neatly into the existing taxonomic label. Given the trade-off between accommodating more candidates and requiring informative taxonomic knowledge, we categorized viruses into 30 orders following ViralZone [31]. The measure is also expected to improve the learning performance by avoiding imbalance sizes of different virus orders within the same host groups. Then, we built independent classifiers for every order by leveraging the virus members’ genomic traits and sequence homologies.

RNAVirHost predicts the most likely hosts of queries hierarchically. The models are of 2-layer tree structures, with the first layer corresponding to the host’s kingdom and phylum level and the second layer to the host’s class and order level (Section Labels screening). Once input to corresponding models, the query will be encoded as its genomic traits and sequence homologies (Section Feature Encoding). RNAVirHost will output the host labels along the tree. Based on comprehensive benchmarks, eXtreme gradient boosting (XGBoost), a scalable machine learning technique, delivered the top performance, making it the preferred choice as the default architecture for RNAVirHost. When more data are available, RNAVirHost can be easily scaled to include the new members.

### Data preprocessing

#### Data collection

We curated a comprehensive dataset of RNA viruses for our analysis. First, we collected 6,735 viruses from the Virus–Host Database [10], a popular virus–host association database covering complete viral genomes from NCBI RefSeq and other reliable sources. Given the high diversity of RNA viruses, we further supplemented the dataset with 126,417 complete viral sequences with host annotations from NCBI GenBank. To ensure data quality, we filtered GenBank sequences for “complete genome” or “complete cds” with lengths between 3 and 50 kbp, representing the typical genome size range for RNA viruses. To remove redundancy, we combined these 2 datasets and used CD-HIT [32] to de-replicate the reference sequences at 90% average nucleotide identity and 80% coverage.

Although the processes above promised the quality of the sequences, we need to further check the reliability of the host annotations from GenBank. We cross-referenced them with the NCBI Taxonomy database [33] and manual validation. Host tags were standardized to the corresponding scientific names, and the ambiguous annotations were removed. Then, the dataset was used as a high-quality reference dataset as input to the label screening step. For detailed information on data collection and preprocessing, please refer to the Supplementary Information (Section 1).

#### Label screening

Although numerous RNA viruses have been found, the current database is biased toward human- and mammalian-associated viruses. To better predict the host lineage, we carefully curated the host labels based on both the host phylogenetic tree and the data availability. For each virus order, we built a 2-layer host phylogenetic tree. Layer 1 contains 5 branches (Chordata, Invertebrate, Viridiplantae, Fungi, and Bacteria), which are categorized into kingdom and phylum level. Layer 2, designed for the Chordata subtree, has 10 leaves, which are at the class and order level. Hence, we hierarchically predict the host lineage along the tree.

This hierarchical partition acknowledges the practical consideration that host switch phenomena are more frequently observed at the hosts’ class and order level [34, 35], and it offers users the flexibility to use both layers or 1 layer of host prediction of RNAVirHost.

Some host labels have only a few recorded infecting viruses and thus are not ready for computational host prediction. For virus orders containing more than 30 viruses, we set the threshold as 10 and only kept host labels with at least 10 infecting viruses. Besides, as we are aiming at predicting the most likely host of the metagenome-assembled viruses, which are expected to be shaped by the selection forces during their long-term coevolution with hosts, the viral sequences with multiple labels provide redundant and potentially conflicting information and are not included in the assessment.

After careful label screening, the dataset contains 14,500 viruses and spans 30 virus orders. Their host distribution is shown in Table 1.

**Table 1:** The virus order and host distribution after the label screening

	Layer 1						Layer 2									
Order	Number	Chordata	Invertebrate	Viridiplantae	Fungi	Bacteria	Primates	Rodentia	Carnivora	Artiodactyla	Chiroptera	Other Mammalia	Aves	Reptilia	Amphibia	Fish
Ortervirales	2,764	92.0%	—	8.0%	—	—	85.9%	0.9%	0.9%	2.0%	—	0.7%	1.6%	—	—	—
Picornavirales	2,647	74.2%	12.2%	13.6%	—	—	40.2%	4.1%	4.2%	14.5%	2.6%	1.7%	3.9%	0.9%	—	2.0%
Bunyavirales	1,524	52.4%	31.6%	15.2%	0.9%	—	18.0%	17.6%	—	1.6%	1.4%	5.3%	1.7%	5.6%	—	1.2%
Tymovirales	1,042	—	2.6%	94.3%	3.1%	—	—	—	—	—	—	—	—	—	—	—
Reovirales	1,034	52.9%	33.8%	9.7%	3.6%	—	9.9%	1.3%	2.9%	9.0%	5.5%	3.6%	9.6%	1.3%	—	10.0%
Amarillovirales	817	85.2%	14.8%	—	—	—	54.0%	9.7%	—	10.0%	4.0%	4.2%	1.7%	—	—	1.6%
Mononegavirales	758	57.5%	27.8%	11.3%	3.3%	—	11.3%	6.5%	4.0%	5.4%	11.5%	2.4%	9.0%	1.6%	—	5.9%
Martellivirales	670	5.1%	6.3%	73.7%	14.9%	—				5.1%			—	—	—	—
Nidovirales	622	94.4%	5.6%	—	—	—	4.8%	5.3%	5.6%	36.3%	17.4%	5.6%	13.5%	5.8%	—	—
Patatavirales	558	—	—	100.0%	—	—	—	—	—	—	—	—	—	—	—	—
Ghabrivirales	393	—	14.0%	9.9%	76.1%	—	—	—	—	—	—	—	—	—	—	—
Durnavirales	340	5.0%	—	32.4%	62.6%	—				5.0%			—	—	—	—
Stellavirales	296	100.0%	—	—	—	—	10.1%	12.8%	8.4%	33.1%	5.1%	—	16.2%	—	4.4%	9.8%
Tolivirales	226	—	15.0%	73.9%	11.1%	—	—	—	—	—	—	—	—	—	—	—
Hepelivirales	181	80.1%	10.5%	9.4%	—	—	32.0%	16.6%	—	16.6%	—	8.8%	6.1%	—	—	—
Sobelivirales	120	—	12.5%	87.5%	—	—	—	—	—	—	—	—	—	—	—	—
Blubervirales	108	100.0%	—	—	—	—	75.9%	—	—	—	14.8%	—	9.3%	—	—	—
Cryppavirales	80	—	—	—	100.0%	—	—	—	—	—	—	—	—	—	—	—
Articulavirales	77	100.0%	—	—	—	—				59.7%			16.9%	—	—	23.4%
Jingchuvirales	61	—	100.0%	—	—	—	—	—	—	—	—	—	—	—	—	—
Nodamuvirales	42	—	100.0%	—	—	—	—	—	—	—	—	—	—	—	—	—
Ourlivirales	38	—	—	26.3%	73.7%	—	—	—	—	—	—	—	—	—	—	—
Wolframvirales	23	—	—	—	100.0%	—	—	—	—	—	—	—	—	—	—	—
Mindivirales	22	—	—	—	—	100.0%	—	—	—	—	—	—	—	—	—	—
Norzivirales	21	—	—	—	—	100.0%	—	—	—	—	—	—	—	—	—	—
Serpentovirales	16	—	—	100.0%	—	—	—	—	—	—	—	—	—	—	—	—
Muvirales	9	—	100.0%	—	—	—	—	—	—	—	—	—	—	—	—	—
Yadokarivirales	7	—	—	—	100.0%	—	—	—	—	—	—	—	—	—	—	—
Goujianvirales	3	—	100.0%	—	—	—	—	—	—	—	—	—	—	—	—	—
Timlovirales	1	—	—	—	—	100.0%	—	—	—	—	—	—	—	—	—	—
Sum	14,500															

Each row represents the host distribution of a virus order. While the column “Num” shows the total number of viruses in the order, the following columns represent the percentage of viruses infecting the corresponding hosts. Layer 2 consists of the Chordata subgroups from layer 1. Therefore, the sum of values in the second layer equals the value of “Chordata.” In cases (Martellivirales, Durnavirales, Articulavirales) where mammalian viruses are fewer than 50, we merge mammalian members into a single node, Mammalia.

### Feature encoding

Previous studies have extensively explored various features of RNA viruses with different hosts. Both the genomic traits and viruses’ sequence homologies facilitated the host prediction of RNA viruses. RNAVirHost relies on a subset of the genomic traits and the sequence homology. A widely accepted hypothesis is that the biases in genomic composition, also named as genomic traits, may hint the natural selection pressure imposed by their hosts. To escape host immune responses and hijack the cellular machinery, viruses tend to mimic the genomic trait usage of their hosts [36, 37]. It is reported that Flaviviridae viruses associate with 2 host groups, vertebrate and invertebrate. The members infecting a single group have a similar dinucleotide and codon preference as their hosts do [38]. Besides, the changes of codon pair bias were proven to influence the viruses’ pathogenicity [39], showing that the host tropism potentially relates to the genomic traits. To represent the genomic features, we translate the query sequences to proteins using MetaProdigal [40] and generate a 137-dimensional vector $\mathbf {S} \in \mathbb {R}^{137}$, where $S_i$ quantifies the preference of 137 genomic traits, including the usage preference of nucleotide (Eq. 1), dinucleotide (Eq. 2), codon (Eq. 3), and amino acid (Eq. 4).

On the other hand, related viruses tend to infect hosts that share taxonomic associations or have overlapping activity patterns. Thus, the viruses’ sequence homology may indicate their host range. The sequence homology is introduced by conducting sequence alignment. The reference sequences are categorized into different groups by their hosts, and we used BLASTN to get the maximum alignment scores of the query against every virus group. The maximum alignment scores against all groups are converted into a m-dimensional vector, $\mathbf {H} \in \mathbb {R}^{m}$, where m is the number of virus groups (host labels) in the corresponding classifier. Finally, the 2 vectors, $\mathbf {S}$ and $\mathbf {H}$, are concatenated into a (137 + m)–dimensional vector, $\mathbf {X} \in \mathbb {R}^{137+m}$, and used as the representation of the query. The combination of genomic traits and viral sequence homology is expected to facilitate predicting the hosts. Here, we briefly describe the different features, and more details are depicted in the Supplementary Information (Section 2).

#### Features from sequence composition (genomic traits)

The sequence composition describes the relative abundance or occurrence of short strings of nucleotide or amino acid. By far, the *k*-mer frequency has been widely used in various tasks, like taxonomy classification [41], sequence annotation [42], and host prediction [15]. Based on the length, the *k*-mer frequency can be defined in different formats. Referring to the previous works on host determination [27, 38], we define the composition by the following equations.


(1) \begin{eqnarray*}
P_{x} = n_{x} / \sum _{x}{n_{x}}
\end{eqnarray*}

(2) \begin{eqnarray*}
P_{xy} = \frac{n_{xy} / \sum _{x, y}{n_{xy}}}{P_x * P_y}
\end{eqnarray*}

(3) \begin{eqnarray*}
P_{xyz} = \frac{n_{xyz}}{n_{A}}
\end{eqnarray*}

where *x, y*, and *z* are nucleotide and the codon $xyz$ encodes the amino acid *A*,


(4) \begin{eqnarray*}
P_{A} = \frac{n_{A}}{\sum {n_A}}
\end{eqnarray*}

(5) \begin{eqnarray*}
CPS_{x_1y_1z_1, x_2y_2z_2} = \frac{n_{x_1y_1z_1x_2y_2z_2}}{n_{AB}*P_{x_1y_1z_1}* P_{x_2y_2z_2}}
\end{eqnarray*}

where the codon $x_{1}y_{1}z_{1}$ encodes the amino acid *A* and the adjacent codon $x_{2}y_{2}z_{2}$ encodes *B*. The occurrence is denoted as *n*.

To implement normalization, any zero value or missing values are replaced with a small number (1e-4) as a default, and a log2 transformation is applied to all values. Generally, when the value is positive the corresponding feature is overrepresented in the genome; otherwise, the feature is deemed to be underrepresented.

#### Features from sequence alignment

We introduced sequence alignment score as a feature vector of the query. Specifically, the reference sequences are categorized into different groups by their hosts, and we aligned the query against the references. The highest alignment score of each group was kept as the potential association between the query and the corresponding host. Hence, the query will be represented as a vector of m*1, where m is the number of host labels. If no alignment is found, we set the association to be 1. A log10 transformation is applied to all scores. In this research, we used BLASTN as the default alignment tool. Instead of k-best matches, which tend to be affected by the data imbalance, we only consider the best alignment of each group.

### Performance evaluation metrics

The collected 14,500 virus records belong to 30 virus orders. As described in Section Label screening, for each virus order, we examine their host label distribution. We found that 12 virus orders exclusively infected a specific host group, and we directly assigned host labels to them. These virus orders include Patatavirales, Cryppavirales, Jingchuvirales, Nodamuvirales, Wolframvirales, Mindivirales, Norzivirales, Serpentovirales, Muvirales, Yadokarivirales, Goujianvirales, and Timlovirales. The remaining 18 virus orders involved infections across multiple host groups. Among these 18 orders, 13 received labels in the second layer of the host phylogenetic tree. Accordingly, we conducted host prediction and evaluation for the corresponding virus orders and host groups.

To evaluate the performance of RNAVirHost, we employed various metrics, including accuracy, precision, F1 score, and prediction rate. Accuracy serves as a fundamental metric, representing the proportion of correctly predicted queries out of the total number of queries. To provide a nuanced assessment of RNAVirHost’s performance across different taxonomic levels, we introduced rank-wise accuracy (order-wise, family-wise, and genus-wise), which is computed by averaging the accuracy of respective virus orders, families, and genera.

Additionally, prediction rate quantifies the ratio of output predictions to the total number of queries, while precision captures the ratio of correctly predicted queries to the total number of output predictions. Recognizing the potential bias in reference labels toward human and vertebrate hosts, we included the macro F1 score as an additional evaluation metric. The macro F1 score is calculated by averaging the F1 scores of each individual host label, where the F1 score for a specific label is the harmonic mean of its precision and recall. By employing these metrics, we aim to offer a comprehensive and granular evaluation of RNAVirHost’s performance, enabling a robust analysis of its predictive capabilities and strengths.

## Results

We conducted comprehensive benchmark experiments on different scenarios to evaluate the performance of RNAVirHost. First, we compared various feature sets and their combinations by 5-fold cross-validation. The comparison showed that the subset of genomic traits outperformed other features. We also assessed the features’ contribution by machine learning strategies and validated the choice of genomic traits. We then evaluated different learning architectures, among which XGBoost achieved the highest accuracy and thus was chosen as the default architecture. Second, following the cross-validation setting, we assessed the impact of sequence completeness on host prediction by generating viral sequences with various levels of completeness. Third, we focused on host prediction for novel RNA viruses. Specifically, we evaluated RNAVirHost’s performance on novel viruses by conducting leave-one-taxon-out experiments. The results of these experiments demonstrated that RNAVirHost can be effectively applied across the diverse landscape of viruses, without being limited to specific viral types. Finally, to show the accuracy and utility of RNAVirHost in real experiments, we collected some recently identified viruses by researches and tested RNAVirHost’s performance on different host groups.

### Assessment via cross-validation

In this experiment, we follow the standard evaluation strategy in machine learning to examine the performance of different types of features and learning models. There are many types of features that may help host prediction. We first evaluated the different feature sets and their combinations by stratified 5-fold cross-validation. For each virus order, we stratified the virus into nonoverlapping 5-fold by host labels, trained models using 4 out of 5 folds, and tested them in the remaining one. This assessment was performed on each fold, and we presented the overall performance on all 5 folds.

We tested 11 feature sets by ensemble learning, XGBoost. To begin with, we evaluated the classification validity of each feature set without combinations. Referring to Babayan et al. [27], we regarded nucleotide preference, dinucleotide preference, codon usage, codon pair bias, and amino acid usage as 1 feature set, named as “Bias” here. Considering that most RNA viruses are shorter than 50 kbp and there are 3,904 codon pairs (excluding pairs led by stop codons), which is a high-dimensional feature compared to the limited sequences records, we made a subset feature from Bias by excluding the codon pair bias, named as “sBias” (subset). Besides the bias-related features, we evaluated 9 more types of features, including BLASTN (best hit), digital signal processing-based structural patterns (M-SP), and the frequency of 6-mer, 7-mer, 8-mer, amino acid 3-mer (AA3), AA4, physiochemical 5-mer (PC5), and PC6 in [28, 29]. M-SP involves generating the Fourier transformation (FT) of the biological sequences, computing the correlation coefficients among the FT, and obtaining the distances matrix of sequences through the correlation coefficients [28]. A detailed description of the benchmarked feature can be found in the Supplementary Information (Section 2). Then, we assessed feature set combinations among Bias, BLASTN, and M-SP. DeepHoF [30], a host prediction tool based on the convolutional neural network, provided the classification of viruses associated with plants, germs, invertebrates, humans, and other vertebrates, where germs include bacteria and fungi. We included it in the benchmark assessment in layer 1 by merging “human” and “other vertebrate” to be Chordata.

#### The comparison among simple feature sets

**Figure 2:**
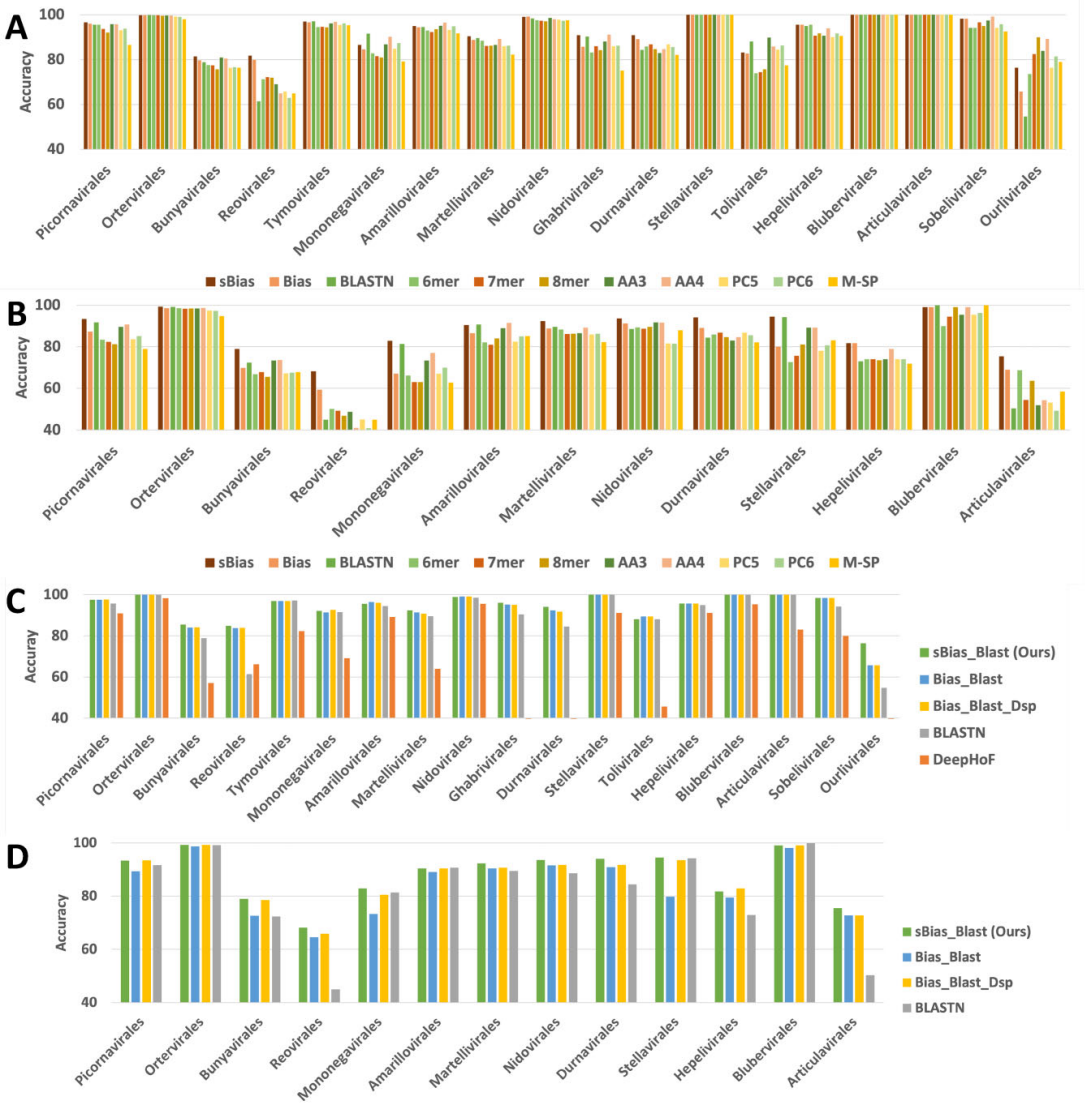
Host prediction performance (accuracy) of different feature sets in layer 1 (A) and layer 2 (B). Here, Bias denotes a set of genomic traits, including nucleotide preference, dinucleotide preference, codon usage, codon pair bias, and amino acid usage, while sBias denotes a subset that excludes the codon pair bias. Host prediction performance (accuracy) of different feature combinations in layer 1 (C) and layer 2 (D). Here, sBias_Blast is the combination of sBias and BLASTN. Bias_Blast is the combination of Bias and BLASTN. Bias_Blast_Dsp is the combination of Bias, BLASTN, and M-SP. DeepHoF is designed to predict hosts at the kingdom and phylum level (layer 1), and its output is transformed to corresponding labels (Germ score to be Fungi; Human score and Vertebrate score to be Chordata). X-axis: Virus orders sorted by size (from largest to smallest). The performance comparison in layer 2 considers the errors from both layer 1 and layer 2. Among the 18 orders, 13 can be further classified in layer 2. Therefore, we focused our performance evaluation solely on layer 2 within these 13 orders.

As shown in Fig. 2A, B), when using each of the 11 feature sets, the classifier with feature sBias achieved the highest accuracy of 92.38% and 83.92% for each virus order in L1 and L2, respectively. The models with feature Bias and BLASTN rank second and third in performance. The model with Bias got an accuracy of 90.89% and 82.14%, while using BLASTN got 89.64% and 81.57%. Besides, the M-SP traits, achieving an accuracy of 87.14% and 76.93%, did not provide accurate predictions when used solely. In terms of F1 score, as demonstrated in Supplementary Fig. S1A, B, the model with sBias ranked second by achieving F1 scores of 84.82% and 74.03%, outperformed by BLASTN, which got F1 scores of 86.40% and 78.22%. The decrease in F1 scores may result from the data imbalance, which has a more pronounced impact on the learning-based method.

The accuracy difference between sBias and Bias drew our attention, and we calculated the feature contribution to classification performance, as shown in the Supplementary Information (Section 3), Supplementary Table S1, and Supplementary Fig. S2. The result indicated that most codon pair scores did not contribute to the prediction, which is consistent with previous studies [27]. This can be explained by the sparsity of the codon pair in RNA viruses.

#### The comparison among feature combinations

Out of the 11 feature sets, Bias, sBias, and BLASTN scores show promising results in the cross-validation experiment. Hence, we further evaluated their combinations and reported the results in Fig. 2C, D and Supplementary Fig. S1C, D. When combined, the models with sBias_Blast achieved the best order-wise accuracy of 93.99% and 88.01%. While Bias_Blast and Bias_Blast_Dsp (the combination of Bias, BLASTN, and M-SP as [28]) are the second and third best groups, they had little difference in accuracy (93.14% and 86.98% for Bias_Blast, 93.14% and 86.96% for Bias_Blast_Dsp). With regards to F1 scores, the models with sBias_Blast also got the highest F1 scores of 88.41% and 80.59%. The combination significantly improved the prediction performance. The drops in accuracy and F1 scores from sBias to Bias and that from sBias_Blast to Bias_Blast validated the feature reduction that excludes the codon pair features. On the other hand, the result implied that the M-SP traits did not significantly benefit the prediction of RNA viruses’ hosts.

An important observation is that using BLASTN achieves quite comparable host prediction results as our learning-based methods. We thus further analyzed this. First, to ensure that the improvements observed in the host prediction models are not due to chance events, we conducted a further comparison among models based on different feature sets. This comparison involved examining the accuracy distribution across virus orders, as detailed in Supplementary Fig. S3. Through the 1-sided Wilcoxon test, we demonstrated that the observed improvements in host prediction accuracy are statistically significant and not merely a result of random chance.

Second, it is worth mentioning that when employing a random data partitioning strategy (5-fold cross-validation), it is not unexpected to observe that the predictive performance of BLAST yields similar results as learning-based methods. One main reason for this is that the test viruses, when randomly partitioned, tend to exhibit high similarity to certain viruses in the training data, resulting in a less challenging test scenario. Our next set of experiments will explore more realistic usage scenarios.

#### The comparison among machine learning architectures

Finally, we conducted an evaluation of several learning architectures, encompassing XGBoost, gradient boosting decision tree (GBDT), random forest (RF), support vector machine with RBF kernel, logistic regression, k-nearest neighbors, and Gaussian naive Bayes. Using the scikit-learn package [43], default parameters were employed to train these models, and their performance was assessed using accuracy as the evaluation metric. As demonstrated in Supplementary Fig. S4, XGBoost exhibited the highest accuracy of 94.0% (L1) and 88.0% (L2), while the second-best architecture (RF) achieved those of 93.3% and 87.5%, and the third best one (GBDT) got 92.7% and 86.2%.

Collectively, the model, trained with XGBoost and the combination of sBias and BLASTN, achieved the outperforming accuracy among various learning architectures, feature sets, virus groups, and host ranks.

#### Handling out-of-distribution host labels

As our primary objective is to predict the hosts of metagenome-assembled RNA viruses, we have designed a comprehensive label list that includes 4 eukaryotic kingdoms and 1 prokaryotic domain. However, considering the continuous emergence of novel RNA viruses, there are instances when the queried viruses may infect hosts outside the label list. To prioritize precision, we opt to reject virus queries that may not infect the target hosts, even if it leads to a lower prediction rate. To determine the trade-off between prediction rate and precision for both our tool and BLASTN, we analyzed the distribution of prediction scores, which is shown in Supplementary Information (Section 4) and Supplementary Fig. S5. Our analysis revealed that, while predicting hosts for the same number of queries, RNAVirHost achieved higher precision compared to BLASTN. Consequently, we provide users with an empirical prediction score cutoff for each virus order, allowing them to choose predictions with greater confidence. This empirical approach enables users to obtain more reliable predictions.

### Family-wise analysis reveals the potential host switch

**Figure 3:**
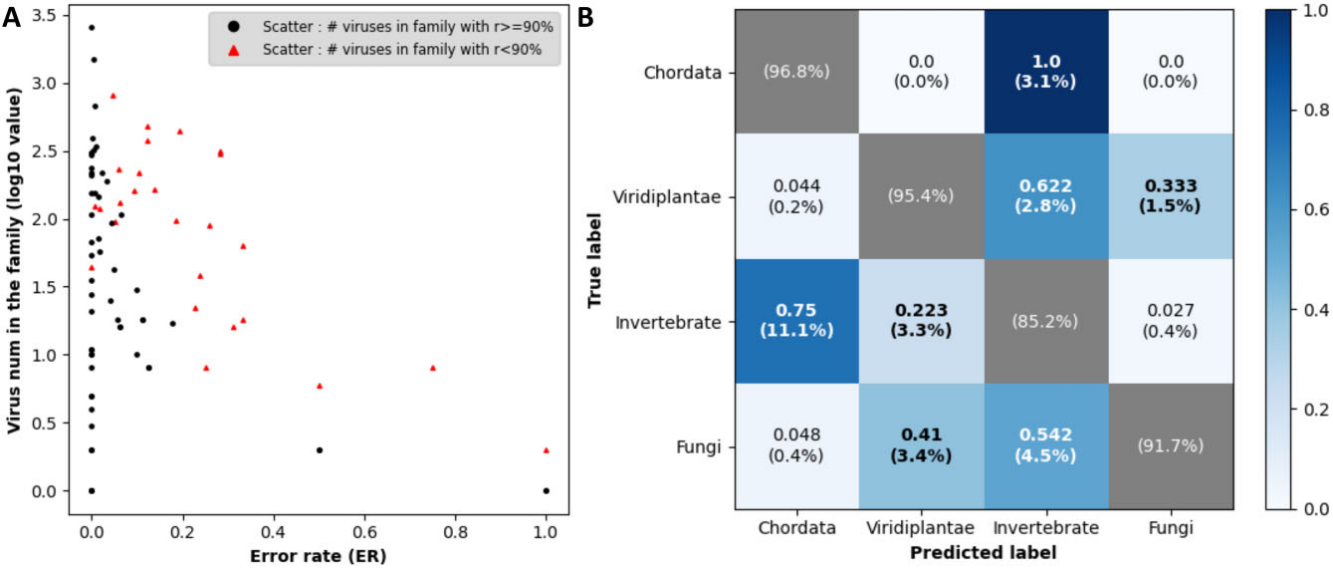
(A) The error rate distribution of RNAVirHost across different virus families in layer 1. The dots denote the number of virus members in each family (y-axis) and its corresponding error rate (x-axis). (B) The confusion matrix of RNAVirHost’s prediction in layer 1. The values outside the brackets denote the percentage of the misclassified members, which were normalized to the sum of misclassified viruses of the corresponding groups (by row). The values in the brackets denote the percentage of the prediction labels, normalized to the corresponding groups’ total number (by row). The viruses infecting bacteria are limited to specific virus orders, which exclusively infect bacteria. Hence, we do not visualize bacteria here.

In comparison to viruses within the same order, viruses in the same family generally have more consistent host ranges. We investigated the host distribution of each virus family and visualized the error rates of virus families in the first layer in Fig. 3A. In the 18 evaluated virus orders, there are 83 families and 13,238 evaluated viruses. In the first layer, the family-wise average accuracy is 87.55%, meaning that 658 out of the evaluated 13,238 viruses got wrong assignments of hosts. It is observed that the misclassification of RNAVirHost mainly distributes to those virus families with mixed host groups. To better visualize the trend, we counted the dominant host labels in every family and the ratio of members infecting the dominant hosts, denoted as $\mathbb {r}$. As a measure of homogeneity, $\mathbb {r}$ will have a small value if a family mainly contains viruses infecting different host groups. Otherwise, a virus family infecting only 1 dominant host group will get a $\mathbb {r}$ value close to 1. To examine the homogeneity values of various families, we set $\mathbb {r}$ as 90% as the threshold for high and low host homogeneity. Out of the 83 RNA virus families, there are 57 families with over 90% of their members infecting a unique host group ($\mathbb {r}$  $\ge$ 90%) and 26 families infecting multiple host groups ($\mathbb {r}$  $< $ 90%). In 658 error cases, only 57 (8.66%) came from the former ($\mathbb {r}$  $\ge$ 90%), while 593 (90.12%) belonged to the latter case ($\mathbb {r}$  $< $ 90%), implying that these wrong predictions are mainly from viruses with multiple hosts across phylum. For instance, Peribunyaviridae ($\mathbb {r} =$ 56.7%), Phenuiviridae ($\mathbb {r} =$ 53%), and Nairoviridae $\mathbb {r} =$ 70.8%) are virus families in Bunyavirales. They contain members that are well known for causing vector-boned diseases [44–46]. RNAVirHost achieved an accuracy of 71.34%, 70.67%, and 74.16% for the 3 families, which is lower than the family-wise average accuracy.

Additionally, we generated the confusion matrix to evaluate the predictions made by RNAVirHost, as shown in Fig. 3B. Analysis of the matrix revealed that viruses infecting chordates and Viridiplantae (plants) could be accurately classified. Only 0.2% of viruses infecting Viridiplantae were misclassified as chordate-associated, and no viruses infecting chordates were predicted to infect Viridiplantae. Similarly, viruses infecting chordates and fungi could be clearly distinguished. However, misclassification predominantly occurred among invertebrate-associated queries, with 11.1% of invertebrate-infecting viruses predicted to infect chordates and 3.1% of chordate-infecting viruses predicted to infect invertebrates. These predictions strongly suggest the presence of potential vector-borne viruses within the dataset. Additionally, errors occurred when distinguishing between viruses that infect invertebrates and those that infect plants and fungi. This confusion may arise due to the contact and dietary interactions between invertebrates and plants or fungi [47, 48]. Furthermore, there is some overlap between the viruses infecting fungi and those infecting plants, which could result from the symbiotic relationships between certain plants and fungi [49]. Although the focus of this work is placed on predicting the hosts, vector-borne viruses still have a great influence on the performance of host prediction.

### Performance on fragmented viral sequences

Given that the metagenome-assembled viruses are not always complete, we assessed the impact of sequence completeness on the host prediction. Following the setting of cross-validation, we partitioned the reference data into training and test sets. Then, we evaluated the robustness of RNAVirHost on the fragmented viral sequences with different length ratios generated from the test set.

**Figure 4:**
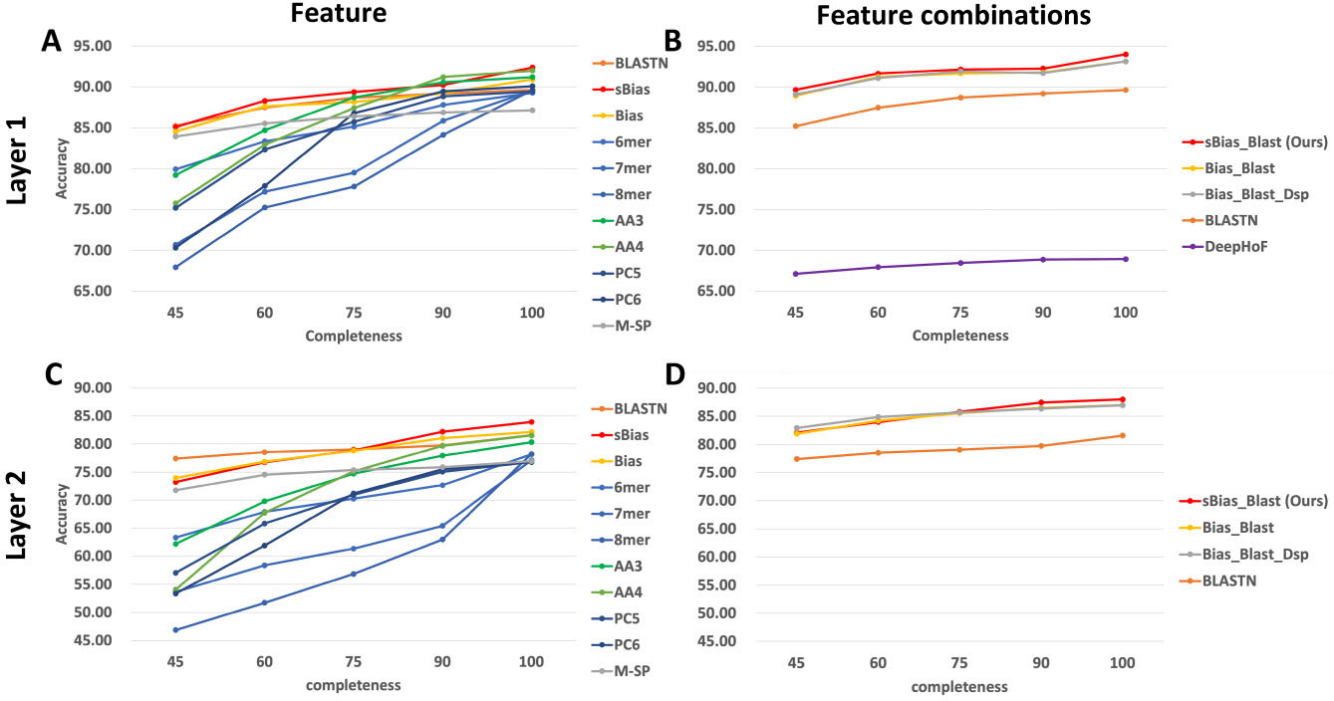
Host prediction performance (order-wise accuracy) of different feature sets in layer 1 (A, B) and layer 2 (C, D). X-axis: the completeness of viral sequences. Y-axis: The order-wise accuracy of the corresponding feature set.

First, we generated contigs by cutting the test sequences with 4 length ratios of the reference sequences: 90%, 75%, 60%, and 45%. Given that the collected reference sequences are of high quality and complete, it is reasonable to regard the length ratio as “completeness.” To avoid the potential bias, we generated 1 contig per reference sequence per group. For every completeness group, there are 13,737 contigs in the 18 targeted virus orders. We used these contigs to evaluate all strategies and reported the order-wise performance in Fig. 4 and Supplementary Fig. S6.

As the queries’ completeness decreased, the performance of all strategies deteriorated. Among the simple feature sets, sbias, being the most promising feature, outperformed other feature sets in every group except BLASTN in the 45% group and AA3 and AA4 in the 90% group in layer 1, and their performance differences are less than 1%. In layer 2, sBias, Bias, and BLASTN achieved the highest accuracy in all groups, while they showed less accuracy fluctuation with the decrease in completeness. These indicate the robustness of sBias and Bias against the changes of queries’ completeness.

Then, we compared the feature combinations. In layer 1, RNAVirHost achieved higher accuracy than other strategies. In layer 2, RNAVirHost outperformed others in the 75% and 90% groups. In the 45% and 60% groups, RNAVirHost got an accuracy of 82.09% and 83.98%, comparable to those of Bias_Blast_Dsp, 82.92% and 84.87%. Considering that maintaining a small feature number benefits the generalization of machine learning, we believe that sBias_Blast is more suitable in host prediction.

To sum up, sBias, Bias, and BLASTN showed their strong potential to avoid the effect of incompleteness. RNAVirHost, combining sBias and BLASTN, achieved the most advantageous performance in multiple completeness levels.

### Performance on novel RNA viruses

**Figure 5:**
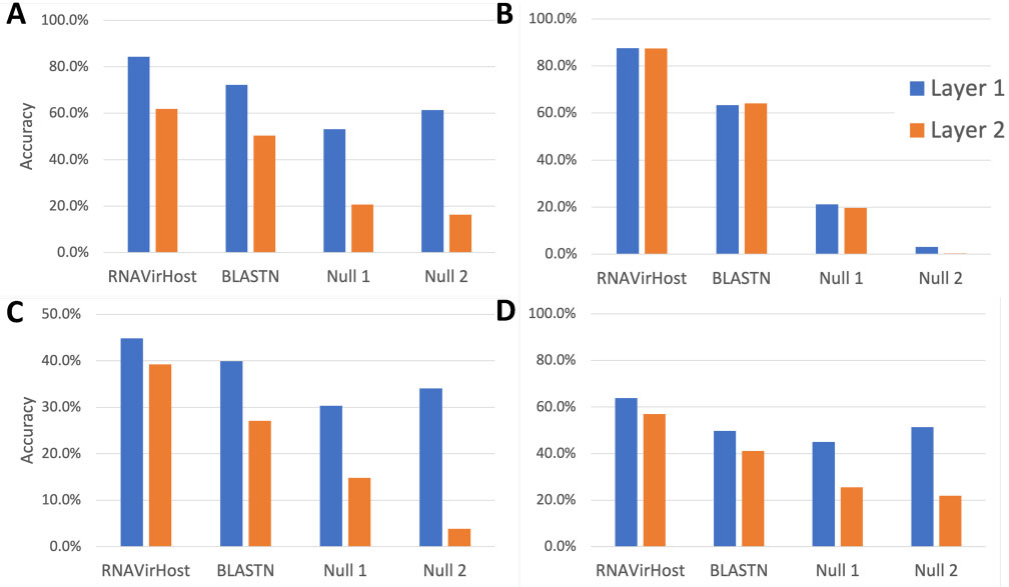
The performance comparison of different strategies in the leave-one-genus-out experiment. The figures demonstrate the average accuracy of (A) all 448 genera; (B) 138 genera in case 1, whose dominant host label is different from their order’s; (C) 50 genera in case 2, whose dominant host label is different from its family’s; and (D) 56 genera in case 3, with members infecting hosts of different kingdoms or phyla. Null 1 randomly assigns the host labels following the distribution of references. Null 2 determines the query’s host by the dominant host label in references.

With fast accumulated RNA viruses from the environmental sequencing samples, determining hosts of novel viruses is becoming more important. In the second part, we evaluated the capability of RNAVirHost to identify hosts of novel viruses using the leave-one-genus-out strategy. Specifically, we train the model without including specific genera and then assess its performance on those genera to mimic the situation where a novel query, particularly with an unknown genus label, is used as input. In this benchmark, we compared RNAVirHost against the alignment-based method, BLASTN, and 2 null models (null 1 and null 2). Null 1 randomly assigns the host labels following the host label distribution of reference viruses in the training data, while null 2 determines the query’s host using the dominant host label in the training data. The result is shown in Fig. 5. As DeepHoF does not allow us to retrain their model, we cannot include DeepHoF in this experiment.

In our dataset, there are 448 virus genera across 18 orders. We repeated the evaluation for each genus and assessed the genus-wise accuracy of the tools. Besides, some genera might have different host tropisms with their closely related viruses under the same order or family, which poses a great challenge in predicting their hosts. Below, we provide a comprehensive discussion of these challenging cases. We counted the dominant host labels in layer 1 for each virus order, family, and genus. According to the difference between the host tropisms of the genus and its corresponding order or family, we consider 3 challenging cases. **Case 1:** The genus’s dominant host label is different from its order’s. A typical case is *Nepovirus* in Picornavirales. While *Nepovirus* is a plant-infecting virus genus, 74% of members of Picornavirales infect chordates. **Case 2:** The genus’s dominant host label is different from its family’s, such as *Seadornavirus* in Sedoreoviridae. While mosquitoes are the natural host of *Seadornavirus*, members of Spinareoviridae mainly infect chordates. Some genera in case 2 also belong to case 1, indicating that the host tropism of these genera is divergent from their homologous viruses within the same family and order. **Case 3:** The members of the genus infect hosts from divergent kingdoms or phyla. By discussing the challenging cases, we expect to exhibit the performance of RNAVirHost in different situations more comprehensively.

In all 448 virus genera across 18 orders, RNAVirHost achieved the highest genus-wise average accuracy of 84.3% and 61.9% for L1 and L2, respectively, outperforming BLASTN by 12.1% and 11.5%. There are 138 genera in case 1, whose dominant host labels varied from their order’s; 50 genera in case 2, whose dominant host labels differ from their family’s; and 56 genera infecting hosts from multiple phyla. RNAVirHost achieved the best result in various cases. Specifically, in 138 genera in case 1, RNAVirHost got an accuracy of 87.5% (L1) and 87.4% (L2), outperforming BLASTN by 24.1% and 23.4%. In 50 genera in case 2, RNAVirHost got an accuracy of 44.8% (L1) and 39.2% (L2), surpassing BLASTN by 4.9% and 12.1%. In 56 genera with divergent host groups, RNAVirHost achieved an accuracy of 63.8% (L1) and 57.0% (L2), exceeding BLASTN by 14.1% and 15.9%. The improvement of RNAVirHost likely results from the utilization of genomic traits and the superiority of the machine learning method.

### Performance on recently identified viruses

Finally, we retrained RNAVirHost on all the reference viruses and assessed its accuracy on identifying hosts for recently identified viruses, whose hosts are derived based on experimental evidence. This process was designed to replicate scenarios that potential users of RNAVirHost might typically encounter.

To cover different host types, we collected 20 works that focused on identifying novel viruses on specific hosts and confirmed the viral infection by laboratory measures. While 15 were published after 2023, we expanded the dataset by including 5 more works published before 2023 to cover different hosts as comprehensively as possible. We manually curated the dataset to ensure that the reference dataset did not include any new viral sequences presented in the 20 works. To better demonstrate the accuracy of RNAVirHost on different hosts, we partitioned the sequences into 4 datasets, which associate with plants, invertebrates, fungi, and fishes (chordates), respectively, corresponding to the first layer’s host labels in our tool. Each dataset comprises viral sequences originating from diverse hosts, including those from economically important species like shrimp and salmon, as well as those from less-studied species like seahorses, *Stellaria aquatica* and *Cnidium officinale*. The statistics are listed in Table 2.

**Table 2:** The statistics regarding taxonomic groups and host labels of the newly sequenced datasets

	# Viruses	# Virus	Host group	# Host	# Host
		orders		classes	orders
Dataset 1	21	5	Plant	1	6
Dataset 2	15	3	Invertebrate	2	4
Dataset 3	69	9	Fungi	4	6
Dataset 4	21	6	Chordata—Fish	1	2

**Figure 6:**
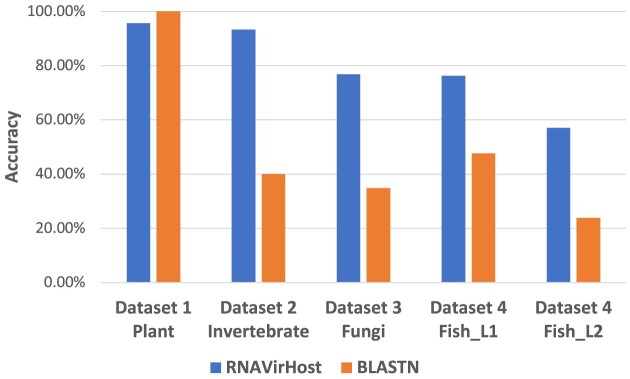
The host prediction accuracy of RNAVirHost and BLASTN on recently identified virus datasets. In dataset 4, L1 and L2 denote layer 1 and layer 2, respectively.

In all 4 datasets, RNAVirHost achieved better or comparable performance than BLASTN, as shown in Fig. 6. The first dataset consists of 21 viruses, whose hosts are from 6 orders under Magnoliopsida plants [50–56]. RNAVirHost predicted 1 case to be fungi-associated, which is a member of *Alphapartitivirus*, a genus infecting both plants and fungi. This may suggest that the query has the ability to infect fungi. The second dataset includes 15 viruses, infecting hosts of 4 Arthropoda orders [57–60]. RNAVirHost achieved a high accuracy of 93.3% while BLASTN got 40%. The only misclassified query obtained a balanced prediction score of RNAVirHost between Chordata and Invertebrate, which implied its potential to infect vertebrates. The third dataset involves 69 viruses from 9 orders, and their hosts span 6 fungi orders [61–67]. RNAVirHost achieved an accuracy of 76.8%, outperforming BLASTN by 28.6%. Finally, the fourth dataset comprises 21 viruses from 6 orders, infecting seahorses and salmon [68, 69]. We achieved an accuracy of 76.2% and 57.1% in layer 1 and layer 2, respectively, outperforming BLASTN by 28.6% and 33.3%.

## Discussion

The application of metagenomic sequencing promotes the identification of novel viruses from host-associated and environmental samples of a diverse set of ecosystems. However, the high diversity of the potential hosts, the sampling method and location, and the heterogeneity of metagenomic sequencing make it hard to determine hosts of the detected viruses, which is a critical step for pandemic surveillance and One Health. In this study, we compared various features used in host prediction of RNA viruses and developed a tool, RNAVirHost, that allows a fast and reliable host prediction by only using the virus sequences. Compared to the laborious and expensive process of host verification in the wet lab, RNAVirHost offers a time- and resource-effective strategy. By integrating genomic traits and sequence homology of viruses, RNAVirHost achieved higher accuracy than the alignment-based method. Instead of focusing on vertebrate-associated viruses, we extend the host range to plants, fungi, and bacteria. With the increasing availability of viruses’ host annotations, RNAVirHost can be easily scaled to more viruses and hosts.

Despite the potential implications of our study, it is important to acknowledge the challenges that lie ahead for future research in this field. Two particular challenges warrant careful consideration: the unexpected host switch and the existence of multiple hosts. The long-term coevolution between hosts and viruses may shape the genomic traits of viruses in a way that enables the distinction of their hosts. However, when viruses transfer to new hosts, their genome heritage may no longer reflect their host tropism, and the selection force may not immediately reveal their potential. To anticipate the phenomenon of host switching, additional information is required, including the host lifestyle, virus infection patterns, and virus–host protein–protein interactions. Obtaining such information can provide a more comprehensive understanding of the factors influencing host switching and improve our ability to forecast and respond to pandemics.

**Figure 7:**
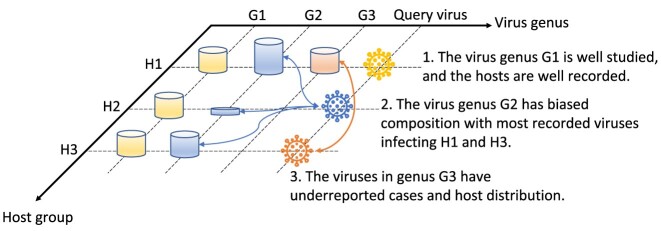
A visualization illustrating the potential sequencing bias in the reference database, posing a challenge to the prediction of hosts. On the virus genus axis, there are 3 reference genera represented by colors: yellow for G1, blue for G2, and red for G3. The host group axis consists of 3 distinct host groups (H1, H2, H3) present in the reference database. The cylinders at the intersections of the dashed lines represent viruses belonging to the respective genera that infect the corresponding host groups. The height of each cylinder indicates the relative number of viruses. G1 is extensively studied, and its hosts are well documented. G2 and G3 are understudied, resulting in limited information about their hosts.

Turning our attention to the second challenge, we must address the inherent limitations of RNAVirHost, which is designed to predict the viruses’ hosts. In specific cases where viruses can infect multiple hosts, it requires more comprehensive analysis, like single nucleotide variants, to draw the host range accurately. On the other hand, due to the sequencing bias in the database, there exists a knowledge gap that some potential hosts of detected viruses have yet to record. We discussed how the sequencing bias toward different hosts can lead to misclassification, as shown in Fig. 7. Our focus was primarily on the genus-level analysis. To account for the sequencing bias, we categorized virus genera into 2 groups: well-recorded cases and underreported cases. Well-recorded cases refer to virus genera that have been exhaustively sequenced in all of their hosts without any sequencing bias, represented by virus genus G1. The availability of a complete reference database greatly benefits the host prediction of new queries. On the contrary, the underreported cases depict the virus genera whose diversity is understudied among their hosts, such as the virus genera G2 and G3. In instances where the query virus infects a host that is underreported in the reference database, the prediction relies heavily on the available hosts and thus may be biased toward hosts that have been extensively recorded. For example, if there is a virus from genus G3 infecting host group H3, but the host recorded in the reference database is H1, there is a high possibility that it will be misclassified as infecting H1. Therefore, the incomplete host range record can negatively impact the accuracy of host prediction. To mitigate this bias, it is crucial to continue expanding and updating the reference database by including data from underreported hosts. Further understanding of these viruses will enhance the prediction and help to determine the boundary of infection.

## Availability of Source Code and Requirements

- Project name: RNAVirHost
- Project homepage: https://github.com/GreyGuoweiChen/VirHost
- Operating system(s): Platform independent
- Programming language: Python
- Other requirements: Python 3.8, BLAST 2.12.0+, Prodigal 2.6.3+, xgboost 2.0.3, pandas 2.0.3, biopython 1.83, numpy 1.23.5
- License: MIT license
- RRID: SCR_025061

## Supplementary Material

giae059_GIGA-D-24-00081_Original_Submission

giae059_GIGA-D-24-00081_Revision_1

giae059_Response_to_Reviewer_Comments_Original_Submission

giae059_Reviewer_1_Report_Original_Submission Mohammadali Khan Mirzaei -- 4/2/2024 Reviewed

giae059_Reviewer_1_Report_Revision_1 Mohammadali Khan Mirzaei -- 6/4/2024 Reviewed

giae059_Reviewer_2_Report_Original_Submission Michael Roach -- 4/10/2024 Reviewed

giae059_Reviewer_2_Report_Revision_1 Michael Roach -- 6/7/2024 Reviewed

giae059_Reviewer_3_Report_Original_Submission Vijini Mallawaarachchi -- 4/12/2024 Reviewed

giae059_Supplemental_Files

## Data Availability

All supporting data and materials are available in the *GigaScience* repository, GigaDB [70].

## Additional Files

**Supplementary Fig. S1**. Host prediction performance (F1 score) of different feature sets in layer 1 (A) and layer 2 (B). Host prediction performance (accuracy) of different feature combinations in layer 1 (C) and layer 2 (D). X-axis: Virus orders sorted by size (from largest to smallest).

**Supplementary Fig. S2**. The accuracy of models using genomic traits in the layer 1 when considering a different number of codon pair bias.

**Supplementary Fig. S3**. The accuracy distribution of models using different feature sets across virus orders. To demonstrate the effectiveness of our method, we evaluated the median accuracy of different models using Wilcoxon test. Specifically, we conducted 1-sided Wilcoxon tests to compare our method with BLASTN (P1) and Bias_Blast (P2). A small *P*-value (*P*  $< $ 0.05) suggests strong evidence to support the alternative hypothesis that the median accuracy of our method is significantly larger than that of the compared methods.

**Supplementary Fig. S4**. The order-wise accuracy of models using different learning architectures with the same features as RNAVirHost.

**Supplementary Fig. S5**. The Precision-Prediction_rate curve of RNAVirHost and BLASTN in 9 virus orders that contain more than 500 viruses. A curve closer to the upper-right corner indicates better model performance. The blue dot represents RNAVirHost’s performance when applying the empirical prediction score cutoff. The red dot represents the performance of BLASTN’s final prediction using the best alignment strategy. The prediction rate is lower than 100% due to the inability to align some query sequences with the reference sequences. Diff denotes the precision difference between RNAVirHost and BLASTN at the same prediction rate.

**Supplementary Fig. S6**. Host prediction performance (order-wise F1 score) of different feature sets in layer 1 (A, B) and layer 2 (C, D). X-axis: The completeness of viral sequences. Y-axis: The order-wise F1 score of the corresponding feature set.

**Supplementary Table S1**. The feature contribution of genomic traits to host prediction was assessed using XGBoost models across different virus orders. The contribution is measured as the feature importance. The fold between the observed and expected importance is calculated as the ratio between the mean of importance and the reciprocal of the total number of genomic traits (4,041). A higher fold value indicates greater importance demonstrated by the feature.

**Supplementary Information**. Section 1: Details of data collection. Section 2: The description of benchmark features. Section 3: Feature selection. Section 4: The trade-off between prediction rate and precision.
