# Supplementary material for: RNAVirHost: a machine learning–based method for predicting hosts of RNA viruses through viral genomes
Source: Gigascience. 2024 Aug 22;13:giae059. doi: 10.1093/gigascience/giae059 (PMC11340644; doi:10.1093/gigascience/giae059)
Supplement: giae059_GIGA-D-24-00081_Original_Submission [file giae059_giga-d-24-00081_original_submission.pdf]

GigaScience

VirHost: a machine learning-based method for predicting reservoir hosts of RNA viruses through viral genomes

--Manuscript Draft--

**Manuscript Number:**	GIGA-D-24-00081
**Full Title:**	VirHost: a machine learning-based method for predicting reservoir hosts of RNA viruses through viral genomes
**Article Type:**	Research
**Funding Information:**	Research Grants Council of Hong Kong (11206819, 11217521) — Dr. Yanni Sun<br>Natural Science Foundation of Hainan Province (324CXTD435) — Dr. Jingzhe Jiang
**Abstract:**	Background: The advent of high-throughput sequencing technologies has revolutionized the identification of novel RNA viruses in host-associated or environmental samples. Given that viruses are infectious agents, identifying the reservoir hosts of these new viruses carries significant implications for public health and provides valuable insights into the dynamics of the microbiome. However, determining the hosts of these newly discovered viruses is not always straightforward, especially in the case of viruses detected in environmental samples. Even for host-associated samples, it is not always correct to assign the sample origin as the host of the identified viruses. The process of assigning reservoirs to RNA viruses remains challenging due to their high mutation rates and vast diversity.<br>Result: In this study, we introduce VirHost, a machine learning-based tool that predicts the reservoirs of RNA viruses solely based on viral genomes. VirHost is a hierarchical classification framework that predicts reservoirs at different taxonomic levels. We demonstrate the superior accuracy of VirHost in predicting reservoirs of RNA viruses through comprehensive comparisons with various state-of-the-art techniques. When applying to viruses from novel genera, VirHost achieved the highest accuracy of 84.3%, outperforming the alignment-based strategy by 12.1%.<br>Conclusions: The application of machine learning models has proven beneficial in predicting reservoirs of RNA viruses. By integrating genomic traits and sequence homologies, VirHost provides a cost-effective and efficient strategy for reservoir prediction. We believe that VirHost can greatly assist in RNA virus analyses and contribute to pandemic surveillance.
**Corresponding Author:**	Yanni Sun<br>City University of Hong Kong<br>Hong Kong, HONG KONG
**Corresponding Author Secondary Information:**	
**Corresponding Author's Institution:**	City University of Hong Kong
**Corresponding Author's Secondary Institution:**	
**First Author:**	Guowei Chen, Ph.D.
**First Author Secondary Information:**	
**Order of Authors:**	Guowei Chen, Ph.D.<br>Yanni Sun<br>Jingzhe Jiang
**Order of Authors Secondary Information:**	
**Additional Information:**	
**Question**	**Response**

Powered by Editorial Manager® and ProduXion Manager® from Aries Systems Corporation

Are you submitting this manuscript to a special series or article collection?	No
**Experimental design and statistics**<br><br>Full details of the experimental design and statistical methods used should be given in the Methods section, as detailed in our Minimum Standards Reporting Checklist. Information essential to interpreting the data presented should be made available in the figure legends.<br><br>Have you included all the information requested in your manuscript?	Yes
**Resources**<br><br>A description of all resources used, including antibodies, cell lines, animals and software tools, with enough information to allow them to be uniquely identified, should be included in the Methods section. Authors are strongly encouraged to cite Research Resource Identifiers (RRIDs) for antibodies, model organisms and tools, where possible.<br><br>Have you included the information requested as detailed in our Minimum Standards Reporting Checklist?	Yes
**Availability of data and materials**<br><br>All datasets and code on which the conclusions of the paper rely must be either included in your submission or deposited in publicly available repositories (where available and ethically appropriate), referencing such data using a unique identifier in the references and in the "Availability of Data and Materials" section of your manuscript.<br><br>Have you have met the above requirement as detailed in our Minimum	No

Powered by Editorial Manager® and ProduXion Manager® from Aries Systems Corporation

Standards Reporting Checklist?	
If not, please give reasons for any omissions below.<br><br>as follow-up to "**Availability of data and materials**<br><br>All datasets and code on which the conclusions of the paper rely must be either included in your submission or deposited in publicly available repositories (where available and ethically appropriate), referencing such data using a unique identifier in the references and in the "Availability of Data and Materials" section of your manuscript.<br><br>Have you have met the above requirement as detailed in our Minimum Standards Reporting Checklist?<br><br>"	We will submit the analytic code and reference data to GigaDB when it is allocated to us.

LaTeX - Main Document (TeX file)

Click here to access/download;LaTeX - Main Document (TeX file);main.tex

Placeholder for OUP logo oup.pdf

Placeholder for journal logo gigascience-logo.pdf

GigaScience, 2023, 1–11

doi: xx.xxxx/xxxx
Manuscript in Preparation
Research

RESEARCH

VirHost: a machine learning-based method for predicting reservoir hosts of RNA viruses through viral genomes

Guowei Chen[1], Jingzhe Jiang[2] and Yanni Sun[1,*]

[1]Department of Electrical Engineering, City University of Hong Kong, Hong Kong (SAR), China and [2]Key Laboratory of South China Sea Fishery Resources Exploitation & Utilization, Ministry of Agriculture and Rural Affairs, South China Sea Fisheries Research Institute, Chinese Academy of Fishery Sciences, Guangzhou 510300, China

[*]Correspondence address. Department of Electrical Engineering, City University of Hong Kong, 83 Tat Chee Avenue, Kowloon, Hong Kong (SAR), China. E-mail: yannisun@cityu.edu.hk

Abstract

Background: The advent of high-throughput sequencing technologies has revolutionized the identification of novel RNA viruses in host-associated or environmental samples. Given that viruses are infectious agents, identifying the reservoir hosts of these new viruses carries significant implications for public health and provides valuable insights into the dynamics of the microbiome. However, determining the hosts of these newly discovered viruses is not always straightforward, especially in the case of viruses detected in environmental samples. Even for host-associated samples, it is not always correct to assign the sample origin as the host of the identified viruses. The process of assigning reservoirs to RNA viruses remains challenging due to their high mutation rates and vast diversity.
Results: In this study, we introduce VirHost, a machine learning-based tool that predicts the reservoirs of RNA viruses solely based on viral genomes. VirHost is a hierarchical classification framework that predicts reservoirs at different taxonomic levels. We demonstrate the superior accuracy of VirHost in predicting reservoirs of RNA viruses through comprehensive comparisons with various state-of-the-art techniques. When applying to viruses from novel genera, VirHost achieved the highest accuracy of 84.3%, outperforming the alignment-based strategy by 12.1%.
Conclusions: The application of machine learning models has proven beneficial in predicting reservoirs of RNA viruses. By integrating genomic traits and sequence homologies, VirHost provides a cost-effective and efficient strategy for reservoir prediction. We believe that VirHost can greatly assist in RNA virus analyses and contribute to pandemic surveillance.

Key words: RNA virus; host prediction; reservoir; machine learning; metagenomics

Introduction

Viruses are obligate intracellular parasites that depend on living organisms for their replication and survival. RNA viruses, possessing RNA as their genetic material, have the capability to infect a diverse array of organisms. For example, several types of RNA viruses are causal agents of the most disastrous pandemics in human history, including COVID-19, SARS, the annual influenza, etc. Furthermore, certain plant and animal RNA viruses pose a threat to agricultural and animal sectors, jeopardizing crop growth, livestock health, and subsequently leading to substantial economic losses in agriculture and animal husbandry. Besides eukaryotic hosts, some RNA viruses can also infect bacteria and thus directly affect the dynamics of microbiome [1].

Understanding the interaction between viruses and their hosts is a fundamental step in characterizing the viruses' roles in public health, animal husbandry, agriculture, etc. While known RNA viruses often have annotated hosts, many newly discovered viruses

Compiled on: March 7, 2024.
Draft manuscript prepared by the author.

lack the host information. Viruses are believed to be the most diverse and abundant biological entities in the world [2]. Currently, metagenomic sequencing has emerged as the primary approach for the discovery of novel viruses, as it eliminates the need for virus isolation and cultivation in laboratory settings. This method involves directly sequencing genetic material from host associated or environmental samples, allowing for the identification of viruses present within these complex ecosystems. A series of studies has been conducted to identify novel viruses in various ecosystems [3, 4, 5].

While the application of metagenomic high-throughput sequencing technologies have facilitated the discovery of the viral dark matter [6], how to determine the reservoir hosts of the metagenome-originated viruses remains challenging owing to the complex composition of the metagenomic sequencing samples. Regardless of the various definitions of reservoir hosts [7, 8], in this context, we highlight the natural hosts that allow the persistence of the viruses, instead of the circulation or transmission. In some cases, viruses may be detected in non-host organisms due to the symbiotic relationship, the dietary interaction, or the physical contact, like the bacteria-infecting viruses and the plant-associated viruses found in bird digestive tract [9] and the plant-infecting viruses found in insect vectors [10]. These carriers are not the primary focus of our study.

Traditionally, the host verification requires stringent experimental contribution, including the isolation of viral particles from hosts of interests, the serological tests, the epidemiological investigation, and virus phylogenetic analyses. These processes are time-consuming, labor-intensive, and often require specialized equipment and expertise. While the metagenomic sequencing is becoming the main source of the novel viruses, the heterogeneous composition made it harder to determine the target hosts. Therefore, when the novel viruses rapidly emerge, predicting the hosts from the virus genome sequences, avoiding the tedious laboratory steps, show its attractive advantage in terms of economy and efficiency.

By far, a number of computational works have been conducted to explore the association between viruses and the potential hosts. While RNA viruses dominate the eukaryotic viruses, the majority of prokaryotic viruses are DNA viruses. Due to the extensive host range of RNA viruses and the limited availability of reference genomes for potential eukaryotic hosts, the development of computational tools for predicting hosts of prokaryotic viruses is faster than the development of host prediction tools for eukaryotic viruses. To predict the hosts of phages (viruses infecting prokaryotes), VPF-Class classified a set of Viral Protein Families (VPFs) and aligned the queries virus to the categorized references [11]. RaFAH generated protein clusters, constructed profile Hidden Markov Models (pHMMs), and trained a random forest classification model using the pHMMs alignment score [12]. DeepHost encoded the spaced k-mer feature by a three-dimensional matrix and trained a convolutional neural network (CNN) to predict the hosts [13]. CHERRY integrated various signals, including gene organization, CRISPR, sequence similarity, and k-mer usage, and predicted the virus-host association by a graph convolutional encoder and decoder [14]. Currently, these tools allow host prediction at different ranks and the accuracy decreases with more refined host range (e.g. from class to species). Nevertheless, these tools are limited to host prediction for prokaryotic viruses.

Compared to the extensive studies on phages, the host prediction of RNA viruses remains challenging and arduous. The typical genomes of RNA viruses, ranging from 3kbp to 41kbp [15], is smaller than that of DNA viruses (5kbp to 600kbp) and has limited capacity to carry host tropism signals [16]. While the sequence matches between phages and the prokaryotic genomes facilitate the host prediction of phages, they are less common in RNA viruses [1, 17]. Furthermore, while many bacterial genomes have been sequenced with metagenomic sequencing, the extensive host range of RNA viruses, limited availability of the potential hosts, and the very large sizes of the potential host genomes make adding host genome features very difficult. Finally, the high mutation rate of RNA viruses makes the genomes less conserved, so that the existing achievement can not be extend to the novel viruses.

With these challenges, the computational frameworks still show outstanding performance in two host prediction scenarios. The first is to predict the host for a specific group of RNA viruses. Raj et al. [18] counted the spaced amino acid k-mer frequency and trained an Alternating Decision Tree classifier for two families, Picornaviridae and Rhabdoviridae. Eng et al. [19] encoded the protein sequences by the physical and chemical property of the amino acid and trained a random forest for the influenza A virus. Mock et al. [20] developed two deep neural network models for the host classification of three viruses, respectively (influenza A virus, rabies lyssavirus, and rotavirus A). These viruses associated closely with human's activity and thus attracted attention.

In another scenario, the researchers discuss whether the query viruses will infect the targeted host group, particularly human and mammals. Zhang et al. [21] leveraged the k-mer frequency and designed a k-nearest neighbor model to discriminate the human-infecting viruses from other viruses. Bartoszewicz et al. [22] applied the reverse-complement neural networks to do read-based prediction of the viral host (human or non-human). Pandit et al. [23] investigated the host sharing network of mammalian viruses and trained gradient boosting models to predict the host sharing situation of two viruses. Zhang et al. [24] generated a set of protein families that commonly shared by mammals, studied the correlation between these proteins and the cross-species transmission, and trained a random forest model to predict the transmission of viruses.

However, these two categories of works are hard to extend to the host prediction of metagenome-assembled RNA viruses. They overlooked the broader host range of RNA viruses, including additional host candidates, like plants, invertebrates, and fungi. To predict hosts of the increasing novel RNA viruses, some primary explorations have been made. Babayan et al. [25] investigated the genomic traits and the sequences homologs of viruses, and developed a classification model considering viruses from 12 taxonomic groups and 11 reservoir groups. Building upon Babayan's study, Lee et al. [26] further evaluated the application of Machine learning with digital signal processing-based Structural Patterns (M-SP) of viruses in reservoirs prediction. Young et al. [27] assessed the gene content and the frequency of short sequences, and developed a hierarchical host classification framework based on support vector machine. Guo et al. [28] trained a two-branch convolutional neural network to capture the informative motifs and classified the viruses into five host groups. Despite the promising results obtained from these validations, these studies still face the challenge of limited viruses and host ranges.

In this work, we concentrate on predicting reservoirs of emergent novel viruses and thus developed a hierarchical host classification framework, VirHost. Combining virus taxonomy, genomic traits, and sequence homologies, VirHost allows predicting the reservoirs using only viral genomes. To cover as many viruses and reservoirs as we can, VirHost accepts queries from over 30 virus orders, and includes five host types in its first layer, including Chordata (Vertebrate), Invertebrate, Plant, Fungi, Bacteria. After obtaining the prediction results in the first layer, VirHost will perform additional predictions in the second layer, to obtain more precise host classification information. By evaluating various features and learning architectures in a more comprehensive database, we demonstrated the outstanding performance of VirHost in reservoir prediction of RNA viruses. We also evaluated VirHost's performance on novel viruses by conducting leave-one-taxon-out experiments. The results of these experiments demonstrated that VirHost can be effectively applied across the diverse landscape of viruses, without being limited to specific viral types.

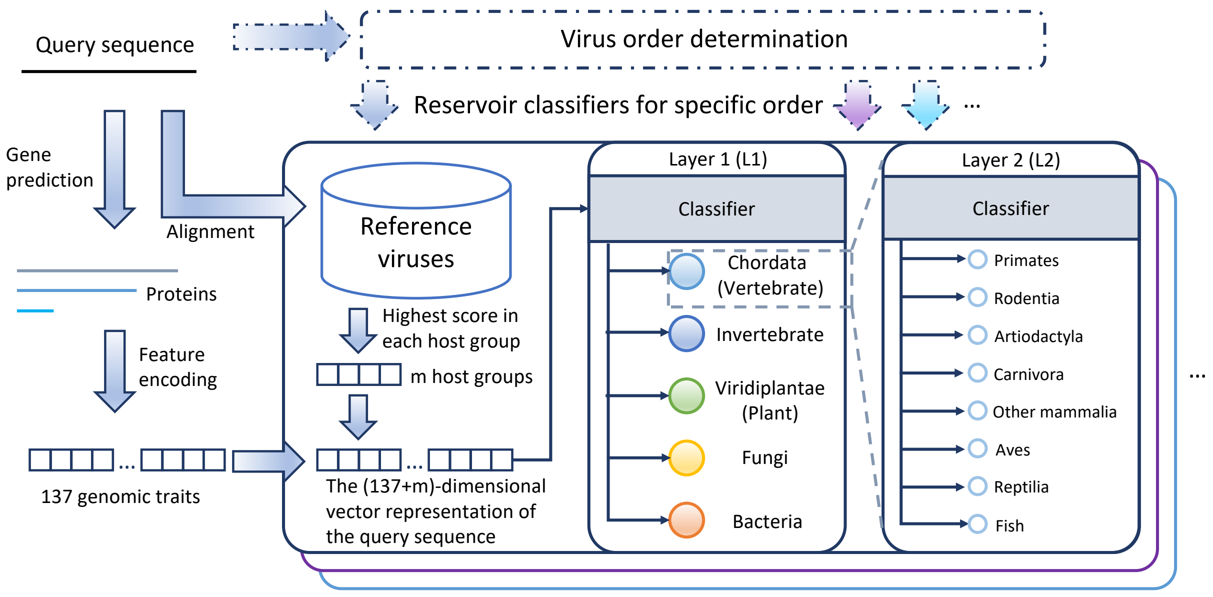

Figure 1. The framework of VirHost. The hierarchical host prediction consists of two classification layers, Layer 1 (L1) and Layer 2 (L2). In Layer 1, we predict hosts at kingdom and phylum level, including Chordata, Invertebrate, Viridiplantae, Fungi, Bacteria. In Layer 2, we further predict the specific host groups under Chordata at class and order level. The genomic traits consist of the usage preference of nucleotide, dinucleotide, codon, and amino acid. We categorized the reference viruses based on their hosts, and m denotes the number of host groups (output labels) in the corresponding classifiers.

Method

Overview of the method

The framework of VirHost is depicted in Fig. 1. Initially, we categorize the queries based on their taxonomic information to narrow down the potential host range. Then, we extract two types of features, genomic traits and sequence homology, for reservoir prediction using a learning-based model. This two-step approach incorporates both the taxonomic information and the potential host signal in the sequences and is anticipated to enhance the accuracy of our predictions with greater confidence.

The origin and evolution of RNA viruses remain complicated and puzzling [5]. Different groups of viruses have distinct infection mechanisms and divergent host ranges. To benefit the reservoir prediction, we incorporated prior virus taxonomic knowledge in VirHost to mitigate potential interference among viruses originating from different sources. While the lower taxonomic rank may provide a precise host range, the emergent novel sequences may not fit neatly into the existing taxonomic label. Given the trade-off between accommodating more candidates and requiring informative taxonomic knowledge, we categorized viruses into 30 orders following ViralZone [29]. The measure is also expected to improve the learning performance by avoiding imbalance sizes of different virus orders within the same host groups. Then, we built independent classifiers for every order by leveraging the virus members' genomic traits and sequence homologies.

VirHost predicts the reservoirs of queries hierarchically. The models are of two-layer tree structures, with the first layer corresponding to the host's kingdom and phylum level, and the second layer to the host's class and order level (Section. Labels screening). Once input to corresponding models, the query will be encoded as its genomic traits and sequence homologies (Section. Feature Encoding). VirHost will output the reservoir labels along the tree. Based on comprehensive benchmarks, eXtreme Gradient Boosting (XGBoost), a scalable machine learning technique, delivered the top performance, making it the preferred choice as the default architecture for VirHost. When more data are available, VirHost can be easily scaled to include the new members.

Data preprocessing

Data collection

We collected 6,735 viruses from Virus-Host Database [30] and 126,417 records with host annotations from NCBI GenBank. To remove redundancy, we used CD-HIT [31] to de-replicate the identical sequences. Then we check the host annotations using NCBI Taxonomy database [32] and manual validation. Host tags are renamed to the corresponding scientific names, while the ambiguous host annotations were further removed. The details can be found in the Supplementary Information (section 1).

Label screening

Although numerous RNA viruses has been found, the current database is biased towards human and mammalian-associated viruses. To better predict the host lineage, we carefully curated the host labels based on both the host phylogenetic tree and the data availability. For each virus order, we built a two-layer host phylogenetic tree. Layer 1 contains 5 branches (Chordata, Invertebrate, Viridiplantae, Fungi, Bacteria), which are categorized into kingdom and phylum level. Layer 2, designed for Chordata subtree, has 10 leaves, which are at the class and order level. Hence, we hierarchically predict the host lineage along the tree. This hierarchical partition acknowledges the practical consideration, that host switch phenomena are more frequently observed at the hosts' class and order level [33, 34], and offers users the flexibility to use both layers or one layer of host prediction of VirHost.

Some host labels have only a few recorded infecting viruses and thus are not ready for computational host prediction. For virus orders containing more then 30 viruses, we set the threshold as 10 and only keep host labels with at least 10 infecting viruses. After removing those "rare" host labels, the dataset contains 14,500 viruses and spans 30 virus orders. Their host distribution is shown in Table 1.

Feature Encoding

Previous studies have extensively explored various features of RNA viruses with different hosts. Both the genomic traits and viruses' sequence homologies facilitated the host prediction of RNA viruses.

Table 1. The virus order and host distribution after the label screening.

		Layer 1					Layer 2									
order	num	Chordata	Invertebrate	Viridiplantae	Fungi	Bacteria	Primates	Rodentia	Carnivora	Artiodactyla	Chiroptera	Other Mammalia	Aves	Reptilia	Amphibia	Fish
Ortervirales	2764	92.0%	-	8.0%	-	-	85.9%	0.9%	0.9%	2.0%	-	0.7%	1.6%	-	-	-
Picornaviral	2647	74.2%	12.2%	13.6%	-	-	40.2%	4.1%	4.2%	14.5%	2.6%	1.7%	3.9%	0.9%	-	2.0%
Bunyavirale	1524	52.4%	31.6%	15.2%	0.9%	-	18.0%	17.6%	-	1.6%	1.4%	5.3%	1.7%	5.6%	-	1.2%
Tymovirales	1042	-	2.6%	94.3%	3.1%	-	-	-	-	-	-	-	-	-	-	-
Reovirales	1034	52.9%	33.8%	9.7%	3.6%	-	9.9%	1.3%	2.9%	9.0%	5.5%	3.6%	9.6%	1.3%	-	10.0%
Amarillovira	817	85.2%	14.8%	-	-	-	54.0%	9.7%	-	10.0%	4.0%	4.2%	1.7%	-	-	1.6%
Mononegav	758	57.5%	27.8%	11.3%	3.3%	-	11.3%	6.5%	4.0%	5.4%	11.5%	2.4%	9.0%	1.6%	-	5.9%
Martelliviral	670	5.1%	6.3%	73.7%	14.9%	-	5.1%						-	-	-	-
Nidovirales	622	94.4%	5.6%	-	-	-	4.8%	5.3%	5.6%	36.3%	17.4%	5.6%	13.5%	5.8%	-	-
Patatavirale	558	-	-	100.0%	-	-	-	-	-	-	-	-	-	-	-	-
Ghabrivirale	393	-	14.0%	9.9%	76.1%	-	-	-	-	-	-	-	-	-	-	-
Durnavirale	340	5.0%	-	32.4%	62.6%	-	5.0%						-	-	-	-
Stellavirales	296	100.0%	-	-	-	-	10.1%	12.8%	8.4%	33.1%	5.1%	-	16.2%	-	4.4%	9.8%
Tolivirales	226	-	15.0%	73.9%	11.1%	-	-	-	-	-	-	-	-	-	-	-
Hepelivirale	181	80.1%	10.5%	9.4%	-	-	32.0%	16.6%	-	16.6%	-	8.8%	6.1%	-	-	-
Sobelivirale	120	-	12.5%	87.5%	-	-	-	-	-	-	-	-	-	-	-	-
Blubervirale	108	100.0%	-	-	-	-	75.9%	-	-	-	14.8%	-	9.3%	-	-	-
Cryppavirale	80	-	-	-	100.0%	-	-	-	-	-	-	-	-	-	-	-
Articulaviral	77	100.0%	-	-	-	-	59.7%						16.9%	-	-	23.4%
Jingchuvira	61	-	100.0%	-	-	-	-	-	-	-	-	-	-	-	-	-
Nodamuvir	42	-	100.0%	-	-	-	-	-	-	-	-	-	-	-	-	-
Ourlivirales	38	-	-	26.3%	73.7%	-	-	-	-	-	-	-	-	-	-	-
Wolframvir	23	-	-	-	100.0%	-	-	-	-	-	-	-	-	-	-	-
Mindivirales	22	-	-	-	-	100.0%	-	-	-	-	-	-	-	-	-	-
Norzivirales	21	-	-	-	-	100.0%	-	-	-	-	-	-	-	-	-	-
Serpentovir	16	-	-	100.0%	-	-	-	-	-	-	-	-	-	-	-	-
Muvirales	9	-	100.0%	-	-	-	-	-	-	-	-	-	-	-	-	-
Yadokarivir	7	-	-	-	100.0%	-	-	-	-	-	-	-	-	-	-	-
Goujianviral	3	-	100.0%	-	-	-	-	-	-	-	-	-	-	-	-	-
Timlovirales	1	-	-	-	-	100.0%	-	-	-	-	-	-	-	-	-	-
sum	14500															

Each row represents the host distribution of a virus order. While the column "num" shows the total number of viruses in the order, the following columns represent the percent of viruses infecting the corresponding hosts. Layer 2 consists of the Chordata subgroups from Layer 1, therefore the sum of values in the second layer is equal to the value of "Chordata". In cases (Martellivirales, Durnavirales, Articulavirales) where mammalian viruses are less than 50, we merge mammalian members into a single node, Mammalia.

VirHost relies on a subset of the genomic traits and the sequence homology. A widely accepted hypothesis is that the biases in genomic composition, also named as genomic trait, may hinted the natural selection pressure imposed by their hosts. To escape host immune responses and hijack the cellular machinery, viruses tend to mimic the genomic trait usage of their hosts [35, 36]. It is reported that Flaviridae viruses associate with two host groups, vertebrate and invertebrate. The members infecting a single group have similar dinucleotide and codon preference as their hosts do [37]. Besides, the changes of codon pair bias were proven to influence the viruses' pathogenicity [38], showing that the host tropism potentially relates to the the genomic trait. To represents the genomic feature, we translate the query sequences to proteins using MetaProdigal [39] and generate a 137-dimensional vector $\mathbf{S} \in \mathbb{R}^{137}$, where S_i quantifies the preference of 137 genomic traits, including the usage preference of nucleotide (Eq. 1), dinucleotide (Eq. 2), codon (Eq. 3), and amino acid (Eq. 4).

On the other hand, related viruses tend to infect hosts that share taxonomic associations or have overlapping activity patterns. Thus, the viruses' sequence homology may indicate their host range. The sequence homology is introduced by conducting sequence alignment. The reference sequences are categorized into different groups by their hosts, and we used BLASTN to get the maximum alignment scores of the query against every virus group. The maximum alignment scores against all groups are converted into a m-dimensional vector, $\mathbf{H} \in \mathbb{R}^{m}$, where m is the number of virus groups (host labels) in the corresponding classifier. Finally, the two vectors, **S** and **H**, are concatenated into a (137+m)-dimensional vector, $\mathbf{X} \in \mathbb{R}^{137+m}$, and used as the representation of the query. The combination of genomic traits and viral sequence homology is expected to facilitate predicting the reservoir hosts. Here, we briefly describe the different features and more details are depicted in the Supplementary Information section 2.

Features from sequence composition (genomic traits)

The sequence composition describes the relative abundance or occurrence of short strings of nucleotide or amino acid. By far, the k-mer frequency has been widely used in various tasks, like taxonomy classification [40], sequences annotation [41], and host prediction [13]. Based on the length, the k-mer frequency can be defined in different formats. Referring to the previous works on reservoir determination [25, 37], we define the composition by the following equations.

$$P_x = n_x / \sum_x n_x \tag{1}$$

$$P_{xy} = \frac{n_{xy} / \sum_{x,y} n_{xy}}{P_x * P_y} \tag{2}$$

$$P_{xyz} = \frac{n_{xyz}}{n_A} \tag{3}$$

where x, y, and z are nucleotide and the codon xyz encodes the amino acid A,

$$P_A = \frac{n_A}{\sum n_A} \tag{4}$$

$$CPS_{x_1y_1z_1,x_2y_2z_2} = \frac{n_{x_1y_1z_1x_2y_2z_2}}{n_{AB} * P_{x_1y_1z_1} * P_{x_2y_2z_2}} \tag{5}$$

where the codon $x_1y_1z_1$ encodes the amino acid A and the adjacent codon $x_2y_2z_2$ encodes B. The occurrence is denoted as n.

To implement normalization, any zero value or missing values are replaced with a small number (1e-4) as a default. and a log2 transformation is applied to all values. Generally, when the value is positive the corresponding feature is over-represented in the genome; otherwise, the feature is deem to be under-represented.

Features from sequence alignment

We introduced sequence alignment score as a feature vector of the query. Specifically, the reference sequences are categorized into different groups by their hosts and we aligned the query against the references. The highest alignment score of each group were kept as the potential association between the query and the corresponding host. Hence, the query will be represent as a vector of m*1, where

m is the number of host labels. If no alignment is found, we set the association to be one. A log10 transformation is applied to all scores. In this research, we used BLASTN as the default alignment tool. Instead of k-best matches, which tends to be affected by the data imbalance, we only consider the best alignment of each group.

Performance evaluation metrics

The collected 14,500 virus records belong to 30 virus orders. As described in Section Label screening, for each virus order, we examine their host label distribution. We found that 12 virus orders exclusively infected a specific host group, and we directly assigned host labels to them. These virus orders include Patatavirales, Cryppavirales, Jingchuvirales, Nodamuvirales, Wolframvirales, Mindivirales, Norzivirales, Serpentovirales, Muvirales, Yadokarivirales, Goujianvirales, and Timlovirales. The remaining 18 virus orders involved infections across multiple host groups. Among these 18 orders, thirteen received labels in the second layer of the host phylogenetic tree. Accordingly, we conducted host prediction and evaluation for the corresponding virus orders and host groups.

To evaluate the performance of VirHost, we employed various metrics, including accuracy, precision, and prediction rate. Accuracy serves as a fundamental metric, representing the proportion of correctly predicted queries out of the total number of queries. To provide a nuanced assessment of VirHost's performance across different taxonomic levels, we introduced family-wise accuracy and genus-wise accuracy, which are computed by averaging the accuracy of respective virus families and genera. Additionally, prediction rate quantifies the ratio of output predictions to the total number of queries, while precision captures the ratio of correctly predicted queries to the total number of output predictions. By employing these metrics, we aim to offer a comprehensive and granular evaluation of VirHost's performance, enabling a robust analysis of its predictive capabilities and strengths.

Result

We conducted comprehensive benchmark experiments on different scenarios to evaluate the performance of VirHost. First, we compared various feature sets and their combinations by 5 fold cross-validation. The comparison showed that the subset of genomic traits outperformed other features. We also assessed the features' contribution by machine learning strategies and validated the choice of genomic traits. We then evaluated different learning architectures, among which XGBoost achieved the highest accuracy and thus was chosen as the default architecture. Second, we focused on host prediction for novel RNA viruses. Specifically, we evaluated VirHost's performance on novel viruses by conducting leave-one-taxon-out experiments. The results of these experiments demonstrated that VirHost can be effectively applied across the diverse landscape of viruses, without being limited to specific viral types. Finally, to show the accuracy and utility of VirHost in real experiments, we collected some recently identified viruses by researches and tested VirHost's performance on different host groups.

Assessment via cross-validation

In this experiment, we follow the standard evaluation strategy in machine learning to examine the performance of different types of features and learning models. There are many types of features that may help host prediction. We first evaluated the different feature sets and their combinations by stratified 5-fold cross-validation. For each virus order, we stratified the virus into non-overlapping 5-fold by host labels, trained models using 4 out of 5 folds, and tested them in the remaining one. This assessement was performanced on each fold and we presented the overall performance on all 5 folds.

We tested 11 feature sets by ensemble learning, XGBoost. To begin with, we evaluated the classification validity of each feature set without combinations. Referring to Babayan [25], we regarded nucleotide preference, dinucleotide preference, codon usage, codon pair bias, and amino acid usage as one feature set, named as *"Bias"* here. Considering that most RNA virus are shorted than 50 kbp and there are 3,904 codon pairs (excluding pairs led by stop codons), which is a high-dimensional feature compared to the limited sequences records, we made a subset feature from Bias by excluding the codon pair bias, named as *"sBias"* (subset). Besides the bias-related features, we evaluated 9 more types of features, including BLASTN (best hit), digital signal processing-based Structural Patterns (M-SP), the frequency of 6-mer, 7-mer, 8-mer, amino acid 3-mer (AA3) , AA4, Physio-chemical 5-mer (PC5), and PC6 in [26, 27]. M-SP involved in generating the Fourier transformation (FT) of the biological sequences, computing the correlation coefficients among the FT, and obtaining the distances matrix of sequences through the correlation coefficients [26]. A detailed description of the benchmarked feature can be found in the Supplementary Information (section 2). Then, we assessed feature set combinations among Bias, BLASTN, and M-SP. DeepHoF [28], a host prediction tool based on convolutional neural network, provided the classification of viruses associated with plant, germ, invertebrate, human and other vertebrate, where germ include bacteria and fungi. We included it in the benchmark assessment in Layer 1 by merging "human" and "other vertebrate" to be Chordata.

As shown in Fig. 2 (A,B), when using each of the 11 feature sets, the classifier with feature *sBias* achieved the highest accuracy of 92.38% and 83.92% for each virus order in L1 and L2, respectively. The models with feature Bias and BLASTN rank the second and the third in performance. The model with *Bias* got accuracy of 90.89% and 82.14%, while using BLASTN got 89.64% and 81.57%. The accuracy difference between *sBias* and *Bias* drew our attention and we calculated the feature contribution to classification performance, as shown in the Supplementary Information (section 3), Table S1 and Fig. S1. The result indicated that most codon pair scores did not contribute to the prediction, which is consistent with previous studies [25]. This can be explained by the sparsity of the codon pair in RNA viruses. Besides, the M-SP traits, achieving accuracy of 87.14% and 76.93%, did not provide accurate predictions when used solely.

Out of the 11 feature sets, *Bias*, *sBias*, and BLASTN scores show promising results in the cross-validation experiment. Hence, we further evaluated their combinations and reported the results in Fig. 2 (C,D). When combined, the models with *sBias_Blast* achieved the best order-wise accuracy of 93.99% and 88.01%. The combination significantly improved the prediction accuracy. While *Bias_Blast* and *Bias_Blast_Dsp* (the combination of *Bias*, BLASTN, and M-SP as [26]) are the second and third best group, they got little difference in accuracy (93.14% and 86.98% for *Bias_Blast*, 93.14% and 86.96% for *Bias_Blast_Dsp*). The result implied that the M-SP traits did not benefit the prediction of RNA viruses' host significantly. On the other hand, the drops of accuracy from *sBias* to *Bias* and that from *sBias_Blast* to *Bias_Blast* validated the feature reduction that excludes the codon pair features.

An important observation is that using BLASTN achieves quite comparable host prediction results as our learning-based methods. We thus further analyzed this. First, to ensure that the improvements observed in the host prediction models are not due to chance events, we conducted a further comparison among models based on different feature sets. This comparison involved examining the accuracy distribution across virus orders, as detailed in Fig. S2. Through the one-sided Wilcoxon test, we demonstrated that the observed improvements in host prediction accuracy are statistically significant and not merely a result of random chance.

Second, it is worth mentioning that when employing a random data partitioning strategy (5-fold cross validation), it is not unex-

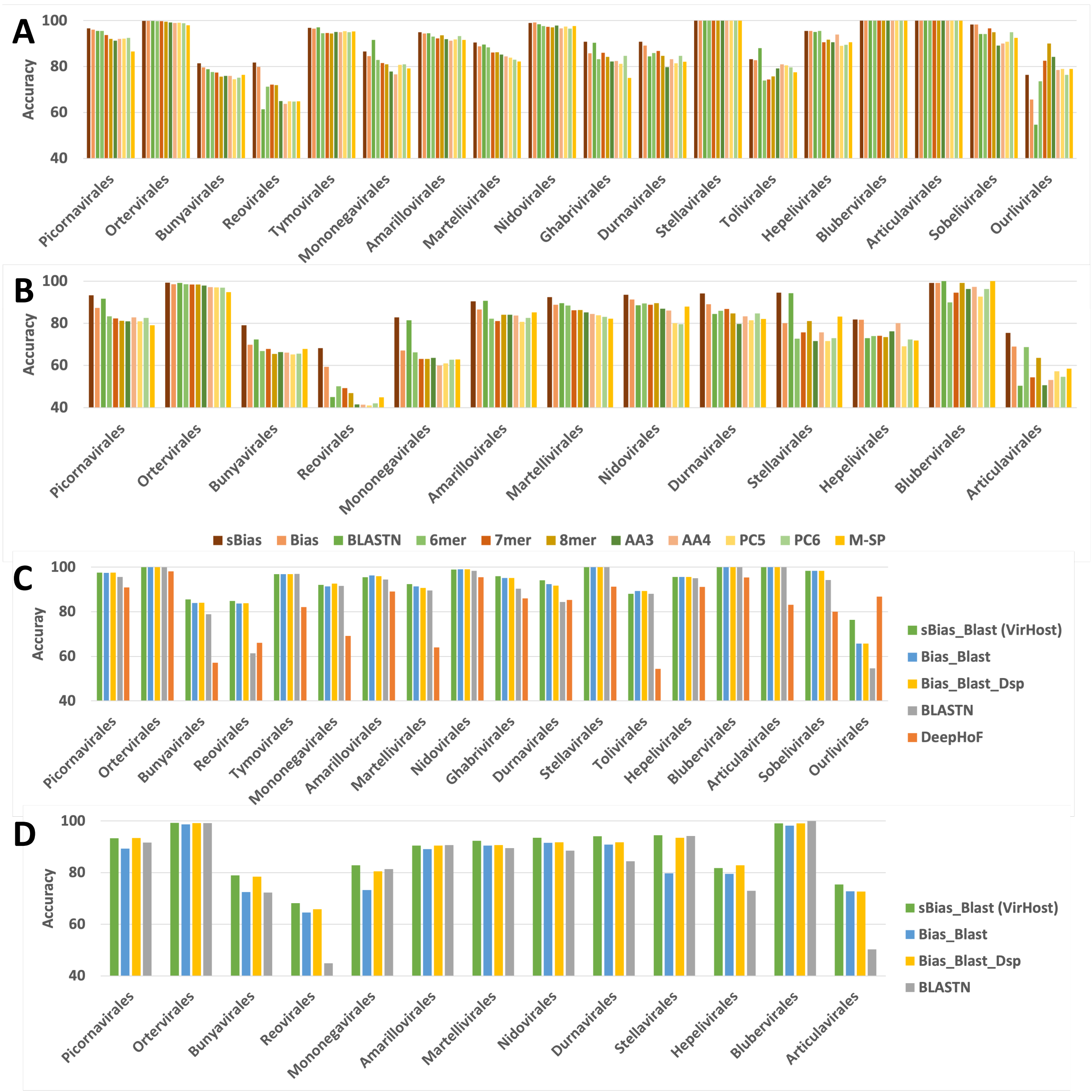

Figure 2. Reservoir prediction performance of different feature sets in Layer 1 (A) and Layer 2 (B). Here, Bias denotes a set of genomic traits, including nucleotide preference, dinucleotide preference, codon usage, codon pair bias, and amino acid usage, while sBias denotes a subset which excludes the codon pair bias. Reservoir prediction performance of different feature combinations in Layer 1 (C) and Layer 2 (D). Here, sBias_Blast is the combination of sBias and BLASTN. Bias_Blast is the combination of Bias and BLASTN. Bias_Blast_Dsp is the combination of Bias, BLASTN, and M-SP. DeepHoF is designed to predict hosts at the kingdom and phylum level (Layer 1), and its output is transformed to corresponding labels (Germ score to be Fungi; Human score and Vertebrate score to be Chordata). X-axis: virus orders sorted by the size (from largest to smallest). The performance comparison in Layer 2 considers the errors from both Layer 1 and Layer 2. Among the 18 orders, thirteen can be further classified in Layer 2. Therefore, we focused our performance evaluation solely on Layer 2 within these thirteen orders.

pected to observe that the predictive performance of BLAST yields similar results as learning-based methods. One main reason for this is that the test viruses, when randomly partitioned, tend to exhibit high similarity to certain viruses in the training data, resulting in a less challenging test scenario. Our next set of experiments will explore more realistic usage scenarios.

Handling Out-of-Distribution host labels

As our primary objective is to predict the natural hosts of metagenomic-assembled RNA viruses, we have designed a comprehensive label list that includes four eukaryotic kingdoms and one prokaryotic domain. However, considering the continuous emergence of novel RNA viruses, there are instances where the queried viruses may infect hosts outside the label list. To prioritize precision, we opt to reject virus queries that may not infect the target hosts, even if it leads to a lower prediction rate. To determine the trade-off between prediction rate and precision for both our tool and BLASTN, we analyzed the distribution of prediction scores, which is shown in Supplementary Information (section 4) and Fig. S3. Our analysis revealed that, while predicting hosts for the same number of queries, VirHost achieved higher precision compared to BLASTN. Consequently, we provide users with an empirical prediction score cutoff for each virus order, allowing them to choose predictions with greater confidence. This empirical approach enables users to obtain more reliable predictions.

The comparison among machine learning architectures

Finally, we conducted an evaluation of several learning architectures, encompassing XGBoost, Gradient Boosting Decision Tree (GBDT), Random Forest (RF), Support Vector Machine with RBF ker-

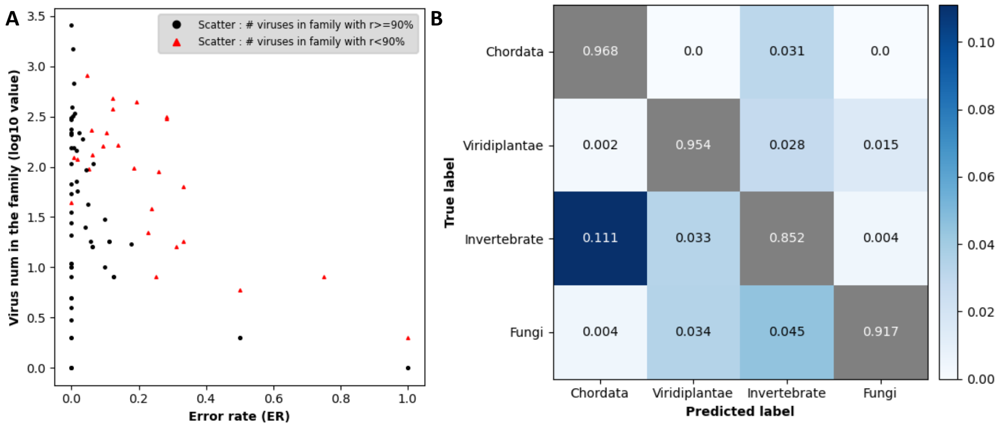

Figure 3. (A) The error rate distribution of VirHost across different virus families in Layer 1. The dots denote the number of virus members in each family (Y-axis) and its corresponding error rate (X-axis). (B) The confusion matrix of VirHost's prediction in Layer 1. The values denote the percentage of mis-classified members, which were normalized to total number of the corresponding groups. The viruses infecting bacteria are limited to specific virus orders, which exclusively infect bacteria. Hence, we do not visualize bacteria here.

nel (SVM), Logistic Regression (LR), K-Nearest Neighbors (KNN), and Gaussian Naive Bayes (GNB). Using the scikit-learn package [42], default parameters were employed to train these models, and their performance was assessed using accuracy as the evaluation metric. As demonstrated in Fig. S4, XGBoost exhibited the highest accuracy of 94.0% (L1) and 88.0% (L2), while the second best architecture (RF) achieved those of 93.3% and 87.5%, and the third best one (GBDT) got 92.7% and 86.2%.

Collectively, the model trained with XGBoost and the combination of *sBias* and BLASTN, achieved the outperforming accuracy among various learning architectures, feature sets, virus groups, and host ranks.

Family-wise analysis reveals the potential host switch

In comparison to viruses within the same order, viruses in the same family generally have more consistent host ranges. We investigated host distribution of each virus family and visualized the errors rate of virus families in the first layer in Fig. 3A. In the 18 evaluated virus orders, there are 83 families and 13,238 evaluated viruses. In the first layer, the family-wise average accuracy is 87.55%, meaning that 658 out of evaluated 13,238 viruses got wrong assignments of hosts. It is observed that the misclassification of VirHost mainly distributes to those virus families with mixed host groups. To better visualize the trend, we counted the dominant host labels in every family and the ratio of members infecting the dominant hosts, denoted as r. As a measure of homogeneity, r will have a small value if a family mainly contains viruses infecting different host groups. Otherwise, a virus family infecting only one dominant host group will get an r value close to 1. To examine the homogeneity values of various families, we set r as 90% as the threshold for high and low host homogeneity. Out of the 83 RNA virus families, there are 57 families with over 90% of their members infecting a unique host group ($r \geq 90\%$) and 26 families infecting multiple host groups ($r < 90\%$). In 658 error cases, only 57 (8.66%) came from the former ($r \geq 90\%$), while 593 (90.12%) belong to the latter case ($r < 90\%$), implying that these wrong prediction are mainly from viruses with multiple hosts across phylum. For instances, Peribunyaviridae (r = 56.7%), Phenuiviridae (r = 53%), Nairoviridae r = 70.8%) are virus families in Bunyavirales. They contains members that are well known for causing vector-boned diseases [43, 44, 45]. VirHost achieved accuracy of 71.34%, 70.67%, and 74.16% for the three families, which is lower than the family-wise average accuracy.

Additionally, we generated the confusion matrix to evaluate the predictions made by VirHost, as shown in Fig. 3B. Analysis of the matrix revealed that viruses infecting chordates and Viridiplantae (plants) could be accurately classified. Only 0.2% of viruses infecting Viridiplantae were misclassified as chordate-associated, and no viruses infecting chordates were predicted to infect Viridiplantae. Similarly, viruses infecting chordates and fungi could be clearly distinguished. However, misclassification predominantly occurred among invertebrate-associated queries, with 11.1% of invertebrate-infecting viruses predicted to infect chordates, and 3.1% of chordate-infecting viruses predicted to infect invertebrates. These predictions strongly suggest the presence of potential vector-borne viruses within the dataset. Additionally, errors occurred when distinguishing between viruses that infect invertebrates and those that infect plants and fungi. This confusion may arise due to the contact and dietary interactions between invertebrates and plants or fungi [46, 47]. Furthermore, there is some overlap between the viruses infecting fungi and those infecting plants, which could result from the symbiotic relationships between certain plants and fungi [48]. Although the focus of this work is placed on predicting the reservoir hosts, the vector-borne viruses still have a great influence on the performance of host prediction.

Host prediction for novel RNA viruses

With fast accumulated RNA viruses from the environmental sequencing samples, determining reservoirs of novel viruses is becoming more important. In the second part, we evaluated the capability of VirHost to identify hosts of novel viruses using leave-one-genus-out strategy. Specifically, we train the model without including specific genera and then assess its performance on those genera to mimic the situation where a novel query, particularly with unknown genus label, is used as input. In this benchmark, we compared VirHost against the alignment-based method, BLASTN, and two null models (Null 1 and Null 2). Null 1 randomly assigns the host labels following the host label distribution of reference viruses in the training data, while Null 2 determines the query's host using the dominant host label in the training data. The result is shown in Fig. 4. As DeepHoF does not allow us to retrain their model, we cannot include DeepHoF in this experiment.

Some genera might have different host tropisms with their closely-related viruses under the same order or family, which poses a great challenge in predicting their reservoirs. Below we provide a comprehensive discussion of these challenging cases. We counted the dominant host labels in Layer 1 for each virus order, family, and genus. According to the difference between the host tropisms of the genus and its corresponding order or family, we consider three chal-

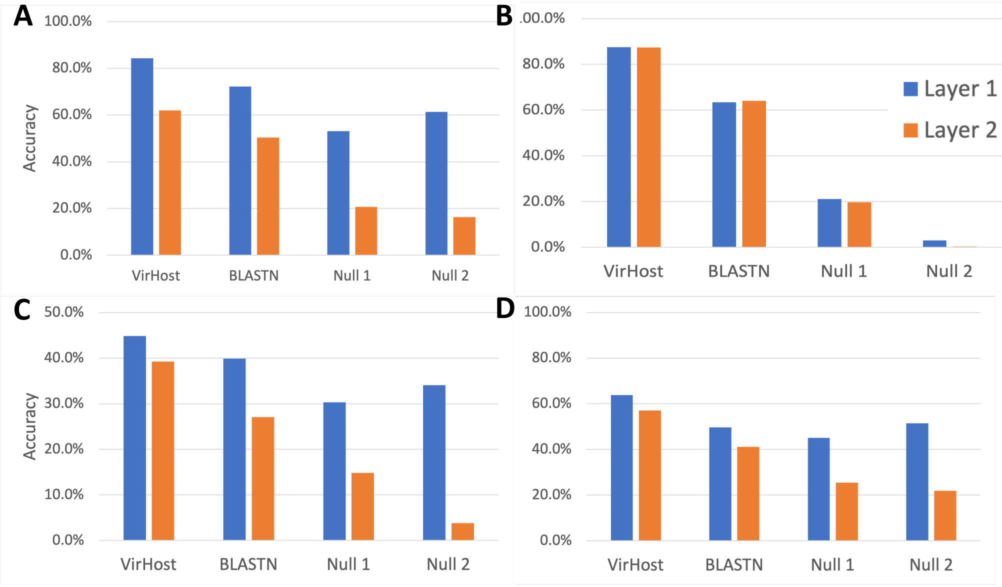

Figure 4. The performance comparison of different strategies in the leave-one-genus-out experiment. The figures demonstrate the average accuracy of A) all 448 genera, B) 138 genera of case 1, whose dominant host label is different from their order's, C) 50 genera of case 2, whose dominant host label is different from its family's, D) 56 genera of cases 3 with members infecting hosts of different kingdoms or phyla. Null 1 randomly assigns the host labels following the distribution of references. Null 2 determines the query's host by the dominant host label in references.

lenging cases. **Case 1)**: the genus's dominant host label is different from its order's. A typical case is Nepovirus in Picornavirales. While Nepovirus is a plant-infecting virus genus, seventy-four percent of members of the Picornavirales infect chordates. **Case 2)** the genus's dominant host label is different from its family's, such as Seadornavirus in Sedoreoviridae. While mosquitoes are the natural host of Seadornavirus, Spinareoviridae mainly infect chordates. Some genera in case 2 also belong to case 1, indicating that the host tropism of these genera are divergent from their homologous viruses within the same family and order. **Case 3)**: the members of the genus infecting hosts from divergent kingdoms or phyla. By discussing the challenging cases, we expect to exhibit the performance of VirHost in different situations more comprehensively.

In our dataset, there are 448 virus genera across 18 orders. VirHost achieved the highest genus-wise average accuracy of 84.3% and 61.9% for L1 and L2, respectively, outperforming BLASTN by 12.1% and 11.5%. There are 138 genera as case 1, whose dominant host labels varied from their order's, 50 genera as case 2, whose dominant host labels differ from their family's, and 56 genera infecting hosts from multiple phyla. VirHost achieved the best result in various cases. Specifically, in 138 genera of case 1, VirHost got accuracy of 87.5%(L1) and 87.4%(L2), outperforming BLASTN by 24.1% and 23.4%. In 50 genera of case 2, VirHost got accuracy of 44.8%(L1) and 39.2%(L2), surpassing BLASTN by 4.9% and 12.1%. In 56 genera with divergent host groups, VirHost achieved accuracy of 63.8%(L1) and 57.0%(L2), exceeding BLASTN by 14.1% and 15.9%. The improvement of VirHost likely result from the utilization of genomic traits and the superiority of the machine learning method.

Experiments on recently identified viruses

Finally, we retrained VirHost on all the reference viruses and assessed its accuracy on identifying hosts for recently identified viruses, whose hosts are derived based on experimental evidence. This process was designed to replicate scenarios that potential users of VirHost might typically encounter.

The four datasets associate with plants, invertebrate, fungi, and fishes (chordates), respectively, corresponding to the first layer's host labels in our tool. Each dataset comprised of viruses

Table 2. The statistics regarding taxonomic groups and host labels of the newly sequenced datasets.

	# viruses	# virus orders	Host group	# host classes	# host orders
Dataset 1	21	5	Plant	1	6
Dataset 2	15	3	Invertebrate	2	4
Dataset 3	69	9	Fungi	4	6
Dataset 4	21	6	Chordata - Fish	1	2

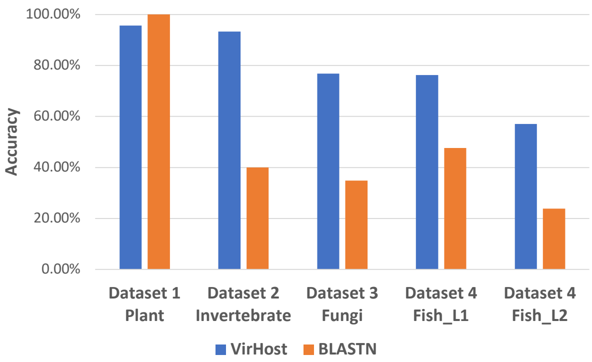

Figure 5. Host prediction accuracy of VirHost and BLASTN on recently identified virus datasets. In dataset 4, L1 and L2 denote Layer 1 and Layer 2, respectively.

sequenced from different hosts. The statistics is listed in Table 2. In all four datasets, VirHost achieved better or comparable performance than BLASTN, as shown in Fig. 5. The first dataset consists of 21 viruses, whose hosts are from 6 orders under Magnoliopsida plants [49, 50, 51, 52, 53, 54, 55]. VirHost predicted one case to be fungi-associated, which is a member of Alphapartitivirus, a genus that infecting both plant and fungi. This may suggest that the query has the ability to infect fungi. The second dataset includes 15 viruses, infecting hosts of 4 Arthropoda orders [56, 57, 58, 59]. VirHost achieved a high accuracy of 93.3% while BLASTN got 40%. The only misclassified query obtained balanced

prediction score of VirHost between Chordata and Invertebrate, which implied its potential to infect vertebrate. The third dataset involves 69 viruses from 9 orders and their hosts spanned 6 fungi orders [60, 61, 62, 63, 64, 65, 66]. VirHost achieved an accuracy of 76.8%, outperforming BLASTN by 28.6%. Finally, the fourth dataset comprises 21 viruses from 6 orders, infecting seahorses and salmon [67, 68]. We achieved accuracy of 76.2% and 57.1% in Layer 1 and Layer 2, respectively, outperforming BLASTN by 28.6% and 33.3%.

Discussion

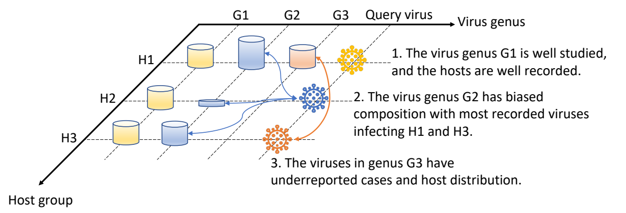

Figure 6. A visualization illustrating the potential sequencing bias in the reference database, posing a challenge to the prediction of reservoirs. On the virus genus axis, there are three reference genera represented by colors: yellow for G1, blue for G2, and red for G3. The host group axis consists of three distinct host groups (H1, H2, H3) present in the reference database. The cylinders at the intersections of the dashed lines represent viruses belonging to the respective genera that infect the corresponding host groups. The height of each cylinder indicates the relative number of viruses. G1 is extensively studied, and the hosts associated with it are well-documented. G2 and G3 are under studied, resulting in limited information about their hosts.

The application of metagenomic sequencing promotes the identification of novel viruses from host-associated and environmental samples of a diverse set of ecosystems. However, the high diversity of the potential hosts, the sampling method and location, and the heterogeneity of metagenomic sequencing make it hard to determine reservoirs of the detected viruses, which is a critical step for pandemics surveillance and One Health. In this study, we compared various features used in host prediction of RNA viruses and developed a tool, VirHost, that allows a fast and reliable reservoir prediction by only using the virus sequences. Compared to the laborious and expensive process of host verification in wet lab, VirHost offers a time and resource-effective strategy. By integrating genome traits and sequence homology of viruses, VirHost achieved higher accuracy than the alignment based method. Instead of focusing on vertebrate associated viruses, we extend the host range to plants, fungi and bacteria. With the increasing available viruses' host annotations, VirHost can be can be easily scaled to more viruses and hosts.

Despite the potential implications of our study, it is important to acknowledge the challenges that lie ahead for future research in this field. Two particular challenges warrant careful consideration: the unexpected host switch and the existence of multiple hosts. The long-term co-evolution between hosts and viruses may shape the genomic traits of viruses in a way that enables the distinction of their reservoirs. However, when viruses transfer to new hosts, their genome heritage may no longer reflect their host tropism, and the selection force may not immediately reveal their potential. To anticipate the phenomenon of host switching, additional information is required, including the host lifestyle, virus infection patterns, and virus-host protein-protein interactions. Obtaining such information can provide a more comprehensive understanding of the factors influencing host switching and improve our ability to forecast and respond to pandemics.

Turning our attention to the second challenge, we must address the inherent limitations of VirHost, which is designed to predict the viruses' reservoirs. In specific cases where viruses can infect multiple hosts, it requires more comprehensive analysis, like single nucleotide variants, to draw the host range accurately. On the other hand, due to the sequencing bias in the database, there exists a knowledge gap that some potential hosts of detected viruses have yet to record. We discussed how the sequencing bias towards different hosts can lead to misclassification, as shown in Fig. 6. Our focus was primarily on the genus-level analysis. To account for the sequencing bias, we categorized virus genera into two groups: well-recorded cases and under-reported cases. Well-recorded cases refer to virus genera that have been exhaustively sequenced in all of their hosts without any sequencing bias, represented by virus genus G1. The availability of a complete reference database greatly benefits the host prediction of new queries. On the contrary, the under-reported cases depict the virus genera whose diversity is under-studied among their hosts, such as the virus genera G2 and G3. In instances where the query virus infects a host that is under-reported in the reference database, the prediction relies heavily on the available hosts and thus may be biased towards hosts that have been extensively recorded. For example, if there is a virus from genus G3 infecting host group H3, but the host recorded in the reference database is H1, there is a high possibility that it will be misclassified as infecting H1. Therefore, the incomplete host range record can negatively impact the accuracy of host prediction. To mitigate this bias, it is crucial to continue expanding and updating the reference database by including data from under-reported hosts. Further understanding of these viruses will enhance the prediction and help to determine the boundary of infection.

Availability of source code and requirements

- Project name: VirHost
- Project home page: https://github.com/GreyGuoweiChen/VirHost
- Operating system(s): Platform independent
- Programming language: Python
- Other requirements: Python 3.8, BLAST 2.12.0+, Prodigal 2.6.3+, xgboost 2.0.3, pandas 2.0.3, biopython 1.83, numpy 1.23.5
- License: MIT license
- RRID: SCR_025061

Data availability

Additional Files

Supplementary Table S1. The feature contribution of genomic traits to host prediction was assessed using XGBoost models across different virus orders. The contribution is measured as the feature importance. The fold between the observed and expected importance is calculated as the ratio between the mean of importance and the reciprocal of the total number of genomic traits (4041). A higher fold value indicates a greater importance demonstrated by the feature.

Supplementary Fig. S1. The accuracy of models using genomic traits in the Layer 1 when considering different number of codon pair bias.

Supplementary Fig. S2. The accuracy distribution of models using different feature set across virus orders. To demonstrate the effectiveness of our method, we evaluated the median accuracy of different models using Wilcoxon test. Specifically, we conducted one-sided Wilcoxon test to compare our method with BLASTN (P1) and Bias_Blast (P2). A small p-value ($P < 0.05$) suggests strong evidence to support the alternative hypothesis that the median accuracy of our method is significantly larger than that of the compared methods.

Supplementary Fig. S3. The Precision-Prediction_rate curve of

VirHost and BLASTN in 9 virus orders that contain more than 500 viruses. A curve closer to the upper-right corner indicates better model performance. The blue dot represents VirHost's performance when applying the empirical prediction score cutoff. The red dot represents the performance of BLASTN's final prediction using the best alignment strategy. The prediction rate is lower than 100% due to the inability to align some query sequences with the reference sequences. Diff denotes the precision difference between VirHost and BLASTN at the same prediction rate.

Supplementary Fig. S4. The average accuracy of models using different learning architectures with the same features as VirHost.

Supplementary Information Section 1: Details of data collection. Section 2: The description of benchmark features. Section 3: Feature selection. Section 4: The trade-off between prediction rate and precision.

Competing Interests

The authors declare that they have no competing interests.

Funding

The study is supported by Hong Kong Research Grants Council (RGC) General Research Fund (GRF) [11206819, 11217521] and Hainan Provincial Natural Science Foundation of China [324CXTD435].

Author's Contributions

GC contributed to the data collection and analysis, experiment execution, framework design, and paper writing. YS conceptualized the study. JJ and YS oversaw the project. All authors reviewed, contributed to, and approved the manuscript.

References

1. Callanan J, Stockdale SR, Shkoporov A, Draper LA, Ross RP, Hill C. Expansion of known ssRNA phage genomes: from tens to over a thousand. Science advances 2020;6(6):eaay5981.
2. Mushegian A. Are there 10^{31} virus particles on earth, or more, or fewer? Journal of bacteriology 2020;202(9):10–1128.
3. Wolf YI, Silas S, Wang Y, Wu S, Bocek M, Kazlauskas D, et al. Doubling of the known set of RNA viruses by metagenomic analysis of an aquatic virome. Nature microbiology 2020;5(10):1262–1270.
4. Neri U, Wolf YI, Roux S, Camargo AP, Lee B, Kazlauskas D, et al. Expansion of the global RNA virome reveals diverse clades of bacteriophages. Cell 2022;185(21):4023–4037.
5. Zayed AA, Wainaina JM, Dominguez-Huerta G, Pelletier E, Guo J, Mohssen M, et al. Cryptic and abundant marine viruses at the evolutionary origins of Earth's RNA virome. Science 2022;376(6589):156–162.
6. Santiago-Rodriguez TM, Hollister EB. Unraveling the viral dark matter through viral metagenomics. Frontiers in Immunology 2022;13:1005107.
7. Haydon DT, Cleaveland S, Taylor LH, Laurenson MK. Identifying reservoirs of infection: a conceptual and practical challenge. Emerging infectious diseases 2002;8(12):1468–1473.
8. Swinton, Harwood, Grenfell, Gilligan. Persistence thresholds for phocine distemper virus infection in harbour seal Phoca vitulina metapopulations. Journal of Animal Ecology 1998;67(1):54–68.
9. Shan T, Yang S, Wang H, Wang H, Zhang J, Gong G, et al. Virome in the cloaca of wild and breeding birds revealed a diversity of significant viruses. Microbiome 2022;10(1):1–21.
10. Blanc S, Michalakis Y. Manipulation of hosts and vectors by plant viruses and impact of the environment. Current opinion in insect science 2016;16:36–43.
11. Pons JC, Paez-Espino D, Riera G, Ivanova N, Kyrpides NC, Llabrés M. VPF-Class: taxonomic assignment and host prediction of uncultivated viruses based on viral protein families. Bioinformatics 2021;37(13):1805–1813.
12. Coutinho FH, Zaragoza-Solas A, López-Pérez M, Barylski J, Zielezinski A, Dutilh BE, et al. RaFAH: Host prediction for viruses of Bacteria and Archaea based on protein content. Patterns 2021;2(7).
13. Ruohan W, Xianglilan Z, Jianping W, Shuai Cheng L. DeepHost: phage host prediction with convolutional neural network. Briefings in Bioinformatics 2022;23(1):bbab385.
14. Shang J, Sun Y. CHERRY: a Computational metHod for accuratE pRediction of virus–pRokarYotic interactions using a graph encoder–decoder model. Briefings in Bioinformatics 2022;23(5):bbac182.
15. Wolf YI, Kazlauskas D, Iranzo J, Lucía-Sanz A, Kuhn JH, Krupovic M, et al. Origins and evolution of the global RNA virome. MBio 2018;9(6):10–1128.
16. Campillo-Balderas JA, Lazcano A, Becerra A. Viral genome size distribution does not correlate with the antiquity of the host lineages. Frontiers in Ecology and Evolution 2015;3:143.
17. Silas S, Makarova KS, Shmakov S, Páez-Espino D, Mohr G, Liu Y, et al. On the origin of reverse transcriptase-using CRISPR-Cas systems and their hyperdiverse, enigmatic spacer repertoires. MBio 2017;8(4):10–1128.
18. Raj A, Dewar M, Palacios G, Rabadan R, Wiggins CH. Identifying hosts of families of viruses: a machine learning approach. PLoS One 2011;6(12):e27631.
19. Eng CL, Tong JC, Tan TW. Predicting host tropism of influenza A virus proteins using random forest. BMC medical genomics 2014;7:1–11.
20. Mock F, Viehweger A, Barth E, Marz M. VIDHOP, viral host prediction with deep learning. Bioinformatics 2021;37(3):318–325.
21. Zhang Z, Cai Z, Tan Z, Lu C, Jiang T, Zhang G, et al. Rapid identification of human-infecting viruses. Transboundary and emerging diseases 2019;66(6):2517–2522.
22. Bartoszewicz JM, Seidel A, Renard BY. Interpretable detection of novel human viruses from genome sequencing data. NAR genomics and bioinformatics 2021;3(1):lqab004.
23. Pandit PS, Anthony SJ, Goldstein T, Olival KJ, Doyle MM, Gardner NR, et al. Predicting the potential for zoonotic transmission and host associations for novel viruses. Communications biology 2022;5(1):844.
24. Zhang Z, Lu C, Mo B, Bai K, Ge XY, Deng L, et al. Prediction of mammalian virus cross-species transmission based on host proteins. Microbiology Spectrum 2023;11(5):e05368–22.
25. Babayan SA, Orton RJ, Streicker DG. Predicting reservoir hosts and arthropod vectors from evolutionary signatures in RNA virus genomes. Science 2018;362(6414):577–580.
26. Lee B, Smith DK, Guan Y. Alignment free sequence comparison methods and reservoir host prediction. Bioinformatics 2021;37(19):3337–3342.
27. Young F, Rogers S, Robertson DL. Predicting host taxonomic information from viral genomes: A comparison of feature representations. PLoS computational biology 2020;16(5):e1007894.
28. Guo Q, Li M, Wang C, Guo J, Jiang X, Tan J, et al. Predicting hosts based on early SARS-CoV-2 samples and analyzing the 2020 pandemic. Scientific Reports 2021;11(1):17422.
29. Hulo C, De Castro E, Masson P, Bougueleret L, Bairoch A, Xenarios I, et al. ViralZone: a knowledge resource to understand virus diversity. Nucleic acids research 2011;39(suppl_1):D576–D582.
30. Mihara T, Nishimura Y, Shimizu Y, Nishiyama H, Yoshikawa G, Uehara H, et al. Linking virus genomes with host taxonomy. Viruses 2016;8(3):66.

31. Fu L, Niu B, Zhu Z, Wu S, Li W. CD-HIT: accelerated for clustering the next-generation sequencing data. Bioinformatics 2012;28(23):3150–3152.
32. Schoch CL, Ciufo S, Domrachev M, Hotton CL, Kannan S, Khovanskaya R, et al. NCBI Taxonomy: a comprehensive update on curation, resources and tools. Database 2020;2020:baaa062.
33. Shi M, Lin XD, Chen X, Tian JH, Chen LJ, Li K, et al. The evolutionary history of vertebrate RNA viruses. Nature 2018;556(7700):197–202.
34. Taubenberger JK, Kash JC. Influenza virus evolution, host adaptation, and pandemic formation. Cell host & microbe 2010;7(6):440–451.
35. Jitobaom K, Phakaratsakul S, Sirihongthong T, Chotewutmontri S, Suriyaphol P, Suptawiwat O, et al. Codon usage similarity between viral and some host genes suggests a codon-specific translational regulation. Heliyon 2020;6(5).
36. Kustin T, Stern A. Biased mutation and selection in RNA viruses. Molecular Biology and Evolution 2021;38(2):575–588.
37. Lobo FP, Mota BE, Pena SD, Azevedo V, Macedo AM, Tauch A, et al. Virus-host coevolution: common patterns of nucleotide motif usage in Flaviviridae and their hosts. PloS one 2009;4(7):e6282.
38. Coleman JR, Papamichail D, Skiena S, Futcher B, Wimmer E, Mueller S. Virus attenuation by genome-scale changes in codon pair bias. Science 2008;320(5884):1784–1787.
39. Hyatt D, LoCascio PF, Hauser LJ, Uberbacher EC. Gene and translation initiation site prediction in metagenomic sequences. Bioinformatics 2012;28(17):2223–2230.
40. Wood DE, Lu J, Langmead B. Improved metagenomic analysis with Kraken 2. Genome biology 2019;20:1–13.
41. Chang CH, Nelson WC, Jerger A, Wright AT, Egbert RG, McDermott JE. Snekmer: a scalable pipeline for protein sequence fingerprinting based on amino acid recoding. Bioinformatics Advances 2023;3(1):vbad005.
42. Pedregosa F, Varoquaux G, Gramfort A, Michel V, Thirion B, Grisel O, et al. Scikit-learn: Machine Learning in Python. Journal of Machine Learning Research 2011;12:2825–2830.
43. Hughes HR, Adkins S, Alkhovskiy S, Beer M, Blair C, Calisher CH, et al. ICTV virus taxonomy profile: Peribunyaviridae. Journal of General Virology 2020;101(1):1–2.
44. Sasaya T, Palacios G, Briese T, Di Serio F, Groschup MH, Neriya Y, et al. ICTV Virus Taxonomy Profile: Phenuiviridae 2023. Journal of General Virology 2023;104(9):001893.
45. Garrison AR, Alkhovsky SV, Avšič-Županc T, Bente DA, Bergeron É, Burt F, et al. ICTV virus taxonomy profile: Nairoviridae. Journal of General Virology 2020;101(8):798–799.
46. Xie J, Jiang D. New insights into mycoviruses and exploration for the biological control of crop fungal diseases. Annual Review of Phytopathology 2014;52:45–68.
47. Casteel CL, Falk BW. Plant virus-vector interactions: More than just for virus transmission. Current research topics in plant virology 2016;p. 217–240.
48. Eppler A, Heinze C, Adam G. Invertebrates and Fungi in Plant Virus Diseases. e LS 2001;.
49. Cruz J, Freire A, Polimeni J, Blawid R. A novel deltacryptic virus identified in Allium cepa from Brazil. Acta virologica 2023 01;67(1):109–113.
50. Maachi A, Hernando Y, Aranda MA, Donaire L. Cohombrillo-associated virus: a novel virus infecting Ecballium elaterium plants. Archives of Virology 2023;168(1):16.
51. Gudeta WF, Shin AY, Kim SE, Jeong-A K, Seok-Yoon K, Moon JS. Complete genome sequence of Stellaria aquatica virus B, a novel polerovirus that infects Stellaria aquatica. Archives of Virology 2023;168(1):22.
52. Park YC, Kim KK, Jun HJ, Kang EH, Lee SR, Ahn JK, et al. Complete genome sequence of a novel member of the genus Polerovirus from Cnidium officinale in South Korea. Archives of Virology 2023;168(4):104.
53. Wang Z, Anane RF, Chen Z, Gao L, Li S, Chu B, et al. Complete genome sequence analysis of Paris alphapartitivirus 1: a novel member of the genus Alphapartitivirus infecting Paris polyphylla var. y unnanensis. Archives of Virology 2022;167(11):2365–2370.
54. Guo J, Yin J, Hu H, Zhang T, Ye Z, Yang J, et al. Molecular characterization of a novel benyvirus infecting wheat in China. Archives of Virology 2023;168(12):284.
55. Nie Z, Zhang X, Li Y, Zhang Z, Han C, Wang Y. Molecular characterization of a novel cytorhabdovirus infecting Plumbago indica L. Archives of Virology 2023;168(12):289.
56. Ryabov EV, Nearman AJ, Nessa A, Grubbs K, Sallmann B, Fahey R, et al. Apis mellifera Solinvivirus-1, a Novel Honey Bee Virus That Remained Undetected for over a Decade, Is Widespread in the USA. Viruses 2023;15(7):1597.
57. Xu A, Xu S, Tu Q, Qiao H, Lin W, Li J, et al. A novel virus in the family Marnaviridae as a potential pathogen of Penaeus vannamei glass post-larvae disease. Virus Research 2023;324:199026.
58. Martinez-Mercado MA, de Jesús JLD, Galindo-Sánchez CE, Saavedra-Flores A, Carrillo-Tripp J. Novel viral RNA genomes of the vine mealybug Planococcus ficus. Journal of General Virology 2022;103(3):001717.
59. da Silva LA, Basso MF, Ribeiro BM. A novel picorna-like virus identified in the cotton boll weevil Anthonomus grandis (Coleoptera: Curculionidae). Archives of Virology 2023;168(1):29.
60. Liu H, Zhang Y, Liu Y, Xiao J, Huang Z, Li Y, et al. Virome analysis of an ectomycorrhizal fungus Suillus luteus revealing potential evolutionary implications. Frontiers in Cellular and Infection Microbiology 2023;13.
61. Pagnoni S, Oufensou S, Balmas V, Bulgari D, Gobbi E, Forgia M, et al. A collection of Trichoderma isolates from natural environments in Sardinia reveals a complex virome that includes negative-sense fungal viruses with unprecedented genome organizations. Virus Evolution 2023;9(2):vead042.
62. Zhao Y, Zhang Y, Wan X, She Y, Li M, Xi H, et al. A novel ourmia-like mycovirus confers hypovirulence-associated traits on Fusarium oxysporum. Frontiers in Microbiology 2020;11:569869.
63. Ye T, Lu Z, Li H, Duan J, Hai D, Lin Y, et al. Characterization of a fungal virus representing a novel genus in the family Alphaflexiviridae. Viruses 2023;15(2):339.
64. Zhao Yj, Shirouzu T, Chiba Y, Hosaka K, Moriyama H, Urayama Si, et al. Identification of novel RNA mycoviruses from wild mushroom isolates in Japan. Virus Research 2023;325:199045.
65. Fu Y, Wang T, Zhou S, Zhou J, Zhao Y, Chen D, et al. A novel narnavirus isolated from Colletotrichum curcumae strain 780-2T. Archives of Virology 2023 08;168.
66. Wang H, Luo J, Dai R, Shah KU, Andika IB, Sun L. Complete genome sequence of a novel double-stranded RNA virus infecting the phytopathogenic fungus Rhizopus stolonifer. Archives of Virology 2023;168(9):239.
67. Mordecai GJ, Miller KM, Di Cicco E, Schulze AD, Kaukinen KH, Ming TJ, et al. Endangered wild salmon infected by newly discovered viruses. Elife 2019;8:e47615.
68. Zhang F, Ren Z, Guo X, Wang Y, Meng F, Shi W, et al. Meta-Transcriptomic Analysis Reveals Novel RNA Viruses in Hippocampus erectus. Viruses 2023;15(3):772.

Manuscript Click here to access/download;Manuscript;main.pdf

Placeholder for OUP logo oup.pdf

Placeholder for journal logo gigascience-logo.pdf

GigaScience, 2023, 1–??

doi: xx.xxxx/xxxx
Manuscript in Preparation
Research

RESEARCH

VirHost: a machine learning-based method for predicting reservoir hosts of RNA viruses through viral genomes

Guowei Chen[1], Jingzhe Jiang[2] and Yanni Sun[1,*]

[1]Department of Electrical Engineering, City University of Hong Kong, Hong Kong (SAR), China and [2]Key Laboratory of South China Sea Fishery Resources Exploitation & Utilization, Ministry of Agriculture and Rural Affairs, South China Sea Fisheries Research Institute, Chinese Academy of Fishery Sciences, Guangzhou 510300, China

[*]Correspondence address. Department of Electrical Engineering, City University of Hong Kong, 83 Tat Chee Avenue, Kowloon, Hong Kong (SAR), China. E-mail: yannisun@cityu.edu.hk

Abstract

Background: The advent of high-throughput sequencing technologies has revolutionized the identification of novel RNA viruses in host-associated or environmental samples. Given that viruses are infectious agents, identifying the reservoir hosts of these new viruses carries significant implications for public health and provides valuable insights into the dynamics of the microbiome. However, determining the hosts of these newly discovered viruses is not always straightforward, especially in the case of viruses detected in environmental samples. Even for host-associated samples, it is not always correct to assign the sample origin as the host of the identified viruses. The process of assigning reservoirs to RNA viruses remains challenging due to their high mutation rates and vast diversity.
Results: In this study, we introduce VirHost, a machine learning-based tool that predicts the reservoirs of RNA viruses solely based on viral genomes. VirHost is a hierarchical classification framework that predicts reservoirs at different taxonomic levels. We demonstrate the superior accuracy of VirHost in predicting reservoirs of RNA viruses through comprehensive comparisons with various state-of-the-art techniques. When applying to viruses from novel genera, VirHost achieved the highest accuracy of 84.3%, outperforming the alignment-based strategy by 12.1%.
Conclusions: The application of machine learning models has proven beneficial in predicting reservoirs of RNA viruses. By integrating genomic traits and sequence homologies, VirHost provides a cost-effective and efficient strategy for reservoir prediction. We believe that VirHost can greatly assist in RNA virus analyses and contribute to pandemic surveillance.

Key words: RNA virus; host prediction; reservoir; machine learning; metagenomics

Introduction

Viruses are obligate intracellular parasites that depend on living organisms for their replication and survival. RNA viruses, possessing RNA as their genetic material, have the capability to infect a diverse array of organisms. For example, several types of RNA viruses are causal agents of the most disastrous pandemics in human history, including COVID-19, SARS, the annual influenza, etc. Furthermore, certain plant and animal RNA viruses pose a threat to agricultural and animal sectors, jeopardizing crop growth, livestock health, and subsequently leading to substantial economic losses in agriculture and animal husbandry. Besides eukaryotic hosts, some RNA viruses can also infect bacteria and thus directly affect the dynamics of microbiome [?].

Understanding the interaction between viruses and their hosts is a fundamental step in characterizing the viruses' roles in public health, animal husbandry, agriculture, etc. While known RNA viruses often have annotated hosts, many newly discovered viruses

Compiled on: March 7, 2024.
Draft manuscript prepared by the author.

lack the host information. Viruses are believed to be the most diverse and abundant biological entities in the world [?]. Currently, metagenomic sequencing has emerged as the primary approach for the discovery of novel viruses, as it eliminates the need for virus isolation and cultivation in laboratory settings. This method involves directly sequencing genetic material from host associated or environmental samples, allowing for the identification of viruses present within these complex ecosystems. A series of studies has been conducted to identify novel viruses in various ecosystems [? ? ?].

While the application of metagenomic high-throughput sequencing technologies have facilitated the discovery of the viral dark matter [?], how to determine the reservoir hosts of the metagenome-originated viruses remains challenging owing to the complex composition of the metagenomic sequencing samples. Regardless of the various definitions of reservoir hosts [? ?], in this context, we highlight the natural hosts that allow the persistence of the viruses, instead of the circulation or transmission. In some cases, viruses may be detected in non-host organisms due to the symbiotic relationship, the dietary interaction, or the physical contact, like the bacteria-infecting viruses and the plant-associated viruses found in bird digestive tract [?] and the plant-infecting viruses found in insect vectors [?]. These carriers are not the primary focus of our study.

Traditionally, the host verification requires stringent experimental contribution, including the isolation of viral particles from hosts of interests, the serological tests, the epidemiological investigation, and virus phylogenetic analyses. These processes are time-consuming, labor-intensive, and often require specialized equipment and expertise. While the metagenomic sequencing is becoming the main source of the novel viruses, the heterogeneous composition made it harder to determine the target hosts. Therefore, when the novel viruses rapidly emerge, predicting the hosts from the virus genome sequences, avoiding the tedious laboratory steps, show its attractive advantage in terms of economy and efficiency.

By far, a number of computational works have been conducted to explore the association between viruses and the potential hosts. While RNA viruses dominate the eukaryotic viruses, the majority of prokaryotic viruses are DNA viruses. Due to the extensive host range of RNA viruses and the limited availability of reference genomes for potential eukaryotic hosts, the development of computational tools for predicting hosts of prokaryotic viruses is faster than the development of host prediction tools for eukaryotic viruses. To predict the hosts of phages (viruses infecting prokaryotes), VPF-Class classified a set of Viral Protein Families (VPFs) and aligned the queries virus to the categorized references [?]. RaFAH generated protein clusters, constructed profile Hidden Markov Models (pHMMs), and trained a random forest classification model using the pHMMs alignment score [?]. DeepHost encoded the spaced k-mer feature by a three-dimensional matrix and trained a convolutional neural network (CNN) to predict the hosts [?]. CHERRY integrated various signals, including gene organization, CRISPR, sequence similarity, and k-mer usage, and predicted the virus-host association by a graph convolutional encoder and decoder [?]. Currently, these tools allow host prediction at different ranks and the accuracy decreases with more refined host range (e.g. from class to species). Nevertheless, these tools are limited to host prediction for prokaryotic viruses.

Compared to the extensive studies on phages, the host prediction of RNA viruses remains challenging and arduous. The typical genomes of RNA viruses, ranging from 3kbp to 41kbp [?], is smaller than that of DNA viruses (5kbp to 600kbp) and has limited capacity to carry host tropism signals [?]. While the sequence matches between phages and the prokaryotic genomes facilitate the host prediction of phages, they are less common in RNA viruses [? ?]. Furthermore, while many bacterial genomes have been sequenced with metagenomic sequencing, the extensive host range of RNA viruses, limited availability of the potential hosts, and the very large sizes of the potential host genomes make adding host genome features very difficult. Finally, the high mutation rate of RNA viruses makes the genomes less conserved, so that the existing achievement can not be extend to the novel viruses.

With these challenges, the computational frameworks still show outstanding performance in two host prediction scenarios. The first is to predict the host for a specific group of RNA viruses. ?] counted the spaced amino acid k-mer frequency and trained an Alternating Decision Tree classifier for two families, Picornaviridae and Rhabdoviridae. ?] encoded the protein sequences by the physical and chemical property of the amino acid and trained a random forest for the influenza A virus. ?] developed two deep neural network models for the host classification of three viruses, respectively (influenza A virus, rabies lyssavirus, and rotavirus A). These viruses associated closely with human's activity and thus attracted attention.

In another scenario, the researchers discuss whether the query viruses will infect the targeted host group, particularly human and mammals. ?] leveraged the k-mer frequency and designed a k-nearest neighbor model to discriminate the human-infecting viruses from other viruses. ?] applied the reverse-complement neural networks to do read-based prediction of the viral host (human or non-human). ?] investigated the host sharing network of mammalian viruses and trained gradient boosting models to predict the host sharing situation of two viruses. ?] generated a set of protein families that commonly shared by mammals, studied the correlation between these proteins and the cross-species transmission, and trained a random forest model to predict the transmission of viruses.

However, these two categories of works are hard to extend to the host prediction of metagenome-assembled RNA viruses. They overlooked the broader host range of RNA viruses, including additional host candidates, like plants, invertebrates, and fungi. To predict hosts of the increasing novel RNA viruses, some primary explorations have been made. ?] investigated the genomic traits and the sequences homologs of viruses, and developed a classification model considering viruses from 12 taxonomic groups and 11 reservoir groups. Building upon Babayan's study, ?] further evaluated the application of Machine learning with digital signal processing-based Structural Patterns (M-SP) of viruses in reservoirs prediction. ?] assessed the gene content and the frequency of short sequences, and developed a hierarchical host classification framework based on support vector machine. ?] trained a two-branch convolutional neural network to capture the informative motifs and classified the viruses into five host groups. Despite the promising results obtained from these validations, these studies still face the challenge of limited viruses and host ranges.

In this work, we concentrate on predicting reservoirs of emergent novel viruses and thus developed a hierarchical host classification framework, VirHost. Combining virus taxonomy, genomic traits, and sequence homologies, VirHost allows predicting the reservoirs using only viral genomes. To cover as many viruses and reservoirs as we can, VirHost accepts queries from over 30 virus orders, and includes five host types in its first layer, including Chordata (Vertebrate), Invertebrate, Plant, Fungi, Bacteria. After obtaining the prediction results in the first layer, VirHost will perform additional predictions in the second layer, to obtain more precise host classification information. By evaluating various features and learning architectures in a more comprehensive database, we demonstrated the outstanding performance of VirHost in reservoir prediction of RNA viruses. We also evaluated VirHost's performance on novel viruses by conducting leave-one-taxon-out experiments. The results of these experiments demonstrated that VirHost can be effectively applied across the diverse landscape of viruses, without being limited to specific viral types.

Method

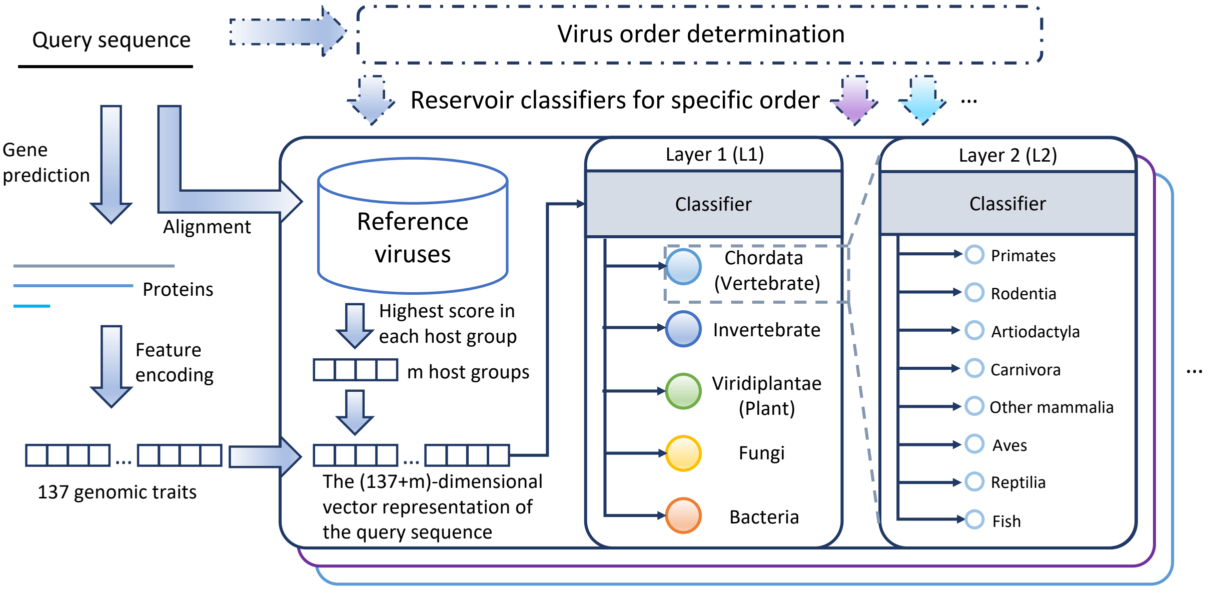

Figure 1. The framework of VirHost. The hierarchical host prediction consists of two classification layers, Layer 1 (L1) and Layer 2 (L2). In Layer 1, we predict hosts at kingdom and phylum level, including Chordata, Invertebrate, Viridiplantae, Fungi, Bacteria. In Layer 2, we further predict the specific host groups under Chordata at class and order level. The genomic traits consist of the usage preference of nucleotide, dinucleotide, codon, and amino acid. We categorized the reference viruses based on their hosts, and m denotes the number of host groups (output labels) in the corresponding classifiers.

Overview of the method

The framework of VirHost is depicted in Fig. ??. Initially, we categorize the queries based on their taxonomic information to narrow down the potential host range. Then, we extract two types of features, genomic traits and sequence homology, for reservoir prediction using a learning-based model. This two-step approach incorporates both the taxonomic information and the potential host signal in the sequences and is anticipated to enhance the accuracy of our predictions with greater confidence.

The origin and evolution of RNA viruses remain complicated and puzzling [?]. Different groups of viruses have distinct infection mechanisms and divergent host ranges. To benefit the reservoir prediction, we incorporated prior virus taxonomic knowledge in VirHost to mitigate potential interference among viruses originating from different sources. While the lower taxonomic rank may provide a precise host range, the emergent novel sequences may not fit neatly into the existing taxonomic label. Given the trade-off between accommodating more candidates and requiring informative taxonomic knowledge, we categorized viruses into 30 orders following ViralZone [?]. The measure is also expected to improve the learning performance by avoiding imbalance sizes of different virus orders within the same host groups. Then, we built independent classifiers for every order by leveraging the virus members' genomic traits and sequence homologies.

VirHost predicts the reservoirs of queries hierarchically. The models are of two-layer tree structures, with the first layer corresponding to the host's kingdom and phylum level, and the second layer to the host's class and order level (Section. Labels screening). Once input to corresponding models, the query will be encoded as its genomic traits and sequence homologies (Section. Feature Encoding). VirHost will output the reservoir labels along the tree. Based on comprehensive benchmarks, eXtreme Gradient Boosting (XGBoost), a scalable machine learning technique, delivered the top performance, making it the preferred choice as the default architecture for VirHost. When more data are available, VirHost can be easily scaled to include the new members.

Data preprocessing

Data collection

We collected 6,735 viruses from Virus-Host Database [?] and 126,417 records with host annotations from NCBI GenBank. To remove redundancy, we used CD-HIT [?] to de-replicate the identical sequences. Then we check the host annotations using NCBI Taxonomy database [?] and manual validation. Host tags are renamed to the corresponding scientific names, while the ambiguous host annotations were further removed. The details can be found in the Supplementary Information (section 1).

Label screening

Although numerous RNA viruses has been found, the current database is biased towards human and mammalian-associated viruses. To better predict the host lineage, we carefully curated the host labels based on both the host phylogenetic tree and the data availability. For each virus order, we built a two-layer host phylogenetic tree. Layer 1 contains 5 branches (Chordata, Invertebrate, Viridiplantae, Fungi, Bacteria), which are categorized into kingdom and phylum level. Layer 2, designed for Chordata subtree, has 10 leaves, which are at the class and order level. Hence, we hierarchically predict the host lineage along the tree. This hierarchical partition acknowledges the practical consideration, that host switch phenomena are more frequently observed at the hosts' class and order level [? ?], and offers users the flexibility to use both layers or one layer of host prediction of VirHost.

Some host labels have only a few recorded infecting viruses and thus are not ready for computational host prediction. For virus orders containing more then 30 viruses, we set the threshold as 10 and only keep host labels with at least 10 infecting viruses. After removing those "rare" host labels, the dataset contains 14,500 viruses and spans 30 virus orders. Their host distribution is shown in Table ??.

Feature Encoding

Previous studies have extensively explored various features of RNA viruses with different hosts. Both the genomic traits and viruses' sequence homologies facilitated the host prediction of RNA viruses.

Table 1. The virus order and host distribution after the label screening.

		Layer 1					Layer 2									
order	num	Chordata	Invertebrate	Viridiplantae	Fungi	Bacteria	Primates	Rodentia	Carnivora	Artiodactyla	Chiroptera	Other Mammalia	Aves	Reptilia	Amphibia	Fish
Ortervirales	2764	92.0%	-	8.0%	-	-	85.9%	0.9%	0.9%	2.0%	-	0.7%	1.6%	-	-	-
Picornaviral	2647	74.2%	12.2%	13.6%	-	-	40.2%	4.1%	4.2%	14.5%	2.6%	1.7%	3.9%	0.9%	-	2.0%
Bunyavirale	1524	52.4%	31.6%	15.2%	0.9%	-	18.0%	17.6%	-	1.6%	1.4%	5.3%	1.7%	5.6%	-	1.2%
Tymovirales	1042	-	2.6%	94.3%	3.1%	-	-	-	-	-	-	-	-	-	-	-
Reovirales	1034	52.9%	33.8%	9.7%	3.6%	-	9.9%	1.3%	2.9%	9.0%	5.5%	3.6%	9.6%	1.3%	-	10.0%
Amarillovira	817	85.2%	14.8%	-	-	-	54.0%	9.7%	-	10.0%	4.0%	4.2%	1.7%	-	-	1.6%
Mononegav	758	57.5%	27.8%	11.3%	3.3%	-	11.3%	6.5%	4.0%	5.4%	11.5%	2.4%	9.0%	1.6%	-	5.9%
Martelliviral	670	5.1%	6.3%	73.7%	14.9%	-	5.1%						-	-	-	-
Nidovirales	622	94.4%	5.6%	-	-	-	4.8%	5.3%	5.6%	36.3%	17.4%	5.6%	13.5%	5.8%	-	-
Patatavirale	558	-	-	100.0%	-	-	-	-	-	-	-	-	-	-	-	-
Ghabrivirale	393	-	14.0%	9.9%	76.1%	-	-	-	-	-	-	-	-	-	-	-
Durnavirale	340	5.0%	-	32.4%	62.6%	-	5.0%						-	-	-	-
Stellavirales	296	100.0%	-	-	-	-	10.1%	12.8%	8.4%	33.1%	5.1%	-	16.2%	-	4.4%	9.8%
Tolivirales	226	-	15.0%	73.9%	11.1%	-	-	-	-	-	-	-	-	-	-	-
Hepelivirale	181	80.1%	10.5%	9.4%	-	-	32.0%	16.6%	-	16.6%	-	8.8%	6.1%	-	-	-
Sobelivirale	120	-	12.5%	87.5%	-	-	-	-	-	-	-	-	-	-	-	-
Blubervirale	108	100.0%	-	-	-	-	75.9%	-	-	-	14.8%	-	9.3%	-	-	-
Cryppaviral	80	-	-	-	100.0%	-	-	-	-	-	-	-	-	-	-	-
Articulaviral	77	100.0%	-	-	-	-	59.7%						16.9%	-	-	23.4%
Jingchuvira	61	-	100.0%	-	-	-	-	-	-	-	-	-	-	-	-	-
Nodamuvir	42	-	100.0%	-	-	-	-	-	-	-	-	-	-	-	-	-
Ourlivirales	38	-	-	26.3%	73.7%	-	-	-	-	-	-	-	-	-	-	-
Wolframvir	23	-	-	-	100.0%	-	-	-	-	-	-	-	-	-	-	-
Mindivirales	22	-	-	-	-	100.0%	-	-	-	-	-	-	-	-	-	-
Norzivirales	21	-	-	-	-	100.0%	-	-	-	-	-	-	-	-	-	-
Serpentovir	16	-	-	100.0%	-	-	-	-	-	-	-	-	-	-	-	-
Muvirales	9	-	100.0%	-	-	-	-	-	-	-	-	-	-	-	-	-
Yadokarivir	7	-	-	-	100.0%	-	-	-	-	-	-	-	-	-	-	-
Goujianviral	3	-	100.0%	-	-	-	-	-	-	-	-	-	-	-	-	-
Timlovirales	1	-	-	-	-	100.0%	-	-	-	-	-	-	-	-	-	-
sum	14500															

Each row represents the host distribution of a virus order. While the column "num" shows the total number of viruses in the order, the following columns represent the percent of viruses infecting the corresponding hosts. Layer 2 consists of the Chordata subgroups from Layer 1, therefore the sum of values in the second layer is equal to the value of "Chordata". In cases (Martellivirales, Durnavirales, Articulavirales) where mammalian viruses are less than 50, we merge mammalian members into a single node, Mammalia.

VirHost relies on a subset of the genomic traits and the sequence homology. A widely accepted hypothesis is that the biases in genomic composition, also named as genomic trait, may hinted the natural selection pressure imposed by their hosts. To escape host immune responses and hijack the cellular machinery, viruses tend to mimic the genomic trait usage of their hosts [? ?]. It is reported that Flaviridae viruses associate with two host groups, vertebrate and invertebrate. The members infecting a single group have similar dinucleotide and codon preference as their hosts do [?]. Besides, the changes of codon pair bias were proven to influence the viruses' pathogenicity [?], showing that the host tropism potentially relates to the the genomic trait. To represents the genomic feature, we translate the query sequences to proteins using MetaProdigal [?] and generate a 137-dimensional vector $\mathbf{S} \in \mathbb{R}^{137}$, where S_i quantifies the preference of 137 genomic traits, including the usage preference of nucleotide (Eq. ??), dinucleotide (Eq. ??), codon (Eq. ??), and amino acid (Eq. ??).

On the other hand, related viruses tend to infect hosts that share taxonomic associations or have overlapping activity patterns. Thus, the viruses' sequence homology may indicate their host range. The sequence homology is introduced by conducting sequence alignment. The reference sequences are categorized into different groups by their hosts, and we used BLASTN to get the maximum alignment scores of the query against every virus group. The maximum alignment scores against all groups are converted into a m-dimensional vector, $\mathbf{H} \in \mathbb{R}^m$, where m is the number of virus groups (host labels) in the corresponding classifier. Finally, the two vectors, **S** and **H**, are concatenated into a (137+m)-dimensional vector, $\mathbf{X} \in \mathbb{R}^{137+m}$, and used as the representation of the query. The combination of genomic traits and viral sequence homology is expected to facilitate predicting the reservoir hosts. Here, we briefly describe the different features and more details are depicted in the Supplementary Information section 2.

Features from sequence composition (genomic traits)

The sequence composition describes the relative abundance or occurrence of short strings of nucleotide or amino acid. By far, the k-mer frequency has been widely used in various tasks, like taxonomy classification [?], sequences annotation [?], and host prediction [?]. Based on the length, the k-mer frequency can be defined in different formats. Referring to the previous works on reservoir determination [? ?], we define the composition by the following equations.

$$P_x = n_x / \sum_x n_x \tag{1}$$

$$P_{xy} = \frac{n_{xy} / \sum_{x,y} n_{xy}}{P_x * P_y} \tag{2}$$

$$P_{xyz} = \frac{n_{xyz}}{n_A} \tag{3}$$

where x, y, and z are nucleotide and the codon xyz encodes the amino acid A,

$$P_A = \frac{n_A}{\sum n_A} \tag{4}$$

$$CPS_{x_1y_1z_1,x_2y_2z_2} = \frac{n_{x_1y_1z_1x_2y_2z_2}}{n_{AB} * P_{x_1y_1z_1} * P_{x_2y_2z_2}} \tag{5}$$

where the codon $x_1y_1z_1$ encodes the amino acid A and the adjacent codon $x_2y_2z_2$ encodes B. The occurrence is denoted as n.

To implement normalization, any zero value or missing values are replaced with a small number (1e-4) as a default. and a log2 transformation is applied to all values. Generally, when the value is positive the corresponding feature is over-represented in the genome; otherwise, the feature is deem to be under-represented.

Features from sequence alignment

We introduced sequence alignment score as a feature vector of the query. Specifically, the reference sequences are categorized into different groups by their hosts and we aligned the query against the references. The highest alignment score of each group were kept as the potential association between the query and the corresponding host. Hence, the query will be represent as a vector of m*1, where

m is the number of host labels. If no alignment is found, we set the association to be one. A log10 transformation is applied to all scores. In this research, we used BLASTN as the default alignment tool. Instead of k-best matches, which tends to be affected by the data imbalance, we only consider the best alignment of each group.

Performance evaluation metrics

The collected 14,500 virus records belong to 30 virus orders. As described in Section Label screening, for each virus order, we examine their host label distribution. We found that 12 virus orders exclusively infected a specific host group, and we directly assigned host labels to them. These virus orders include Patatavirales, Cryppavirales, Jingchuvirales, Nodamuvirales, Wolframvirales, Mindivirales, Norzivirales, Serpentovirales, Muvirales, Yadokarivirales, Goujianvirales, and Timlovirales. The remaining 18 virus orders involved infections across multiple host groups. Among these 18 orders, thirteen received labels in the second layer of the host phylogenetic tree. Accordingly, we conducted host prediction and evaluation for the corresponding virus orders and host groups.

To evaluate the performance of VirHost, we employed various metrics, including accuracy, precision, and prediction rate. Accuracy serves as a fundamental metric, representing the proportion of correctly predicted queries out of the total number of queries. To provide a nuanced assessment of VirHost's performance across different taxonomic levels, we introduced family-wise accuracy and genus-wise accuracy, which are computed by averaging the accuracy of respective virus families and genera. Additionally, prediction rate quantifies the ratio of output predictions to the total number of queries, while precision captures the ratio of correctly predicted queries to the total number of output predictions. By employing these metrics, we aim to offer a comprehensive and granular evaluation of VirHost's performance, enabling a robust analysis of its predictive capabilities and strengths.

Result

We conducted comprehensive benchmark experiments on different scenarios to evaluate the performance of VirHost. First, we compared various feature sets and their combinations by 5 fold cross-validation. The comparison showed that the subset of genomic traits outperformed other features. We also assessed the features' contribution by machine learning strategies and validated the choice of genomic traits. We then evaluated different learning architectures, among which XGBoost achieved the highest accuracy and thus was chosen as the default architecture. Second, we focused on host prediction for novel RNA viruses. Specifically, we evaluated VirHost's performance on novel viruses by conducting leave-one-taxon-out experiments. The results of these experiments demonstrated that VirHost can be effectively applied across the diverse landscape of viruses, without being limited to specific viral types. Finally, to show the accuracy and utility of VirHost in real experiments, we collected some recently identified viruses by researches and tested VirHost's performance on different host groups.

Assessment via cross-validation

In this experiment, we follow the standard evaluation strategy in machine learning to examine the performance of different types of features and learning models. There are many types of features that may help host prediction. We first evaluated the different feature sets and their combinations by stratified 5-fold cross-validation. For each virus order, we stratified the virus into non-overlapping 5-fold by host labels, trained models using 4 out of 5 folds, and tested them in the remaining one. This assessement was performanced on each fold and we presented the overall performance on all 5 folds.

We tested 11 feature sets by ensemble learning, XGBoost. To begin with, we evaluated the classification validity of each feature set without combinations. Referring to Babayan [?], we regarded nucleotide preference, dinucleotide preference, codon usage, codon pair bias, and amino acid usage as one feature set, named as *"Bias"* here. Considering that most RNA virus are shorted than 50 kbp and there are 3,904 codon pairs (excluding pairs led by stop codons), which is a high-dimensional feature compared to the limited sequences records, we made a subset feature from Bias by excluding the codon pair bias, named as *"sBias"* (subset). Besides the bias-related features, we evaluated 9 more types of features, including BLASTN (best hit), digital signal processing-based Structural Patterns (M-SP), the frequency of 6-mer, 7-mer, 8-mer, amino acid 3-mer (AA3) , AA4, Physio-chemical 5-mer (PC5), and PC6 in [? ?]. M-SP involved in generating the Fourier transformation (FT) of the biological sequences, computing the correlation coefficients among the FT, and obtaining the distances matrix of sequences through the correlation coefficients [?]. A detailed description of the benchmarked feature can be found in the Supplementary Information (section 2). Then, we assessed feature set combinations among Bias, BLASTN, and M-SP. DeepHoF [?], a host prediction tool based on convolutional neural network, provided the classification of viruses associated with plant, germ, invertebrate, human and other vertebrate, where germ include bacteria and fungi. We included it in the benchmark assessment in Layer 1 by merging "human" and "other vertebrate" to be Chordata.

As shown in Fig. ?? (A,B), when using each of the 11 feature sets, the classifier with feature *sBias* achieved the highest accuracy of 92.38% and 83.92% for each virus order in L1 and L2, respectively. The models with feature Bias and BLASTN rank the second and the third in performance. The model with *Bias* got accuracy of 90.89% and 82.14%, while using BLASTN got 89.64% and 81.57%. The accuracy difference between *sBias* and *Bias* drew our attention and we calculated the feature contribution to classification performance, as shown in the Supplementary Information (section 3), Table S1 and Fig. S1. The result indicated that most codon pair scores did not contribute to the prediction, which is consistent with previous studies [?]. This can be explained by the sparsity of the codon pair in RNA viruses. Besides, the M-SP traits, achieving accuracy of 87.14% and 76.93%, did not provide accurate predictions when used solely.

Out of the 11 feature sets, *Bias*, *sBias*, and BLASTN scores show promising results in the cross-validation experiment. Hence, we further evaluated their combinations and reported the results in Fig. ?? (C,D). When combined, the models with *sBias_Blast* achieved the best order-wise accuracy of 93.99% and 88.01%. The combination significantly improved the prediction accuracy. While *Bias_Blast* and *Bias_Blast_Dsp* (the combination of *Bias*, BLASTN, and M-SP as [?]) are the second and third best group, they got little difference in accuracy (93.14% and 86.98% for *Bias_Blast*, 93.14% and 86.96% for *Bias_Blast_Dsp*). The result implied that the M-SP traits did not benefit the prediction of RNA viruses' host significantly. On the other hand, the drops of accuracy from *sBias* to *Bias* and that from *sBias_Blast* to *Bias_Blast* validated the feature reduction that excludes the codon pair features.

An important observation is that using BLASTN achieves quite comparable host prediction results as our learning-based methods. We thus further analyzed this. First, to ensure that the improvements observed in the host prediction models are not due to chance events, we conducted a further comparison among models based on different feature sets. This comparison involved examining the accuracy distribution across virus orders, as detailed in Fig. S2. Through the one-sided Wilcoxon test, we demonstrated that the observed improvements in host prediction accuracy are statistically significant and not merely a result of random chance.

Second, it is worth mentioning that when employing a random data partitioning strategy (5-fold cross validation), it is not unex-

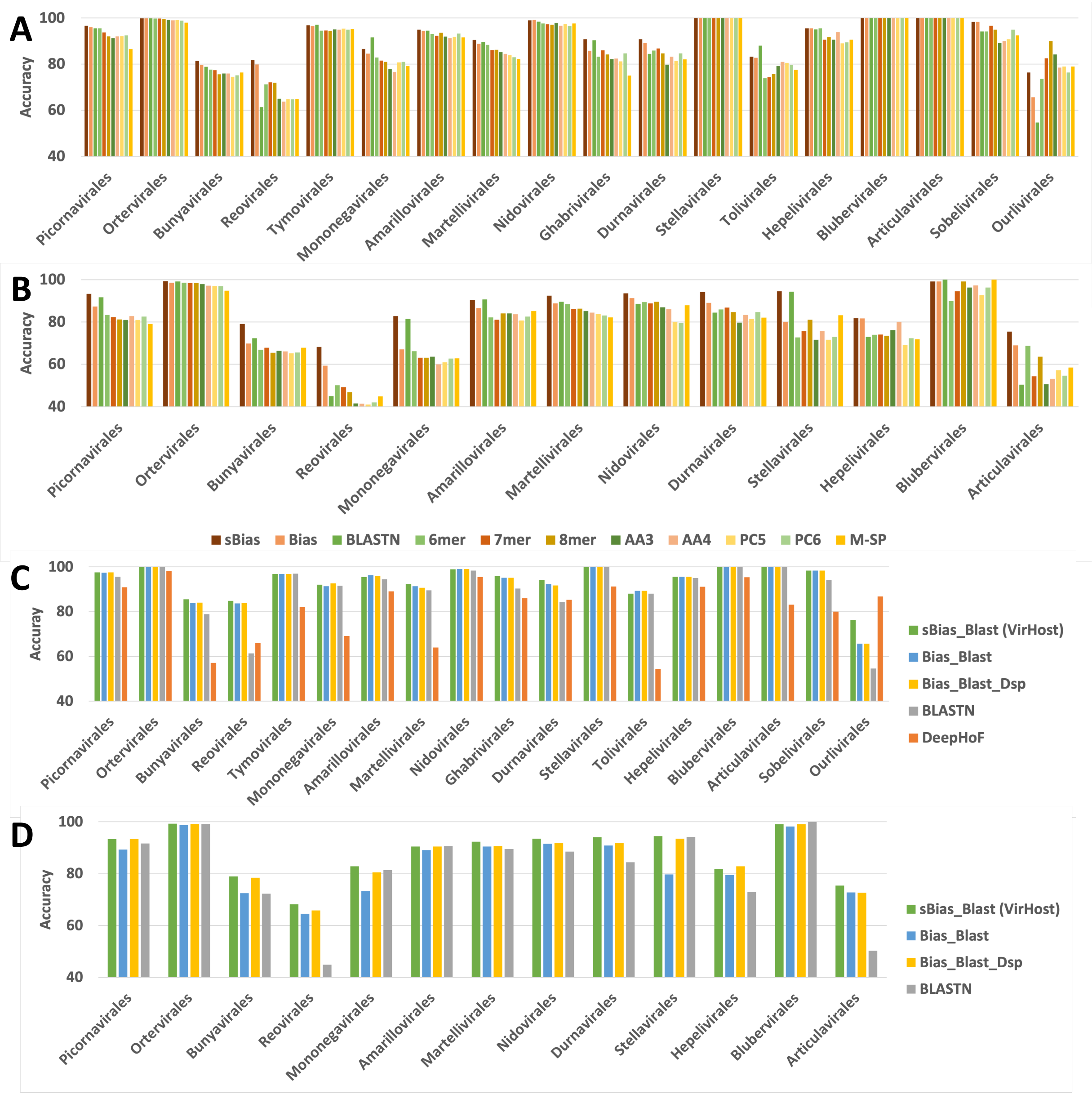

Figure 2. Reservoir prediction performance of different feature sets in Layer 1 (A) and Layer 2 (B). Here, Bias denotes a set of genomic traits, including nucleotide preference, dinucleotide preference, codon usage, codon pair bias, and amino acid usage, while sBias denotes a subset which excludes the codon pair bias. Reservoir prediction performance of different feature combinations in Layer 1 (C) and Layer 2 (D). Here, sBias_Blast is the combination of sBias and BLASTN. Bias_Blast is the combination of Bias and BLASTN. Bias_Blast_Dsp is the combination of Bias, BLASTN, and M-SP. DeepHoF is designed to predict hosts at the kingdom and phylum level (Layer 1), and its output is transformed to corresponding labels (Germ score to be Fungi; Human score and Vertebrate score to be Chordata). X-axis: virus orders sorted by the size (from largest to smallest). The performance comparison in Layer 2 considers the errors from both Layer 1 and Layer 2. Among the 18 orders, thirteen can be further classified in Layer 2. Therefore, we focused our performance evaluation solely on Layer 2 within these thirteen orders.

pected to observe that the predictive performance of BLAST yields similar results as learning-based methods. One main reason for this is that the test viruses, when randomly partitioned, tend to exhibit high similarity to certain viruses in the training data, resulting in a less challenging test scenario. Our next set of experiments will explore more realistic usage scenarios.

Handling Out-of-Distribution host labels

As our primary objective is to predict the natural hosts of metagenomic-assembled RNA viruses, we have designed a comprehensive label list that includes four eukaryotic kingdoms and one prokaryotic domain. However, considering the continuous emergence of novel RNA viruses, there are instances where the queried viruses may infect hosts outside the label list. To prioritize precision, we opt to reject virus queries that may not infect the target hosts, even if it leads to a lower prediction rate. To determine the trade-off between prediction rate and precision for both our tool and BLASTN, we analyzed the distribution of prediction scores, which is shown in Supplementary Information (section 4) and Fig. S3. Our analysis revealed that, while predicting hosts for the same number of queries, VirHost achieved higher precision compared to BLASTN. Consequently, we provide users with an empirical prediction score cutoff for each virus order, allowing them to choose predictions with greater confidence. This empirical approach enables users to obtain more reliable predictions.

The comparison among machine learning architectures

Finally, we conducted an evaluation of several learning architectures, encompassing XGBoost, Gradient Boosting Decision Tree (GBDT), Random Forest (RF), Support Vector Machine with RBF ker-

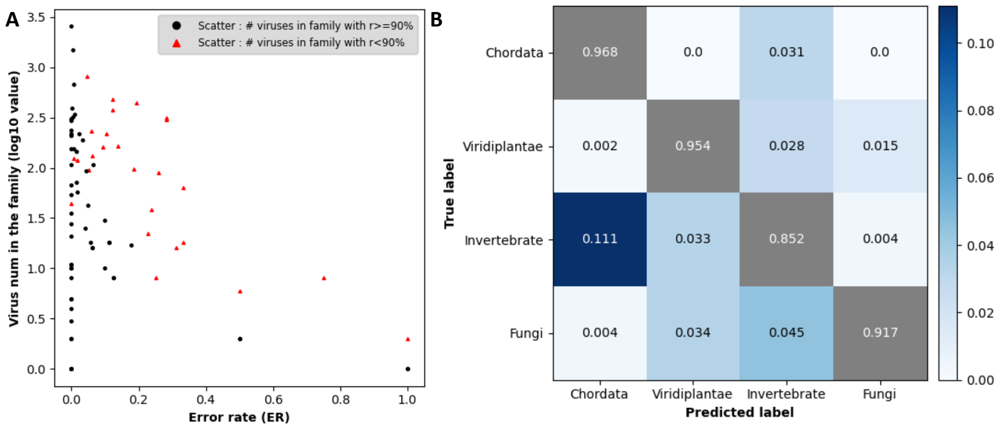

Figure 3. (A) The error rate distribution of VirHost across different virus families in Layer 1. The dots denote the number of virus members in each family (Y-axis) and its corresponding error rate (X-axis). (B) The confusion matrix of VirHost's prediction in Layer 1. The values denote the percentage of mis-classified members, which were normalized to total number of the corresponding groups. The viruses infecting bacteria are limited to specific virus orders, which exclusively infect bacteria. Hence, we do not visualize bacteria here.

nel (SVM), Logistic Regression (LR), K-Nearest Neighbors (KNN), and Gaussian Naive Bayes (GNB). Using the scikit-learn package [?], default parameters were employed to train these models, and their performance was assessed using accuracy as the evaluation metric. As demonstrated in Fig. S4, XGBoost exhibited the highest accuracy of 94.0% (L1) and 88.0% (L2), while the second best architecture (RF) achieved those of 93.3% and 87.5%, and the third best one (GBDT) got 92.7% and 86.2%.

Collectively, the model trained with XGBoost and the combination of *sBias* and BLASTN, achieved the outperforming accuracy among various learning architectures, feature sets, virus groups, and host ranks.

Family-wise analysis reveals the potential host switch

In comparison to viruses within the same order, viruses in the same family generally have more consistent host ranges. We investigated host distribution of each virus family and visualized the errors rate of virus families in the first layer in Fig. ??A. In the 18 evaluated virus orders, there are 83 families and 13,238 evaluated viruses. In the first layer, the family-wise average accuracy is 87.55%, meaning that 658 out of evaluated 13,238 viruses got wrong assignments of hosts. It is observed that the misclassification of VirHost mainly distributes to those virus families with mixed host groups. To better visualize the trend, we counted the dominant host labels in every family and the ratio of members infecting the dominant hosts, denoted as $\mathtt{r}$. As a measure of homogeneity, $\mathtt{r}$ will have a small value if a family mainly contains viruses infecting different host groups. Otherwise, a virus family infecting only one dominant host group will get an $\mathtt{r}$ value close to 1. To examine the homogeneity values of various families, we set $\mathtt{r}$ as 90% as the threshold for high and low host homogeneity. Out of the 83 RNA virus families, there are 57 families with over 90% of their members infecting a unique host group ($\mathtt{r} \geq 90\%$) and 26 families infecting multiple host groups ($\mathtt{r} < 90\%$). In 658 error cases, only 57 (8.66%) came from the former ($\mathtt{r} \geq 90\%$), while 593 (90.12%) belong to the latter case ($\mathtt{r} < 90\%$), implying that these wrong prediction are mainly from viruses with multiple hosts across phylum. For instances, Peribunyaviridae ($\mathtt{r}$ = 56.7%), Phenuiviridae ($\mathtt{r}$ = 53%), Nairoviridae $\mathtt{r}$ = 70.8%) are virus families in Bunyavirales. They contains members that are well known for causing vector-boned diseases [? ? ?]. VirHost achieved accuracy of 71.34%, 70.67%, and 74.16% for the three families, which is lower than the family-wise average accuracy.

Additionally, we generated the confusion matrix to evaluate the predictions made by VirHost, as shown in Fig. ??B. Analysis of the matrix revealed that viruses infecting chordates and Viridiplantae (plants) could be accurately classified. Only 0.2% of viruses infecting Viridiplantae were misclassified as chordate-associated, and no viruses infecting chordates were predicted to infect Viridiplantae. Similarly, viruses infecting chordates and fungi could be clearly distinguished. However, misclassification predominantly occurred among invertebrate-associated queries, with 11.1% of invertebrate-infecting viruses predicted to infect chordates, and 3.1% of chordate-infecting viruses predicted to infect invertebrates. These predictions strongly suggest the presence of potential vector-borne viruses within the dataset. Additionally, errors occurred when distinguishing between viruses that infect invertebrates and those that infect plants and fungi. This confusion may arise due to the contact and dietary interactions between invertebrates and plants or fungi [? ?]. Furthermore, there is some overlap between the viruses infecting fungi and those infecting plants, which could result from the symbiotic relationships between certain plants and fungi [?]. Although the focus of this work is placed on predicting the reservoir hosts, the vector-borne viruses still have a great influence on the performance of host prediction.

Host prediction for novel RNA viruses

With fast accumulated RNA viruses from the environmental sequencing samples, determining reservoirs of novel viruses is becoming more important. In the second part, we evaluated the capability of VirHost to identify hosts of novel viruses using leave-one-genus-out strategy. Specifically, we train the model without including specific genera and then assess its performance on those genera to mimic the situation where a novel query, particularly with unknown genus label, is used as input. In this benchmark, we compared VirHost against the alignment-based method, BLASTN, and two null models (Null 1 and Null 2). Null 1 randomly assigns the host labels following the host label distribution of reference viruses in the training data, while Null 2 determines the query's host using the dominant host label in the training data. The result is shown in Fig. ??. As DeepHoF does not allow us to retrain their model, we cannot include DeepHoF in this experiment.

Some genera might have different host tropisms with their closely-related viruses under the same order or family, which poses a great challenge in predicting their reservoirs. Below we provide a comprehensive discussion of these challenging cases. We counted the dominant host labels in Layer 1 for each virus order, family, and genus. According to the difference between the host tropisms of the genus and its corresponding order or family, we consider three chal-

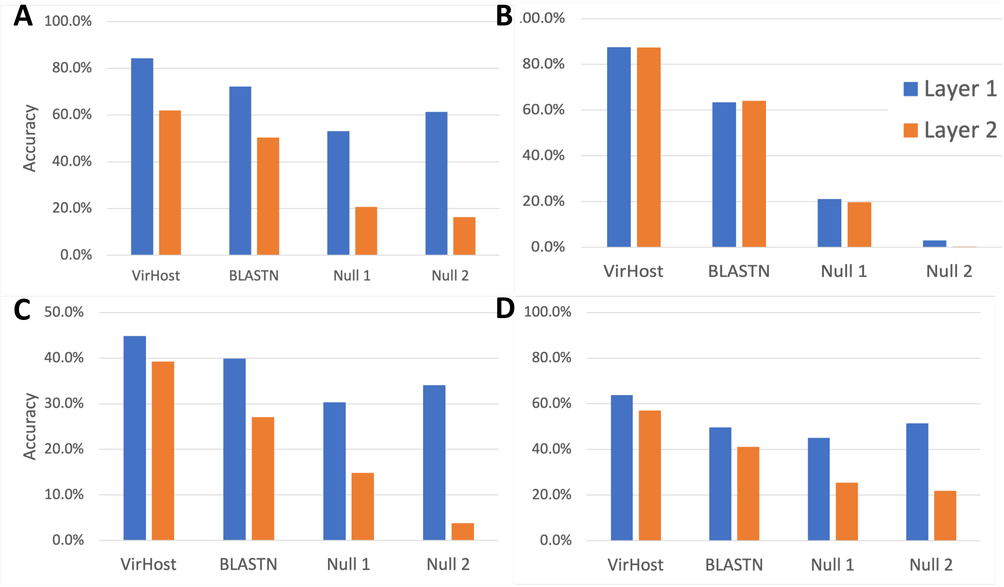

Figure 4. The performance comparison of different strategies in the leave-one-genus-out experiment. The figures demonstrate the average accuracy of A) all 448 genera, B) 138 genera of case 1, whose dominant host label is different from their order's, C) 50 genera of case 2, whose dominant host label is different from its family's, D) 56 genera of cases 3 with members infecting hosts of different kingdoms or phyla. Null 1 randomly assigns the host labels following the distribution of references. Null 2 determines the query's host by the dominant host label in references.

lenging cases. **Case 1)**: the genus's dominant host label is different from its order's. A typical case is Nepovirus in Picornavirales. While Nepovirus is a plant-infecting virus genus, seventy-four percent of members of the Picornavirales infect chordates. **Case 2)** the genus's dominant host label is different from its family's, such as Seadornavirus in Sedoreoviridae. While mosquitoes are the natural host of Seadornavirus, Spinareoviridae mainly infect chordates. Some genera in case 2 also belong to case 1, indicating that the host tropism of these genera are divergent from their homologous viruses within the same family and order. **Case 3)**: the members of the genus infecting hosts from divergent kingdoms or phyla. By discussing the challenging cases, we expect to exhibit the performance of VirHost in different situations more comprehensively.

In our dataset, there are 448 virus genera across 18 orders. VirHost achieved the highest genus-wise average accuracy of 84.3% and 61.9% for L1 and L2, respectively, outperforming BLASTN by 12.1% and 11.5%. There are 138 genera as case 1, whose dominant host labels varied from their order's, 50 genera as case 2, whose dominant host labels differ from their family's, and 56 genera infecting hosts from multiple phyla. VirHost achieved the best result in various cases. Specifically, in 138 genera of case 1, VirHost got accuracy of 87.5%(L1) and 87.4%(L2), outperforming BLASTN by 24.1% and 23.4%. In 50 genera of case 2, VirHost got accuracy of 44.8%(L1) and 39.2%(L2), surpassing BLASTN by 4.9% and 12.1%. In 56 genera with divergent host groups, VirHost achieved accuracy of 63.8%(L1) and 57.0%(L2), exceeding BLASTN by 14.1% and 15.9%. The improvement of VirHost likely result from the utilization of genomic traits and the superiority of the machine learning method.

Experiments on recently identified viruses

Finally, we retrained VirHost on all the reference viruses and assessed its accuracy on identifying hosts for recently identified viruses, whose hosts are derived based on experimental evidence. This process was designed to replicate scenarios that potential users of VirHost might typically encounter.

The four datasets associate with plants, invertebrate, fungi, and fishes (chordates), respectively, corresponding to the first layer's host labels in our tool. Each dataset comprised of viruses sequenced

Table 2. The statistics regarding taxonomic groups and host labels of the newly sequenced datasets.

	# viruses	# virus orders	Host group	# host classes	# host orders
Dataset 1	21	5	Plant	1	6
Dataset 2	15	3	Invertebrate	2	4
Dataset 3	69	9	Fungi	4	6
Dataset 4	21	6	Chordata - Fish	1	2

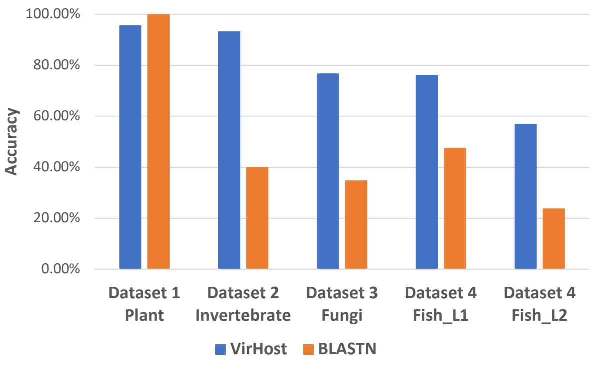

Figure 5. Host prediction accuracy of VirHost and BLASTN on recently identified virus datasets. In dataset 4, L1 and L2 denote Layer 1 and Layer 2, respectively.

from different hosts. The statistics is listed in Table ??. In all four datasets, VirHost achieved better or comparable performance than BLASTN, as shown in Fig. ??. The first dataset consists of 21 viruses, whose hosts are from 6 orders under Magnoliopsida plants [? ? ? ? ? ? ?]. VirHost predicted one case to be fungi-associated, which is a member of Alphapartitivirus, a genus that infecting both plant and fungi. This may suggest that the query has the ability to infect fungi. The second dataset includes 15 viruses, infecting hosts of 4 Arthropoda orders [? ? ? ?]. VirHost achieved a high accuracy of 93.3% while BLASTN got 40%. The only misclassified query obtained balanced prediction score of VirHost between Chordata

and Invertebrate, which implied its potential to infect vertebrate. The third dataset involves 69 viruses from 9 orders and their hosts spanned 6 fungi orders [? ? ? ? ? ? ?]. VirHost achieved an accuracy of 76.8%, outperforming BLASTN by 28.6%. Finally, the fourth dataset comprises 21 viruses from 6 orders, infecting seahorses and salmon [? ?]. We achieved accuracy of 76.2% and 57.1% in Layer 1 and Layer 2, respectively, outperforming BLASTN by 28.6% and 33.3%.

Discussion

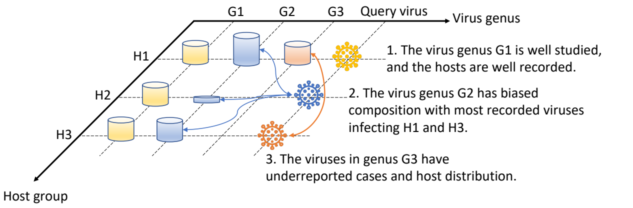

Figure 6. A visualization illustrating the potential sequencing bias in the reference database, posing a challenge to the prediction of reservoirs. On the virus genus axis, there are three reference genera represented by colors: yellow for G1, blue for G2, and red for G3. The host group axis consists of three distinct host groups (H1, H2, H3) present in the reference database. The cylinders at the intersections of the dashed lines represent viruses belonging to the respective genera that infect the corresponding host groups. The height of each cylinder indicates the relative number of viruses. G1 is extensively studied, and the hosts associated with it are well-documented. G2 and G3 are under studied, resulting in limited information about their hosts.

The application of metagenomic sequencing promotes the identification of novel viruses from host-associated and environmental samples of a diverse set of ecosystems. However, the high diversity of the potential hosts, the sampling method and location, and the heterogeneity of metagenomic sequencing make it hard to determine reservoirs of the detected viruses, which is a critical step for pandemics surveillance and One Health. In this study, we compared various features used in host prediction of RNA viruses and developed a tool, VirHost, that allows a fast and reliable reservoir prediction by only using the virus sequences. Compared to the laborious and expensive process of host verification in wet lab, VirHost offers a time and resource-effective strategy. By integrating genome traits and sequence homology of viruses, VirHost achieved higher accuracy than the alignment based method. Instead of focusing on vertebrate associated viruses, we extend the host range to plants, fungi and bacteria. With the increasing available viruses' host annotations, VirHost can be can be easily scaled to more viruses and hosts.

Despite the potential implications of our study, it is important to acknowledge the challenges that lie ahead for future research in this field. Two particular challenges warrant careful consideration: the unexpected host switch and the existence of multiple hosts. The long-term co-evolution between hosts and viruses may shape the genomic traits of viruses in a way that enables the distinction of their reservoirs. However, when viruses transfer to new hosts, their genome heritage may no longer reflect their host tropism, and the selection force may not immediately reveal their potential. To anticipate the phenomenon of host switching, additional information is required, including the host lifestyle, virus infection patterns, and virus-host protein-protein interactions. Obtaining such information can provide a more comprehensive understanding of the factors influencing host switching and improve our ability to forecast and respond to pandemics.

Turning our attention to the second challenge, we must address the inherent limitations of VirHost, which is designed to predict the viruses' reservoirs. In specific cases where viruses can infect multiple hosts, it requires more comprehensive analysis, like single nucleotide variants, to draw the host range accurately. On the other hand, due to the sequencing bias in the database, there exists a knowledge gap that some potential hosts of detected viruses have yet to record. We discussed how the sequencing bias towards different hosts can lead to misclassification, as shown in Fig. ??. Our focus was primarily on the genus-level analysis. To account for the sequencing bias, we categorized virus genera into two groups: well-recorded cases and under-reported cases. Well-recorded cases refer to virus genera that have been exhaustively sequenced in all of their hosts without any sequencing bias, represented by virus genus G1. The availability of a complete reference database greatly benefits the host prediction of new queries. On the contrary, the under-reported cases depict the virus genera whose diversity is under-studied among their hosts, such as the virus genera G2 and G3. In instances where the query virus infects a host that is under-reported in the reference database, the prediction relies heavily on the available hosts and thus may be biased towards hosts that have been extensively recorded. For example, if there is a virus from genus G3 infecting host group H3, but the host recorded in the reference database is H1, there is a high possibility that it will be misclassified as infecting H1. Therefore, the incomplete host range record can negatively impact the accuracy of host prediction. To mitigate this bias, it is crucial to continue expanding and updating the reference database by including data from under-reported hosts. Further understanding of these viruses will enhance the prediction and help to determine the boundary of infection.

Availability of source code and requirements

- Project name: VirHost
- Project home page: https://github.com/GreyGuoweiChen/VirHost
- Operating system(s): Platform independent
- Programming language: Python
- Other requirements: Python 3.8, BLAST 2.12.0+, Prodigal 2.6.3+, xgboost 2.0.3, pandas 2.0.3, biopython 1.83, numpy 1.23.5
- License: MIT license
- RRID: SCR_025061

Data availability

Additional Files

Supplementary Table S1. The feature contribution of genomic traits to host prediction was assessed using XGBoost models across different virus orders. The contribution is measured as the feature importance. The fold between the observed and expected importance is calculated as the ratio between the mean of importance and the reciprocal of the total number of genomic traits (4041). A higher fold value indicates a greater importance demonstrated by the feature.

Supplementary Fig. S1. The accuracy of models using genomic traits in the Layer 1 when considering different number of codon pair bias.

Supplementary Fig. S2. The accuracy distribution of models using different feature set across virus orders. To demonstrate the effectiveness of our method, we evaluated the median accuracy of different models using Wilcoxon test. Specifically, we conducted one-sided Wilcoxon test to compare our method with BLASTN (P1) and Bias_Blast (P2). A small p-value ($P < 0.05$) suggests strong evidence to support the alternative hypothesis that the median accuracy of our method is significantly larger than that of the compared methods.

Supplementary Fig. S3. The Precision-Prediction_rate curve of VirHost and BLASTN in 9 virus orders that contain more than 500

viruses. A curve closer to the upper-right corner indicates better model performance. The blue dot represents VirHost's performance when applying the empirical prediction score cutoff. The red dot represents the performance of BLASTN's final prediction using the best alignment strategy. The prediction rate is lower than 100% due to the inability to align some query sequences with the reference sequences. Diff denotes the precision difference between VirHost and BLASTN at the same prediction rate.

Supplementary Fig. S4. The average accuracy of models using different learning architectures with the same features as VirHost.

Supplementary Information Section 1: Details of data collection. Section 2: The description of benchmark features. Section 3: Feature selection. Section 4: The trade-off between prediction rate and precision.

Competing Interests

The authors declare that they have no competing interests.

Funding

The study is supported by Hong Kong Research Grants Council (RGC) General Research Fund (GRF) [11206819, 11217521] and Hainan Provincial Natural Science Foundation of China [324CXTD435].

Author's Contributions

GC contributed to the data collection and analysis, experiment execution, framework design, and paper writing. YS conceptualized the study. JJ and YS oversaw the project. All authors reviewed, contributed to, and approved the manuscript.

 Click here to access/download;Table;Table_1.xlsx

Table 1: The virus order and host distribution after the label screening. Each row represents

		Layer 1			
**order**	**num**	**Chordata**	**Invertebrate**	**Viridiplantae**	**Fungi**
**Ortervirales**	**2764**	92.0%	-	8.0%	-
**Picornavirales**	**2647**	74.2%	12.2%	13.6%	-
**Bunyavirales**	**1524**	52.4%	31.6%	15.2%	0.9%
**Tymovirales**	**1042**	-	2.6%	94.3%	3.1%
**Reovirales**	**1034**	52.9%	33.8%	9.7%	3.6%
**Amarillovirales**	**817**	85.2%	14.8%	-	-
**Mononegavirales**	**758**	57.5%	27.8%	11.3%	3.3%
**Martellivirales**	**670**	5.1%	6.3%	73.7%	14.9%
**Nidovirales**	**622**	94.4%	5.6%	-	-
**Patatavirales**	**558**	-	-	100.0%	-
**Ghabrivirales**	**393**	-	14.0%	9.9%	76.1%
**Durnavirales**	**340**	5.0%	-	32.4%	62.6%
**Stellavirales**	**296**	100.0%	-	-	-
**Tolivirales**	**226**	-	15.0%	73.9%	11.1%
**Hepelivirales**	**181**	80.1%	10.5%	9.4%	-
**Sobelivirales**	**120**	-	12.5%	87.5%	-
**Blubervirales**	**108**	100.0%	-	-	-
**Cryppavirales**	**80**	-	-	-	100.0%
**Articulavirales**	**77**	100.0%	-	-	-
**Jingchuvirales**	**61**	-	100.0%	-	-
**Nodamuvirales**	**42**	-	100.0%	-	-
**Ourlivirales**	**38**	-	-	26.3%	73.7%
**Wolframvirales**	**23**	-	-	-	100.0%
**Mindivirales**	**22**	-	-	-	-
**Norzivirales**	**21**	-	-	-	-
**Serpentovirales**	**16**	-	-	100.0%	-
**Muvirales**	**9**	-	100.0%	-	-
**Yadokarivirales**	**7**	-	-	-	100.0%
**Goujianvirales**	**3**	-	100.0%	-	-
**Timlovirales**	**1**	-	-	-	-
**sum**	**14500**				

; the host distribution of a virus order. While the column "num" shows the total num

Bacteria	Primates	Rodentia	Carnivora	Artiodactyla	Chiroptera
-	85.9%	0.9%	0.9%	2.0%	-
-	40.2%	4.1%	4.2%	14.5%	2.6%
-	18.0%	17.6%	-	1.6%	1.4%
-	-	-	-	-	-
-	9.9%	1.3%	2.9%	9.0%	5.5%
-	54.0%	9.7%	-	10.0%	4.0%
-	11.3%	6.5%	4.0%	5.4%	11.5%
-	5.1%				
-	4.8%	5.3%	5.6%	36.3%	17.4%
-	-	-	-	-	-
-	-	-	-	-	-
-	5.0%				
-	10.1%	12.8%	8.4%	33.1%	5.1%
-	-	-	-	-	-
-	32.0%	16.6%	-	16.6%	-
-	-	-	-	-	-
-	75.9%	-	-	-	14.8%
-	-	-	-	-	-
-	59.7%				
-	-	-	-	-	-
-	-	-	-	-	-
-	-	-	-	-	-
-	-	-	-	-	-
100.0%	-	-	-	-	-
100.0%	-	-	-	-	-
-	-	-	-	-	-
-	-	-	-	-	-
-	-	-	-	-	-
-	-	-	-	-	-
100.0%	-	-	-	-	-

ıber of viruses in the order, the following columns represent the percent of viruses infecting the corresp

.ayer 2

Other Mammalia	Aves	Reptilia	Amphibia	Fish
0.7%	1.6%	-	-	-
1.7%	3.9%	0.9%	-	2.0%
5.3%	1.7%	5.6%	-	1.2%
-	-	-	-	-
3.6%	9.6%	1.3%	-	10.0%
4.2%	1.7%	-	-	1.6%
2.4%	9.0%	1.6%	-	5.9%
	-	-	-	-
5.6%	13.5%	5.8%	-	-
-	-	-	-	-
-	-	-	-	-
	-	-	-	-
-	16.2%	-	4.4%	9.8%
-	-	-	-	-
8.8%	6.1%	-	-	-
-	-	-	-	-
-	9.3%	-	-	-
-	-	-	-	-
	16.9%	-	-	23.4%
-	-	-	-	-
-	-	-	-	-
-	-	-	-	-
-	-	-	-	-
-	-	-	-	-
-	-	-	-	-
-	-	-	-	-
-	-	-	-	-
-	-	-	-	-
-	-	-	-	-
-	-	-	-	-

onding hosts. Layer 2 consists of the Chordata subgroups from Layer 1, therefore the sum of valu

ues in the second layer is equal to the value of "Chordata". In cases (Martellivirales, Durnavirales,

, Articulavirales) where mammalian viruses are less than 50, we merge mammalian members intc

ɔ a single node, Mammalia.

 Click here to access/download;Table;Table_2.xlsx

Table 2: The statistics regarding taxonomic groups and host labels of the newly sequenced datas

	# viruses	# virus orders	Host group	# host classes	# host orders
Dataset 1	21	5	Plant	1	6
Dataset 2	15	3	Invertebrate	2	4
Dataset 3	69	9	Fungi	4	6
Dataset 4	21	6	Chordata - Fish	1	2

sets.

Click here to access/download
Supplementary Material
Supplementary_Table_S1.pdf

Click here to access/download
Supplementary Material
Supplementary_Fig_S1.pdf

Click here to access/download
Supplementary Material
Supplementary_Fig_S2.pdf

Click here to access/download
Supplementary Material
Supplementary_Fig_S3.pdf

Click here to access/download
Supplementary Material
Supplementary_Fig_S4.pdf

Supplementary Material

Click here to access/download
Supplementary Material
Supplementary information.pdf
