# Supplementary material for: RNAVirHost: a machine learning–based method for predicting hosts of RNA viruses through viral genomes
Source: Gigascience. 2024 Aug 22;13:giae059. doi: 10.1093/gigascience/giae059 (PMC11340644; doi:10.1093/gigascience/giae059)
Supplement: giae059_GIGA-D-24-00081_Revision_1 [file giae059_giga-d-24-00081_revision_1.pdf]

GigaScience

RNAVirHost: a machine learning-based method for predicting hosts of RNA viruses through viral genomes

--Manuscript Draft--

**Manuscript Number:**	GIGA-D-24-00081R1
**Full Title:**	RNAVirHost: a machine learning-based method for predicting hosts of RNA viruses through viral genomes
**Article Type:**	Research
**Funding Information:**	Natural Science Foundation of Hainan Province (324CXTD435) — Dr. Jingzhe Jiang<br>Research Grants Council of Hong Kong (11206819, 11217521) — Dr. Yanni Sun
**Abstract:**	Background: The high-throughput sequencing technologies have revolutionized the identification of novel RNA viruses. Given that viruses are infectious agents, identifying hosts of these new viruses carries significant implications for public health and provides valuable insights into the dynamics of the microbiome. However, determining the hosts of these newly discovered viruses is not always straightforward, especially in the case of viruses detected in environmental samples. Even for host-associated samples, it is not always correct to assign the sample origin as the host of the identified viruses. The process of assigning hosts to RNA viruses remains challenging due to their high mutation rates and vast diversity.<br>Results: In this study, we introduce RNAVirHost, a machine learning-based tool that predicts the hosts of RNA viruses solely based on viral genomes. RNAVirHost is a hierarchical classification framework that predicts hosts at different taxonomic levels. We demonstrate the superior accuracy of RNAVirHost in predicting hosts of RNA viruses through comprehensive comparisons with various state-of-the-art techniques. When applying to viruses from novel genera, RNAVirHost achieved the highest accuracy of 84.3%, outperforming the alignment-based strategy by 12.1%.<br>Conclusions: The application of machine learning models has proven beneficial in predicting hosts of RNA viruses. By integrating genomic traits and sequence homologies, RNAVirHost provides a cost-effective and efficient strategy for host prediction. We believe that RNAVirHost can greatly assist in RNA virus analyses and contribute to pandemic surveillance.
**Corresponding Author:**	Yanni Sun<br>City University of Hong Kong<br>Hong Kong, HONG KONG
**Corresponding Author Secondary Information:**	
**Corresponding Author's Institution:**	City University of Hong Kong
**Corresponding Author's Secondary Institution:**	
**First Author:**	Guowei Chen, Ph.D.
**First Author Secondary Information:**	
**Order of Authors:**	Guowei Chen, Ph.D.<br>Yanni Sun<br>Jingzhe Jiang
**Order of Authors Secondary Information:**	
**Response to Reviewers:**	Dear Editor and Reviewers,<br>We would like to thank you for your careful reading of our paper and the valuable comments. We have addressed the comments of all reviewers, with their comments in italic and our responses in blue. The revised figures and descriptions were added to

Powered by Editorial Manager® and ProduXion Manager® from Aries Systems Corporation

the main manuscript and the supplementary file. The changes in the main draft and the supplementary file are highlighted in red. We also submitted a response letter with refined figures and typesetting as a supplementary material for your convenience.

Before we provide the detailed responses, we would like to summarize the major improvements or modifications below.
1.We expanded the discussion on host prediction problem to better define the challenge. This clarifies the complexities and nuances involved in this area of research, while maintaining the reliability of the study.
2.We provided more details about the benchmark datasets, including data sources, taxonomic distributions of hosts and viruses, and data generation standards, to ensure fair and transparent comparisons.
3.We further evaluated the different strategies in the data imbalanced task using the F1 score as an additional metric, providing a more comprehensive assessment of performance.
4.We conducted an additional assessment on predicting hosts using fragmented viral contigs.
5.We carefully revised and polished the manuscript and figures to enhance clarity, readability, and overall presentation.
6.We changed the tool's name to better reflect the tool's function and input requirement.

We hope that the following point-to-point responses will be clear enough to convey our ideas and address the comments of the reviewers.

Response to Reviewer 1's comments
Chen et al. have developed a new tool to predict hosts for RNA viruses. Tools like VirHost are highly beneficial for understanding the interactions between viruses and their hosts. However, I believe the current manuscript has several limitations that need to be addressed before further assessment:
Thank you for your summary. We greatly appreciate your time in reviewing our manuscript. We have revised the manuscript accordingly. Our point-by-point responses are detailed below.

1) The authors should share their training and test datasets. There is not enough information about these datasets in the manuscript.
Thank you for your valuable feedback. Following this suggestion, we have expanded the "Data Collection" section to provide more details about the reference dataset, including its sources and filtering criteria.
In the experiments on "novel RNA viruses", we generate the "novel RNA viruses" by leaving a specific genus of reference viruses as the test set. This genus is not part of the training data, thus ensuring a rigorous test of our method. Then, we trained the model without including the specific genus and assessed its performance on that genus to mimic the situation where a novel query, particularly with an unknown genus label, is used as input. The leave-one-genus-out evaluations are conducted on all virus genera (448 virus genera). As all the test sequences were generated from the reference dataset, we got the genus labels from the annotation files of Virus-Host DB and NCBI GenBank. The information can be found in section "Performance on novel RNA viruses".
For the "recently identified viruses", we collected 20 publications that focused on identifying novel viruses on specific hosts and confirmed the viral infection by laboratory measures. While fifteen of them were published after 2023, we expanded the dataset by including five more works that were published before 2023 to cover different hosts as comprehensively as possible. We manually curated the dataset to ensure that the reference dataset does not include any new viral sequences presented in the 20 works. Besides, most of the works were designed on understudied hosts, for example, Hippocampus erectus (seahorse) as fishes, various fungi, Stellaria aquatica and Cnidium officinale as plant, and Apis mellifera (Western honey bee) as Invertebrate. More details can be accessed in section "Performance on recently identified viruses".
Furthermore, for reproducibility and further analysis, we have deposited all data used in the study in GigaDB, making it readily accessible to the research community. Upon publication, the GigaDB repository will be accessible through the link provided in section "Data availability".

2) They should compare their tool with similar tools/ pipelines for its prediction performance.
Thank you for this comment. We have compared our pipeline with related methods/tools/pipelines. In the section "related work", we introduced a list of works that can be used to predict the hosts of metagenomic-assembled viruses instead of specific viruses or hosts. For those that can be used on RNA viruses, we have included them in the comparison. The corresponding publications for the benchmarked methods are: Bias [1], Bias_Blast [1], DSP [2], Bias_Blast_Dsp [2], all types of k-mer [3], and DeepHoF [4]. Except DeepHoF, other pipelines lack well-packaged software. Thus, we retrained/reimplemented their methods and renamed them based on their main features. The performance comparison with these tools can be found in Figures 2, 4, S1, and S6. More detailed information is provided in the second paragraph of "Assessment via cross-validation" section.
[1] Babayan, S. A., Orton, R. J., & Streicker, D. G. (2018). Predicting reservoir hosts and arthropod vectors from evolutionary signatures in RNA virus genomes. Science, 362(6414), 577-580.
[2] Lee, B., Smith, D. K., & Guan, Y. (2021). Alignment free sequence comparison methods and reservoir host prediction. Bioinformatics, 37(19), 3337-3342.
[3] Young, F., Rogers, S., & Robertson, D. L. (2020). Predicting host taxonomic information from viral genomes: A comparison of feature representations. PLoS computational biology, 16(5), e1007894.
[4] Guo, Q., Li, M., Wang, C., Guo, J., Jiang, X., Tan, J., ... & Zhu, H. (2021). Predicting hosts based on early SARS-CoV-2 samples and analyzing the 2020 pandemic. Scientific Reports, 11(1), 17422.

4) The manuscript could benefit from proofreading.
Thank you for your suggestions. We have carefully revised and polished the manuscript to enhance clarity.

5) I found the introduction to be unfocused and lacking enough coherence.
Thank you for your comment. Our introduction intends to provide a more comprehensive background for host prediction problem. To better convey the challenges of host prediction, we not only discussed the host range and definition of viruses but also compared different challenges of host prediction problems between DNA viruses and RNA viruses.
To improve the manuscript's readability and coherence, we have reorganized its structure and introduced a new subsection titled "Related Work." With the new changes, the organization of Introduction is: 1) introduction of RNA viruses, 2) introduction of the data for virus discovery and need for host identification, 3) problem formulation with a focus on the discussion of "hosts", 4) a comprehensive discussion of the current methods for identifying the virus host. In the subsection "Related work", we first acknowledged the experimental methods. Then, as there is a fast accumulation of prokaryotic virus host prediction tools, we briefly covered them and concluded why these tools cannot be applied to eukaryotic viruses. Then, we discussed the state-of-the-art RNA virus host prediction in two scenarios. The first scenario covers tools that predict the hosts for a targeted group of viruses, such as viruses in only a couple of families. The second scenario covers tools that predicted a specified set of hosts for a virus, such as deciding whether a virus can infect human.
We expect these modifications to highlight the focus of our work.

Response to Reviewer 2's comments
The authors present a new tool for annotation of RNA viruses to generate host reservoirs for a viral genome of a known viral order. The paper is generally well written and this new concept for viral annotations would be of interest to the wider scientific community.
Thank you for providing the summary. We sincerely appreciate your time and comments. Our point-to-point responses are shown below.

Main comments:
I would recommend a change in the tool name (you can keep if you wish). There are many viral host prediction tools, and the name VirHost implies a general host prediction

tool whereas it appears to be more specialized.
Thank you for this suggestion. To better abbreviate the function of our tool, we change the name to “RNAVirHost”, which further clarifies the scope of virus type and improves the conciseness. We supposed this name could be a better summary of our tool.

I would like a clearer definition of "natural-hosts" and "reservoir hosts", and what make a reservoir host annotation different to a host annotation. This should also be clearer in the abstract.
Thank you for this insightful comment. While viruses’ hosts are clearly defined as cellular organisms that the viruses can infect and replicate within their cells, there are various conflicting definitions of “reservoir”. Some studies highlighted that the infections in reservoirs are always nonpathogenic; some stressed that the reservoirs allow the persistence of the viruses; some suggested the uniqueness of the reservoir; others claimed that reservoirs could be a community involving various species [1,2,3]. Usually, the minimum set of host communities where the viruses can sustain can be accepted as “reservoirs”. For example, wild aquatic birds are considered reservoirs for avian influenza viruses [4]. However, it is hard to state who is not part of the “reservoirs”. In some cases, viruses can be sustained in a larger set of hosts. For instance, dogs and bats are individual sources of rabies viruses, which can be sustained in both communities solely [5].
By definition, the natural hosts comprise reservoir and secondary hosts. The secondary hosts are supposed to be infected in the spillover events, and the viruses show distinctive characteristics in secondary hosts, like higher pathogenicity and strong lethality [3,8]. Even though spillover events have shown their significant threat to human, they are relatively rare during the long co-evolutionary period with their reservoir hosts [6].
In the first draft, we followed Babayan [7] and defined the problem as a reservoir prediction problem. However, the definition of a reservoir is more stringent and complex than the definition a host. In this revision, we followed the usage of the “host” in the Virus-Host Database and NCBI GenBank. These widely used reference datasets use “hosts” rather than “reservoirs”.
Therefore, given that the concept of “reservoir” involves various aspects of viruses, including replication, transmission, maintenance, pathogenicity, and virulence, and has not yet received a common-acknowledged definition, we decided to shift the name from “reservoir” to “host” in this context, which is expected to define the objective better and enhance the clarity. This modification aligns our research goals with the two data sources' definitions of viruses’ hosts.
[1] Haydon, D. T., Cleaveland, S., Taylor, L. H., & Laurenson, M. K. (2002). Identifying reservoirs of infection: a conceptual and practical challenge. Emerging infectious diseases, 8(12), 1468-1473.
[2] Guth, S., Visher, E., Boots, M., & Brook, C. E. (2019). Host phylogenetic distance drives trends in virus virulence and transmissibility across the animal–human interface. Philosophical Transactions of the Royal Society B, 374(1782), 20190296.
[3] Brook, C. E., Rozins, C., Guth, S., & Boots, M. (2023). Reservoir host immunology and life history shape virulence evolution in zoonotic viruses. Plos Biology, 21(9), e3002268.
[4] Blagodatski, A., Trutneva, K., Glazova, O., Mityaeva, O., Shevkova, L., Kegeles, E., ... & Volchkov, P. (2021). Avian influenza in wild birds and poultry: dissemination pathways, monitoring methods, and virus ecology. Pathogens, 10(5), 630.
[5] Brunker, K., & Mollentze, N. (2018). Rabies virus. Trends in microbiology, 26(10), 886-887.
[6] Plowright, R. K., Parrish, C. R., McCallum, H., Hudson, P. J., Ko, A. I., Graham, A. L., & Lloyd-Smith, J. O. (2017). Pathways to zoonotic spillover. Nature Reviews Microbiology, 15(8), 502-510.
[7] Babayan, S. A., Orton, R. J., & Streicker, D. G. (2018). Predicting reservoir hosts and arthropod vectors from evolutionary signatures in RNA virus genomes. Science, 362(6414), 577-580.
[8] Mollentze, N., Streicker, D. G., Murcia, P. R., Hampson, K., & Biek, R. (2020). Virulence mismatches in index hosts shape the outcomes of cross-species transmission. Proceedings of the National Academy of Sciences, 117(46), 28859-28866.

The benchmarking datasets should be provided prior to publication. The authors also need to describe their approach for selecting genomes to include for the benchmarking

datasets for 'novel' viruses and for the recently identified viruses.
Thank you for your valuable feedback. Following this suggestion, we have expanded the “Data Collection” section to provide more details about the reference dataset, including its sources and filtering criteria.
In the experiments on “novel RNA viruses”, we generate the “novel RNA viruses” by leaving a specific genus of reference viruses as the test set. This genus is not part of the training data, thus ensuring a rigorous test of our method. Then, we trained the model without including the specific genus and assessed its performance on that genus to mimic the situation where a novel query, particularly with an unknown genus label, is used as input. The leave-one-genus-out evaluations are conducted on all virus genera (448 virus genera). As all the test sequences were generated from the reference dataset, we got the genus labels from the annotation files of Virus-Host DB and NCBI GenBank. The information can be found in section “Performance on novel RNA viruses”.
For the “recently identified viruses”, we collected 20 works that focused on identifying novel viruses on specific hosts and confirmed the viral infection by laboratory measures. While fifteen of them were published after 2023, we expanded the dataset by including five more works that were published before 2023 to cover different hosts as comprehensively as possible. We manually curated the dataset to ensure that the reference dataset does not include any new viral sequences presented in the 20 works. Besides, most of the works were designed on understudied hosts, for example, Hippocampus erectus (seahorse) as fishes, various fungi, Stellaria aquatica and Cnidium officinale as plant, and Apis mellifera (Western honey bee) as Invertebrate. More details can be accessed in section “Performance on recently identified viruses”.
Furthermore, for reproducibility and further analysis, we have deposited all data used in the study in GigaDB, making it readily accessible to the research community. Upon publication, the GigaDB repository will be accessible through the link provided in section “Data availability”.

The authors could calculate F1 scores to highlight optimizing the trade-off between sensitivity and precision.
Thank you for your comment. We have further evaluated the different prediction methods using the F1 score as an additional metric. Our tool still showed superior performance in most experiments. The new figures can be found in Supplementary Figure S1.
In the cross-validation experiments, our tool showed the highest F1 scores across various virus orders and host layers. In Layer 1 (hosts’ kingdom or phylum level), we achieved the highest F1 score of 88.41%, outperforming the second-best strategy, Bias_Blast_Dsp (a method from [1]), by 1.6%. In Layer 2 (hosts’ class or order level), we also achieved the highest F1 score of 80.59%, outperforming the second-best strategy, Bias_Blast (a method from [2]), by 2.25%. The higher F1 score of our tool indicates its robustness against the imbalanced host labels. The result is shown below:

Fig. S1 Host prediction performance (F1 score) of different feature sets in Layer 1 (A) and Layer 2 (B). Host prediction performance (F1 score) of different feature combinations in Layer 1 (C) and Layer 2 (D).
X-axis: Virus orders sorted by size (from largest to smallest). The performance comparison in Layer 2 considers the errors from both Layer 1 and Layer 2. Among the 18 orders, thirteen can be further classified in Layer 2. Therefore, we focused our performance evaluation solely on Layer 2 within these thirteen orders.
[1] Lee, B., Smith, D. K., & Guan, Y. (2021). Alignment free sequence comparison methods and reservoir host prediction. Bioinformatics, 37(19), 3337-3342.
[2] Babayan, S. A., Orton, R. J., & Streicker, D. G. (2018). Predicting reservoir hosts and arthropod vectors from evolutionary signatures in RNA virus genomes. Science, 362(6414), 577-580.

Minor comments:
-The concept of a reservoir instead of a host should be explained in the abstract.
Thank you for your comment. We added more discussion about hosts in Introduction and Abstract. More detailed information about this can be found in our response to the second major comment.

-Please check spelling of virus names. Flaviviridae is misspelled.
Thank for pointing this out. We have carefully checked the spelling of virus taxa.

-Code looks good, were you planning on releasing it as a bioconda or pypi package?
Thank you for your comment. Now, we have packaged and distributed the project in a more accessible way through Bioconda, PyPi, and GitHub.

-The color scale for fig3b is odd. Is there a better way to highlight the chordata bias for invertebrate?
Thank you for this valuable comment. We now modified Fig. 3(B) as shown below.

Fig. 3 (B) The confusion matrix of \tool's prediction in Layer 1. The values outside the brackets denote the percentage of the misclassified members, which were normalized to the sum of misclassified viruses of the corresponding groups (by row). The values in the brackets denote the percentage of the prediction labels, normalized to the corresponding groups' total number (by row). The viruses infecting bacteria are limited to specific virus orders, which exclusively infect bacteria. Hence, we do not visualize bacteria here.

Response to Reviewer 3’s comments
The authors present a machine learning-based tool named VirHost that can predict the reservoir hosts of RNA viruses. The motivation behind the development of the tool is clear and well-justified. The authors have thoroughly benchmarked the tool against existing tools such as DeepHoF based on different genomic features and machine learning architectures. The tool has also been tested on newly identified viruses whose hosts have been experimentally determined which shows the tool's potential. I appreciate the fact that the authors have identified and discussed the limitations of VirHost. Overall, I think this is a solid piece of work.
Thank you for providing the summary. We sincerely appreciate the time and effort you dedicated to reviewing our manuscript. Our point-to-point responses are shown below.

Comments for revisions in text
Metagenome assembly can be a challenging task, especially in recovering RNA viruses and genomes can be fragmented. In cases where the complete contiguous genomes are not recovered, especially for larger viruses, how will the length of the fragments (contigs) affect the prediction accuracy? The authors are welcome to discuss this aspect.
Thank you for this valuable comment. We have conducted an additional assessment on the fragmented viral sequences to evaluate the performance of the tools. Specifically, we followed the cross-validation setting and generated four groups of short contigs by cutting the test sequences with different length ratios, including 90%, 75%, 60%, 45%. We then evaluated the tools by their order-wise accuracy and F1 scores. Our results, presented in Fig. 4 and Fig. S6 as below, demonstrate superior performance compared to other strategies.
Among the simple feature sets, “sBias” and “Bias” consistently outperformed other alignment-free feature sets. Notably, even when the completeness of viral sequences was reduced to 45%, “sBias” maintained comparable accuracy compared to the alignment-based method, BLASTN. Among the feature combinations, our tool, combining “sBias” and BLASTN, demonstrated the highest accuracy and F1 scores in most cases.
The new figures were added to the main manuscript and Supplementary File.

Fig. 4 Host prediction performance (order-wise accuracy) of different feature sets in Layer 1 (A, B) and Layer 2 (C, D). X-axis: the completeness of viral sequences. Y-axis: The order-wise accuracy of the corresponding feature set.

Fig. S6 Host prediction performance (order-wise F1 score) of different feature sets in Layer 1 (A, B) and Layer 2 (C, D). X-axis: the completeness of viral sequences. Y-axis: The order-wise F1 score of the corresponding feature set.

In Figure 2, do A and B have the same legend? It would be clearer to include separate legends for A and B.
Thank you for this comment. They do have the same legend. Following your suggestion, we added separate legends for A and B.

Powered by Editorial Manager® and ProduXion Manager® from Aries Systems Corporation

	Minor comments<br>I noticed a couple of typos.<br>- Page 3: Although numerous RNA viruses "have" been found, the current...<br>- Page 4: To "represent" the "genomic features", we translate the query...<br>- Page 5: Considering that most RNA "viruses" are "shorter" than 50 kbp and...<br>- Page 5: A detailed description of the benchmarked "features" can be found in the...<br>- Page 7: We investigated host distribution of each virus family and visualized the "error rates" of virus...<br>- Page 7: ... implying that these wrong "predictions" are mainly from viruses with multiple hosts across phylum. - Page 8: The statistics "are" listed in Table 2.<br>- Page 8: ... which is a member of Alphaparti-tivirus, a genus that "infects" both "plants" and fungi.<br>- Page 9: With the increasing "availability of" viruses' host annotations...<br>I recommend the authors to proofread the final manuscript thoroughly.<br>Thank you for catching these. We have conducted additional rounds of proofreading and carefully revised and polished the manuscript with the assistance of Grammlary.<br><br>Comments for revisions in GitHub<br>Please add a .gitignore file so you can ignore the files and directories which are unnecessary to the git project (e.g. .idea).<br>Thank you for pointing this out. We have removed the unnecessary files.<br><br>With the current version of the code, I had to always either run virhost.py from the VirHost directory or give the full path to virhost.py. It would be more convenient if VirHost could be installed in the current Python environment so it can be called from anywhere. It might be worth looking into how to package and distribute Python projects.<br>It would be nice to put VirHost on Bioconda and PyPI so the users can easily install it.<br>Thank you for your comment. Now, we have packaged and distributed the project in a more accessible way through Bioconda, PyPi, and GitHub.<br><br>The authors mention "However, we recommend users to generate their own taxonomic classification result, which is expected to improve the prediction confidence.". It would be good to have more details on how to generate custom taxonomic classification results with recommended tools to use so that even someone who is not familiar with the available bioinformatics solutions can easily get started.<br>Thank you for your comment. The taxonomic classification file is necessary for the input requirement. We provide easy access for the users to get the order-level label through BLASTN. We aligned the query against the reference dataset and used the best hit strategy to assign the order-level virus label. While the order-level classification is not a hard task, this method can provide sufficiently accurate prediction. Besides the default BLASTN program, we recommend two classification programs that can be applied to RNA viruses for users: PhaGCN2, CAT.<br>The resulting file includes two columns: the sequence ID and the viruses' order labels (including 30 orders), as shown below. Now, we provide more detailed guidance in GitHub for users' convenience.
**Additional Information:**	
**Question**	**Response**
Are you submitting this manuscript to a special series or article collection?	No
**Experimental design and statistics**<br><br>Full details of the experimental design and statistical methods used should be given in the Methods section, as detailed in our Minimum Standards Reporting Checklist. Information essential to interpreting the data presented should be made available	Yes

Powered by Editorial Manager® and ProduXion Manager® from Aries Systems Corporation

in the figure legends.<br><br>Have you included all the information requested in your manuscript?	
**Resources**<br><br>A description of all resources used, including antibodies, cell lines, animals and software tools, with enough information to allow them to be uniquely identified, should be included in the Methods section. Authors are strongly encouraged to cite Research Resource Identifiers (RRIDs) for antibodies, model organisms and tools, where possible.<br><br>Have you included the information requested as detailed in our Minimum Standards Reporting Checklist?	Yes
**Availability of data and materials**<br><br>All datasets and code on which the conclusions of the paper rely must be either included in your submission or deposited in publicly available repositories (where available and ethically appropriate), referencing such data using a unique identifier in the references and in the “Availability of Data and Materials” section of your manuscript.<br><br>Have you have met the above requirement as detailed in our Minimum Standards Reporting Checklist?	No
If not, please give reasons for any omissions below.<br><br>as follow-up to "**Availability of data and materials**<br><br>All datasets and code on which the	We will submit the analytic code and reference data to GigaDB when it is allocated to us.

conclusions of the paper rely must be either included in your submission or deposited in publicly available repositories (where available and ethically appropriate), referencing such data using a unique identifier in the references and in the "Availability of Data and Materials" section of your manuscript.<br><br>Have you have met the above requirement as detailed in our Minimum Standards Reporting Checklist?<br><br>"	

Powered by Editorial Manager® and ProduXion Manager® from Aries Systems Corporation

Click here to access/download;LaTeX - Main Document (TeX file);main.tex

```
This is pdfTeX, Version 3.141592653-2.6-1.40.25 (TeX Live 2023)
(preloaded format=pdflatex 2024.3.8)  22 JUL 2024 09:25
entering extended mode
 restricted \write18 enabled.
 %&-line parsing enabled.
**main.tex
(./main.tex
LaTeX2e <2023-11-01> patch level 1
L3 programming layer <2024-02-20>
(./oup-contemporary.cls
Document Class: oup-contemporary 2023/06/12, v1.2
(c:/texlive/2023/texmf-dist/tex/latex/base/article.cls
Document Class: article 2023/05/17 v1.4n Standard LaTeX document class
(c:/texlive/2023/texmf-dist/tex/latex/base/size10.clo
File: size10.clo 2023/05/17 v1.4n Standard LaTeX file (size option)
)
\c@part=\count188
\c@section=\count189
\c@subsection=\count190
\c@subsubsection=\count191
\c@paragraph=\count192
\c@subparagraph=\count193
\c@figure=\count194
\c@table=\count195
\abovecaptionskip=\skip48
\belowcaptionskip=\skip49
\bibindent=\dimen140
) (c:/texlive/2023/texmf-dist/tex/latex/base/inputenc.sty
Package: inputenc 2021/02/14 v1.3d Input encoding file
\inpenc@prehook=\toks17
\inpenc@posthook=\toks18
) (c:/texlive/2023/texmf-dist/tex/latex/base/fontenc.sty
Package: fontenc 2021/04/29 v2.0v Standard LaTeX package
) (c:/texlive/2023/texmf-dist/tex/generic/iftex/ifpdf.sty
Package: ifpdf 2019/10/25 v3.4 ifpdf legacy package. Use iftex instead.
(c:/texlive/2023/texmf-dist/tex/generic/iftex/iftex.sty
Package: iftex 2022/02/03 v1.0f TeX engine tests
)) (c:/texlive/2023/texmf-dist/tex/latex/microtype/microtype.sty
Package: microtype 2023/03/13 v3.1a Micro-typographical refinements (RS)
(c:/texlive/2023/texmf-dist/tex/latex/graphics/keyval.sty
Package: keyval 2022/05/29 v1.15 key=value parser (DPC)
\KV@toks@=\toks19
) (c:/texlive/2023/texmf-dist/tex/latex/etoolbox/etoolbox.sty
Package: etoolbox 2020/10/05 v2.5k e-TeX tools for LaTeX (JAW)
\etb@tempcnta=\count196
)
\MT@toks=\toks20
\MT@tempbox=\box51
\MT@count=\count197
LaTeX Info: Redefining \noprotrusionifhmode on input line 1059.
LaTeX Info: Redefining \leftprotrusion on input line 1060.
\MT@prot@toks=\toks21
LaTeX Info: Redefining \rightprotrusion on input line 1078.
LaTeX Info: Redefining \textls on input line 1368.
```

```
\MT@outer@kern=\dimen141
LaTeX Info: Redefining \textmicrotypecontext on input line 1988.
\MT@listname@count=\count198
(c:/texlive/2023/texmf-dist/tex/latex/microtype/microtype-pdftex.def
File: microtype-pdftex.def 2023/03/13 v3.1a Definitions specific to
pdftex (RS)

LaTeX Info: Redefining \lsstyle on input line 902.
LaTeX Info: Redefining \lslig on input line 902.
\MT@outer@space=\skip50
)
Package microtype Info: Loading configuration file microtype.cfg.
(c:/texlive/2023/texmf-dist/tex/latex/microtype/microtype.cfg
File: microtype.cfg 2023/03/13 v3.1a microtype main configuration file
(RS)
)) (c:/texlive/2023/texmf-dist/tex/latex/euler/euler.sty
Package: euler 1995/03/05 v2.5
Package: `euler' v2.5 <1995/03/05> (FJ and FMi)
LaTeX Font Info:    Redeclaring symbol font `letters' on input line 35.
LaTeX Font Info:    Encoding `OML' has changed to `U' for symbol font
(Font)              `letters' in the math version `normal' on input line
35.
LaTeX Font Info:    Overwriting symbol font `letters' in version `normal'
(Font)                  OML/cmm/m/it --> U/eur/m/n on input line 35.
LaTeX Font Info:    Encoding `OML' has changed to `U' for symbol font
(Font)              `letters' in the math version `bold' on input line
35.
LaTeX Font Info:    Overwriting symbol font `letters' in version `bold'
(Font)                  OML/cmm/b/it --> U/eur/m/n on input line 35.
LaTeX Font Info:    Overwriting symbol font `letters' in version `bold'
(Font)                  U/eur/m/n --> U/eur/b/n on input line 36.
LaTeX Font Info:    Redeclaring math symbol \Gamma on input line 47.
LaTeX Font Info:    Redeclaring math symbol \Delta on input line 48.
LaTeX Font Info:    Redeclaring math symbol \Theta on input line 49.
LaTeX Font Info:    Redeclaring math symbol \Lambda on input line 50.
LaTeX Font Info:    Redeclaring math symbol \Xi on input line 51.
LaTeX Font Info:    Redeclaring math symbol \Pi on input line 52.
LaTeX Font Info:    Redeclaring math symbol \Sigma on input line 53.
LaTeX Font Info:    Redeclaring math symbol \Upsilon on input line 54.
LaTeX Font Info:    Redeclaring math symbol \Phi on input line 55.
LaTeX Font Info:    Redeclaring math symbol \Psi on input line 56.
LaTeX Font Info:    Redeclaring math symbol \Omega on input line 57.
\symEulerFraktur=\mathgroup4
LaTeX Font Info:    Overwriting symbol font `EulerFraktur' in version
`bold'
(Font)                  U/euf/m/n --> U/euf/b/n on input line 63.
LaTeX Info: Redefining \oldstylenums on input line 85.
\symEulerScript=\mathgroup5
LaTeX Font Info:    Overwriting symbol font `EulerScript' in version
`bold'
(Font)                  U/eus/m/n --> U/eus/b/n on input line 93.
LaTeX Font Info:    Redeclaring math symbol \aleph on input line 97.
LaTeX Font Info:    Redeclaring math symbol \Re on input line 98.
LaTeX Font Info:    Redeclaring math symbol \Im on input line 99.
```

```
LaTeX Font Info:    Redeclaring math delimiter \vert on input line 101.
LaTeX Font Info:    Redeclaring math delimiter \backslash on input line
103.
LaTeX Font Info:    Redeclaring math symbol \neg on input line 106.
LaTeX Font Info:    Redeclaring math symbol \wedge on input line 108.
LaTeX Font Info:    Redeclaring math symbol \vee on input line 110.
LaTeX Font Info:    Redeclaring math symbol \setminus on input line 112.
LaTeX Font Info:    Redeclaring math symbol \sim on input line 113.
LaTeX Font Info:    Redeclaring math symbol \mid on input line 114.
LaTeX Font Info:    Redeclaring math delimiter \arrowvert on input line
116.
LaTeX Font Info:    Redeclaring math symbol \mathsection on input line
117.
\symEulerExtension=\mathgroup6
LaTeX Font Info:    Redeclaring math symbol \coprod on input line 125.
LaTeX Font Info:    Redeclaring math symbol \prod on input line 125.
LaTeX Font Info:    Redeclaring math symbol \sum on input line 125.
LaTeX Font Info:    Redeclaring math symbol \intop on input line 130.
LaTeX Font Info:    Redeclaring math symbol \ointop on input line 131.
LaTeX Font Info:    Redeclaring math symbol \braceld on input line 132.
LaTeX Font Info:    Redeclaring math symbol \bracerd on input line 133.
LaTeX Font Info:    Redeclaring math symbol \bracelu on input line 134.
LaTeX Font Info:    Redeclaring math symbol \braceru on input line 135.
LaTeX Font Info:    Redeclaring math symbol \infty on input line 136.
LaTeX Font Info:    Redeclaring math symbol \nearrow on input line 153.
LaTeX Font Info:    Redeclaring math symbol \searrow on input line 154.
LaTeX Font Info:    Redeclaring math symbol \nwarrow on input line 155.
LaTeX Font Info:    Redeclaring math symbol \swarrow on input line 156.
LaTeX Font Info:    Redeclaring math symbol \Leftrightarrow on input line
157.
LaTeX Font Info:    Redeclaring math symbol \Leftarrow on input line 158.
LaTeX Font Info:    Redeclaring math symbol \Rightarrow on input line
159.
LaTeX Font Info:    Redeclaring math symbol \leftrightarrow on input line
160.
LaTeX Font Info:    Redeclaring math symbol \leftarrow on input line 161.
LaTeX Font Info:    Redeclaring math symbol \rightarrow on input line
163.
LaTeX Font Info:    Redeclaring math delimiter \uparrow on input line
166.
LaTeX Font Info:    Redeclaring math delimiter \downarrow on input line
168.
LaTeX Font Info:    Redeclaring math delimiter \updownarrow on input line
170.
LaTeX Font Info:    Redeclaring math delimiter \Uparrow on input line
172.
LaTeX Font Info:    Redeclaring math delimiter \Downarrow on input line
174.
LaTeX Font Info:    Redeclaring math delimiter \Updownarrow on input line
176.
LaTeX Font Info:    Redeclaring math symbol \leftharpoonup on input line
177.
LaTeX Font Info:    Redeclaring math symbol \leftharpoondown on input
line 178.
```

```
LaTeX Font Info:    Redeclaring math symbol \rightharpoonup on input line
179.
LaTeX Font Info:    Redeclaring math symbol \rightharpoondown on input
line 180
.
LaTeX Font Info:    Redeclaring math delimiter \lbrace on input line 182.
LaTeX Font Info:    Redeclaring math delimiter \rbrace on input line 184.
\symcmmigroup=\mathgroup7
LaTeX Font Info:    Overwriting symbol font `cmmigroup' in version `bold'
(Font)                  OML/cmm/m/it --> OML/cmm/b/it on input line 200.
LaTeX Font Info:    Redeclaring math accent \vec on input line 201.
LaTeX Font Info:    Redeclaring math symbol \triangleleft on input line
202.
LaTeX Font Info:    Redeclaring math symbol \triangleright on input line
203.
LaTeX Font Info:    Redeclaring math symbol \star on input line 204.
LaTeX Font Info:    Redeclaring math symbol \lhook on input line 205.
LaTeX Font Info:    Redeclaring math symbol \rhook on input line 206.
LaTeX Font Info:    Redeclaring math symbol \flat on input line 207.
LaTeX Font Info:    Redeclaring math symbol \natural on input line 208.
LaTeX Font Info:    Redeclaring math symbol \sharp on input line 209.
LaTeX Font Info:    Redeclaring math symbol \smile on input line 210.
LaTeX Font Info:    Redeclaring math symbol \frown on input line 211.
LaTeX Font Info:    Redeclaring math accent \grave on input line 245.
LaTeX Font Info:    Redeclaring math accent \acute on input line 246.
LaTeX Font Info:    Redeclaring math accent \tilde on input line 247.
LaTeX Font Info:    Redeclaring math accent \ddot on input line 248.
LaTeX Font Info:    Redeclaring math accent \check on input line 249.
LaTeX Font Info:    Redeclaring math accent \breve on input line 250.
LaTeX Font Info:    Redeclaring math accent \bar on input line 251.
LaTeX Font Info:    Redeclaring math accent \dot on input line 252.
LaTeX Font Info:    Redeclaring math accent \hat on input line 254.
) (c:/texlive/2023/texmf-dist/tex/latex/merriweather/merriweather.sty
Package: merriweather 2022/09/20 (Bob Tennent) Supports
Merriweather(Sans) font
s for all LaTeX engines.
(c:/texlive/2023/texmf-dist/tex/generic/iftex/ifxetex.sty
Package: ifxetex 2019/10/25 v0.7 ifxetex legacy package. Use iftex
instead.
) (c:/texlive/2023/texmf-dist/tex/generic/iftex/ifluatex.sty
Package: ifluatex 2019/10/25 v1.5 ifluatex legacy package. Use iftex
instead.
) (c:/texlive/2023/texmf-dist/tex/latex/base/textcomp.sty
Package: textcomp 2020/02/02 v2.0n Standard LaTeX package
) (c:/texlive/2023/texmf-dist/tex/latex/xkeyval/xkeyval.sty
Package: xkeyval 2022/06/16 v2.9 package option processing (HA)
(c:/texlive/2023/texmf-dist/tex/generic/xkeyval/xkeyval.tex
(c:/texlive/2023/te
xmf-dist/tex/generic/xkeyval/xkvutils.tex
\XKV@toks=\toks22
\XKV@tempa@toks=\toks23
)
\XKV@depth=\count199
```

```
File: xkeyval.tex 2014/12/03 v2.7a key=value parser (HA)
)) (c:/texlive/2023/texmf-dist/tex/latex/base/fontenc.sty
Package: fontenc 2021/04/29 v2.0v Standard LaTeX package
) (c:/texlive/2023/texmf-dist/tex/latex/fontaxes/fontaxes.sty
Package: fontaxes 2020/07/21 v1.0e Font selection axes
LaTeX Info: Redefining \upshape on input line 29.
LaTeX Info: Redefining \itshape on input line 31.
LaTeX Info: Redefining \slshape on input line 33.
LaTeX Info: Redefining \swshape on input line 35.
LaTeX Info: Redefining \scshape on input line 37.
LaTeX Info: Redefining \sscshape on input line 39.
LaTeX Info: Redefining \ulcshape on input line 41.
LaTeX Info: Redefining \textsw on input line 47.
LaTeX Info: Redefining \textssc on input line 48.
LaTeX Info: Redefining \textulc on input line 49.
)) (c:/texlive/2023/texmf-dist/tex/latex/mathastext/mathastext.sty
Package: mathastext 2023/12/29 v1.3zb Use the text font in math mode
(JFB)

Package mathastext Info: Starting the math mode configuration.
\mst@exists@muskip=\muskip16
\mst@forall@muskip=\muskip17
\mst@prime@muskip=\muskip18
\mst@do@nonletters=\toks24
\mst@do@easynonletters=\toks25
\mst@do@az=\toks26
\mst@do@AZ=\toks27
\symmtoperatorfont=\mathgroup8
\symmtletterfont=\mathgroup9
(   mathastext:   ) ! and ?
(   mathastext:   ) punctuation: , . : ; and \colon
LaTeX Info: Redefining \relbar on input line 894.
LaTeX Info: Redefining \rightarrowfill on input line 897.
LaTeX Info: Redefining \leftarrowfill on input line 902.
(   mathastext:   ) + and =
LaTeX Info: Redefining \Relbar on input line 993.
(   mathastext:   ) adding = ; and + to \nfss@catcodes
(   mathastext:   ) parentheses ( ) [ ] and slash /
(   mathastext:   ) alldelims: < > \backslash \setminus | \vert \mid \{
\}
LaTeX Font Info:    Redeclaring math delimiter \backslash on input line
1039.
LaTeX Font Info:    Redeclaring math symbol \setminus on input line 1051.
LaTeX Info: Redefining \models on input line 1060.
(   mathastext:   ) \# \mathdollar \% \&
(   mathastext:   ) \imath and \jmath
LaTeX Font Info:    Overwriting math alphabet `\Mathnormalbold' in
version `nor
mal'
(Font)                  T1/Merriwthr-OsF/b/it --> T1/Merriwthr-OsF/b/it
on inpu
t line 2516.
LaTeX Font Info:    Overwriting math alphabet `\Mathnormalbold' in
version `bol
```

```
d'
(Font)                  T1/Merriwthr-OsF/b/it --> T1/Merriwthr-OsF/b/it
on inpu
t line 2516.
LaTeX Font Info:    Overwriting symbol font `mtletterfont' in version
`normal'
(Font)                  T1/Merriwthr-OsF/m/it --> T1/Merriwthr-OsF/m/it
on inpu
t line 2516.
LaTeX Font Info:    Overwriting symbol font `mtletterfont' in version
`bold'
(Font)                  T1/Merriwthr-OsF/m/it --> T1/Merriwthr-OsF/b/it
on inpu
t line 2516.
LaTeX Font Info:    Overwriting symbol font `mtoperatorfont' in version
`normal
'
(Font)                  T1/Merriwthr-OsF/m/n --> T1/Merriwthr-OsF/m/n on
input
line 2516.
LaTeX Font Info:    Overwriting symbol font `mtoperatorfont' in version
`bold'
(Font)                  T1/Merriwthr-OsF/m/n --> T1/Merriwthr-OsF/b/n on
input
line 2516.
LaTeX Font Info:    Overwriting math alphabet `\Mathbf' in version
`normal'
(Font)                  T1/Merriwthr-OsF/b/n --> T1/Merriwthr-OsF/b/n on
input
line 2516.
LaTeX Font Info:    Overwriting math alphabet `\Mathbf' in version `bold'
(Font)                  T1/Merriwthr-OsF/b/n --> T1/Merriwthr-OsF/b/n on
input
line 2516.
LaTeX Font Info:    Overwriting math alphabet `\Mathit' in version
`normal'
(Font)                  T1/Merriwthr-OsF/m/it --> T1/Merriwthr-OsF/m/it
on inpu
t line 2516.
LaTeX Font Info:    Overwriting math alphabet `\Mathit' in version `bold'
(Font)                  T1/Merriwthr-OsF/m/it --> T1/Merriwthr-OsF/b/it
on inpu
t line 2516.
LaTeX Font Info:    Overwriting math alphabet `\Mathsf' in version
`normal'
(Font)                  T1/MerriwthrSans-OsF/m/n --> T1/MerriwthrSans-
OsF/m/n o
n input line 2516.
LaTeX Font Info:    Overwriting math alphabet `\Mathsf' in version `bold'
(Font)                  T1/MerriwthrSans-OsF/m/n --> T1/MerriwthrSans-
OsF/b/n o
n input line 2516.
LaTeX Font Info:    Overwriting math alphabet `\Mathtt' in version
`normal'
```

```
(Font)                  T1/lmtt/m/n --> T1/lmtt/m/n on input line 2516.
LaTeX Font Info:    Overwriting math alphabet `\Mathtt' in version `bold'
(Font)                  T1/lmtt/m/n --> T1/lmtt/b/n on input line 2516.
(   mathastext:   ) Latin letters in the `normal', resp. `bold',
(   mathastext:   ) math versions are now set up to use the fonts
(   mathastext:   ) T1/Merriwthr-OsF/m/it, resp. T1/Merriwthr-OsF/b/it.
(   mathastext:   ) Other characters (digits, ...) and \log-like names
will be
(   mathastext:   ) typeset with the n shape.
(   mathastext:   ) \hbar
(   mathastext:   ) minus as endash
(   mathastext:   ) The italic option is in effect.
(   mathastext:   ) \HUGE has been (re)-defined.
(   mathastext:   ) mathastext has declared larger sizes for subscripts.
(   mathastext:   ) To keep LaTeX defaults, use option
`defaultmathsizes'.

Package mathastext Info: Loading is complete.  You can now use
\Mathastext to
(mathastext)             modify the normal and bold math versions.  Use
it
(mathastext)             with optional argument or use \MTDeclareVersion
to
(mathastext)             declare additional math versions.
) (c:/texlive/2023/texmf-dist/tex/latex/relsize/relsize.sty
Package: relsize 2013/03/29 ver 4.1
) (c:/texlive/2023/texmf-dist/tex/latex/ragged2e/ragged2e.sty
Package: ragged2e 2023/06/22 v3.6 ragged2e Package
\CenteringLeftskip=\skip51
\RaggedLeftLeftskip=\skip52
\RaggedRightLeftskip=\skip53
\CenteringRightskip=\skip54
\RaggedLeftRightskip=\skip55
\RaggedRightRightskip=\skip56
\CenteringParfillskip=\skip57
\RaggedLeftParfillskip=\skip58
\RaggedRightParfillskip=\skip59
\JustifyingParfillskip=\skip60
\CenteringParindent=\skip61
\RaggedLeftParindent=\skip62
\RaggedRightParindent=\skip63
\JustifyingParindent=\skip64
) (c:/texlive/2023/texmf-dist/tex/latex/xcolor/xcolor.sty
Package: xcolor 2023/11/15 v3.01 LaTeX color extensions (UK)
(c:/texlive/2023/texmf-dist/tex/latex/graphics-cfg/color.cfg
File: color.cfg 2016/01/02 v1.6 sample color configuration
)
Package xcolor Info: Driver file: pdftex.def on input line 274.
(c:/texlive/2023/texmf-dist/tex/latex/graphics-def/pdftex.def
File: pdftex.def 2022/09/22 v1.2b Graphics/color driver for pdftex
) (c:/texlive/2023/texmf-dist/tex/latex/graphics/mathcolor.ltx)
Package xcolor Info: Model `cmy' substituted by `cmy0' on input line
1350.
Package xcolor Info: Model `hsb' substituted by `rgb' on input line 1354.
```

```
Package xcolor Info: Model `RGB' extended on input line 1366.
Package xcolor Info: Model `HTML' substituted by `rgb' on input line
1368.
Package xcolor Info: Model `Hsb' substituted by `hsb' on input line 1369.
Package xcolor Info: Model `tHsb' substituted by `hsb' on input line
1370.
Package xcolor Info: Model `HSB' substituted by `hsb' on input line 1371.
Package xcolor Info: Model `Gray' substituted by `gray' on input line
1372.
Package xcolor Info: Model `wave' substituted by `hsb' on input line
1373.
) (c:/texlive/2023/texmf-dist/tex/latex/colortbl/colortbl.sty
Package: colortbl 2024/02/20 v1.0g Color table columns (DPC)
(c:/texlive/2023/texmf-dist/tex/latex/tools/array.sty
Package: array 2023/10/16 v2.5g Tabular extension package (FMi)
\col@sep=\dimen142
\ar@mcellbox=\box52
\extrarowheight=\dimen143
\NC@list=\toks28
\extratabsurround=\skip65
\backup@length=\skip66
\ar@cellbox=\box53
)
\everycr=\toks29
\minrowclearance=\skip67
\rownum=\count266
) (c:/texlive/2023/texmf-dist/tex/latex/graphics/graphicx.sty
Package: graphicx 2021/09/16 v1.2d Enhanced LaTeX Graphics (DPC,SPQR)
(c:/texlive/2023/texmf-dist/tex/latex/graphics/graphics.sty
Package: graphics 2022/03/10 v1.4e Standard LaTeX Graphics (DPC,SPQR)
(c:/texlive/2023/texmf-dist/tex/latex/graphics/trig.sty
Package: trig 2021/08/11 v1.11 sin cos tan (DPC)
) (c:/texlive/2023/texmf-dist/tex/latex/graphics-cfg/graphics.cfg
File: graphics.cfg 2016/06/04 v1.11 sample graphics configuration
)
Package graphics Info: Driver file: pdftex.def on input line 107.
)
\Gin@req@height=\dimen144
\Gin@req@width=\dimen145
) (c:/texlive/2023/texmf-dist/tex/latex/xpatch/xpatch.sty
(c:/texlive/2023/texm
f-dist/tex/latex/l3kernel/expl3.sty
Package: expl3 2024-02-20 L3 programming layer (loader)
(c:/texlive/2023/texmf-dist/tex/latex/l3backend/l3backend-pdftex.def
File: l3backend-pdftex.def 2024-02-20 L3 backend support: PDF output
(pdfTeX)
\l__color_backend_stack_int=\count267
\l__pdf_internal_box=\box54
))
Package: xpatch 2020/03/25 v0.3a Extending etoolbox patching commands
(c:/texlive/2023/texmf-dist/tex/latex/l3packages/xparse/xparse.sty
Package: xparse 2024-02-18 L3 Experimental document command parser
)) (c:/texlive/2023/texmf-dist/tex/latex/environ/environ.sty
Package: environ 2014/05/04 v0.3 A new way to define environments
```

```
(c:/texlive/2023/texmf-dist/tex/latex/trimspaces/trimspaces.sty
Package: trimspaces 2009/09/17 v1.1 Trim spaces around a token list
)
\@envbody=\toks30
) (c:/texlive/2023/texmf-dist/tex/latex/lastpage/lastpage.sty
Package: lastpage 2023/10/14 v2.0e lastpage: 2.09 or 2e? (HMM)
(c:/texlive/2023/texmf-dist/tex/latex/lastpage/lastpage2e.sty
Package: lastpage2e 2023/10/14 v2.0e Decide which 2e lastpage version to
use (H
MM)
(c:/texlive/2023/texmf-dist/tex/latex/lastpage/lastpagemodern.sty
Package: lastpagemodern 2023-10-14 v2.0e Refers to last page's name (HMM;
JPG)
\c@lastpagecount=\count268
)
)) (c:/texlive/2023/texmf-dist/tex/latex/graphics/rotating.sty
Package: rotating 2016/08/11 v2.16d rotated objects in LaTeX
(c:/texlive/2023/texmf-dist/tex/latex/base/ifthen.sty
Package: ifthen 2022/04/13 v1.1d Standard LaTeX ifthen package (DPC)
)
\c@r@tfl@t=\count269
\rotFPtop=\skip68
\rotFPbot=\skip69
\rot@float@box=\box55
\rot@mess@toks=\toks31
) (c:/texlive/2023/texmf-dist/tex/latex/graphics/lscape.sty
Package: lscape 2020/05/28 v3.02 Landscape Pages (DPC)
) (c:/texlive/2023/texmf-dist/tex/latex/tools/afterpage.sty
Package: afterpage 2023/07/04 v1.08 After-Page Package (DPC)
\AP@output=\toks32
\AP@partial=\box56
\AP@footins=\box57
) (c:/texlive/2023/texmf-dist/tex/latex/textpos/textpos.sty
Package: textpos 2022/07/23 v1.10.1
Package textpos Info: choosing support for LaTeX3 on input line 60.
\TP@textbox=\box58
\TP@holdbox=\box59
\TPHorizModule=\dimen146
\TPVertModule=\dimen147
\TP@margin=\dimen148
\TP@absmargin=\dimen149
Grid set 16 x 16 = 37.34424pt x 52.81541pt
\TPboxrulesize=\dimen150
\TP@ox=\dimen151
\TP@oy=\dimen152
\TP@tbargs=\toks33
TextBlockOrigin set to 0pt x 0pt
) (c:/texlive/2023/texmf-dist/tex/latex/url/url.sty
\Urlmuskip=\muskip19
Package: url 2013/09/16  ver 3.4  Verb mode for urls, etc.
) (c:/texlive/2023/texmf-dist/tex/latex/newfloat/newfloat.sty
Package: newfloat 2023/10/01 v1.2 Defining new floating environments (AR)
Package newfloat Info: `rotating' package detected.
) (c:/texlive/2023/texmf-dist/tex/latex/mdframed/mdframed.sty
```

```
Package: mdframed 2013/07/01 1.9b: mdframed
(c:/texlive/2023/texmf-dist/tex/latex/kvoptions/kvoptions.sty
Package: kvoptions 2022-06-15 v3.15 Key value format for package options
(HO)
(c:/texlive/2023/texmf-dist/tex/generic/ltxcmds/ltxcmds.sty
Package: ltxcmds 2023-12-04 v1.26 LaTeX kernel commands for general use
(HO)
) (c:/texlive/2023/texmf-dist/tex/latex/kvsetkeys/kvsetkeys.sty
Package: kvsetkeys 2022-10-05 v1.19 Key value parser (HO)
)) (c:/texlive/2023/texmf-dist/tex/latex/zref/zref-abspage.sty
Package: zref-abspage 2023-09-14 v2.35 Module abspage for zref (HO)
(c:/texlive/2023/texmf-dist/tex/latex/zref/zref-base.sty
Package: zref-base 2023-09-14 v2.35 Module base for zref (HO)
(c:/texlive/2023/texmf-dist/tex/generic/infwarerr/infwarerr.sty
Package: infwarerr 2019/12/03 v1.5 Providing info/warning/error messages
(HO)
) (c:/texlive/2023/texmf-dist/tex/generic/kvdefinekeys/kvdefinekeys.sty
Package: kvdefinekeys 2019-12-19 v1.6 Define keys (HO)
) (c:/texlive/2023/texmf-dist/tex/generic/pdftexcmds/pdftexcmds.sty
Package: pdftexcmds 2020-06-27 v0.33 Utility functions of pdfTeX for
LuaTeX (HO
)
Package pdftexcmds Info: \pdf@primitive is available.
Package pdftexcmds Info: \pdf@ifprimitive is available.
Package pdftexcmds Info: \pdfdraftmode found.
) (c:/texlive/2023/texmf-dist/tex/generic/etexcmds/etexcmds.sty
Package: etexcmds 2019/12/15 v1.7 Avoid name clashes with e-TeX commands
(HO)
) (c:/texlive/2023/texmf-dist/tex/latex/auxhook/auxhook.sty
Package: auxhook 2019-12-17 v1.6 Hooks for auxiliary files (HO)
)
Package zref Info: New property list: main on input line 767.
Package zref Info: New property: default on input line 768.
Package zref Info: New property: page on input line 769.
)
\c@abspage=\count270
Package zref Info: New property: abspage on input line 67.
) (c:/texlive/2023/texmf-dist/tex/latex/needspace/needspace.sty
Package: needspace 2010/09/12 v1.3d reserve vertical space
)
\mdf@templength=\skip70
\c@mdf@globalstyle@cnt=\count271
\mdf@skipabove@length=\skip71
\mdf@skipbelow@length=\skip72
\mdf@leftmargin@length=\skip73
\mdf@rightmargin@length=\skip74
\mdf@innerleftmargin@length=\skip75
\mdf@innerrightmargin@length=\skip76
\mdf@innertopmargin@length=\skip77
\mdf@innerbottommargin@length=\skip78
\mdf@splittopskip@length=\skip79
\mdf@splitbottomskip@length=\skip80
\mdf@outermargin@length=\skip81
\mdf@innermargin@length=\skip82
```

```
\mdf@linewidth@length=\skip83
\mdf@innerlinewidth@length=\skip84
\mdf@middlelinewidth@length=\skip85
\mdf@outerlinewidth@length=\skip86
\mdf@roundcorner@length=\skip87
\mdf@footenotedistance@length=\skip88
\mdf@userdefinedwidth@length=\skip89
\mdf@needspace@length=\skip90
\mdf@frametitleaboveskip@length=\skip91
\mdf@frametitlebelowskip@length=\skip92
\mdf@frametitlerulewidth@length=\skip93
\mdf@frametitleleftmargin@length=\skip94
\mdf@frametitlerightmargin@length=\skip95
\mdf@shadowsize@length=\skip96
\mdf@extratopheight@length=\skip97
\mdf@subtitleabovelinewidth@length=\skip98
\mdf@subtitlebelowlinewidth@length=\skip99
\mdf@subtitleaboveskip@length=\skip100
\mdf@subtitlebelowskip@length=\skip101
\mdf@subtitleinneraboveskip@length=\skip102
\mdf@subtitleinnerbelowskip@length=\skip103
\mdf@subsubtitleabovelinewidth@length=\skip104
\mdf@subsubtitlebelowlinewidth@length=\skip105
\mdf@subsubtitleaboveskip@length=\skip106
\mdf@subsubtitlebelowskip@length=\skip107
\mdf@subsubtitleinneraboveskip@length=\skip108
\mdf@subsubtitleinnerbelowskip@length=\skip109
(c:/texlive/2023/texmf-dist/tex/latex/mdframed/md-frame-0.mdf
File: md-frame-0.mdf 2013/07/01\ 1.9b: md-frame-0
)
\mdf@frametitlebox=\box60
\mdf@footnotebox=\box61
\mdf@splitbox@one=\box62
\mdf@splitbox@two=\box63
\mdf@splitbox@save=\box64
\mdfsplitboxwidth=\skip110
\mdfsplitboxtotalwidth=\skip111
\mdfsplitboxheight=\skip112
\mdfsplitboxdepth=\skip113
\mdfsplitboxtotalheight=\skip114
\mdfframetitleboxwidth=\skip115
\mdfframetitleboxtotalwidth=\skip116
\mdfframetitleboxheight=\skip117
\mdfframetitleboxdepth=\skip118
\mdfframetitleboxtotalheight=\skip119
\mdffootnoteboxwidth=\skip120
\mdffootnoteboxtotalwidth=\skip121
\mdffootnoteboxheight=\skip122
\mdffootnoteboxdepth=\skip123
\mdffootnoteboxtotalheight=\skip124
\mdftotallinewidth=\skip125
\mdfboundingboxwidth=\skip126
\mdfboundingboxtotalwidth=\skip127
\mdfboundingboxheight=\skip128
```

```
\mdfboundingboxdepth=\skip129
\mdfboundingboxtotalheight=\skip130
\mdf@freevspace@length=\skip131
\mdf@horizontalwidthofbox@length=\skip132
\mdf@verticalmarginwhole@length=\skip133
\mdf@horizontalspaceofbox=\skip134
\mdfsubtitleheight=\skip135
\mdfsubsubtitleheight=\skip136
\c@mdfcountframes=\count272

****** mdframed patching \endmdf@trivlist

****** -- success******

\mdf@envdepth=\count273
\c@mdf@env@i=\count274
\c@mdf@env@ii=\count275
\c@mdf@zref@counter=\count276
Package zref Info: New property: mdf@pagevalue on input line 895.
) (c:/texlive/2023/texmf-dist/tex/latex/titlesec/titlesec.sty
Package: titlesec 2023/10/27 v2.16 Sectioning titles
\ttl@box=\box65
\beforetitleunit=\skip137
\aftertitleunit=\skip138
\ttl@plus=\dimen153
\ttl@minus=\dimen154
\ttl@toksa=\toks34
\titlewidth=\dimen155
\titlewidthlast=\dimen156
\titlewidthfirst=\dimen157
) (c:/texlive/2023/texmf-dist/tex/latex/koma-script/scrextend.sty
Package: scrextend 2023/07/07 v3.41 KOMA-Script package (extend other
classes w
ith features of KOMA-Script classes)
(c:/texlive/2023/texmf-dist/tex/latex/koma-script/scrkbase.sty
Package: scrkbase 2023/07/07 v3.41 KOMA-Script package (KOMA-Script-
dependent b
asics and keyval usage)
(c:/texlive/2023/texmf-dist/tex/latex/koma-script/scrbase.sty
Package: scrbase 2023/07/07 v3.41 KOMA-Script package (KOMA-Script-
independent
basics and keyval usage)
(c:/texlive/2023/texmf-dist/tex/latex/koma-script/scrlfile.sty
Package: scrlfile 2023/07/07 v3.41 KOMA-Script package (file load hooks)
(c:/texlive/2023/texmf-dist/tex/latex/koma-script/scrlfile-hook.sty
Package: scrlfile-hook 2023/07/07 v3.41 KOMA-Script package (using LaTeX
hooks)

(c:/texlive/2023/texmf-dist/tex/latex/koma-script/scrlogo.sty
Package: scrlogo 2023/07/07 v3.41 KOMA-Script package (logo)
)))
Applying: [2021/05/01] Usage of raw or classic option list on input line
252.
```

```
Already applied: [0000/00/00] Usage of raw or classic option list on
input line
 368.
))
Package scrextend Info: unexpected definition of `\@makefnmark'.
(scrextend)             Trying to patch it on input line 1762.
Package scrextend Info: patch seems to be successfull on input line 1762.
)

LaTeX Font Warning: Font shape `T1/cmr/m/n' in size <7.5> not available
(Font)              size <7> substituted on input line 69.

(c:/texlive/2023/texmf-dist/tex/latex/tools/calc.sty
Package: calc 2023/07/08 v4.3 Infix arithmetic (KKT,FJ)
\calc@Acount=\count277
\calc@Bcount=\count278
\calc@Adimen=\dimen158
\calc@Bdimen=\dimen159
\calc@Askip=\skip139
\calc@Bskip=\skip140
LaTeX Info: Redefining \setlength on input line 80.
LaTeX Info: Redefining \addtolength on input line 81.
\calc@Ccount=\count279
\calc@Cskip=\skip141
) (c:/texlive/2023/texmf-dist/tex/latex/geometry/geometry.sty
Package: geometry 2020/01/02 v5.9 Page Geometry
(c:/texlive/2023/texmf-dist/tex/generic/iftex/ifvtex.sty
Package: ifvtex 2019/10/25 v1.7 ifvtex legacy package. Use iftex instead.
)
\Gm@cnth=\count280
\Gm@cntv=\count281
\c@Gm@tempcnt=\count282
\Gm@bindingoffset=\dimen160
\Gm@wd@mp=\dimen161
\Gm@odd@mp=\dimen162
\Gm@even@mp=\dimen163
\Gm@layoutwidth=\dimen164
\Gm@layoutheight=\dimen165
\Gm@layouthoffset=\dimen166
\Gm@layoutvoffset=\dimen167
\Gm@dimlist=\toks35
) (c:/texlive/2023/texmf-dist/tex/latex/preprint/authblk.sty
Package: authblk 2001/02/27 1.3 (PWD)
\affilsep=\skip142
\@affilsep=\skip143
\c@Maxaffil=\count283
\c@authors=\count284
\c@affil=\count285
) (c:/texlive/2023/texmf-dist/tex/latex/footmisc/footmisc.sty
Package: footmisc 2023/07/05 v6.0f a miscellany of footnote facilities
\FN@temptoken=\toks36
\footnotemargin=\dimen168
\@outputbox@depth=\dimen169
```

```
Package footmisc Info: Declaring symbol style bringhurst on input line
696.
Package footmisc Info: Declaring symbol style chicago on input line 704.
Package footmisc Info: Declaring symbol style wiley on input line 713.
Package footmisc Info: Declaring symbol style lamport-robust on input
line 724.

Package footmisc Info: Declaring symbol style lamport* on input line 744.
Package footmisc Info: Declaring symbol style lamport*-robust on input
line 765
.
) (c:/texlive/2023/texmf-dist/tex/latex/fancyhdr/fancyhdr.sty
Package: fancyhdr 2022/11/09 v4.1 Extensive control of page headers and
footers

\f@nch@headwidth=\skip144
\f@nch@O@elh=\skip145
\f@nch@O@erh=\skip146
\f@nch@O@olh=\skip147
\f@nch@O@orh=\skip148
\f@nch@O@elf=\skip149
\f@nch@O@erf=\skip150
\f@nch@O@olf=\skip151
\f@nch@O@orf=\skip152
) (c:/texlive/2023/texmf-dist/tex/generic/alphalph/alphalph.sty
Package: alphalph 2019/12/09 v2.6 Convert numbers to letters (HO)
(c:/texlive/2023/texmf-dist/tex/generic/intcalc/intcalc.sty
Package: intcalc 2019/12/15 v1.3 Expandable calculations with integers
(HO)
))
\c@authorfn=\count286
(c:/texlive/2023/texmf-dist/tex/latex/abstract/abstract.sty
Package: abstract 2009/06/08 v1.2a configurable abstracts
\abstitleskip=\skip153
\absleftindent=\skip154
\absrightindent=\skip155
\absparindent=\skip156
\absparsep=\skip157
)
Package newfloat Info: New float `keypoints' with options
`placement=t!,name=kp
t' on input line 291.
\c@keypoints=\count287
\newfloat@ftype=\count288
Package newfloat Info: float type `keypoints'=8 on input line 291.
(c:/texlive/2023/texmf-dist/tex/latex/enumitem/enumitem.sty
Package: enumitem 2019/06/20 v3.9 Customized lists
\labelindent=\skip158
\enit@outerparindent=\dimen170
\enit@toks=\toks37
\enit@inbox=\box66
\enit@count@id=\count289
\enitdp@description=\count290
) (c:/texlive/2023/texmf-dist/tex/latex/quoting/quoting.sty
```

```
Package: quoting 2014/01/28 v0.1c Consolidated environment for displayed
text
\quo@toppartop=\skip159
) (c:/texlive/2023/texmf-dist/tex/latex/sttools/stfloats.sty
Package: stfloats 2017/03/27 v3.3 Improve float mechanism and
baselineskip sett
ings
\@dblbotnum=\count291
\c@dblbotnumber=\count292
) (c:/texlive/2023/texmf-dist/tex/latex/booktabs/booktabs.sty
Package: booktabs 2020/01/12 v1.61803398 Publication quality tables
\heavyrulewidth=\dimen171
\lightrulewidth=\dimen172
\cmidrulewidth=\dimen173
\belowrulesep=\dimen174
\belowbottomsep=\dimen175
\aboverulesep=\dimen176
\abovetopsep=\dimen177
\cmidrulesep=\dimen178
\cmidrulekern=\dimen179
\defaultaddspace=\dimen180
\@cmidla=\count293
\@cmidlb=\count294
\@aboverulesep=\dimen181
\@belowrulesep=\dimen182
\@thisruleclass=\count295
\@lastruleclass=\count296
\@thisrulewidth=\dimen183
) (c:/texlive/2023/texmf-dist/tex/latex/tools/tabularx.sty
Package: tabularx 2023/07/08 v2.11c `tabularx' package (DPC)
\TX@col@width=\dimen184
\TX@old@table=\dimen185
\TX@old@col=\dimen186
\TX@target=\dimen187
\TX@delta=\dimen188
\TX@cols=\count297
\TX@ftn=\toks38
)
\enitdp@tablenotes=\count298
(c:/texlive/2023/texmf-dist/tex/latex/caption/caption.sty
Package: caption 2023/08/05 v3.6o Customizing captions (AR)
(c:/texlive/2023/texmf-dist/tex/latex/caption/caption3.sty
Package: caption3 2023/07/31 v2.4d caption3 kernel (AR)
\caption@tempdima=\dimen189
\captionmargin=\dimen190
\caption@leftmargin=\dimen191
\caption@rightmargin=\dimen192
\caption@width=\dimen193
\caption@indent=\dimen194
\caption@parindent=\dimen195
\caption@hangindent=\dimen196
Package caption Info: Standard document class detected.
)
\c@caption@flags=\count299
```

```
\c@continuedfloat=\count300
Package caption Info: rotating package is loaded.
Package caption Info: scrextend package is loaded.
\caption@addmargin@hsize=\dimen197
\caption@addmargin@linewidth=\dimen198
) (c:/texlive/2023/texmf-dist/tex/latex/natbib/natbib.sty
Package: natbib 2010/09/13 8.31b (PWD, AO)
\bibhang=\skip160
\bibsep=\skip161
LaTeX Info: Redefining \cite on input line 694.
\c@NAT@ctr=\count301
)) (c:/texlive/2023/texmf-dist/tex/latex/siunitx/siunitx.sty
Package: siunitx 2024-02-15 v3.3.12 A comprehensive (SI) units package
\l__siunitx_number_uncert_offset_int=\count302
\l__siunitx_number_exponent_fixed_int=\count303
\l__siunitx_number_min_decimal_int=\count304
\l__siunitx_number_min_integer_int=\count305
\l__siunitx_number_round_precision_int=\count306
\l__siunitx_number_lower_threshold_int=\count307
\l__siunitx_number_upper_threshold_int=\count308
\l__siunitx_number_group_first_int=\count309
\l__siunitx_number_group_size_int=\count310
\l__siunitx_number_group_minimum_int=\count311
\l__siunitx_angle_tmp_dim=\dimen199
\l__siunitx_angle_marker_box=\box67
\l__siunitx_angle_unit_box=\box68
\l__siunitx_compound_count_int=\count312
(c:/texlive/2023/texmf-dist/tex/latex/translations/translations.sty
Package: translations 2022/02/05 v1.12 internationalization of LaTeX2e
packages
 (CN)
) (c:/texlive/2023/texmf-dist/tex/latex/amsmath/amstext.sty
Package: amstext 2021/08/26 v2.01 AMS text
(c:/texlive/2023/texmf-dist/tex/latex/amsmath/amsgen.sty
File: amsgen.sty 1999/11/30 v2.0 generic functions
\@emptytoks=\toks39
\ex@=\dimen256
))
\l__siunitx_table_tmp_box=\box69
\l__siunitx_table_tmp_dim=\dimen257
\l__siunitx_table_column_width_dim=\dimen258
\l__siunitx_table_integer_box=\box70
\l__siunitx_table_decimal_box=\box71
\l__siunitx_table_uncert_box=\box72
\l__siunitx_table_before_box=\box73
\l__siunitx_table_after_box=\box74
\l__siunitx_table_before_dim=\dimen259
\l__siunitx_table_carry_dim=\dimen260
\l__siunitx_unit_tmp_int=\count313
\l__siunitx_unit_position_int=\count314
\l__siunitx_unit_total_int=\count315
) (c:/texlive/2023/texmf-dist/tex/latex/tabularray/tabularray.sty
Package: tabularray 2024-02-16 v2024A Typeset tabulars and arrays with
LaTeX3
```

```
\l__tblr_a_int=\count316
\l__tblr_c_int=\count317
\l__tblr_r_int=\count318
\l__tblr_d_dim=\dimen261
\l__tblr_h_dim=\dimen262
\l__tblr_o_dim=\dimen263
\l__tblr_p_dim=\dimen264
\l__tblr_q_dim=\dimen265
\l__tblr_r_dim=\dimen266
\l__tblr_s_dim=\dimen267
\l__tblr_t_dim=\dimen268
\l__tblr_v_dim=\dimen269
\l__tblr_w_dim=\dimen270
\l__tblr_a_box=\box75
\l__tblr_b_box=\box76
\l__tblr_c_box=\box77
\l__tblr_d_box=\box78
\g__tblr_table_count_int=\count319
\c@colnum=\count320
\c@rowcount=\count321
\c@colcount=\count322
\abovesep=\dimen271
\belowsep=\dimen272
\leftsep=\dimen273
\rightsep=\dimen274
\g_tblr_level_int=\count323
\g__tblr_data_row_key_count_int=\count324
\g__tblr_data_column_key_count_int=\count325
\g__tblr_data_cell_key_count_int=\count326
\g__tblr_array_int=\count327
\l__tblr_key_count_int=\count328
\l__tblr_key_quotient_int=\count329
\l__tblr_key_quotient_two_int=\count330
\l__tblr_key_remainder_int=\count331
\g__tblr_data_str_value_count_int=\count332
\rulewidth=\dimen275
\l__tblr_strut_dp_dim=\dimen276
\l__tblr_strut_ht_dim=\dimen277
\g__tblr_cell_wd_dim=\dimen278
\g__tblr_cell_ht_dim=\dimen279
\g__tblr_cell_head_dim=\dimen280
\g__tblr_cell_foot_dim=\dimen281
\l__column_target_dim=\dimen282
\l__tblr_caption_box=\box79
\l__tblr_caption_left_box=\box80
\l__tblr_row_head_box=\box81
\l__tblr_row_foot_box=\box82
\l__tblr_row_head_foot_dim=\dimen283
\tablewidth=\dimen284
\l__tblr_table_firsthead_box=\box83
\l__tblr_table_middlehead_box=\box84
\l__tblr_table_lasthead_box=\box85
\l__tblr_table_firstfoot_box=\box86
\l__tblr_table_middlefoot_box=\box87
```

```
\l__tblr_table_lastfoot_box=\box88
\l__tblr_remain_height_dim=\dimen285
\l__tblr_long_from_int=\count333
\l__tblr_long_to_int=\count334
\l__tblr_curr_i_int=\count335
\l__tblr_prev_i_int=\count336
\l__tblr_table_page_int=\count337
\l__tblr_table_head_box=\box89
\l__tblr_table_foot_box=\box90
\l__tblr_table_head_foot_dim=\dimen286
\l__tblr_table_head_body_foot_dim=\dimen287
\l__tblr_table_box=\box91
\l__tblr_table_hlines_box=\box92
\l__tblr_hline_box=\box93
\l__tblr_row_box=\box94
\l__tblr_col_o_wd_dim=\dimen288
\l__tblr_col_b_wd_dim=\dimen289
\l__tblr_hline_leftskip_dim=\dimen290
\l__tblr_hline_rightskip_dim=\dimen291
\l__tblr_row_ht_dim=\dimen292
\l__tblr_row_dp_dim=\dimen293
\l__tblr_row_abovesep_dim=\dimen294
\l__tblr_row_belowsep_dim=\dimen295
\l__tblr_row_vlines_box=\box95
\l__tblr_vline_box=\box96
\l__tblr_cell_box=\box97
\l__row_upper_dim=\dimen296
\l__row_lower_dim=\dimen297
\l__row_vpace_dim=\dimen298
\l__tblr_vline_aboveskip_dim=\dimen299
\l__tblr_vline_belowskip_dim=\dimen300
\l__tblr_cell_wd_dim=\dimen301
\l__tblr_cell_ht_dim=\dimen302
\l__tblr_diag_box=\box98
) (c:/texlive/2023/texmf-dist/tex/latex/float/float.sty
Package: float 2001/11/08 v1.3d Float enhancements (AL)
\c@float@type=\count338
\float@exts=\toks40
\float@box=\box99
\@float@everytoks=\toks41
\@floatcapt=\box100
) (c:/texlive/2023/texmf-dist/tex/latex/subfigure/subfigure.sty
Package: subfigure 2002/03/15 v2.1.5 subfigure package
\subfigtopskip=\skip162
\subfigcapskip=\skip163
\subfigcaptopadj=\dimen303
\subfigbottomskip=\skip164
\subfigcapmargin=\dimen304
\subfiglabelskip=\skip165
\c@subfigure=\count339
\c@lofdepth=\count340
\c@subtable=\count341
\c@lotdepth=\count342
****************************************
```

```
* Local config file subfigure.cfg used *
****************************************
(c:/texlive/2023/texmf-dist/tex/latex/subfigure/subfigure.cfg)
\subfig@top=\skip166
\subfig@bottom=\skip167
) (c:/texlive/2023/texmf-dist/tex/latex/amsfonts/amsfonts.sty
Package: amsfonts 2013/01/14 v3.01 Basic AMSFonts support
\symAMSa=\mathgroup10
\symAMSb=\mathgroup11
LaTeX Font Info:    Redeclaring math symbol \hbar on input line 98.
LaTeX Info: Redefining \frak on input line 111.
) (c:/texlive/2023/texmf-dist/tex/latex/bbm-macros/bbm.sty
Package: bbm 1999/03/15 V 1.2 provides fonts for set symbols - TH
LaTeX Font Info:    Overwriting math alphabet `\mathbbm' in version
`bold'
(Font)                  U/bbm/m/n --> U/bbm/bx/n on input line 33.
LaTeX Font Info:    Overwriting math alphabet `\mathbbmss' in version
`bold'
(Font)                  U/bbmss/m/n --> U/bbmss/bx/n on input line 35.
) (c:/texlive/2023/texmf-dist/tex/latex/orcidlink/orcidlink.sty
Package: orcidlink 2023/12/30 v1.0.5 Linked ORCiD logo macro package
(c:/texlive/2023/texmf-dist/tex/latex/hyperref/hyperref.sty
Package: hyperref 2024-01-20 v7.01h Hypertext links for LaTeX
(c:/texlive/2023/texmf-dist/tex/generic/pdfescape/pdfescape.sty
Package: pdfescape 2019/12/09 v1.15 Implements pdfTeX's escape features
(HO)
) (c:/texlive/2023/texmf-dist/tex/latex/hycolor/hycolor.sty
Package: hycolor 2020-01-27 v1.10 Color options for hyperref/bookmark
(HO)
) (c:/texlive/2023/texmf-dist/tex/latex/hyperref/nameref.sty
Package: nameref 2023-11-26 v2.56 Cross-referencing by name of section
(c:/texlive/2023/texmf-dist/tex/latex/refcount/refcount.sty
Package: refcount 2019/12/15 v3.6 Data extraction from label references
(HO)
) (c:/texlive/2023/texmf-
dist/tex/generic/gettitlestring/gettitlestring.sty
Package: gettitlestring 2019/12/15 v1.6 Cleanup title references (HO)
)
\c@section@level=\count343
)
\@linkdim=\dimen305
\Hy@linkcounter=\count344
\Hy@pagecounter=\count345
(c:/texlive/2023/texmf-dist/tex/latex/hyperref/pd1enc.def
File: pd1enc.def 2024-01-20 v7.01h Hyperref: PDFDocEncoding definition
(HO)
Now handling font encoding PD1 ...
... no UTF-8 mapping file for font encoding PD1
)
\Hy@SavedSpaceFactor=\count346
(c:/texlive/2023/texmf-dist/tex/latex/hyperref/puenc.def
File: puenc.def 2024-01-20 v7.01h Hyperref: PDF Unicode definition (HO)
Now handling font encoding PU ...
... no UTF-8 mapping file for font encoding PU
```

```
)
Package hyperref Info: Hyper figures OFF on input line 4179.
Package hyperref Info: Link nesting OFF on input line 4184.
Package hyperref Info: Hyper index ON on input line 4187.
Package hyperref Info: Plain pages OFF on input line 4194.
Package hyperref Info: Backreferencing OFF on input line 4199.
Package hyperref Info: Implicit mode ON; LaTeX internals redefined.
Package hyperref Info: Bookmarks ON on input line 4446.
\c@Hy@tempcnt=\count347
LaTeX Info: Redefining \url on input line 4784.
\XeTeXLinkMargin=\dimen306
(c:/texlive/2023/texmf-dist/tex/generic/bitset/bitset.sty
Package: bitset 2019/12/09 v1.3 Handle bit-vector datatype (HO)
(c:/texlive/2023/texmf-dist/tex/generic/bigintcalc/bigintcalc.sty
Package: bigintcalc 2019/12/15 v1.5 Expandable calculations on big
integers (HO
)
))
\Fld@menulength=\count348
\Field@Width=\dimen307
\Fld@charsize=\dimen308
Package hyperref Info: Hyper figures OFF on input line 6063.
Package hyperref Info: Link nesting OFF on input line 6068.
Package hyperref Info: Hyper index ON on input line 6071.
Package hyperref Info: backreferencing OFF on input line 6078.
Package hyperref Info: Link coloring OFF on input line 6083.
Package hyperref Info: Link coloring with OCG OFF on input line 6088.
Package hyperref Info: PDF/A mode OFF on input line 6093.
(c:/texlive/2023/texmf-dist/tex/latex/base/atbegshi-ltx.sty
Package: atbegshi-ltx 2021/01/10 v1.0c Emulation of the original atbegshi
package with kernel methods
)
\Hy@abspage=\count349
\c@Item=\count350
\c@Hfootnote=\count351
)
Package hyperref Info: Driver (autodetected): hpdftex.
(c:/texlive/2023/texmf-dist/tex/latex/hyperref/hpdftex.def
File: hpdftex.def 2024-01-20 v7.01h Hyperref driver for pdfTeX
(c:/texlive/2023/texmf-dist/tex/latex/base/atveryend-ltx.sty
Package: atveryend-ltx 2020/08/19 v1.0a Emulation of the original
atveryend pac
kage
with kernel methods
)
\HyAnn@Count=\count352
\Fld@listcount=\count353
\c@bookmark@seq@number=\count354
(c:/texlive/2023/texmf-dist/tex/latex/rerunfilecheck/rerunfilecheck.sty
Package: rerunfilecheck 2022-07-10 v1.10 Rerun checks for auxiliary files
(HO)
(c:/texlive/2023/texmf-dist/tex/generic/uniquecounter/uniquecounter.sty
Package: uniquecounter 2019/12/15 v1.4 Provide unlimited unique counter
(HO)
```

```
)
Package uniquecounter Info: New unique counter `rerunfilecheck' on input
line 2
85.
)
\Hy@SectionHShift=\skip168
) (c:/texlive/2023/texmf-dist/tex/latex/pgf/frontendlayer/tikz.sty
(c:/texlive/
2023/texmf-dist/tex/latex/pgf/basiclayer/pgf.sty (c:/texlive/2023/texmf-
dist/te
x/latex/pgf/utilities/pgfrcs.sty (c:/texlive/2023/texmf-
dist/tex/generic/pgf/ut
ilities/pgfutil-common.tex
\pgfutil@everybye=\toks42
\pgfutil@tempdima=\dimen309
\pgfutil@tempdimb=\dimen310
) (c:/texlive/2023/texmf-dist/tex/generic/pgf/utilities/pgfutil-latex.def
\pgfutil@abb=\box101
) (c:/texlive/2023/texmf-dist/tex/generic/pgf/utilities/pgfrcs.code.tex
(c:/tex
live/2023/texmf-dist/tex/generic/pgf/pgf.revision.tex)
Package: pgfrcs 2023-01-15 v3.1.10 (3.1.10)
))
Package: pgf 2023-01-15 v3.1.10 (3.1.10)
(c:/texlive/2023/texmf-dist/tex/latex/pgf/basiclayer/pgfcore.sty
(c:/texlive/20
23/texmf-dist/tex/latex/pgf/systemlayer/pgfsys.sty
(c:/texlive/2023/texmf-dist/
tex/generic/pgf/systemlayer/pgfsys.code.tex
Package: pgfsys 2023-01-15 v3.1.10 (3.1.10)
(c:/texlive/2023/texmf-dist/tex/generic/pgf/utilities/pgfkeys.code.tex
\pgfkeys@pathtoks=\toks43
\pgfkeys@temptoks=\toks44

(c:/texlive/2023/texmf-
dist/tex/generic/pgf/utilities/pgfkeyslibraryfiltered.co
de.tex
\pgfkeys@tmptoks=\toks45
))
\pgf@x=\dimen311
\pgf@y=\dimen312
\pgf@xa=\dimen313
\pgf@ya=\dimen314
\pgf@xb=\dimen315
\pgf@yb=\dimen316
\pgf@xc=\dimen317
\pgf@yc=\dimen318
\pgf@xd=\dimen319
\pgf@yd=\dimen320
\w@pgf@writea=\write3
\r@pgf@reada=\read2
\c@pgf@counta=\count355
\c@pgf@countb=\count356
\c@pgf@countc=\count357
```

```
\c@pgf@countd=\count358
\t@pgf@toka=\toks46
\t@pgf@tokb=\toks47
\t@pgf@tokc=\toks48
\pgf@sys@id@count=\count359
(c:/texlive/2023/texmf-dist/tex/generic/pgf/systemlayer/pgf.cfg
File: pgf.cfg 2023-01-15 v3.1.10 (3.1.10)
)
Driver file for pgf: pgfsys-pdftex.def
(c:/texlive/2023/texmf-dist/tex/generic/pgf/systemlayer/pgfsys-pdftex.def
File: pgfsys-pdftex.def 2023-01-15 v3.1.10 (3.1.10)
(c:/texlive/2023/texmf-dist/tex/generic/pgf/systemlayer/pgfsys-common-
pdf.def
File: pgfsys-common-pdf.def 2023-01-15 v3.1.10 (3.1.10)
)))
(c:/texlive/2023/texmf-
dist/tex/generic/pgf/systemlayer/pgfsyssoftpath.code.tex
File: pgfsyssoftpath.code.tex 2023-01-15 v3.1.10 (3.1.10)
\pgfsyssoftpath@smallbuffer@items=\count360
\pgfsyssoftpath@bigbuffer@items=\count361
)
(c:/texlive/2023/texmf-
dist/tex/generic/pgf/systemlayer/pgfsysprotocol.code.tex
File: pgfsysprotocol.code.tex 2023-01-15 v3.1.10 (3.1.10)
)) (c:/texlive/2023/texmf-
dist/tex/generic/pgf/basiclayer/pgfcore.code.tex
Package: pgfcore 2023-01-15 v3.1.10 (3.1.10)
(c:/texlive/2023/texmf-dist/tex/generic/pgf/math/pgfmath.code.tex
(c:/texlive/2
023/texmf-dist/tex/generic/pgf/math/pgfmathutil.code.tex)
(c:/texlive/2023/texm
f-dist/tex/generic/pgf/math/pgfmathparser.code.tex
\pgfmath@dimen=\dimen321
\pgfmath@count=\count362
\pgfmath@box=\box102
\pgfmath@toks=\toks49
\pgfmath@stack@operand=\toks50
\pgfmath@stack@operation=\toks51
) (c:/texlive/2023/texmf-
dist/tex/generic/pgf/math/pgfmathfunctions.code.tex)
(c:/texlive/2023/texmf-
dist/tex/generic/pgf/math/pgfmathfunctions.basic.code.te
x)
(c:/texlive/2023/texmf-
dist/tex/generic/pgf/math/pgfmathfunctions.trigonometric
.code.tex)
(c:/texlive/2023/texmf-
dist/tex/generic/pgf/math/pgfmathfunctions.random.code.t
ex)
(c:/texlive/2023/texmf-
dist/tex/generic/pgf/math/pgfmathfunctions.comparison.co
de.tex)
(c:/texlive/2023/texmf-
dist/tex/generic/pgf/math/pgfmathfunctions.base.code.tex
```

```
)
(c:/texlive/2023/texmf-
dist/tex/generic/pgf/math/pgfmathfunctions.round.code.te
x)
(c:/texlive/2023/texmf-
dist/tex/generic/pgf/math/pgfmathfunctions.misc.code.tex
)
(c:/texlive/2023/texmf-
dist/tex/generic/pgf/math/pgfmathfunctions.integerarithm
etics.code.tex) (c:/texlive/2023/texmf-
dist/tex/generic/pgf/math/pgfmathcalc.co
de.tex) (c:/texlive/2023/texmf-
dist/tex/generic/pgf/math/pgfmathfloat.code.tex
\c@pgfmathroundto@lastzeros=\count363
)) (c:/texlive/2023/texmf-dist/tex/generic/pgf/math/pgfint.code.tex)
(c:/texliv
e/2023/texmf-dist/tex/generic/pgf/basiclayer/pgfcorepoints.code.tex
File: pgfcorepoints.code.tex 2023-01-15 v3.1.10 (3.1.10)
\pgf@picminx=\dimen322
\pgf@picmaxx=\dimen323
\pgf@picminy=\dimen324
\pgf@picmaxy=\dimen325
\pgf@pathminx=\dimen326
\pgf@pathmaxx=\dimen327
\pgf@pathminy=\dimen328
\pgf@pathmaxy=\dimen329
\pgf@xx=\dimen330
\pgf@xy=\dimen331
\pgf@yx=\dimen332
\pgf@yy=\dimen333
\pgf@zx=\dimen334
\pgf@zy=\dimen335
)
(c:/texlive/2023/texmf-
dist/tex/generic/pgf/basiclayer/pgfcorepathconstruct.cod
e.tex
File: pgfcorepathconstruct.code.tex 2023-01-15 v3.1.10 (3.1.10)
\pgf@path@lastx=\dimen336
\pgf@path@lasty=\dimen337
)
(c:/texlive/2023/texmf-
dist/tex/generic/pgf/basiclayer/pgfcorepathusage.code.te
x
File: pgfcorepathusage.code.tex 2023-01-15 v3.1.10 (3.1.10)
\pgf@shorten@end@additional=\dimen338
\pgf@shorten@start@additional=\dimen339
) (c:/texlive/2023/texmf-
dist/tex/generic/pgf/basiclayer/pgfcorescopes.code.tex
File: pgfcorescopes.code.tex 2023-01-15 v3.1.10 (3.1.10)
\pgfpic=\box103
\pgf@hbox=\box104
\pgf@layerbox@main=\box105
\pgf@picture@serial@count=\count364
)
```

```
(c:/texlive/2023/texmf-
dist/tex/generic/pgf/basiclayer/pgfcoregraphicstate.code
.tex
File: pgfcoregraphicstate.code.tex 2023-01-15 v3.1.10 (3.1.10)
\pgflinewidth=\dimen340
)
(c:/texlive/2023/texmf-
dist/tex/generic/pgf/basiclayer/pgfcoretransformations.c
ode.tex
File: pgfcoretransformations.code.tex 2023-01-15 v3.1.10 (3.1.10)
\pgf@pt@x=\dimen341
\pgf@pt@y=\dimen342
\pgf@pt@temp=\dimen343
) (c:/texlive/2023/texmf-
dist/tex/generic/pgf/basiclayer/pgfcorequick.code.tex
File: pgfcorequick.code.tex 2023-01-15 v3.1.10 (3.1.10)
) (c:/texlive/2023/texmf-
dist/tex/generic/pgf/basiclayer/pgfcoreobjects.code.te
x
File: pgfcoreobjects.code.tex 2023-01-15 v3.1.10 (3.1.10)
)
(c:/texlive/2023/texmf-
dist/tex/generic/pgf/basiclayer/pgfcorepathprocessing.co
de.tex
File: pgfcorepathprocessing.code.tex 2023-01-15 v3.1.10 (3.1.10)
) (c:/texlive/2023/texmf-
dist/tex/generic/pgf/basiclayer/pgfcorearrows.code.tex
File: pgfcorearrows.code.tex 2023-01-15 v3.1.10 (3.1.10)
\pgfarrowsep=\dimen344
) (c:/texlive/2023/texmf-
dist/tex/generic/pgf/basiclayer/pgfcoreshade.code.tex
File: pgfcoreshade.code.tex 2023-01-15 v3.1.10 (3.1.10)
\pgf@max=\dimen345
\pgf@sys@shading@range@num=\count365
\pgf@shadingcount=\count366
) (c:/texlive/2023/texmf-
dist/tex/generic/pgf/basiclayer/pgfcoreimage.code.tex
File: pgfcoreimage.code.tex 2023-01-15 v3.1.10 (3.1.10)
)
(c:/texlive/2023/texmf-
dist/tex/generic/pgf/basiclayer/pgfcoreexternal.code.tex
File: pgfcoreexternal.code.tex 2023-01-15 v3.1.10 (3.1.10)
\pgfexternal@startupbox=\box106
) (c:/texlive/2023/texmf-
dist/tex/generic/pgf/basiclayer/pgfcorelayers.code.tex
File: pgfcorelayers.code.tex 2023-01-15 v3.1.10 (3.1.10)
)
(c:/texlive/2023/texmf-
dist/tex/generic/pgf/basiclayer/pgfcoretransparency.code
.tex
File: pgfcoretransparency.code.tex 2023-01-15 v3.1.10 (3.1.10)
)
(c:/texlive/2023/texmf-
dist/tex/generic/pgf/basiclayer/pgfcorepatterns.code.tex
```

```
File: pgfcorepatterns.code.tex 2023-01-15 v3.1.10 (3.1.10)
) (c:/texlive/2023/texmf-
dist/tex/generic/pgf/basiclayer/pgfcorerdf.code.tex
File: pgfcorerdf.code.tex 2023-01-15 v3.1.10 (3.1.10)
))) (c:/texlive/2023/texmf-
dist/tex/generic/pgf/modules/pgfmoduleshapes.code.te
x
File: pgfmoduleshapes.code.tex 2023-01-15 v3.1.10 (3.1.10)
\pgfnodeparttextbox=\box107
) (c:/texlive/2023/texmf-
dist/tex/generic/pgf/modules/pgfmoduleplot.code.tex
File: pgfmoduleplot.code.tex 2023-01-15 v3.1.10 (3.1.10)
)
(c:/texlive/2023/texmf-dist/tex/latex/pgf/compatibility/pgfcomp-version-
0-65.st
y
Package: pgfcomp-version-0-65 2023-01-15 v3.1.10 (3.1.10)
\pgf@nodesepstart=\dimen346
\pgf@nodesepend=\dimen347
)
(c:/texlive/2023/texmf-dist/tex/latex/pgf/compatibility/pgfcomp-version-
1-18.st
y
Package: pgfcomp-version-1-18 2023-01-15 v3.1.10 (3.1.10)
)) (c:/texlive/2023/texmf-dist/tex/latex/pgf/utilities/pgffor.sty
(c:/texlive/2
023/texmf-dist/tex/latex/pgf/utilities/pgfkeys.sty
(c:/texlive/2023/texmf-dist/
tex/generic/pgf/utilities/pgfkeys.code.tex)) (c:/texlive/2023/texmf-
dist/tex/la
tex/pgf/math/pgfmath.sty (c:/texlive/2023/texmf-
dist/tex/generic/pgf/math/pgfma
th.code.tex)) (c:/texlive/2023/texmf-
dist/tex/generic/pgf/utilities/pgffor.code
.tex
Package: pgffor 2023-01-15 v3.1.10 (3.1.10)
\pgffor@iter=\dimen348
\pgffor@skip=\dimen349
\pgffor@stack=\toks52
\pgffor@toks=\toks53
)) (c:/texlive/2023/texmf-
dist/tex/generic/pgf/frontendlayer/tikz/tikz.code.tex
Package: tikz 2023-01-15 v3.1.10 (3.1.10)

(c:/texlive/2023/texmf-
dist/tex/generic/pgf/libraries/pgflibraryplothandlers.co
de.tex
File: pgflibraryplothandlers.code.tex 2023-01-15 v3.1.10 (3.1.10)
\pgf@plot@mark@count=\count367
\pgfplotmarksize=\dimen350
)
\tikz@lastx=\dimen351
\tikz@lasty=\dimen352
\tikz@lastxsaved=\dimen353
```

```
\tikz@lastysaved=\dimen354
\tikz@lastmovetox=\dimen355
\tikz@lastmovetoy=\dimen356
\tikzleveldistance=\dimen357
\tikzsiblingdistance=\dimen358
\tikz@figbox=\box108
\tikz@figbox@bg=\box109
\tikz@tempbox=\box110
\tikz@tempbox@bg=\box111
\tikztreelevel=\count368
\tikznumberofchildren=\count369
\tikznumberofcurrentchild=\count370
\tikz@fig@count=\count371
(c:/texlive/2023/texmf-
dist/tex/generic/pgf/modules/pgfmodulematrix.code.tex
File: pgfmodulematrix.code.tex 2023-01-15 v3.1.10 (3.1.10)
\pgfmatrixcurrentrow=\count372
\pgfmatrixcurrentcolumn=\count373
\pgf@matrix@numberofcolumns=\count374
)
\tikz@expandcount=\count375

(c:/texlive/2023/texmf-
dist/tex/generic/pgf/frontendlayer/tikz/libraries/tikzli
brarytopaths.code.tex
File: tikzlibrarytopaths.code.tex 2023-01-15 v3.1.10 (3.1.10)
)))
(c:/texlive/2023/texmf-
dist/tex/generic/pgf/frontendlayer/tikz/libraries/tikzli
brarysvg.path.code.tex
File: tikzlibrarysvg.path.code.tex 2023-01-15 v3.1.10 (3.1.10)

(c:/texlive/2023/texmf-
dist/tex/generic/pgf/libraries/pgflibrarysvg.path.code.t
ex
File: pgflibrarysvg.path.code.tex 2023-01-15 v3.1.10 (3.1.10)
(c:/texlive/2023/texmf-
dist/tex/generic/pgf/modules/pgfmoduleparser.code.tex
File: pgfmoduleparser.code.tex 2023-01-15 v3.1.10 (3.1.10)
\pgfparserdef@arg@count=\count376
)
\pgf@lib@svg@last@x=\dimen359
\pgf@lib@svg@last@y=\dimen360
\pgf@lib@svg@last@c@x=\dimen361
\pgf@lib@svg@last@c@y=\dimen362
\pgf@lib@svg@count=\count377
\pgf@lib@svg@max@num=\count378
))
\@curXheight=\skip169
) (c:/texlive/2023/texmf-dist/tex/latex/tools/xspace.sty
Package: xspace 2014/10/28 v1.13 Space after command names (DPC,MH)
)

! LaTeX Error: Option clash for package hyperref.
```

```
See the LaTeX manual or LaTeX Companion for explanation.
Type  H <return>  for immediate help.
 ...

l.66 \begin{document}

The package hyperref has already been loaded with options:
  []
There has now been an attempt to load it with options
  [colorlinks,allcolors=black,urlcolor=blue]
Adding the global options:
  ,colorlinks,allcolors=black,urlcolor=blue
to your \documentclass declaration may fix this.
Try typing  <return>  to proceed.

Package translations Info: No language package found. I am going to use
`englis
h' as default language. on input line 66.
LaTeX Font Info:    Trying to load font information for T1+Merriwthr-OsF
on inp
ut line 66.
(c:/texlive/2023/texmf-dist/tex/latex/merriweather/T1Merriwthr-OsF.fd
File: T1Merriwthr-OsF.fd 2020/08/30 (autoinst) Font definitions for
T1/Merriwth
r-OsF.
)
LaTeX Font Info:    Font shape `T1/Merriwthr-OsF/m/n' will be
(Font)              scaled to size 7.5pt on input line 66.
(./main.aux)
\openout1 = `main.aux'.

LaTeX Font Info:    Checking defaults for OML/cmm/m/it on input line 66.
LaTeX Font Info:    ... okay on input line 66.
LaTeX Font Info:    Checking defaults for OMS/cmsy/m/n on input line 66.
LaTeX Font Info:    ... okay on input line 66.
LaTeX Font Info:    Checking defaults for OT1/cmr/m/n on input line 66.
LaTeX Font Info:    ... okay on input line 66.
LaTeX Font Info:    Checking defaults for T1/cmr/m/n on input line 66.
LaTeX Font Info:    ... okay on input line 66.
LaTeX Font Info:    Checking defaults for TS1/cmr/m/n on input line 66.
LaTeX Font Info:    ... okay on input line 66.
LaTeX Font Info:    Checking defaults for OMX/cmex/m/n on input line 66.
LaTeX Font Info:    ... okay on input line 66.
LaTeX Font Info:    Checking defaults for U/cmr/m/n on input line 66.
LaTeX Font Info:    ... okay on input line 66.
LaTeX Font Info:    Checking defaults for PD1/pdf/m/n on input line 66.
LaTeX Font Info:    ... okay on input line 66.
LaTeX Font Info:    Checking defaults for PU/pdf/m/n on input line 66.
LaTeX Font Info:    ... okay on input line 66.
LaTeX Info: Redefining \microtypecontext on input line 66.
Package microtype Info: Applying patch `item' on input line 66.
Package microtype Info: Applying patch `toc' on input line 66.
Package microtype Info: Applying patch `eqnum' on input line 66.
```

```
Package microtype Info: Applying patch `footnote' on input line 66.
Package microtype Info: Applying patch `verbatim' on input line 66.
Package microtype Info: Generating PDF output.
Package microtype Info: Character protrusion enabled (level 2).
Package microtype Info: Using default protrusion set `alltext'.
Package microtype Info: Automatic font expansion enabled (level 2),
(microtype)                stretch: 20, shrink: 20, step: 1, non-selected.
Package microtype Info: Using default expansion set `alltext-nott'.
LaTeX Info: Redefining \showhyphens on input line 66.
Package microtype Info: No adjustment of tracking.
Package microtype Info: No adjustment of interword spacing.
Package microtype Info: No adjustment of character kerning.
Package microtype Info: Loading generic protrusion settings for font
family
(microtype)                `Merriwthr-OsF' (encoding: T1).
(microtype)                For optimal results, create family-specific
settings.
(microtype)                See the microtype manual for details.
LaTeX Font Info:    Redeclaring symbol font `operators' on input line 66.
LaTeX Font Info:    Encoding `OT1' has changed to `T1' for symbol font
(Font)              `operators' in the math version `normal' on input
line 66.
LaTeX Font Info:    Overwriting symbol font `operators' in version
`normal'
(Font)                  OT1/cmr/m/n --> T1/Merriwthr-OsF/m/up on input
line 66.

LaTeX Font Info:    Encoding `OT1' has changed to `T1' for symbol font
(Font)              `operators' in the math version `bold' on input line
66.
LaTeX Font Info:    Overwriting symbol font `operators' in version `bold'
(Font)                  OT1/cmr/bx/n --> T1/Merriwthr-OsF/m/up on input
line 66
.
LaTeX Font Info:    Overwriting symbol font `operators' in version `bold'
(Font)                  T1/Merriwthr-OsF/m/up --> T1/Merriwthr-OsF/b/up
on inpu
t line 66.
LaTeX Font Info:    Redeclaring math alphabet \mathbf on input line 66.
LaTeX Font Info:    Overwriting math alphabet `\mathbf' in version
`normal'
(Font)                  OT1/cmr/bx/n --> T1/Merriwthr-OsF/b/up on input
line 66
.
LaTeX Font Info:    Overwriting math alphabet `\mathbf' in version `bold'
(Font)                  OT1/cmr/bx/n --> T1/Merriwthr-OsF/b/up on input
line 66
.
LaTeX Font Info:    Redeclaring math alphabet \mathsf on input line 66.
LaTeX Font Info:    Overwriting math alphabet `\mathsf' in version
`normal'
(Font)                  OT1/cmss/m/n --> T1/MerriwthrSans-OsF/m/up on
input lin
e 66.
```

```
LaTeX Font Info:    Overwriting math alphabet `\mathsf' in version `bold'
(Font)                  OT1/cmss/bx/n --> T1/MerriwthrSans-OsF/m/up on
input li
ne 66.
LaTeX Font Info:    Redeclaring math alphabet \mathit on input line 66.
LaTeX Font Info:    Overwriting math alphabet `\mathit' in version
`normal'
(Font)                  OT1/cmr/m/it --> T1/Merriwthr-OsF/m/it on input
line 66
.
LaTeX Font Info:    Overwriting math alphabet `\mathit' in version `bold'
(Font)                  OT1/cmr/bx/it --> T1/Merriwthr-OsF/m/it on input
line 6
6.
LaTeX Font Info:    Redeclaring math alphabet \mathtt on input line 66.
LaTeX Font Info:    Overwriting math alphabet `\mathtt' in version
`normal'
(Font)                  OT1/cmtt/m/n --> T1/lmtt/m/up on input line 66.
LaTeX Font Info:    Overwriting math alphabet `\mathtt' in version `bold'
(Font)                  OT1/cmtt/m/n --> T1/lmtt/m/up on input line 66.
LaTeX Font Info:    Overwriting math alphabet `\mathsf' in version `bold'
(Font)                  T1/MerriwthrSans-OsF/m/up --> T1/MerriwthrSans-
OsF/b/up
 on input line 66.
LaTeX Font Info:    Overwriting math alphabet `\mathit' in version `bold'
(Font)                  T1/Merriwthr-OsF/m/it --> T1/Merriwthr-OsF/b/it
on inpu
t line 66.
\c@mv@tabular=\count379
\c@mv@boldtabular=\count380
(c:/texlive/2023/texmf-dist/tex/context/base/mkii/supp-pdf.mkii
[Loading MPS to PDF converter (version 2006.09.02).]
\scratchcounter=\count381
\scratchdimen=\dimen363
\scratchbox=\box112
\nofMPsegments=\count382
\nofMParguments=\count383
\everyMPshowfont=\toks54
\MPscratchCnt=\count384
\MPscratchDim=\dimen364
\MPnumerator=\count385
\makeMPintoPDFobject=\count386
\everyMPtoPDFconversion=\toks55
) (c:/texlive/2023/texmf-dist/tex/latex/epstopdf-pkg/epstopdf-base.sty
Package: epstopdf-base 2020-01-24 v2.11 Base part for package epstopdf
Package epstopdf-base Info: Redefining graphics rule for `.eps' on input
line 4
85.
(c:/texlive/2023/texmf-dist/tex/latex/latexconfig/epstopdf-sys.cfg
File: epstopdf-sys.cfg 2010/07/13 v1.3 Configuration of (r)epstopdf for
TeX Liv
e
))
Package newfloat Info: `float' package detected.
```

```
*geometry* driver: auto-detecting
*geometry* detected driver: pdftex
*geometry* verbose mode - [ preamble ] result:
* driver: pdftex
* paper: a4paper
* layout: <same size as paper>
* layoutoffset:(h,v)=(0.0pt,0.0pt)
* modes: includefoot twoside
* h-part:(L,W,R)=(54.64pt, 488.22787pt, 54.64pt)
* v-part:(T,H,B)=(66.0pt, 745.04684pt, 34.0pt)
* \paperwidth=597.50787pt
* \paperheight=845.04684pt
* \textwidth=488.22787pt
* \textheight=715.04684pt
* \oddsidemargin=-17.62999pt
* \evensidemargin=-17.62999pt
* \topmargin=-47.76999pt
* \headheight=17.5pt
* \headsep=24.0pt
* \topskip=10.0pt
* \footskip=30.0pt
* \marginparwidth=48.0pt
* \marginparsep=10.0pt
* \columnsep=18.0pt
* \skip\footins=22.0pt plus 2.0pt
* \hoffset=0.0pt
* \voffset=0.0pt
* \mag=1000
* \@twocolumntrue
* \@twosidetrue
* \@mparswitchtrue
* \@reversemarginfalse
* (1in=72.27pt=25.4mm, 1cm=28.453pt)

Package caption Info: Begin \AtBeginDocument code.
Package caption Info: float package is loaded.
Package caption Info: hyperref package is loaded.
Package caption Info: subfigure package is loaded.
Package caption Info: End \AtBeginDocument code.

(c:/texlive/2023/texmf-dist/tex/latex/translations/translations-basic-
dictionar
y-english.trsl
File: translations-basic-dictionary-english.trsl (english translation
file `tra
nslations-basic-dictionary')
)
Package translations Info: loading dictionary `translations-basic-
dictionary' f
or `english'. on input line 66.
(c:/texlive/2023/texmf-dist/tex/latex/ninecolors/ninecolors.sty
Package: ninecolors 2022-02-13 v2022D Select colors with proper color
contrast
)
```

```
Package hyperref Info: Link coloring OFF on input line 66.
(./main.out) (./main.out)
\@outlinefile=\write4
\openout4 = `main.out'.

\@gscitedetails=\box113
\@gscitedetailsheight=\skip170
\@gsheadbox=\box114
\@gsheadboxheight=\skip171
LaTeX Font Info:    Font shape `T1/Merriwthr-OsF/b/n' will be
(Font)              scaled to size 6.5pt on input line 66.
LaTeX Font Info:    Calculating math sizes for size <7.5> on input line
66.

LaTeX Font Warning: Font shape `T1/Merriwthr-OsF/m/up' undefined
(Font)              using `T1/Merriwthr-OsF/m/n' instead on input line
66.

LaTeX Font Info:    Font shape `T1/Merriwthr-OsF/m/up' will be
(Font)              scaled to size 6.24973pt on input line 66.
LaTeX Font Info:    Font shape `T1/Merriwthr-OsF/m/up' will be
(Font)              scaled to size 5.24997pt on input line 66.
LaTeX Font Info:    Trying to load font information for U+eur on input
line 66.

(c:/texlive/2023/texmf-dist/tex/latex/amsfonts/ueur.fd
File: ueur.fd 2013/01/14 v3.01 Euler Roman
) (c:/texlive/2023/texmf-dist/tex/latex/microtype/mt-eur.cfg
File: mt-eur.cfg 2006/07/31 v1.1 microtype config. file: AMS Euler Roman
(RS)
)

LaTeX Font Warning: Font shape `OMS/cmsy/m/n' in size <7.5> not available
(Font)              size <7> substituted on input line 66.

LaTeX Font Info:    Trying to load font information for U+euf on input
line 66.

(c:/texlive/2023/texmf-dist/tex/latex/amsfonts/ueuf.fd
File: ueuf.fd 2013/01/14 v3.01 Euler Fraktur
) (c:/texlive/2023/texmf-dist/tex/latex/microtype/mt-euf.cfg
File: mt-euf.cfg 2006/07/03 v1.1 microtype config. file: AMS Euler
Fraktur (RS)

)
LaTeX Font Info:    Trying to load font information for U+eus on input
line 66.

(c:/texlive/2023/texmf-dist/tex/latex/amsfonts/ueus.fd
File: ueus.fd 2013/01/14 v3.01 Euler Script
) (c:/texlive/2023/texmf-dist/tex/latex/microtype/mt-eus.cfg
File: mt-eus.cfg 2006/07/28 v1.2 microtype config. file: AMS Euler Script
(RS)
)
```

```
LaTeX Font Info:    Trying to load font information for U+euex on input
line 66
.
(c:/texlive/2023/texmf-dist/tex/latex/amsfonts/ueuex.fd
File: ueuex.fd 2013/01/14 v3.01 Euler extra symbols
)

LaTeX Font Warning: Font shape `OML/cmm/m/it' in size <7.5> not available
(Font)              size <7> substituted on input line 66.

LaTeX Font Info:    Font shape `T1/Merriwthr-OsF/m/n' will be
(Font)              scaled to size 6.24973pt on input line 66.
LaTeX Font Info:    Font shape `T1/Merriwthr-OsF/m/n' will be
(Font)              scaled to size 5.24997pt on input line 66.
LaTeX Font Info:    Font shape `T1/Merriwthr-OsF/m/it' will be
(Font)              scaled to size 7.5pt on input line 66.
LaTeX Font Info:    Font shape `T1/Merriwthr-OsF/m/it' will be
(Font)              scaled to size 6.24973pt on input line 66.
LaTeX Font Info:    Font shape `T1/Merriwthr-OsF/m/it' will be
(Font)              scaled to size 5.24997pt on input line 66.
LaTeX Font Info:    Trying to load font information for U+msa on input
line 66.

(c:/texlive/2023/texmf-dist/tex/latex/amsfonts/umsa.fd
File: umsa.fd 2013/01/14 v3.01 AMS symbols A
) (c:/texlive/2023/texmf-dist/tex/latex/microtype/mt-msa.cfg
File: mt-msa.cfg 2006/02/04 v1.1 microtype config. file: AMS symbols (a)
(RS)
)
LaTeX Font Info:    Trying to load font information for U+msb on input
line 66.

(c:/texlive/2023/texmf-dist/tex/latex/amsfonts/umsb.fd
File: umsb.fd 2013/01/14 v3.01 AMS symbols B
) (c:/texlive/2023/texmf-dist/tex/latex/microtype/mt-msb.cfg
File: mt-msb.cfg 2005/06/01 v1.0 microtype config. file: AMS symbols (b)
(RS)
)
LaTeX Font Info:    Font shape `T1/Merriwthr-OsF/m/n' will be
(Font)              scaled to size 8.0pt on input line 66.
LaTeX Font Info:    Font shape `T1/Merriwthr-OsF/m/it' will be
(Font)              scaled to size 8.0pt on input line 66.
LaTeX Font Info:    Font shape `T1/Merriwthr-OsF/b/it' will be
(Font)              scaled to size 8.0pt on input line 66.
TextBlockOrigin set to 4pc+6.64pt x 4pc+6pt

Overfull \hbox (54.64pt too wide) in paragraph at lines 85--85
[][]
 []

LaTeX Font Info:    Font shape `T1/Merriwthr-OsF/m/n' will be
(Font)              scaled to size 14.0pt on input line 85.
LaTeX Font Info:    Font shape `T1/Merriwthr-OsF/m/n' will be
(Font)              scaled to size 8.99997pt on input line 85.
```

```
LaTeX Font Info:    Calculating math sizes for size <14> on input line
85.
LaTeX Font Info:    Font shape `T1/Merriwthr-OsF/m/up' will be
(Font)              scaled to size 14.0pt on input line 85.
LaTeX Font Info:    Font shape `T1/Merriwthr-OsF/m/up' will be
(Font)              scaled to size 11.66617pt on input line 85.
LaTeX Font Info:    Font shape `T1/Merriwthr-OsF/m/up' will be
(Font)              scaled to size 9.79996pt on input line 85.
LaTeX Font Info:    Font shape `T1/Merriwthr-OsF/m/n' will be
(Font)              scaled to size 11.66617pt on input line 85.
LaTeX Font Info:    Font shape `T1/Merriwthr-OsF/m/n' will be
(Font)              scaled to size 9.79996pt on input line 85.
LaTeX Font Info:    Font shape `T1/Merriwthr-OsF/m/it' will be
(Font)              scaled to size 14.0pt on input line 85.
LaTeX Font Info:    Font shape `T1/Merriwthr-OsF/m/it' will be
(Font)              scaled to size 11.66617pt on input line 85.
LaTeX Font Info:    Font shape `T1/Merriwthr-OsF/m/it' will be
(Font)              scaled to size 9.79996pt on input line 85.
LaTeX Font Info:    Font shape `T1/Merriwthr-OsF/b/n' will be
(Font)              scaled to size 18.0pt on input line 85.
LaTeX Font Info:    Font shape `T1/Merriwthr-OsF/m/n' will be
(Font)              scaled to size 13.0pt on input line 85.
LaTeX Font Info:    Calculating math sizes for size <13> on input line
85.
LaTeX Font Info:    Font shape `T1/Merriwthr-OsF/m/up' will be
(Font)              scaled to size 13.0pt on input line 85.
LaTeX Font Info:    Font shape `T1/Merriwthr-OsF/m/up' will be
(Font)              scaled to size 10.83287pt on input line 85.
LaTeX Font Info:    Font shape `T1/Merriwthr-OsF/m/up' will be
(Font)              scaled to size 9.09996pt on input line 85.

LaTeX Font Warning: Font shape `OMS/cmsy/m/n' in size <13> not available
(Font)              size <12> substituted on input line 85.


LaTeX Font Warning: Font shape `OML/cmm/m/it' in size <13> not available
(Font)              size <12> substituted on input line 85.

LaTeX Font Info:    Font shape `T1/Merriwthr-OsF/m/n' will be
(Font)              scaled to size 10.83287pt on input line 85.
LaTeX Font Info:    Font shape `T1/Merriwthr-OsF/m/n' will be
(Font)              scaled to size 9.09996pt on input line 85.
LaTeX Font Info:    Font shape `T1/Merriwthr-OsF/m/it' will be
(Font)              scaled to size 13.0pt on input line 85.
LaTeX Font Info:    Font shape `T1/Merriwthr-OsF/m/it' will be
(Font)              scaled to size 10.83287pt on input line 85.
LaTeX Font Info:    Font shape `T1/Merriwthr-OsF/m/it' will be
(Font)              scaled to size 9.09996pt on input line 85.
LaTeX Font Info:    Trying to load font information for TS1+Merriwthr-OsF
on in
put line 85.
(c:/texlive/2023/texmf-dist/tex/latex/merriweather/TS1Merriwthr-OsF.fd
File: TS1Merriwthr-OsF.fd 2020/08/30 (autoinst) Font definitions for
TS1/Merriw
```

```
thr-OsF.
)
LaTeX Font Info:    Font shape `TS1/Merriwthr-OsF/m/n' will be
(Font)              scaled to size 10.83287pt on input line 85.
Package microtype Info: Loading generic protrusion settings for font
family
(microtype)                `Merriwthr-OsF' (encoding: TS1).
(microtype)                For optimal results, create family-specific
settings.
(microtype)                See the microtype manual for details.
LaTeX Font Info:    Font shape `T1/Merriwthr-OsF/m/n' will be
(Font)              scaled to size 9.0pt on input line 85.
LaTeX Font Info:    Font shape `T1/Merriwthr-OsF/m/up' will be
(Font)              scaled to size 9.0pt on input line 85.
LaTeX Font Info:    Font shape `T1/Merriwthr-OsF/m/up' will be
(Font)              scaled to size 7.0pt on input line 85.
LaTeX Font Info:    Font shape `T1/Merriwthr-OsF/m/up' will be
(Font)              scaled to size 5.0pt on input line 85.
LaTeX Font Info:    Font shape `T1/Merriwthr-OsF/m/n' will be
(Font)              scaled to size 7.0pt on input line 85.
LaTeX Font Info:    Font shape `T1/Merriwthr-OsF/m/n' will be
(Font)              scaled to size 5.0pt on input line 85.
LaTeX Font Info:    Font shape `T1/Merriwthr-OsF/m/it' will be
(Font)              scaled to size 9.0pt on input line 85.
LaTeX Font Info:    Font shape `T1/Merriwthr-OsF/m/it' will be
(Font)              scaled to size 7.0pt on input line 85.
LaTeX Font Info:    Font shape `T1/Merriwthr-OsF/m/it' will be
(Font)              scaled to size 5.0pt on input line 85.
LaTeX Font Info:    Font shape `T1/Merriwthr-OsF/m/n' will be
(Font)              scaled to size 6.5pt on input line 85.
LaTeX Font Info:    Calculating math sizes for size <6.5> on input line
85.
LaTeX Font Info:    Font shape `T1/Merriwthr-OsF/m/up' will be
(Font)              scaled to size 6.5pt on input line 85.
LaTeX Font Info:    Font shape `T1/Merriwthr-OsF/m/up' will be
(Font)              scaled to size 5.41643pt on input line 85.
LaTeX Font Info:    Font shape `T1/Merriwthr-OsF/m/up' will be
(Font)              scaled to size 4.54997pt on input line 85.

LaTeX Font Warning: Font shape `OMS/cmsy/m/n' in size <6.5> not available
(Font)              size <6> substituted on input line 85.


LaTeX Font Warning: Font shape `OMS/cmsy/m/n' in size <5.41643> not
available
(Font)              size <5> substituted on input line 85.


LaTeX Font Warning: Font shape `OMS/cmsy/m/n' in size <4.54997> not
available
(Font)              size <5> substituted on input line 85.


LaTeX Font Warning: Font shape `OML/cmm/m/it' in size <6.5> not available
```

```
(Font)              size <6> substituted on input line 85.


LaTeX Font Warning: Font shape `OML/cmm/m/it' in size <5.41643> not
available
(Font)              size <5> substituted on input line 85.


LaTeX Font Warning: Font shape `OML/cmm/m/it' in size <4.54997> not
available
(Font)              size <5> substituted on input line 85.

LaTeX Font Info:    Font shape `T1/Merriwthr-OsF/m/n' will be
(Font)              scaled to size 5.41643pt on input line 85.
LaTeX Font Info:    Font shape `T1/Merriwthr-OsF/m/n' will be
(Font)              scaled to size 4.54997pt on input line 85.
LaTeX Font Info:    Font shape `T1/Merriwthr-OsF/m/it' will be
(Font)              scaled to size 6.5pt on input line 85.
LaTeX Font Info:    Font shape `T1/Merriwthr-OsF/m/it' will be
(Font)              scaled to size 5.41643pt on input line 85.
LaTeX Font Info:    Font shape `T1/Merriwthr-OsF/m/it' will be
(Font)              scaled to size 4.54997pt on input line 85.
LaTeX Font Info:    Font shape `TS1/Merriwthr-OsF/m/n' will be
(Font)              scaled to size 5.41643pt on input line 85.

Overfull \hbox (54.64pt too wide) in paragraph at lines 85--85
[][][]
 []

LaTeX Font Info:    Font shape `T1/Merriwthr-OsF/b/n' will be
(Font)              scaled to size 10.0pt on input line 85.
LaTeX Font Info:    Font shape `T1/Merriwthr-OsF/b/n' will be
(Font)              scaled to size 8.0pt on input line 85.

Overfull \hbox (54.64pt too wide) in paragraph at lines 85--85
[][][]
 []

LaTeX Font Info:    Font shape `T1/Merriwthr-OsF/b/n' will be
(Font)              scaled to size 7.5pt on input line 110.

Package natbib Warning: Citation `phage' on page 1 undefined on input
line 110.



Underfull \vbox (badness 10000) has occurred while \output is active []


Package natbib Warning: Citation `estimate1031' on page 1 undefined on
input li
ne 112.
```

```
Package natbib Warning: Citation `MAV1' on page 1 undefined on input line
112.


Package natbib Warning: Citation `MAV2' on page 1 undefined on input line
112.


Package natbib Warning: Citation `MAV3' on page 1 undefined on input line
112.


Package natbib Warning: Citation `metaunraveling' on page 1 undefined on
input
line 112.


Underfull \vbox (badness 6708) has occurred while \output is active []

LaTeX Font Info:    Font shape `T1/Merriwthr-OsF/m/n' will be
(Font)              scaled to size 7.8pt on input line 116.
LaTeX Font Info:    Font shape `T1/Merriwthr-OsF/b/n' will be
(Font)              scaled to size 7.8pt on input line 116.
[1{c:/texlive/2023/texmf-
var/fonts/map/pdftex/updmap/pdftex.map}{c:/texlive/202
3/texmf-
dist/fonts/enc/dvips/merriweather/merriwthr_posqbl.enc}{c:/texlive/2023
/texmf-dist/fonts/enc/dvips/merriweather/merriwthr_owzwzj.enc}


]

Package natbib Warning: Citation `hostdefinition1' on page 2 undefined on
input
 line 116.


Package natbib Warning: Citation `hostdefinition2' on page 2 undefined on
input
 line 116.


Package natbib Warning: Citation `hostdefinition3' on page 2 undefined on
input
 line 116.


Package natbib Warning: Citation `VHDB' on page 2 undefined on input line
116.


Package natbib Warning: Citation `hostassociation1' on page 2 undefined
on inpu
t line 116.
```

```
Package natbib Warning: Citation `hostassociation2' on page 2 undefined
on inpu
t line 116.

LaTeX Font Info:    Font shape `T1/Merriwthr-OsF/b/n' will be
(Font)              scaled to size 8.5pt on input line 125.

Package natbib Warning: Citation `DNAphage1' on page 2 undefined on input
line
132.


Package natbib Warning: Citation `DNAphage2' on page 2 undefined on input
line
132.


Package natbib Warning: Citation `DNAphage3' on page 2 undefined on input
line
132.


Package natbib Warning: Citation `DNAphage4' on page 2 undefined on input
line
132.


Package natbib Warning: Citation `originRNAV' on page 2 undefined on
input line
 137.


Package natbib Warning: Citation `size' on page 2 undefined on input line
137.


Package natbib Warning: Citation `phage' on page 2 undefined on input
line 137.



Package natbib Warning: Citation `RNAphagecrispr' on page 2 undefined on
input
line 137.


Package natbib Warning: Citation `specificV1' on page 2 undefined on
input line
 142.
```

```
Package natbib Warning: Citation `specificV2' on page 2 undefined on
input line
 142.


Package natbib Warning: Citation `specificV3' on page 2 undefined on
input line
 142.


Package natbib Warning: Citation `specificH1' on page 2 undefined on
input line
 145.


Package natbib Warning: Citation `specificH2' on page 2 undefined on
input line
 145.


Package natbib Warning: Citation `specificH3' on page 2 undefined on
input line
 145.


Package natbib Warning: Citation `specificH4' on page 2 undefined on
input line
 145.


Package natbib Warning: Citation `babayan2018predicting' on page 2
undefined on
 input line 148.


Package natbib Warning: Citation `MLDSPhost' on page 2 undefined on input
line
148.


Package natbib Warning: Citation `Features_cmp' on page 2 undefined on
input li
ne 148.


Package natbib Warning: Citation `DeepHoF' on page 2 undefined on input
line 14
8.

<Fig_1.pdf, id=134, 821.0675pt x 387.4475pt>
File: Fig_1.pdf Graphic file (type pdf)
<use Fig_1.pdf>
Package pdftex.def Info: Fig_1.pdf  used on input line 160.
(pdftex.def)             Requested size: 463.81499pt x 218.86685pt.
```

```
LaTeX Font Info:    Font shape `T1/Merriwthr-OsF/m/n' will be
(Font)              scaled to size 6.0pt on input line 163.
LaTeX Font Info:    Font shape `T1/Merriwthr-OsF/b/n' will be
(Font)              scaled to size 6.0pt on input line 163.
LaTeX Font Info:    Font shape `T1/Merriwthr-OsF/m/it' will be
(Font)              scaled to size 7.8pt on input line 171.
[2]

Package natbib Warning: Citation `MAV3' on page 3 undefined on input line
172.


Package natbib Warning: Citation `viralzone' on page 3 undefined on input
line
172.

LaTeX Font Info:    Font shape `T1/Merriwthr-OsF/b/sl' in size <7.5> not
availa
ble
(Font)              Font shape `T1/Merriwthr-OsF/b/it' tried instead on
input l
ine 178.
LaTeX Font Info:    Font shape `T1/Merriwthr-OsF/b/it' will be
(Font)              scaled to size 7.5pt on input line 178.

Package natbib Warning: Citation `VHDB' on page 3 undefined on input line
179.


Package natbib Warning: Citation `cdhit' on page 3 undefined on input
line 179.



Package natbib Warning: Citation `ncbitaxonomy' on page 3 undefined on
input li
ne 181.


Package natbib Warning: Citation `shi2018evolutionary' on page 3
undefined on i
nput line 188.


Package natbib Warning: Citation `taubenberger2010influenza' on page 3
undefine
d on input line 188.


Package caption Warning: The type was already set to `figure'
(caption)                 on input line 198.
See the caption package documentation for explanation.

LaTeX Font Info:    Font shape `T1/Merriwthr-OsF/b/n' will be
```

```
(Font)              scaled to size 7.0pt on input line 198.
<Table_1.pdf, id=146, 614.295pt x 279.0425pt>
File: Table_1.pdf Graphic file (type pdf)
<use Table_1.pdf>
Package pdftex.def Info: Table_1.pdf  used on input line 199.
(pdftex.def)             Requested size: 488.22787pt x 221.7781pt.
[3 <./Fig_1.pdf>]

Package natbib Warning: Citation `bias_pos_mimic' on page 4 undefined on
input
line 206.


Package natbib Warning: Citation `nu_biased' on page 4 undefined on input
line
206.


Package natbib Warning: Citation `bias_pos_flavivi' on page 4 undefined
on inpu
t line 206.


Package natbib Warning: Citation `bias_pos_attenuation' on page 4
undefined on
input line 206.


Package natbib Warning: Citation `prodigal' on page 4 undefined on input
line 2
06.

LaTeX Font Info:    Font shape `T1/Merriwthr-OsF/m/up' will be
(Font)              scaled to size 7.5pt on input line 206.
LaTeX Font Info:    Font shape `T1/Merriwthr-OsF/b/n' will be
(Font)              scaled to size 6.24973pt on input line 206.
LaTeX Font Info:    Font shape `T1/Merriwthr-OsF/b/n' will be
(Font)              scaled to size 5.24997pt on input line 206.

Underfull \vbox (badness 4001) has occurred while \output is active []


Package natbib Warning: Citation `kmertaxonomy' on page 4 undefined on
input li
ne 212.


Package natbib Warning: Citation `kmerannotation' on page 4 undefined on
input
line 212.


Package natbib Warning: Citation `DNAphage3' on page 4 undefined on input
line
```

```
212.


Package natbib Warning: Citation `babayan2018predicting' on page 4
undefined on
 input line 212.


Package natbib Warning: Citation `bias_pos_flavivi' on page 4 undefined
on inpu
t line 212.

[4 <./Table_1.pdf>]
<Fig_2.pdf, id=245, 525.965pt x 534.99875pt>
File: Fig_2.pdf Graphic file (type pdf)
<use Fig_2.pdf>
Package pdftex.def Info: Fig_2.pdf  used on input line 251.
(pdftex.def)             Requested size: 463.81499pt x 471.80444pt.
LaTeX Font Info:    Font shape `T1/Merriwthr-OsF/m/it' will be
(Font)              scaled to size 6.0pt on input line 254.

Package natbib Warning: Citation `babayan2018predicting' on page 5
undefined on
 input line 283.


Package natbib Warning: Citation `MLDSPhost' on page 5 undefined on input
line
283.


Package natbib Warning: Citation `Features_cmp' on page 5 undefined on
input li
ne 283.


Package natbib Warning: Citation `MLDSPhost' on page 5 undefined on input
line
283.


Package natbib Warning: Citation `DeepHoF' on page 5 undefined on input
line 28
3.


Package natbib Warning: Citation `babayan2018predicting' on page 5
undefined on
 input line 289.


Package natbib Warning: Citation `MLDSPhost' on page 5 undefined on input
line
293.
```

```
Underfull \vbox (badness 2285) has occurred while \output is active []

[5] [6 <./Fig_2.pdf>]
<Fig_3.pdf, id=273, 812.03375pt x 348.30125pt>
File: Fig_3.pdf Graphic file (type pdf)
<use Fig_3.pdf>
Package pdftex.def Info: Fig_3.pdf  used on input line 305.
(pdftex.def)             Requested size: 390.58379pt x 167.52815pt.

Package natbib Warning: Citation `scikit-learn' on page 7 undefined on
input li
ne 315.

<Fig_4.pdf, id=274, 717.68124pt x 386.44376pt>
File: Fig_4.pdf Graphic file (type pdf)
<use Fig_4.pdf>
Package pdftex.def Info: Fig_4.pdf  used on input line 328.
(pdftex.def)             Requested size: 390.58379pt x 210.31578pt.
LaTeX Font Info:    Trying to load font information for U+bbm on input
line 335
.
(c:/texlive/2023/texmf-dist/tex/latex/bbm-macros/ubbm.fd
File: ubbm.fd 1999/03/15 V 1.2 Font definition for bbm font - TH
)

LaTeX Font Warning: Font shape `U/bbm/m/n' in size <7.5> not available
(Font)              size <7> substituted on input line 335.


Package natbib Warning: Citation `ictv_peribunya' on page 7 undefined on
input
line 335.


Package natbib Warning: Citation `ictv_phenui' on page 7 undefined on
input lin
e 335.


Package natbib Warning: Citation `ictv_nairo' on page 7 undefined on
input line
 335.


Underfull \vbox (badness 10000) has occurred while \output is active []


Package natbib Warning: Citation `fungi-inv' on page 7 undefined on input
line
340.
```

```
Package natbib Warning: Citation `plant-inv' on page 7 undefined on input
line
340.


Package natbib Warning: Citation `plant-fungi' on page 7 undefined on
input lin
e 340.


Underfull \vbox (badness 10000) has occurred while \output is active []

[7 <./Fig_3.pdf>]
<Fig_5.pdf, id=294, 577.15625pt x 334.24875pt>
File: Fig_5.pdf Graphic file (type pdf)
<use Fig_5.pdf>
Package pdftex.def Info: Fig_5.pdf  used on input line 363.
(pdftex.def)             Requested size: 390.58379pt x 226.20999pt.

Underfull \vbox (badness 2205) has occurred while \output is active []

[8 <./Fig_4.pdf>]

Package caption Warning: The type was already set to `figure'
(caption)                 on input line 384.
See the caption package documentation for explanation.

<Table_2.pdf, id=321, 412.54124pt x 158.5925pt>
File: Table_2.pdf Graphic file (type pdf)
<use Table_2.pdf>
Package pdftex.def Info: Table_2.pdf  used on input line 385.
(pdftex.def)             Requested size: 219.70105pt x 84.46016pt.
<Fig_6.pdf, id=322, 476.78125pt x 287.0725pt>
File: Fig_6.pdf Graphic file (type pdf)
<use Fig_6.pdf>
Package pdftex.def Info: Fig_6.pdf  used on input line 393.
(pdftex.def)             Requested size: 234.34729pt x 141.10478pt.

Underfull \hbox (badness 10000) in paragraph at lines 382--399
[]$[]$
 []


Package natbib Warning: Citation `DS1_1' on page 9 undefined on input
line 407.



Package natbib Warning: Citation `DS1_2' on page 9 undefined on input
line 407.
```

Package natbib Warning: Citation `DS1_3' on page 9 undefined on input line 407.

Package natbib Warning: Citation `DS1_4' on page 9 undefined on input line 407.

Package natbib Warning: Citation `DS1_5' on page 9 undefined on input line 407.

Package natbib Warning: Citation `DS1_6' on page 9 undefined on input line 407.

Package natbib Warning: Citation `DS1_7' on page 9 undefined on input line 407.

Package natbib Warning: Citation `DS2_1' on page 9 undefined on input line 407.

Package natbib Warning: Citation `DS2_2' on page 9 undefined on input line 407.

Package natbib Warning: Citation `DS2_3' on page 9 undefined on input line 407.

Package natbib Warning: Citation `DS2_4' on page 9 undefined on input line 407.

Package natbib Warning: Citation `DS3_1' on page 9 undefined on input line 407.

Package natbib Warning: Citation `DS3_2' on page 9 undefined on input line 407.

```
Package natbib Warning: Citation `DS3_3' on page 9 undefined on input
line 407.


Package natbib Warning: Citation `DS3_4' on page 9 undefined on input
line 407.


Package natbib Warning: Citation `DS3_5' on page 9 undefined on input
line 407.


Package natbib Warning: Citation `DS3_6' on page 9 undefined on input
line 407.


Package natbib Warning: Citation `DS3_7' on page 9 undefined on input
line 407.


Package natbib Warning: Citation `DS4_1' on page 9 undefined on input
line 407.


Package natbib Warning: Citation `DS4_2' on page 9 undefined on input
line 407.


Underfull \vbox (badness 10000) has occurred while \output is active []

[9 <./Fig_5.pdf> <./Table_2.pdf> <./Fig_6.pdf>]
<Fig_7.pdf, id=366, 796.9775pt x 271.0125pt>
File: Fig_7.pdf Graphic file (type pdf)
<use Fig_7.pdf>
Package pdftex.def Info: Fig_7.pdf  used on input line 416.
(pdftex.def)             Requested size: 239.23433pt x 81.34985pt.

Overfull \hbox (4.12039pt too wide) in paragraph at lines 414--420
[]$[]$
 []

LaTeX Font Info:    Font shape `TS1/Merriwthr-OsF/m/n' will be
(Font)              scaled to size 7.5pt on input line 435.
LaTeX Font Info:    Trying to load font information for T1+lmtt on input
line 4
36.
```

```
(c:/texlive/2023/texmf-dist/tex/latex/lm/t1lmtt.fd
File: t1lmtt.fd 2015/05/01 v1.6.1 Font defs for Latin Modern
)
Package microtype Info: Loading generic protrusion settings for font
family
(microtype)             `lmtt' (encoding: T1).
(microtype)             For optimal results, create family-specific
settings.
(microtype)             See the microtype manual for details.

Underfull \hbox (badness 10000) in paragraph at lines 436--437
[]\T1/Merriwthr-OsF/m/up/7.5 (+20) Project home page: []$\T1/lmtt/m/n/7.5
https
 : / / github . com / GreyGuoweiChen /
 []


Package natbib Warning: Citation `supporting_data' on page 10 undefined
on inpu
t line 445.

[10{c:/texlive/2023/texmf-dist/fonts/enc/dvips/lm/lm-ec.enc}
<./Fig_7.pdf>]
Underfull \hbox (badness 1484) in paragraph at lines 468--469
\T1/Merriwthr-OsF/m/up/7.5 (+20) cil (RGC) Gen-eral Re-search Fund (GRF)
[11206
819, 11217521]
 []


Underfull \hbox (badness 3049) in paragraph at lines 468--469
\T1/Merriwthr-OsF/m/up/7.5 (+20) and Hainan Provin-cial Nat-u-ral Sci-
ence Foun
-da-tion of China
 []

No file main.bbl.

Package natbib Warning: There were undefined citations.

[11

]
enddocument/afterlastpage: lastpage setting LastPage.
(./main.aux)
 ***********
LaTeX2e <2023-11-01> patch level 1
L3 programming layer <2020/03/25>
 ***********

LaTeX Font Warning: Size substitutions with differences
(Font)              up to 1.0pt have occurred.
```

```
LaTeX Font Warning: Some font shapes were not available, defaults
substituted.

Package rerunfilecheck Info: File `main.out' has not changed.
(rerunfilecheck)             Checksum:
0A716A07DCB010583EF8063934DB0651;5473.
 )
Here is how much of TeX's memory you used:
 37070 strings out of 474121
 762052 string characters out of 5747949
 1978190 words of memory out of 5000000
 58063 multiletter control sequences out of 15000+600000
 1878179 words of font info for 633 fonts, out of 8000000 for 9000
 1141 hyphenation exceptions out of 8191
 123i,13n,131p,1818b,971s stack positions out of
10000i,1000n,20000p,200000b,200000s
 <c:/Users/adminuser/.texlive2023/texmf-
var/fonts/pk/ljfour/public/bbm/bbm7.6
00pk><c:/texlive/2023/texmf-
dist/fonts/type1/sorkin/merriweather/Merriwthr-Bold
.pfb><c:/texlive/2023/texmf-
dist/fonts/type1/sorkin/merriweather/Merriwthr-Bold
Italic.pfb><c:/texlive/2023/texmf-
dist/fonts/type1/sorkin/merriweather/Merriwth
r-Italic.pfb><c:/texlive/2023/texmf-
dist/fonts/type1/sorkin/merriweather/Merriw
thr-Regular.pfb><c:/texlive/2023/texmf-
dist/fonts/type1/public/amsfonts/cm/cmsy
7.pfb><c:/texlive/2023/texmf-
dist/fonts/type1/public/amsfonts/euler/euex8.pfb><
c:/texlive/2023/texmf-
dist/fonts/type1/public/lm/lmtt8.pfb><c:/texlive/2023/tex
mf-dist/fonts/type1/public/amsfonts/symbols/msbm7.pfb>
Output written on main.pdf (11 pages, 3771106 bytes).
PDF statistics:
 488 PDF objects out of 1000 (max. 8388607)
 331 compressed objects within 4 object streams
 54 named destinations out of 1000 (max. 500000)
 207642 words of extra memory for PDF output out of 221844 (max.
10000000)
```

manuscript Click here to access/download;Manuscript;main.pdf

Placeholder for OUP logo oup.pdf

Placeholder for journal logo gigascience-logo.pdf

GigaScience, 2023, 1–11

doi: xx.xxxx/xxxx
Manuscript in Preparation
Research

RESEARCH

RNAVirHost: a machine learning-based method for predicting hosts of RNA viruses through viral genomes

Guowei Chen [1], Jingzhe Jiang [2] and Yanni Sun [1,*]

[1]Department of Electrical Engineering, City University of Hong Kong, Hong Kong (SAR), China and [2]Key Laboratory of South China Sea Fishery Resources Exploitation & Utilization, Ministry of Agriculture and Rural Affairs, South China Sea Fisheries Research Institute, Chinese Academy of Fishery Sciences, Guangzhou 510300, China

[*]Correspondence address. Department of Electrical Engineering, City University of Hong Kong, 83 Tat Chee Avenue, Kowloon, Hong Kong (SAR), China. E-mail: yannisun@cityu.edu.hk

Abstract

Background: The high-throughput sequencing technologies have revolutionized the identification of novel RNA viruses. Given that viruses are infectious agents, identifying hosts of these new viruses carries significant implications for public health and provides valuable insights into the dynamics of the microbiome. However, determining the hosts of these newly discovered viruses is not always straightforward, especially in the case of viruses detected in environmental samples. Even for host-associated samples, it is not always correct to assign the sample origin as the host of the identified viruses. The process of assigning hosts to RNA viruses remains challenging due to their high mutation rates and vast diversity.
Results: In this study, we introduce RNAVirHost, a machine learning-based tool that predicts the hosts of RNA viruses solely based on viral genomes. RNAVirHost is a hierarchical classification framework that predicts hosts at different taxonomic levels. We demonstrate the superior accuracy of RNAVirHost in predicting hosts of RNA viruses through comprehensive comparisons with various state-of-the-art techniques. When applying to viruses from novel genera, RNAVirHost achieved the highest accuracy of 84.3%, outperforming the alignment-based strategy by 12.1%.
Conclusions: The application of machine learning models has proven beneficial in predicting hosts of RNA viruses. By integrating genomic traits and sequence homologies, RNAVirHost provides a cost-effective and efficient strategy for host prediction. We believe that RNAVirHost can greatly assist in RNA virus analyses and contribute to pandemic surveillance.

Key words: RNA virus; host prediction; machine learning; metagenomics

Introduction

Viruses are obligate intracellular parasites that depend on living organisms for their replication and survival. RNA viruses, possessing RNA as their genetic material, have the capability to infect a diverse array of organisms. For example, several types of RNA viruses are causal agents of the most disastrous pandemics in human history, including COVID-19, SARS, the annual influenza, etc. Furthermore, certain plant and animal RNA viruses pose a threat to agricultural and animal sectors, jeopardizing crop growth, livestock health, and subsequently leading to substantial economic losses in agriculture and animal husbandry. Besides eukaryotic hosts, some RNA viruses can also infect bacteria and thus directly affect the dynamics of microbiome [?].

Besides its profound influence, viruses are believed to be the most diverse and abundant biological entities in the world [?]. Currently, metagenomic and metatranscriptomic sequencing has emerged as the primary approach for the discovery of novel viruses, as it eliminates the need for virus isolation and cultivation in laboratory settings. This method involves directly sequencing genetic material from host-associated or environmental samples, allowing for the identification of viruses present within these complex ecosystems. While the application of metagenomic/metatranscriptomic sequencing technologies has facilitated the discovery of the viral dark matter [? ? ? ?], how to determine the hosts of the newly identified viruses remains challenging owing to the complex com-

Compiled on: July 22, 2024.
Draft manuscript prepared by the author.

position of the metagenomic sequencing samples.

Understanding the interaction between viruses and their hosts is a fundamental step in characterizing the role of viruses in public health, animal husbandry, agriculture, etc. While the concept of a virus's host has multiple sub-concepts related to the differences in replication, transmission, and pathogenicity [? ? ?], we follow the definition of host in the commonly used database, Virus-Host Database [?], which defines hosts as cellular organisms that viruses can infect and replicate within their cells. Besides their hosts, viruses may be detected in non-host organisms due to the symbiotic relationship, the dietary interaction, or the physical contact, like the bacteria-infecting viruses and the plant-associated viruses found in bird digestive tract [?] and the plant-infecting viruses found in insect vectors [?]. These carriers are not the primary focus of our study.

Related Work

Traditionally, host verification requires stringent experimental contribution, including isolating viral particles from hosts of interest, serological tests, epidemiological investigation, and virus phylogenetic analyses. These processes are time-consuming, labor-intensive, and often require specialized equipment and expertise. While metagenomic sequencing is becoming the main source of novel viruses, the heterogeneous composition made it harder to determine the target hosts. Therefore, when novel viruses rapidly emerge, predicting the hosts from the virus genome sequences, avoiding the tedious laboratory steps, shows its attractive advantage in terms of economy and efficiency.

By far, a number of computational works have been conducted to explore the association between viruses and the potential hosts. Many of these tools focus on prokaryotic viruses. For example, to predict the hosts of phages (viruses infecting prokaryotes), VPF-Class classified a set of Viral Protein Families (VPFs) and aligned the queries virus to the categorized references [?]. RaFAH generated protein clusters, constructed profile Hidden Markov Models (pHMMs), and trained a random forest classification model using the pHMMs alignment score [?]. DeepHost encoded the spaced k-mer feature by a three-dimensional matrix and trained a convolutional neural network (CNN) to predict the hosts [?]. CHERRY integrated various signals, including gene organization, CRISPR, sequence similarity, and k-mer usage, and predicted the virus-host association by a graph convolutional encoder and decoder [?]. Currently, these tools allow host prediction at different ranks, and the accuracy decreases more with a refined host range (e.g., from class to species). Nevertheless, these tools are limited to host prediction for prokaryotic viruses. Neither their tools nor the methodology can be applied to eukaryotic viruses.

Compared to the extensive studies on phages, the host prediction of RNA viruses remains challenging and arduous. The typical genomes of RNA viruses, ranging from 3kbp to 41kbp [?], are smaller than that of DNA viruses (5kbp to 600kbp) and have limited capacity to carry host tropism signals [?]. While the sequence matches between phages and the prokaryotic genomes facilitate the host prediction of phages, they are less common in RNA viruses [? ?]. Furthermore, while many bacterial genomes have been sequenced with metagenomic sequencing, the extensive host range of RNA viruses, limited availability of the potential hosts, and the large sizes of the potential host genomes make adding host genome features very difficult. Finally, the high mutation rate of RNA viruses makes the genomes less conserved, so the existing achievement can not be extended to the novel viruses.

With these challenges, the computational frameworks still show outstanding performance in two host prediction scenarios. The first is to predict the host for a specific group of RNA viruses. ?] counted the spaced amino acid k-mer frequency and trained an Alternating Decision Tree classifier for two families, *Picornaviridae* and *Rhabdoviridae*. ?] encoded the protein sequences by the physical and chemical properties of the amino acid and trained a random forest for the influenza A virus. ?] developed two deep neural network models for the host classification of three viruses, respectively (influenza A virus, rabies lyssavirus, and rotavirus A). These viruses are associated closely with human activity and thus attract attention.

In another scenario, the researchers discuss whether the query viruses will infect the targeted host group, particularly humans and mammals. ?] leveraged the k-mer frequency and designed a k-nearest neighbor model to discriminate the human-infecting viruses from other viruses. ?] applied the reverse-complement neural networks to do read-based prediction of the viral host (human or non-human). ?] investigated the host sharing network of mammalian viruses and trained gradient boosting models to predict the host sharing situation of two viruses. ?] generated a set of protein families that are commonly shared by mammals, studied the correlation between these proteins and cross-species transmission, and trained a random forest model to predict the transmission of viruses.

However, these two categories of works are hard to extend to the host prediction of metagenome-assembled RNA viruses. They overlooked the broader host range of RNA viruses, including additional host candidates, like plants, invertebrates, and fungi. To predict hosts of the increasing novel RNA viruses, some primary explorations have been made. ?] investigated the genomic traits and the sequences homologs of viruses and developed a classification model considering viruses from 12 taxonomic groups and 11 host groups. Building upon Babayan's study, ?] further evaluated the application of Machine learning with digital signal processing-based Structural Patterns (M-SP) of viruses in host prediction. ?] assessed the gene content and the frequency of short sequences and developed a hierarchical host classification framework based on support vector machine. ?] trained a two-branch convolutional neural network to capture the informative motifs and classified the viruses into five host groups. Despite the promising results obtained from these validations, these studies still face the challenge of limited viruses and host ranges.

In this work, we concentrate on predicting hosts of emergent novel viruses and thus developed a hierarchical host classification framework, RNAVirHost. Combining virus taxonomy, genomic traits, and sequence homologies, RNAVirHost allows predicting the hosts using only viral genomes. To cover as many viruses and hosts as we can, RNAVirHost accepts queries from over 30 virus orders, and includes five host types in its first layer, including Chordata (Vertebrate), Invertebrate, Plant, Fungi, Bacteria. After obtaining the prediction results in the first layer, RNAVirHost will perform additional predictions in the second layer to obtain more precise host classification information. By evaluating various features and learning architectures in a more comprehensive database, we demonstrated the outstanding performance of RNAVirHost in host prediction of RNA viruses. We also evaluated RNAVirHost's performance on novel viruses by conducting leave-one-taxon-out experiments. The results of these experiments demonstrated that RNAVirHost can be effectively applied across the diverse landscape of viruses without being limited to specific viral types.

Method

Overview of the method

The framework of RNAVirHost is depicted in Fig. 1. Initially, we categorize the queries based on their taxonomic information to narrow down the potential host range. Then, we extract two types of features, genomic traits and sequence homology, for host prediction using a learning-based model. This two-step approach incorporates both the taxonomic information and the potential host signal

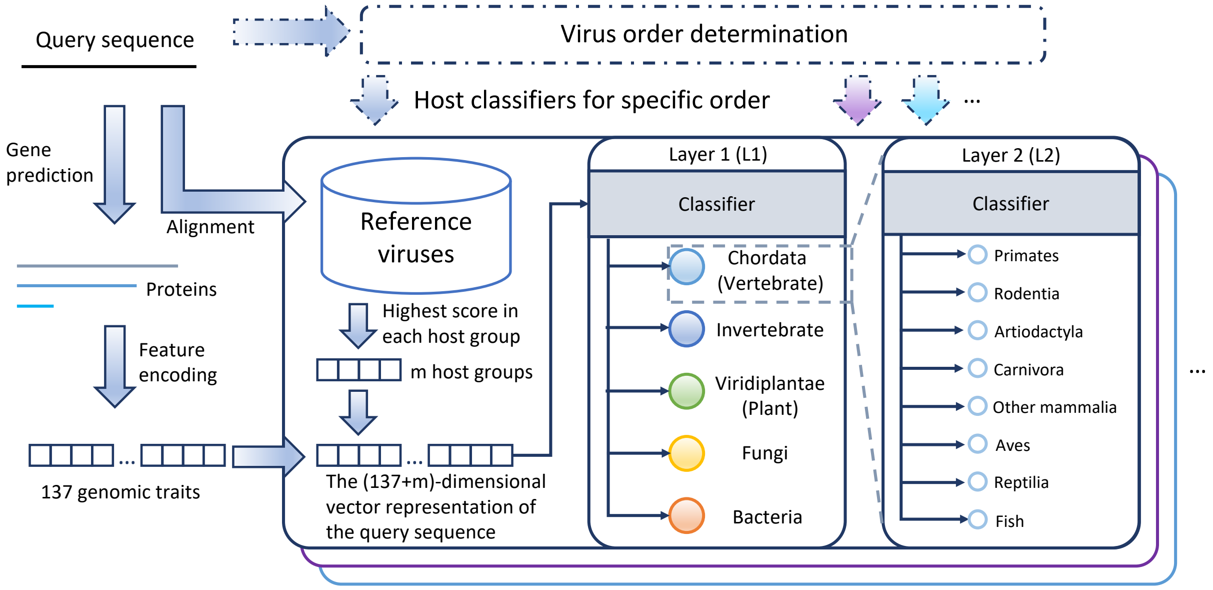

Figure 1. The framework of RNAVirHost. The hierarchical host prediction consists of two classification layers, Layer 1 (L1) and Layer 2 (L2). In Layer 1, we predict hosts at kingdom and phylum level, including Chordata, Invertebrate, Viridiplantae, Fungi, Bacteria. In Layer 2, we further predict the specific host groups under Chordata at class and order level. The genomic traits consist of the usage preference of nucleotide, dinucleotide, codon, and amino acid. We categorized the reference viruses based on their hosts, and m denotes the number of host groups (output labels) in the corresponding classifiers.

in the sequences and is anticipated to enhance the accuracy of our predictions with greater confidence.

The origin and evolution of RNA viruses remain complicated and puzzling [?]. Different groups of viruses have distinct infection mechanisms and divergent host ranges. To benefit the host prediction, we incorporated prior virus taxonomic knowledge in RNAVirHost to mitigate potential interference among viruses originating from different sources. While the lower taxonomic rank may provide a precise host range, the emergent novel sequences may not fit neatly into the existing taxonomic label. Given the trade-off between accommodating more candidates and requiring informative taxonomic knowledge, we categorized viruses into 30 orders following ViralZone [?]. The measure is also expected to improve the learning performance by avoiding imbalance sizes of different virus orders within the same host groups. Then, we built independent classifiers for every order by leveraging the virus members' genomic traits and sequence homologies.

RNAVirHost predicts the most likely hosts of queries hierarchically. The models are of two-layer tree structures, with the first layer corresponding to the host's kingdom and phylum level, and the second layer to the host's class and order level (Section. Labels screening). Once input to corresponding models, the query will be encoded as its genomic traits and sequence homologies (Section. Feature Encoding). RNAVirHost will output the host labels along the tree. Based on comprehensive benchmarks, eXtreme Gradient Boosting (XGBoost), a scalable machine learning technique, delivered the top performance, making it the preferred choice as the default architecture for RNAVirHost. When more data are available, RNAVirHost can be easily scaled to include the new members.

Data preprocessing

Data collection

We curated a comprehensive dataset of RNA viruses for our analysis. First, we collected 6,735 viruses from Virus-Host Database [?], a popular virus-host association database covering complete viral genomes from NCBI RefSeq and other reliable sources. Given the high diversity of RNA viruses, we further supplemented the dataset with 126,417 complete viral sequences with host annotations from NCBI GenBank. To ensure data quality, we filtered GenBank sequences for "complete genome" or "complete cds" with lengths between 3 kbp and 50 kbp, representing the typical genome size range for RNA viruses. To remove redundancy, we combined these two datasets and used CD-HIT [?] to de-replicate the reference sequences at 90% average nucleotide identity and 80% coverage.

Although the processes above promised the quality of the sequences, we need to further check the reliability of the host annotations from GenBank. We cross-referenced them with NCBI Taxonomy database [?] and manual validation. Host tags are standardized to the corresponding scientific names, and the ambiguous annotations were removed. Then, the dataset was used as a high-quality reference dataset as input to the Label screening step. For detailed information on data collection and preprocessing, please refer to the Supplementary Information (Section 1).

Label screening

Although numerous RNA viruses have been found, the current database is biased towards human and mammalian-associated viruses. To better predict the host lineage, we carefully curated the host labels based on both the host phylogenetic tree and the data availability. For each virus order, we built a two-layer host phylogenetic tree. Layer 1 contains 5 branches (Chordata, Invertebrate, Viridiplantae, Fungi, Bacteria), which are categorized into kingdom and phylum level. Layer 2, designed for Chordata subtree, has 10 leaves, which are at the class and order level. Hence, we hierarchically predict the host lineage along the tree. This hierarchical partition acknowledges the practical consideration, that host switch phenomena are more frequently observed at the hosts' class and order level [? ?], and offers users the flexibility to use both layers or one layer of host prediction of RNAVirHost.

Some host labels have only a few recorded infecting viruses and thus are not ready for computational host prediction. For virus orders containing more than 30 viruses, we set the threshold as 10 and only keep host labels with at least 10 infecting viruses. Besides, as we are aiming at predicting the most likely host of the metagenome-assembled viruses, which are expected to be shaped by the selection forces during their long-term co-evolution with hosts, the viral sequences with multiple labels provide redundant and potentially conflicting information and are not included in the assessment. After careful label screening, the dataset contains 14,500 viruses and spans 30 virus orders. Their host distribution is

Table 1. The virus order and host distribution after the label screening.

		Layer 1					Layer 2									
order	num	Chordata	Invertebrate	Viridiplantae	Fungi	Bacteria	Primates	Rodentia	Carnivora	Artiodactyla	Chiroptera	Other Mammalia	Aves	Reptilia	Amphibia	Fish
Ortervirales	2764	92.0%	-	8.0%	-	-	85.9%	0.9%	0.9%	2.0%	-	0.7%	1.6%	-	-	-
Picornavirales	2647	74.2%	12.2%	13.6%	-	-	40.2%	4.1%	4.2%	14.5%	2.6%	1.7%	3.9%	0.9%	-	2.0%
Bunyavirales	1524	52.4%	31.6%	15.2%	0.9%	-	18.0%	17.6%	-	1.6%	1.4%	5.3%	1.7%	5.6%	-	1.2%
Tymovirales	1042	-	2.6%	94.3%	3.1%	-	-	-	-	-	-	-	-	-	-	-
Reovirales	1034	52.9%	33.8%	9.7%	3.6%	-	9.9%	1.3%	2.9%	9.0%	5.5%	3.6%	9.6%	1.3%	-	10.0%
Amarillovirales	817	85.2%	14.8%	-	-	-	54.0%	9.7%	-	10.0%	4.0%	4.2%	1.7%	-	-	1.6%
Mononegavirales	758	57.5%	27.8%	11.3%	3.3%	-	11.3%	6.5%	4.0%	5.4%	11.5%	2.4%	9.0%	1.6%	-	5.9%
Martellivirales	670	5.1%	6.3%	73.7%	14.9%	-	5.1%						-	-	-	-
Nidovirales	622	94.4%	5.6%	-	-	-	4.8%	5.3%	5.6%	36.3%	17.4%	5.6%	13.5%	5.8%	-	-
Patatavirales	558	-	-	100.0%	-	-	-	-	-	-	-	-	-	-	-	-
Ghabrivirales	393	-	14.0%	9.9%	76.1%	-	-	-	-	-	-	-	-	-	-	-
Durnavirales	340	5.0%	-	32.4%	62.6%	-	5.0%						-	-	-	-
Stellavirales	296	100.0%	-	-	-	-	10.1%	12.8%	8.4%	33.1%	5.1%	-	16.2%	-	4.4%	9.8%
Tolivirales	226	-	15.0%	73.9%	11.1%	-	-	-	-	-	-	-	-	-	-	-
Hepelivirales	181	80.1%	10.5%	9.4%	-	-	32.0%	16.6%	-	16.6%	-	8.8%	6.1%	-	-	-
Sobelivirales	120	-	12.5%	87.5%	-	-	-	-	-	-	-	-	-	-	-	-
Blubervirales	108	100.0%	-	-	-	-	75.9%	-	-	-	14.8%	-	9.3%	-	-	-
Cryppavirales	80	-	-	-	100.0%	-	-	-	-	-	-	-	-	-	-	-
Articulavirales	77	100.0%	-	-	-	-	59.7%						16.9%	-	-	23.4%
Jingchuvirales	61	-	100.0%	-	-	-	-	-	-	-	-	-	-	-	-	-
Nodamuvirales	42	-	100.0%	-	-	-	-	-	-	-	-	-	-	-	-	-
Ourlivirales	38	-	-	26.3%	73.7%	-	-	-	-	-	-	-	-	-	-	-
Wolframvirales	23	-	-	-	100.0%	-	-	-	-	-	-	-	-	-	-	-
Mindivirales	22	-	-	-	-	100.0%	-	-	-	-	-	-	-	-	-	-
Norzivirales	21	-	-	-	-	100.0%	-	-	-	-	-	-	-	-	-	-
Serpentovirales	16	-	-	100.0%	-	-	-	-	-	-	-	-	-	-	-	-
Muvirales	9	-	100.0%	-	-	-	-	-	-	-	-	-	-	-	-	-
Yadokarivirales	7	-	-	-	100.0%	-	-	-	-	-	-	-	-	-	-	-
Goujianvirales	3	-	100.0%	-	-	-	-	-	-	-	-	-	-	-	-	-
Timlovirales	1	-	-	-	-	100.0%	-	-	-	-	-	-	-	-	-	-
sum	14500															

Each row represents the host distribution of a virus order. While the column "num" shows the total number of viruses in the order, the following columns represent the percent of viruses infecting the corresponding hosts. Layer 2 consists of the Chordata subgroups from Layer 1. Therefore, the sum of values in the second layer equals the value of "Chordata". In cases (*Martellivirales, Durnavirales, Articulavirales*) where mammalian viruses are less than 50, we merge mammalian members into a single node, Mammalia.

shown in Table 1.

Feature Encoding

Previous studies have extensively explored various features of RNA viruses with different hosts. Both the genomic traits and viruses' sequence homologies facilitated the host prediction of RNA viruses. RNAVirHost relies on a subset of the genomic traits and the sequence homology. A widely accepted hypothesis is that the biases in genomic composition, also named as genomic traits, may hint the natural selection pressure imposed by their hosts. To escape host immune responses and hijack the cellular machinery, viruses tend to mimic the genomic trait usage of their hosts [? ?]. It is reported that Flaviviridae viruses associate with two host groups, vertebrate and invertebrate. The members infecting a single group have similar dinucleotide and codon preference as their hosts do [?]. Besides, the changes of codon pair bias were proven to influence the viruses' pathogenicity [?], showing that the host tropism potentially relates to the genomic traits. To represent the genomic features, we translate the query sequences to proteins using MetaProdigal [?] and generate a 137-dimensional vector $\mathbf{S} \in \mathbb{R}^{137}$, where S_i quantifies the preference of 137 genomic traits, including the usage preference of nucleotide (Eq. 1), dinucleotide (Eq. 2), codon (Eq. 3), and amino acid (Eq. 4).

On the other hand, related viruses tend to infect hosts that share taxonomic associations or have overlapping activity patterns. Thus, the viruses' sequence homology may indicate their host range. The sequence homology is introduced by conducting sequence alignment. The reference sequences are categorized into different groups by their hosts, and we used BLASTN to get the maximum alignment scores of the query against every virus group. The maximum alignment scores against all groups are converted into a m-dimensional vector, $\mathbf{H} \in \mathbb{R}^m$, where m is the number of virus groups (host labels) in the corresponding classifier. Finally, the two vectors, $\mathbf{S}$ and $\mathbf{H}$, are concatenated into a (137+m)-dimensional vector, $\mathbf{X} \in \mathbb{R}^{137+m}$, and used as the representation of the query. The combination of genomic traits and viral sequence homology is expected to facilitate predicting the hosts. Here, we briefly describe the different features, and more details are depicted in the Supplementary Information (section 2).

Features from sequence composition (genomic traits)

The sequence composition describes the relative abundance or occurrence of short strings of nucleotide or amino acid. By far, the k-mer frequency has been widely used in various tasks, like taxonomy classification [?], sequences annotation [?], and host prediction [?]. Based on the length, the k-mer frequency can be defined in different formats. Referring to the previous works on host determination [? ?], we define the composition by the following equations.

$$P_x = n_x / \sum_x n_x \tag{1}$$

$$P_{xy} = \frac{n_{xy} / \sum_{x,y} n_{xy}}{P_x * P_y} \tag{2}$$

$$P_{xyz} = \frac{n_{xyz}}{n_A} \tag{3}$$

where x, y, and z are nucleotide and the codon xyz encodes the amino acid A,

$$P_A = \frac{n_A}{\sum n_A} \tag{4}$$

$$CPS_{x_1y_1z_1,x_2y_2z_2} = \frac{n_{x_1y_1z_1x_2y_2z_2}}{n_{AB} * P_{x_1y_1z_1} * P_{x_2y_2z_2}} \tag{5}$$

where the codon $x_1y_1z_1$ encodes the amino acid A and the adjacent codon $x_2y_2z_2$ encodes B. The occurrence is denoted as n.

To implement normalization, any zero value or missing values are replaced with a small number (1e-4) as a default, and a log2 transformation is applied to all values. Generally, when the value is positive the corresponding feature is over-represented in the genome; otherwise, the feature is deemed to be under-represented.

Features from sequence alignment

We introduced sequence alignment score as a feature vector of the query. Specifically, the reference sequences are categorized into different groups by their hosts, and we aligned the query against the references. The highest alignment score of each group was kept as the potential association between the query and the corresponding host. Hence, the query will be represented as a vector of m*1, where m is the number of host labels. If no alignment is found, we set the association to be one. A log10 transformation is applied to all scores. In this research, we used BLASTN as the default alignment tool. Instead of k-best matches, which tend to be affected by the data imbalance, we only consider the best alignment of each group.

Performance evaluation metrics

The collected 14,500 virus records belong to 30 virus orders. As described in Section Label screening, for each virus order, we examine their host label distribution. We found that 12 virus orders exclusively infected a specific host group, and we directly assigned host labels to them. These virus orders include *Patatavirales, Cryppavirales, Jingchuvirales, Nodamuvirales, Wolframvirales, Mindivirales, Norzivirales, Serpentovirales, Muvirales, Yadokarivirales, Goujianvirales*, and *Timlovirales*. The remaining 18 virus orders involved infections across multiple host groups. Among these 18 orders, thirteen received labels in the second layer of the host phylogenetic tree. Accordingly, we conducted host prediction and evaluation for the corresponding virus orders and host groups.

To evaluate the performance of RNAVirHost, we employed various metrics, including accuracy, precision, F1 score, and prediction rate. Accuracy serves as a fundamental metric, representing the proportion of correctly predicted queries out of the total number of queries. To provide a nuanced assessment of RNAVirHost's performance across different taxonomic levels, we introduced rank-wise accuracy (order-wise, family-wise, and genus-wise), which is computed by averaging the accuracy of respective virus orders, families, and genera.

Additionally, prediction rate quantifies the ratio of output predictions to the total number of queries, while precision captures the ratio of correctly predicted queries to the total number of output predictions. Recognizing the potential bias in reference labels towards human and vertebrate hosts, we included the macro F1 score as an additional evaluation metric. The macro F1 score is calculated by averaging the F1 scores of each individual host label, where the F1 score for a specific label is the harmonic mean of its precision and recall. By employing these metrics, we aim to offer a comprehensive and granular evaluation of RNAVirHost's performance, enabling a robust analysis of its predictive capabilities and strengths.

Result

We conducted comprehensive benchmark experiments on different scenarios to evaluate the performance of RNAVirHost. First, we compared various feature sets and their combinations by 5 fold cross-validation. The comparison showed that the subset of genomic traits outperformed other features. We also assessed the features' contribution by machine learning strategies and validated the choice of genomic traits. We then evaluated different learning architectures, among which XGBoost achieved the highest accuracy and thus was chosen as the default architecture. Second, following the cross-validation setting, we assessed the impact of sequence completeness on host prediction by generating viral sequences with various levels of completeness. Third, we focused on host prediction for novel RNA viruses. Specifically, we evaluated RNAVirHost's performance on novel viruses by conducting leave-one-taxon-out experiments. The results of these experiments demonstrated that RNAVirHost can be effectively applied across the diverse landscape of viruses, without being limited to specific viral types. Finally, to show the accuracy and utility of RNAVirHost in real experiments, we collected some recently identified viruses by researches and tested RNAVirHost's performance on different host groups.

Assessment via cross-validation

In this experiment, we follow the standard evaluation strategy in machine learning to examine the performance of different types of features and learning models. There are many types of features that may help host prediction. We first evaluated the different feature sets and their combinations by stratified 5-fold cross-validation. For each virus order, we stratified the virus into non-overlapping 5-fold by host labels, trained models using 4 out of 5 folds, and tested them in the remaining one. This assessment was performed on each fold, and we presented the overall performance on all 5 folds.

We tested 11 feature sets by ensemble learning, XGBoost. To begin with, we evaluated the classification validity of each feature set without combinations. Referring to Babayan [?], we regarded nucleotide preference, dinucleotide preference, codon usage, codon pair bias, and amino acid usage as one feature set, named as *"Bias"* here. Considering that most RNA viruses are shorter than 50 kbp and there are 3,904 codon pairs (excluding pairs led by stop codons), which is a high-dimensional feature compared to the limited sequences records, we made a subset feature from Bias by excluding the codon pair bias, named as *"sBias"* (subset). Besides the bias-related features, we evaluated 9 more types of features, including BLASTN (best hit), digital signal processing-based Structural Patterns (M-SP), the frequency of 6-mer, 7-mer, 8-mer, amino acid 3-mer (AA3), AA4, Physio-chemical 5-mer (PC5), and PC6 in [? ?]. M-SP involves generating the Fourier transformation (FT) of the biological sequences, computing the correlation coefficients among the FT, and obtaining the distances matrix of sequences through the correlation coefficients [?]. A detailed description of the benchmarked feature can be found in the Supplementary Information (section 2). Then, we assessed feature set combinations among *Bias*, BLASTN, and M-SP. DeepHoF [?], a host prediction tool based on convolutional neural network, provided the classification of viruses associated with plants, germs, invertebrates, humans, and other vertebrates, where germs include bacteria and fungi. We included it in the benchmark assessment in Layer 1 by merging "human" and "other vertebrate" to be Chordata.

The comparison among simple feature sets

As shown in Fig. 2 (A, B), when using each of the 11 feature sets, the classifier with feature *sBias* achieved the highest accuracy of 92.38% and 83.92% for each virus order in L1 and L2, respectively. The models with feature Bias and BLASTN rank the second and the third in performance. The model with *Bias* got accuracy of 90.89% and 82.14%, while using BLASTN got 89.64% and 81.57%. Besides, the M-SP traits, achieving the accuracy of 87.14% and 76.93%, did not provide accurate predictions when used solely. In terms of F1 score, as demonstrated in Fig. S1 (A, B), the model with *sBias* ranked second by achieving F1 scores of 84.82% and 74.03%, outperformed by BLASTN, which got F1 scores of 86.40% and 78.22%. The decrease in F1 scores may result from the data imbalance, which has a more pronounced impact on learning-based method.

The accuracy difference between *sBias* and *Bias* drew our attention, and we calculated the feature contribution to classification performance, as shown in the Supplementary Information (section 3), Table S1 and Fig. S2. The result indicated that most codon pair scores did not contribute to the prediction, which is consistent with previous studies [?]. This can be explained by the sparsity of the codon pair in RNA viruses.

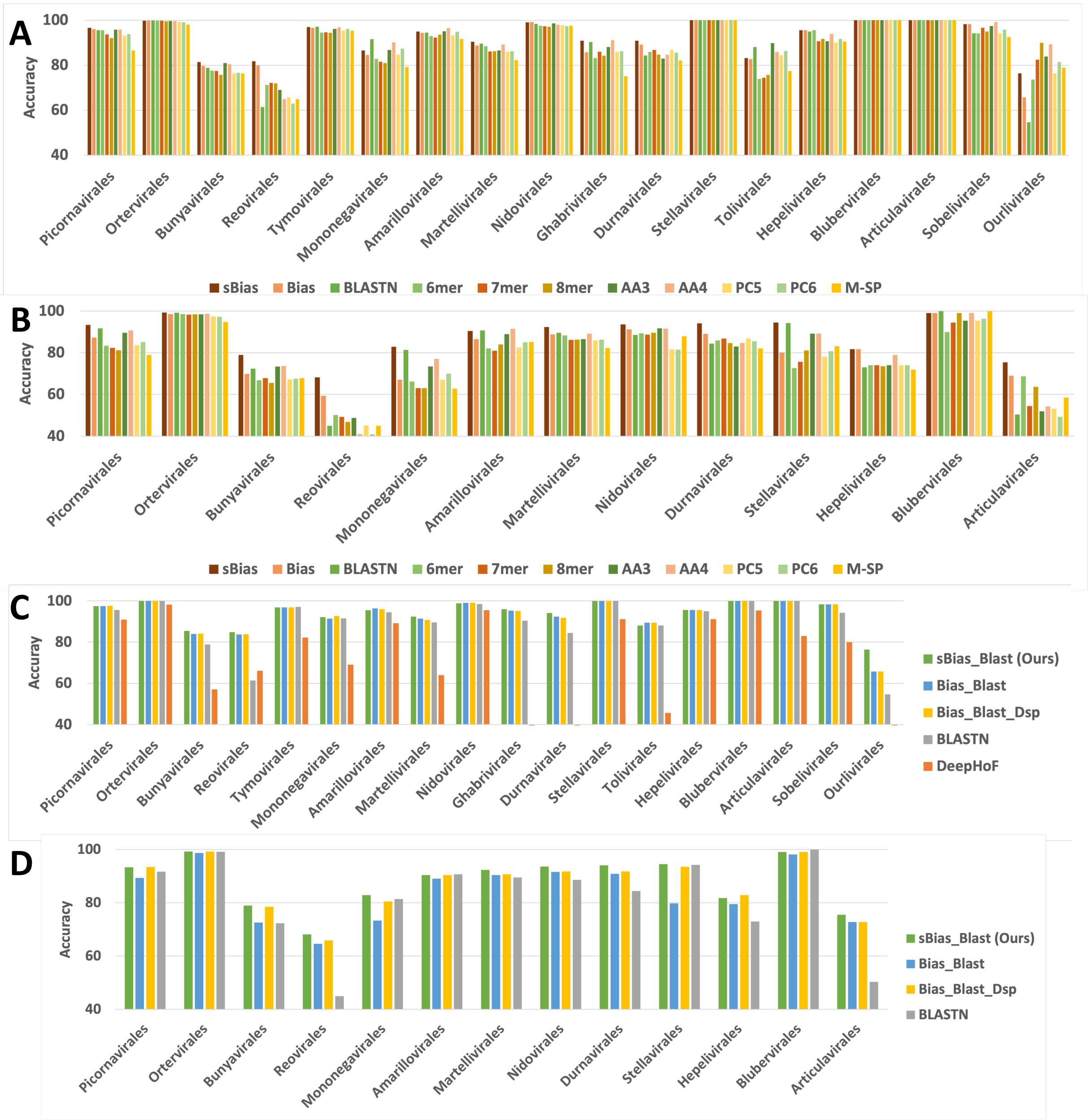

Figure 2. Host prediction performance (accuracy) of different feature sets in Layer 1 (A) and Layer 2 (B). Here, *Bias* denotes a set of genomic traits, including nucleotide preference, dinucleotide preference, codon usage, codon pair bias, and amino acid usage, while *sBias* denotes a subset that excludes the codon pair bias. Host prediction performance (accuracy) of different feature combinations in Layer 1 (C) and Layer 2 (D). Here, *sBias_Blast* is the combination of *sBias* and BLASTN. *Bias_Blast* is the combination of *Bias* and BLASTN. *Bias_Blast_Dsp* is the combination of *Bias*, BLASTN, and M-SP. DeepHoF is designed to predict hosts at the kingdom and phylum level (Layer 1), and its output is transformed to corresponding labels (Germ score to be Fungi; Human score and Vertebrate score to be Chordata). X-axis: Virus orders sorted by size (from largest to smallest). The performance comparison in Layer 2 considers the errors from both Layer 1 and Layer 2. Among the 18 orders, thirteen can be further classified in Layer 2. Therefore, we focused our performance evaluation solely on Layer 2 within these thirteen orders.

The comparison among feature combinations

Out of the 11 feature sets, *Bias*, *sBias*, and BLASTN scores show promising results in the cross-validation experiment. Hence, we further evaluated their combinations and reported the results in Fig. 2 (C, D) and Fig. S1 (C, D). When combined, the models with *sBias_Blast* achieved the best order-wise accuracy of 93.99% and 88.01%. While *Bias_Blast* and *Bias_Blast_Dsp* (the combination of *Bias*, BLASTN, and M-SP as [?]) are the second and third best group, they got little difference in accuracy (93.14% and 86.98% for *Bias_Blast*, 93.14% and 86.96% for *Bias_Blast_Dsp*). With regards to F1 scores, the models with *sBias_Blast* also got the highest F1 scores of 88.41% and 80.59%. The combination significantly improved the prediction performance. The drops of accuracy and F1 scores from *sBias* to *Bias* and that from *sBias_Blast* to *Bias_Blast* validated the feature reduction that excludes the codon pair features. On the other hand, the result implied that the M-SP traits did not significantly benefit the prediction of RNA viruses' hosts.

An important observation is that using BLASTN achieves quite comparable host prediction results as our learning-based methods. We thus further analyzed this. First, to ensure that the improvements observed in the host prediction models are not due to chance events, we conducted a further comparison among models based on different feature sets. This comparison involved examining the accuracy distribution across virus orders, as detailed in Fig. S3. Through the one-sided Wilcoxon test, we demonstrated that the observed improvements in host prediction accuracy are statistically significant and not merely a result of random chance.

Second, it is worth mentioning that when employing a random data partitioning strategy (5-fold cross-validation), it is not unexpected to observe that the predictive performance of BLAST yields

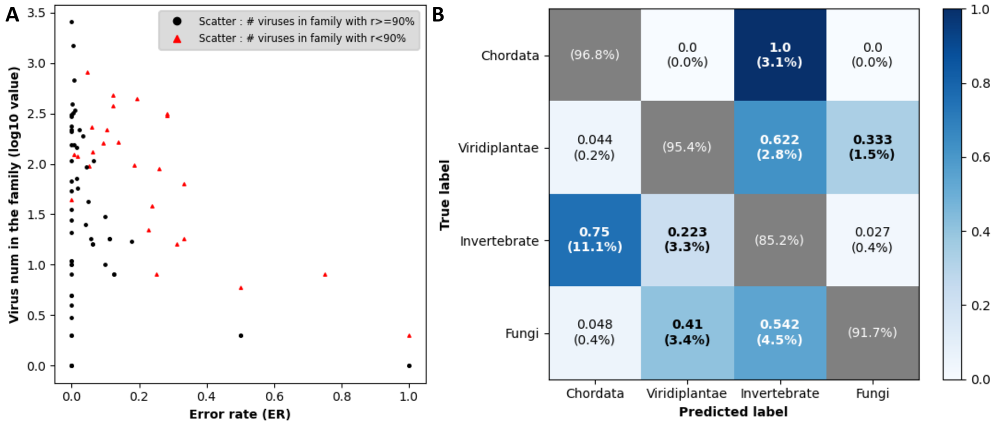

Figure 3. (A) The error rate distribution of RNAVirHost across different virus families in Layer 1. The dots denote the number of virus members in each family (Y-axis) and its corresponding error rate (X-axis). (B) The confusion matrix of RNAVirHost's prediction in Layer 1. The values outside the brackets denote the percentage of the misclassified members, which were normalized to the sum of misclassified viruses of the corresponding groups (by row). The values in the brackets denote the percentage of the prediction labels, normalized to the corresponding groups' total number (by row). The viruses infecting bacteria are limited to specific virus orders, which exclusively infect bacteria. Hence, we do not visualize bacteria here.

similar results as learning-based methods. One main reason for this is that the test viruses, when randomly partitioned, tend to exhibit high similarity to certain viruses in the training data, resulting in a less challenging test scenario. Our next set of experiments will explore more realistic usage scenarios.

The comparison among machine learning architectures

Finally, we conducted an evaluation of several learning architectures, encompassing XGBoost, Gradient Boosting Decision Tree (GBDT), Random Forest (RF), Support Vector Machine with RBF kernel (SVM), Logistic Regression (LR), K-Nearest Neighbors (KNN), and Gaussian Naive Bayes (GNB). Using the scikit-learn package [?], default parameters were employed to train these models, and their performance was assessed using accuracy as the evaluation metric. As demonstrated in Fig. S4, XGBoost exhibited the highest accuracy of 94.0% (L1) and 88.0% (L2), while the second-best architecture (RF) achieved those of 93.3% and 87.5%, and the third best one (GBDT) got 92.7% and 86.2%.

Collectively, the model, trained with XGBoost and the combination of *sBias* and BLASTN, achieved the outperforming accuracy among various learning architectures, feature sets, virus groups, and host ranks.

Handling Out-of-Distribution host labels

As our primary objective is to predict the hosts of metagenome-assembled RNA viruses, we have designed a comprehensive label list that includes four eukaryotic kingdoms and one prokaryotic domain. However, considering the continuous emergence of novel RNA viruses, there are instances where the queried viruses may infect hosts outside the label list. To prioritize precision, we opt to reject virus queries that may not infect the target hosts, even if it leads to a lower prediction rate. To determine the trade-off between prediction rate and precision for both our tool and BLASTN, we analyzed the distribution of prediction scores, which is shown in Supplementary Information (section 4) and Fig. S5. Our analysis revealed that, while predicting hosts for the same number of queries, RNAVirHost achieved higher precision compared to BLASTN. Consequently, we provide users with an empirical prediction score cutoff for each virus order, allowing them to choose predictions with greater confidence. This empirical approach enables users to obtain more reliable predictions.

Family-wise analysis reveals the potential host switch

In comparison to viruses within the same order, viruses in the same family generally have more consistent host ranges. We investigated the host distribution of each virus family and visualized the error rates of virus families in the first layer in Fig. 3A. In the 18 evaluated virus orders, there are 83 families and 13,238 evaluated viruses. In the first layer, the family-wise average accuracy is 87.55%, meaning that 658 out of the evaluated 13,238 viruses got wrong assignments of hosts. It is observed that the misclassification of RNAVirHost mainly distributes to those virus families with mixed host groups. To better visualize the trend, we counted the dominant host labels in every family and the ratio of members infecting the dominant hosts, denoted as r. As a measure of homogeneity, r will have a small value if a family mainly contains viruses infecting different host groups. Otherwise, a virus family infecting only one dominant host group will get an r value close to 1. To examine the homogeneity values of various families, we set r as 90% as the threshold for high and low host homogeneity. Out of the 83 RNA virus families, there are 57 families with over 90% of their members infecting a unique host group ($r \geq 90\%$) and 26 families infecting multiple host groups ($r < 90\%$). In 658 error cases, only 57 (8.66%) came from the former ($r \geq 90\%$), while 593 (90.12%) belong to the latter case ($r < 90\%$), implying that these wrong predictions are mainly from viruses with multiple hosts across phylum. For instances, *Peribunyaviridae* ($r = 56.7\%$), *Phenuiviridae* ($r = 53\%$), *Nairoviridae* $r = 70.8\%$) are virus families in *Bunyavirales*. They contain members that are well known for causing vector-boned diseases [? ? ?]. RNAVirHost achieved accuracy of 71.34%, 70.67%, and 74.16% for the three families, which is lower than the family-wise average accuracy.

Additionally, we generated the confusion matrix to evaluate the predictions made by RNAVirHost, as shown in Fig. 3B. Analysis of the matrix revealed that viruses infecting chordates and Viridiplantae (plants) could be accurately classified. Only 0.2% of viruses infecting Viridiplantae were misclassified as chordate-associated, and no viruses infecting chordates were predicted to infect Viridiplantae. Similarly, viruses infecting chordates and fungi could be clearly distinguished. However, misclassification predominantly occurred among invertebrate-associated queries, with 11.1% of invertebrate-infecting viruses predicted to infect chordates, and 3.1% of chordate-infecting viruses predicted to infect invertebrates. These predictions strongly suggest the presence of potential vector-borne viruses within the dataset. Additionally, errors occurred when distinguishing between viruses that infect invertebrates and those that infect plants and fungi. This confusion may arise due

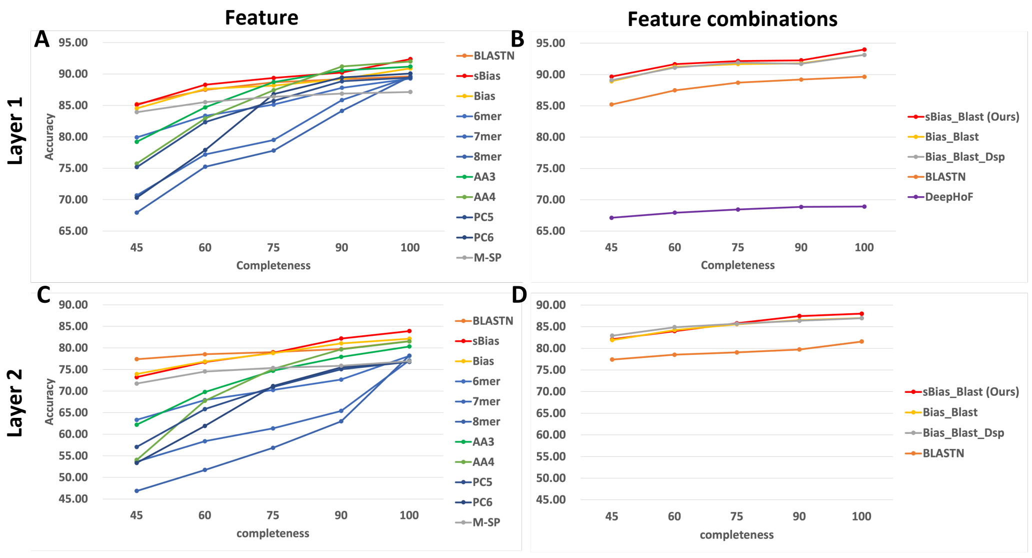

Figure 4. Host prediction performance (order-wise accuracy) of different feature sets in Layer 1 (A, B) and Layer 2 (C, D). X-axis: the completeness of viral sequences. Y-axis: The order-wise accuracy of the corresponding feature set.

to the contact and dietary interactions between invertebrates and plants or fungi [? ?]. Furthermore, there is some overlap between the viruses infecting fungi and those infecting plants, which could result from the symbiotic relationships between certain plants and fungi [?]. Although the focus of this work is placed on predicting the hosts, vector-borne viruses still have a great influence on the performance of host prediction.

Performance on fragmented viral sequences

Given that the metagenome-assembled viruses are not always complete, we assessed the impact of sequence completeness on the host prediction. Following the setting of cross-validation, we partitioned the reference data into training and test sets. Then, we evaluated the robustness of RNAVirHost on the fragmented viral sequences with different length ratios generated from the test set.

First, we generated contigs by cutting the test sequences with four length ratios of the reference sequences: 90%, 75%, 60%, and 45%. Given that the collected reference sequences are of high quality and complete, it is reasonable to regard the length ratio as "completeness". To avoid the potential bias, we generated one contig per reference sequence per group. For every completeness group, there are 13,737 contigs in the 18 targeted virus orders. We used these contigs to evaluate all strategies and reported the order-wise performance in Fig 4 and Fig. S6.

As the queries' completeness decreased, the performance of all strategies deteriorated. Among the simple feature sets, *sbias*, being the most promising feature, outperformed other feature sets in every group except BLASTN in 45% group and AA3 and AA4 in 90% group in Layer 1, and their performance differences are less than 1%. In Layer 2, *sBias*, *Bias*, and BLASTN achieved the highest accuracy in all groups, while they show less accuracy fluctuation with the completeness decrease. These indicate the robustness of *sBias* and *Bias* against the changes of queries' completeness.

Then, we compared the feature combinations. In Layer 1, RNAVirHost achieved higher accuracy than other strategies. In Layer 2, RNAVirHost outperformed others in 75% and 90% groups. In 45% and 60% groups, RNAVirHost got an accuracy of 82.09% and 83.98%, comparable to those of *Bias_Blast_Dsp*, 82.92% and 84.87%. Considering that maintaining a small feature number benefits the generalization of machine learning, we believe that *sBias_Blast* is more suitable in host prediction.

To sum up, *sBias*, *Bias*, and BLASTN showed their strong potential to avoid the effect of incompleteness. RNAVirHost, combining *sBias* and BLASTN, achieved the most advantageous performance in multiple completeness levels.

Performance on novel RNA viruses

With fast accumulated RNA viruses from the environmental sequencing samples, determining hosts of novel viruses is becoming more important. In the second part, we evaluated the capability of RNAVirHost to identify hosts of novel viruses using the leave-one-genus-out strategy. Specifically, we train the model without including specific genera and then assess its performance on those genera to mimic the situation where a novel query, particularly with an unknown genus label, is used as input. In this benchmark, we compared RNAVirHost against the alignment-based method, BLASTN, and two null models (Null 1 and Null 2). Null 1 randomly assigns the host labels following the host label distribution of reference viruses in the training data, while Null 2 determines the query's host using the dominant host label in the training data. The result is shown in Fig. 5. As DeepHoF does not allow us to retrain their model, we cannot include DeepHoF in this experiment.

In our dataset, there are 448 virus genera across 18 orders. We repeated the evaluation for each genus and assessed the genus-wise accuracy of the tools. Besides, some genera might have different host tropisms with their closely related viruses under the same order or family, which poses a great challenge in predicting their hosts. Below, we provide a comprehensive discussion of these challenging cases. We counted the dominant host labels in Layer 1 for each virus order, family, and genus. According to the difference between the host tropisms of the genus and its corresponding order or family, we consider three challenging cases. **Case 1)**: The genus's dominant host label is different from its order's. A typical case is *Nepovirus* in *Picornavirales*. While *Nepovirus* is a plant-infecting virus genus, seventy-four percent of members of the *Picornavirales* infect chordates. **Case 2)** The genus's dominant host label is different from its family's, such as *Seadornavirus* in *Sedoreoviridae*. While mosquitoes are the natural host of *Seadornavirus*, *Spinareoviridae* mainly infect chordates. Some genera in case 2 also belong to case 1, indicating that the host tropism of these genera is divergent from their homologous viruses within the same family and order. **Case 3)**: The members of the genus infect hosts from divergent kingdoms or phyla. By discussing the challenging cases, we expect to exhibit the performance of RNAVirHost in different situations more

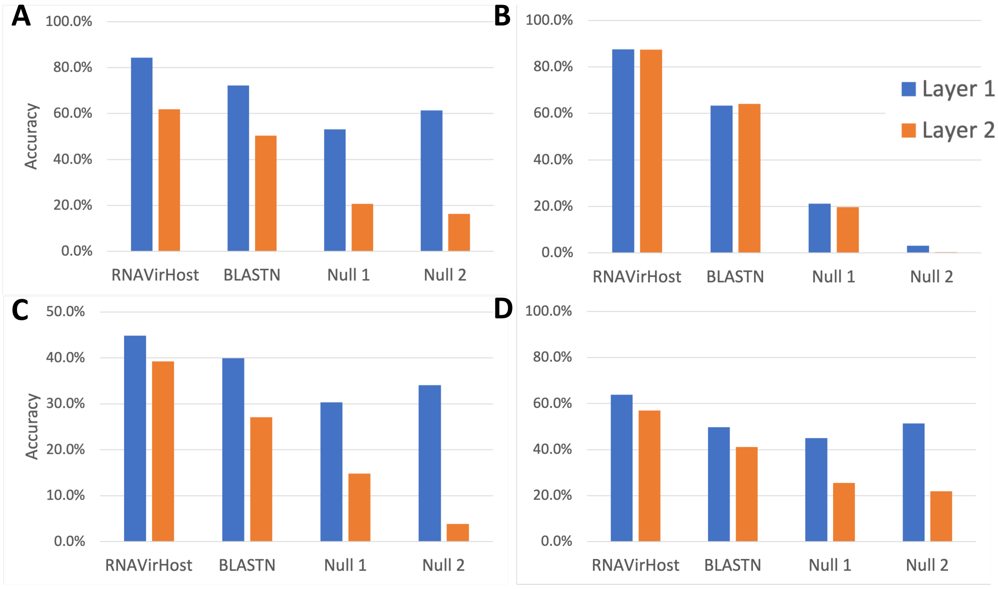

Figure 5. The performance comparison of different strategies in the leave-one-genus-out experiment. The figures demonstrate the average accuracy of A) all 448 genera, B) 138 genera of case 1, whose dominant host label is different from their order's, C) 50 genera of case 2, whose dominant host label is different from its family's, D) 56 genera of cases 3 with members infecting hosts of different kingdoms or phyla. Null 1 randomly assigns the host labels following the distribution of references. Null 2 determines the query's host by the dominant host label in references.

comprehensively.

In all 448 virus genera across 18 orders, RNAVirHost achieved the highest genus-wise average accuracy of 84.3% and 61.9% for L1 and L2, respectively, outperforming BLASTN by 12.1% and 11.5%. There are 138 genera as case 1, whose dominant host labels varied from their order's, 50 genera as case 2, whose dominant host labels differ from their family's, and 56 genera infecting hosts from multiple phyla. RNAVirHost achieved the best result in various cases. Specifically, in 138 genera of case 1, RNAVirHost got accuracy of 87.5%(L1) and 87.4%(L2), outperforming BLASTN by 24.1% and 23.4%. In 50 genera of case 2, RNAVirHost got accuracy of 44.8%(L1) and 39.2%(L2), surpassing BLASTN by 4.9% and 12.1%. In 56 genera with divergent host groups, RNAVirHost achieved an accuracy of 63.8%(L1) and 57.0%(L2), exceeding BLASTN by 14.1% and 15.9%. The improvement of RNAVirHost likely results from the utilization of genomic traits and the superiority of the machine learning method.

Performance on recently identified viruses

Finally, we retrained RNAVirHost on all the reference viruses and assessed its accuracy on identifying hosts for recently identified viruses, whose hosts are derived based on experimental evidence. This process was designed to replicate scenarios that potential users of RNAVirHost might typically encounter.

To cover different host types, we collected 20 works that focused on identifying novel viruses on specific hosts and confirmed the viral infection by laboratory measures. While fifteen were published after 2023, we expanded the dataset by including five more works published before 2023 to cover different hosts as comprehensively as possible. We manually curated the dataset to ensure that the reference dataset did not include any new viral sequences presented in the 20 works. To better demonstrate the accuracy of RNAVirHost on different hosts, we partitioned the sequences into four datasets, which associate with plants, invertebrates, fungi, and fishes (chordates), respectively, corresponding to the first layer's host labels in our tool. Each dataset comprises viral sequences originating from diverse hosts, including those from economically important species like shrimp and salmon, as well as those from less-studied species like seahorses, *Stellaria aquatica* and *Cnidium officinale*. The statistics are listed in Table 2.

Table 2. The statistics regarding taxonomic groups and host labels of the newly sequenced datasets.

	# viruses	# virus orders	Host group	# host classes	# host orders
Dataset 1	21	5	Plant	1	6
Dataset 2	15	3	Invertebrate	2	4
Dataset 3	69	9	Fungi	4	6
Dataset 4	21	6	Chordata - Fish	1	2

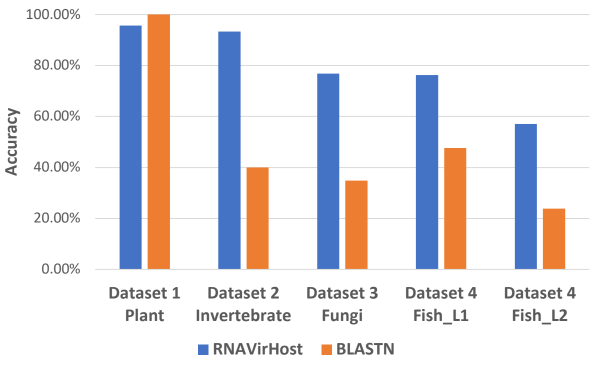

Figure 6. The host prediction accuracy of RNAVirHost and BLASTN on recently identified virus datasets. In dataset 4, L1 and L2 denote Layer 1 and Layer 2, respectively.

In all four datasets, RNAVirHost achieved better or comparable performance than BLASTN, as shown in Fig. 6. The first dataset consists of 21 viruses, whose hosts are from 6 orders under Magnoliopsida plants [? ? ? ? ? ? ?]. RNAVirHost predicted one case to be fungi-associated, which is a member of *Alphapartitivirus*, a genus infecting both plants and fungi. This may suggest that the query has the ability to infect fungi. The second dataset includes 15 viruses, infecting hosts of 4 Arthropoda orders [? ? ? ?]. RNAVirHost achieved a high accuracy of 93.3% while BLASTN got 40%. The only misclassified query obtained a balanced prediction score of

RNAVirHost between Chordata and Invertebrate, which implied its potential to infect vertebrates. The third dataset involves 69 viruses from 9 orders, and their hosts spanned 6 fungi orders [? ? ? ? ? ? ?]. RNAVirHost achieved an accuracy of 76.8%, outperforming BLASTN by 28.6%. Finally, the fourth dataset comprises 21 viruses from 6 orders, infecting seahorses and salmon [? ?]. We achieved accuracy of 76.2% and 57.1% in Layer 1 and Layer 2, respectively, outperforming BLASTN by 28.6% and 33.3%.

Discussion

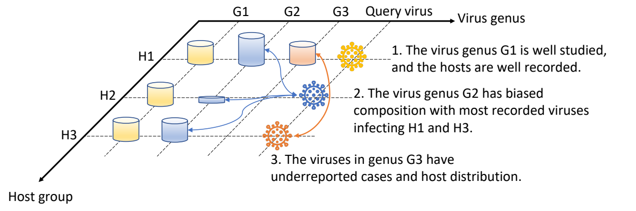

Figure 7. A visualization illustrating the potential sequencing bias in the reference database, posing a challenge to the prediction of hosts. On the virus genus axis, there are three reference genera represented by colors: yellow for G1, blue for G2, and red for G3. The host group axis consists of three distinct host groups (H1, H2, H3) present in the reference database. The cylinders at the intersections of the dashed lines represent viruses belonging to the respective genera that infect the corresponding host groups. The height of each cylinder indicates the relative number of viruses. G1 is extensively studied, and its hosts are well-documented. G2 and G3 are under-studied, resulting in limited information about their hosts.

The application of metagenomic sequencing promotes the identification of novel viruses from host-associated and environmental samples of a diverse set of ecosystems. However, the high diversity of the potential hosts, the sampling method and location, and the heterogeneity of metagenomic sequencing make it hard to determine hosts of the detected viruses, which is a critical step for pandemic surveillance and One Health. In this study, we compared various features used in host prediction of RNA viruses and developed a tool, RNAVirHost, that allows a fast and reliable host prediction by only using the virus sequences. Compared to the laborious and expensive process of host verification in the wet lab, RNAVirHost offers a time and resource-effective strategy. By integrating genomic traits and sequence homology of viruses, RNAVirHost achieved higher accuracy than the alignment-based method. Instead of focusing on vertebrate-associated viruses, we extend the host range to plants, fungi, and bacteria. With the increasing availability of viruses' host annotations, RNAVirHost can be easily scaled to more viruses and hosts.

Despite the potential implications of our study, it is important to acknowledge the challenges that lie ahead for future research in this field. Two particular challenges warrant careful consideration: the unexpected host switch and the existence of multiple hosts. The long-term co-evolution between hosts and viruses may shape the genomic traits of viruses in a way that enables the distinction of their hosts. However, when viruses transfer to new hosts, their genome heritage may no longer reflect their host tropism, and the selection force may not immediately reveal their potential. To anticipate the phenomenon of host switching, additional information is required, including the host lifestyle, virus infection patterns, and virus-host protein-protein interactions. Obtaining such information can provide a more comprehensive understanding of the factors influencing host switching and improve our ability to forecast and respond to pandemics.

Turning our attention to the second challenge, we must address the inherent limitations of RNAVirHost, which is designed to predict the viruses' hosts. In specific cases where viruses can infect multiple hosts, it requires more comprehensive analysis, like single nucleotide variants, to draw the host range accurately. On the other hand, due to the sequencing bias in the database, there exists a knowledge gap that some potential hosts of detected viruses have yet to record. We discussed how the sequencing bias towards different hosts can lead to misclassification, as shown in Fig. 7. Our focus was primarily on the genus-level analysis. To account for the sequencing bias, we categorized virus genera into two groups: well-recorded cases and under-reported cases. Well-recorded cases refer to virus genera that have been exhaustively sequenced in all of their hosts without any sequencing bias, represented by virus genus G1. The availability of a complete reference database greatly benefits the host prediction of new queries. On the contrary, the under-reported cases depict the virus genera whose diversity is under-studied among their hosts, such as the virus genera G2 and G3. In instances where the query virus infects a host that is under-reported in the reference database, the prediction relies heavily on the available hosts and thus may be biased towards hosts that have been extensively recorded. For example, if there is a virus from genus G3 infecting host group H3, but the host recorded in the reference database is H1, there is a high possibility that it will be misclassified as infecting H1. Therefore, the incomplete host range record can negatively impact the accuracy of host prediction. To mitigate this bias, it is crucial to continue expanding and updating the reference database by including data from under-reported hosts. Further understanding of these viruses will enhance the prediction and help to determine the boundary of infection.

Availability of source code and requirements

- Project name: RNAVirHost
- Project home page: `https://github.com/GreyGuoweiChen/VirHost`
- Operating system(s): Platform independent
- Programming language: Python
- Other requirements: Python 3.8, BLAST 2.12.0+, Prodigal 2.6.3+, xgboost 2.0.3, pandas 2.0.3, biopython 1.83, numpy 1.23.5
- License: MIT license
- RRID: SCR_025061

Data availability

All supporting data and materials are available in the GigaScience repository, GigaDB [?].

Additional Files

Supplementary Table S1. The feature contribution of genomic traits to host prediction was assessed using XGBoost models across different virus orders. The contribution is measured as the feature importance. The fold between the observed and expected importance is calculated as the ratio between the mean of importance and the reciprocal of the total number of genomic traits (4041). A higher fold value indicates greater importance demonstrated by the feature.

Supplementary Fig. S1. Host prediction performance (F1 score) of different feature sets in Layer 1 (A) and Layer 2 (B). Host prediction performance (accuracy) of different feature combinations in Layer 1 (C) and Layer 2 (D). X-axis: Virus orders sorted by size (from largest to smallest).

Supplementary Fig. S2. The accuracy of models using genomic traits in the Layer 1 when considering different number of codon pair bias.

Supplementary Fig. S3. The accuracy distribution of models using different feature sets across virus orders. To demonstrate the

effectiveness of our method, we evaluated the median accuracy of different models using Wilcoxon test. Specifically, we conducted one-sided Wilcoxon tests to compare our method with BLASTN (P1) and Bias_Blast (P2). A small p-value ($P < 0.05$) suggests strong evidence to support the alternative hypothesis that the median accuracy of our method is significantly larger than that of the compared methods.

Supplementary Fig. S4. The order-wise accuracy of models using different learning architectures with the same features as RNAVirHost.

Supplementary Fig. S5. The Precision-Prediction_rate curve of RNAVirHost and BLASTN in 9 virus orders that contain more than 500 viruses. A curve closer to the upper-right corner indicates better model performance. The blue dot represents RNAVirHost's performance when applying the empirical prediction score cutoff. The red dot represents the performance of BLASTN's final prediction using the best alignment strategy. The prediction rate is lower than 100% due to the inability to align some query sequences with the reference sequences. Diff denotes the precision difference between RNAVirHost and BLASTN at the same prediction rate.

Supplementary Fig. S6. Host prediction performance (order-wise F1 score) of different feature sets in Layer 1 (A, B) and Layer 2 (C, D). X-axis: the completeness of viral sequences. Y-axis: The order-wise F1 score of the corresponding feature set.

Supplementary Information Section 1: Details of data collection. Section 2: The description of benchmark features. Section 3: Feature selection. Section 4: The trade-off between prediction rate and precision.

Competing Interests

The authors declare that they have no competing interests.

Funding

The study is supported by Hong Kong Research Grants Council (RGC) General Research Fund (GRF) [11206819, 11217521] and Hainan Provincial Natural Science Foundation of China [324CXTD435].

Author's Contributions

GC contributed to the data collection and analysis, experiment execution, framework design, and paper writing. YS conceptualized the study. JJ and YS oversaw the project. All authors reviewed, contributed to, and approved the manuscript.

Table 1: The virus order and host distribution after the label screening. Each row represents the host percent of viruses infecting the corresponding hosts. Layer 2 consists of the Chordata subgroups fron where mammalian viruses are less than 50, we merge mammalian members into a single node, Mam

		Layer 1				
**order**	**num**	**Chordata**	**Invertebrate**	**Viridiplantae**	**Fungi**	**Bacteria**
**Ortervirales**	**2764**	92.0%	-	8.0%	-	-
**Picornavirales**	**2647**	74.2%	12.2%	13.6%	-	-
**Bunyavirales**	**1524**	52.4%	31.6%	15.2%	0.9%	-
**Tymovirales**	**1042**	-	2.6%	94.3%	3.1%	-
**Reovirales**	**1034**	52.9%	33.8%	9.7%	3.6%	-
**Amarillovirales**	**817**	85.2%	14.8%	-	-	-
**Mononegavirales**	**758**	57.5%	27.8%	11.3%	3.3%	-
**Martellivirales**	**670**	5.1%	6.3%	73.7%	14.9%	-
**Nidovirales**	**622**	94.4%	5.6%	-	-	-
**Patatavirales**	**558**	-	-	100.0%	-	-
**Ghabrivirales**	**393**	-	14.0%	9.9%	76.1%	-
**Durnavirales**	**340**	5.0%	-	32.4%	62.6%	-
**Stellavirales**	**296**	100.0%	-	-	-	-
**Tolivirales**	**226**	-	15.0%	73.9%	11.1%	-
**Hepelivirales**	**181**	80.1%	10.5%	9.4%	-	-
**Sobelivirales**	**120**	-	12.5%	87.5%	-	-
**Blubervirales**	**108**	100.0%	-	-	-	-
**Cryppavirales**	**80**	-	-	-	100.0%	-
**Articulavirales**	**77**	100.0%	-	-	-	-
**Jingchuvirales**	**61**	-	100.0%	-	-	-
**Nodamuvirales**	**42**	-	100.0%	-	-	-
**Ourlivirales**	**38**	-	-	26.3%	73.7%	-
**Wolframvirales**	**23**	-	-	-	100.0%	-
**Mindivirales**	**22**	-	-	-	-	100.0%
**Norzivirales**	**21**	-	-	-	-	100.0%
**Serpentovirales**	**16**	-	-	100.0%	-	-
**Muvirales**	**9**	-	100.0%	-	-	-
**Yadokarivirales**	**7**	-	-	-	100.0%	-
**Goujianvirales**	**3**	-	100.0%	-	-	-
**Timlovirales**	**1**	-	-	-	-	100.0%
**sum**	**14500**					

distribution of a virus order. While the column "num" shows the total number of viruses in
n Layer 1. Therefore, the sum of values in the second layer equals the value of "Chordata". I
malia.

				Layer 2	
**Primates**	**Rodentia**	**Carnivora**	**Artiodactyla**	**Chiroptera**	**Other Mammalia**
85.9%	0.9%	0.9%	2.0%	-	0.7%
40.2%	4.1%	4.2%	14.5%	2.6%	1.7%
18.0%	17.6%	-	1.6%	1.4%	5.3%
-	-	-	-	-	-
9.9%	1.3%	2.9%	9.0%	5.5%	3.6%
54.0%	9.7%	-	10.0%	4.0%	4.2%
11.3%	6.5%	4.0%	5.4%	11.5%	2.4%
5.1%					
4.8%	5.3%	5.6%	36.3%	17.4%	5.6%
-	-	-	-	-	-
-	-	-	-	-	-
5.0%					
10.1%	12.8%	8.4%	33.1%	5.1%	-
-	-	-	-	-	-
32.0%	16.6%	-	16.6%	-	8.8%
-	-	-	-	-	-
75.9%	-	-	-	14.8%	-
-	-	-	-	-	-
59.7%					
-	-	-	-	-	-
-	-	-	-	-	-
-	-	-	-	-	-
-	-	-	-	-	-
-	-	-	-	-	-
-	-	-	-	-	-
-	-	-	-	-	-
-	-	-	-	-	-
-	-	-	-	-	-
-	-	-	-	-	-
-	-	-	-	-	-

the order, the following columns represent the
n cases (Martellivirales, Durnavirales, Articulavirales)

Aves	Reptilia	Amphibia	Fish
1.6%	-	-	-
3.9%	0.9%	-	2.0%
1.7%	5.6%	-	1.2%
-	-	-	-
9.6%	1.3%	-	10.0%
1.7%	-	-	1.6%
9.0%	1.6%	-	5.9%
-	-	-	-
13.5%	5.8%	-	-
-	-	-	-
-	-	-	-
-	-	-	-
16.2%	-	4.4%	9.8%
-	-	-	-
6.1%	-	-	-
-	-	-	-
9.3%	-	-	-
-	-	-	-
16.9%	-	-	23.4%
-	-	-	-
-	-	-	-
-	-	-	-
-	-	-	-
-	-	-	-
-	-	-	-
-	-	-	-
-	-	-	-
-	-	-	-
-	-	-	-
-	-	-	-

 Click here to access/download;Table;Table_2.xlsx

Table 2: The statistics regarding taxonomic groups and host labels of the newly sequenced datasets.

	# viruses	# virus orders	Host group	# host classes	# host orders
Dataset 1	21	5	Plant	1	6
Dataset 2	15	3	Invertebrate	2	4
Dataset 3	69	9	Fungi	4	6
Dataset 4	21	6	Chordata - Fish	1	2

Click here to access/download
Supplementary Material
Supplementary Information.pdf

Click here to access/download
Supplementary Material
Supplementary_Table_S1.pdf

Click here to access/download
Supplementary Material
Supplementary_Fig_S1.pdf

Click here to access/download
Supplementary Material
Supplementary_Fig_S2.pdf

Click here to access/download
Supplementary Material
Supplementary_Fig_S3.pdf

Click here to access/download
Supplementary Material
Supplementary_Fig_S4.pdf

Click here to access/download
Supplementary Material
Supplementary_Fig_S5.pdf

Click here to access/download
Supplementary Material
Supplementary_Fig_S6.pdf

Response Letter

Click here to access/download
Supplementary Material
Response_letter.docx
