# Supplementary material for: RNAVirHost: a machine learning–based method for predicting hosts of RNA viruses through viral genomes
Source: Gigascience. 2024 Aug 22;13:giae059. doi: 10.1093/gigascience/giae059 (PMC11340644; doi:10.1093/gigascience/giae059)
Supplement: giae059_Supplemental_Files [file giae059_supplemental_files.zip › Supplementary Information.pdf]

Supplementary Information of RNAVirHost: a machine learning-based method for predicting hosts of RNA viruses through nucleotide sequences

Guowei Chen, Jingze Jiang, and Yanni Sun

1 Details of the data collection

We first collected 6,735 RNA viruses from Virus-Host Database, which are high-quality annotation record that have been manually check by professional scientists. To expand the range of both viruses and hosts, we further downloaded 126,417 RNA virus records with host annotations from NCBI GenBank by the syntax *"Riboviria"[Organism] AND genbank[filter] AND host[All Fields] AND ("3000"[SLEN] : "50000"[SLEN]) AND (complete genome [All Fields] or complete cds [All Fields] or complete genomic [All Fields] or complete cds [All Fields]) NOT Coronaviridae [Organism].* Considering that the viruses in Coronaviridae have been extensively annotated in Virus-Host Database, we remove them from NCBI GenBank to avoid redundancy.

Next, we de-replicated the identical sequences by CD-hit. Following the definition of viral operational taxonomic units (vOTU) of Zayed et al. [1], we clusterd the sequences by 90% average nucleotide identity and 80% coverage for shorter sequences. Specifically, we first conducted CD-hit to remove the redundancy in the high-quality annotations and the data from GenBank respectively by *cd-hit-est -i seq.fasta -o seq_ cdh.fasta -c 0.9 -aS 0.8 -n 8.* Then we clean the overlap between the two datasets by *cd-hit-est-2d -i high_ Q.fasta -i2 GB_ cdh.fasta -o GB_ cdh_ 2D.fasta -c 0.9 -aS 0.8 -n 8.* After clustering, we got 5,785 high-quality records and 14,785 records from GenBank.

Although the "host" field of virus sequence records obtained from NCBI provides information about the hosts, some host annotations may have ambiguous meanings, such as non-scientific names, names with multiple meanings, or typos. In order to maximize the inclusion of relevant data, we replaced the "host" field with the corresponding scientific names recommended by NCBI in cases with typos or non-scientific names. Additionally, we removed cases with multiple meanings. Through this careful curation process, the annotations became explicit enough and we merged the two datasets. This was followed by the final label screening process, as discussed in the paper. Finally, we got 14,500 virus records with host annotation as the reference database.

2 The description of benchmark features

In this section, we show the construction of benchmark features that used in previous studies. Apart from the features that we described in the main manuscript, the nucleotide k-mers, amino acid k-mers, amino acid k-mers grouped by physio-chemical properties, and the Machine learning with digital signal processing-based Structural Patterns (M-SP) are discussed. Additionally, the we assessed multiple feature combinations, whose details are demonstrated below.

k-mer The nucleotide k-mer, a k-length substring of nucleotide sequences, is the most commonly used feature in bioinformatics. Following the calculation of the genomic tarits preference, we calculated the preference of k-mer usage, by counting the occurrence of k-mers in the nucleotide sequence, normalizing it by the total base number, multiplied a weighted constant (4^k), and transformed the final value by the log2 function. If the value is positive, the corresponding k-mer is over-represented; otherwise, the corresponding k-mer is under-represented. The calculation is shown in Eq. 1.

$$P_x = \log_2 \left(\frac{O_x + \epsilon}{\sum_x O_x} * 4^k\right) \tag{1}$$

where x denotes the k-mer x, O denotes the occurrence, ϵ is a small number of 0.001, k is the length of k-mer, and P is the final preference value.

Amino acid k-mer Likewise, the amino acid k-mer is the k-length substring of protein sequences. We also encode the query sequences as the usage preference of the amino acid k-mers across all the coding region (AAk). The calculation is shown in Eq. 3.

$$P_A = \log_2 \left(\frac{O_A + \epsilon}{\sum_A O_A} * 20^k\right) \tag{2}$$

where A denotes the amino acid k-mer A, O denotes the occurrence, ϵ is a small number of 0.001, k is the length of k-mer, and P is the final preference value.

Physio-chemical amino acid k-mer Binning the amino acid based on their physio-chemical (PC) properties is proved to be an effective way to reduce the dimension of amino acid k-mers. After grouping the amino acid into 7 bins, like (AGV, C, FILP, MSTY, HNQW, DE, KR), the query sequence can be represented as the usage preference of the PC amino acid k-mers (PCk). The calculation is shown in Eq. 3.

$$P_A = \log_2 \left(\frac{O_A + \epsilon}{\sum_A O_A} * 7^k\right) \tag{3}$$

where A denotes the PC amino acid k-mer A, O denotes the occurrence, ϵ is a small number of 0.001, k is the length of k-mer, and P is the final preference value.

M-SP: Machine learning with digital signal processing-based structural patterns The Machine learning with digital signal processing (ML-DSP) is an alignment free method that proposed by Randhawa et al. [2] and was further applied on host prediction of RNA viruses by Lee et al. [3], which was named as Machine learning with digital signal processing-based structural patterns (M-SP) of the viral sequences. Specifically, the feature tries to capture the potential host information in the Fourier transformation (FT) of the viral sequences. We totally follow the feature extraction implementation of Lee et al. [3], and a brief description is as below.

First, the nucleotide sequences are transformed into a numeric format by encoding purines (C, T) as $+1$ and pyrimidines (A, G) as -1. Next, the numeric sequences are subjected to Fourier Transform (FT). The correlation coefficients between the FTs serve as a measure of sequence similarity. A similarity matrix is then computed based on the correlation coefficients among the FTs. Subsequently, the top 5 reference sequences exhibiting the highest similarity with the query sequences are identified as candidates for determining the host of the query. The weights of the candidates are positively related to their similarity with the query. After summing the weights associated with the same host group, they are then normalized to derive the final weight vector, known as M-SP traits. These traits are believed to capture the association patterns between the query sequence and its potential hosts.

3 Feature selection

To gain insights into the contribution of genomic traits to host prediction, we examined their importance using XGBoost feature importance. We obtained feature importance from classifiers trained on various virus orders, selected features based on their importance ranks, and retrained models using the chosen subset of features. The genomic traits were grouped based on their definitions, and the importance scores were calculated, as depicted in Table S1. While the total importance of codon pair bias is the largest, the mean importance associated with it is relatively small. This could be attributed to the fact that codon pair bias consists of a significantly larger number of features (3,904), which represents the majority of genomic traits. Consequently, the cumulative importance value is substantial. However, the individual contribution of codon pairs is limited due to their sparsity in RNA viruses.

Furthermore, to assess the impact of codon pair bias on the host prediction of RNA viruses, we conducted additional experiments. We augmented the feature set by including codon pairs with high importance and retrained the classifier models. The evaluation was performed using cross validation. The results are presented in Fig. S2. The model that did not utilize codon pair bias achieved the highest average accuracy across different virus orders in Layer 1. As we gradually incorporated more codon pair bias features into the model, the average accuracy decreased from 92.38% to 90.08%. This decline in accuracy with an increasing number of features aligns with previous findings [4, 3]. Consequently,

we made the decision to exclude codon pair bias for higher prediction accuracy.

4 The trade-off between prediction rate and precision

Given the rapid accumulation of novel RNA viruses and their broad host range, it is possible that some viruses may infect hosts outside the scope of this study, despite our efforts to collect a diverse range of viruses. Therefore, we tend to reject virus queries that may not infect the target hosts, even if it leads to a lower prediction rate. To derive the best prediction score cutoff, we investigated prediction rate and precision at every possible score cutoff for nine virus orders which include more than 500 viruses. Specifically, for each query sequence, the models output a possibility vector corresponding to the hosts. We recorded the highest score in this vector as the prediction score and the corresponding host as the predicted label. Then, we collected all the prediction scores in one virus order and set score cutoffs to decide whether to output the prediction or not. If the prediction score is larger than the cutoff, the model will output the prediction label; otherwise, the prediction of the query will stop. We calculated the prediction rate and precision for every possible cutoff, and derived curves demonstrating the performance of RNAVirHost by connecting the prediction rate and precision. We compared RNAVirHost with BLASTN in the first layer of the host phylogenetic tree. The result is shown in Fig. S3. In the nine orders, the curve generated by RNAVirHost is closer to the upper right corner, indicating that while maintaining the same prediction rate, the prediction made by RNAVirHost is more precise. And then we determined the prediction score cutoff to be the score achieving the same prediction rate as BLASTN (green vertical line). By applying this cutoff, we classified the predictions into two categories: high confidence and low confidence.

References

[1] Ahmed A Zayed, James M Wainaina, Guillermo Dominguez-Huerta, Eric Pelletier, Jiarong Guo, Mohamed Mohssen, Funing Tian, Akbar Adjie Pratama, Benjamin Bolduc, Olivier Zablocki, et al. Cryptic and abundant marine viruses at the evolutionary origins of earth's rna virome. Science, 376(6589):156–162, 2022.

[2] Gurjit S Randhawa, Kathleen A Hill, and Lila Kari. Ml-dsp: Machine learning with digital signal processing for ultrafast, accurate, and scalable genome classification at all taxonomic levels. BMC genomics, 20:1–21, 2019.

[3] Bill Lee, David K Smith, and Yi Guan. Alignment free sequence comparison methods and reservoir host prediction. Bioinformatics, 37(19):3337–3342, 2021.

[4] Simon A Babayan, Richard J Orton, and Daniel G Streicker. Predicting

reservoir hosts and arthropod vectors from evolutionary signatures in rna virus genomes. Science, 362(6414):577–580, 2018.
