# Supplementary material for: RNAVirHost: a machine learning–based method for predicting hosts of RNA viruses through viral genomes
Source: Gigascience. 2024 Aug 22;13:giae059. doi: 10.1093/gigascience/giae059 (PMC11340644; doi:10.1093/gigascience/giae059)
Supplement: giae059_Supplemental_Files [file giae059_supplemental_files.zip › Supplementary_Fig_S4.pdf]

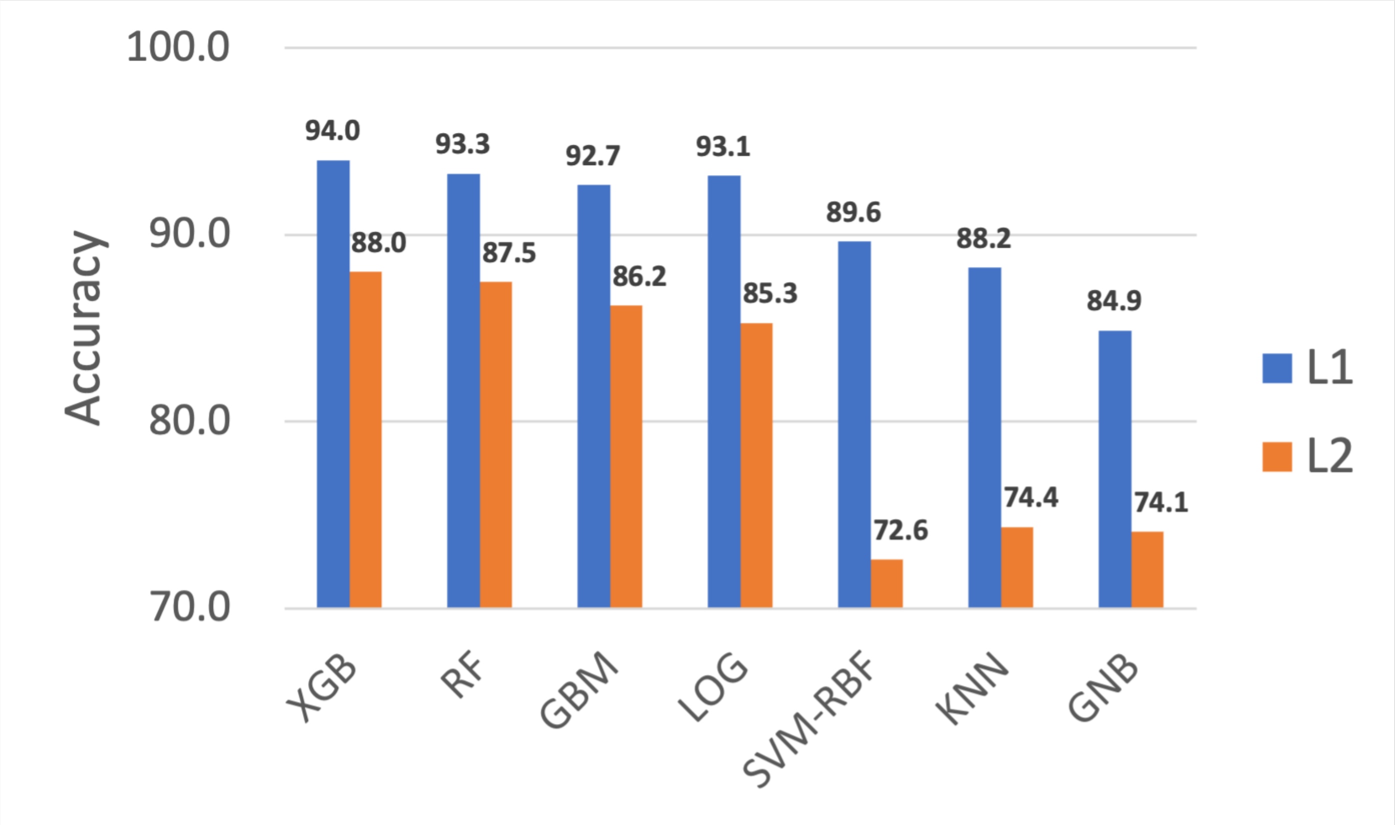
Accuracy
100.0
90.0
80.0
70.0
94.0
88.0
93.3
87.5
92.7
86.2
93.1
85.3
89.6
72.6
88.2
74.4
84.9
74.1
XGB
RF
GBM
LOG
SVM-RBF
KNN
GNB
L1
L2
