# Supplementary material for: RNAVirHost: a machine learning–based method for predicting hosts of RNA viruses through viral genomes
Source: Gigascience. 2024 Aug 22;13:giae059. doi: 10.1093/gigascience/giae059 (PMC11340644; doi:10.1093/gigascience/giae059)
Supplement: giae059_Supplemental_Files [file giae059_supplemental_files.zip › Supplementary_Table_S1.pdf]

	Nu	Dinu	AA_bias	Dinu_bdg	Dinu_non_bdg	codon	Codon_pair
# of features	4	16	21	16	16	64	3904
Sum of importance	2.50%	7.80%	9.80%	3.90%	3.90%	8.10%	63.90%
Mean of importance	0.63%	0.49%	0.47%	0.24%	0.25%	0.13%	0.02%
Fold between the observed and expected importance	25.5	19.7	18.9	9.9	9.9	5.1	0.7
