# Supplementary figures and images for: RNAVirHost: a machine learning–based method for predicting hosts of RNA viruses through viral genomes
Source: Gigascience. 2024 Aug 22;13:giae059. doi: 10.1093/gigascience/giae059 (PMC11340644; doi:10.1093/gigascience/giae059)

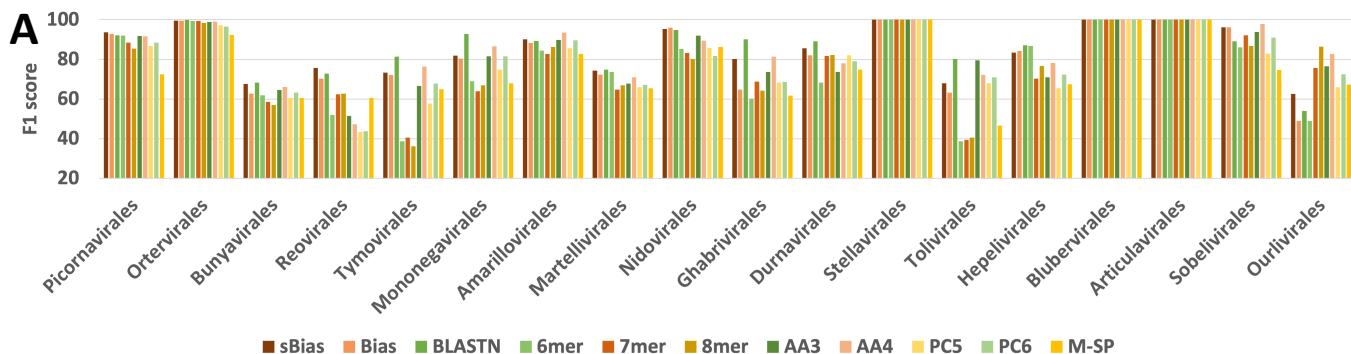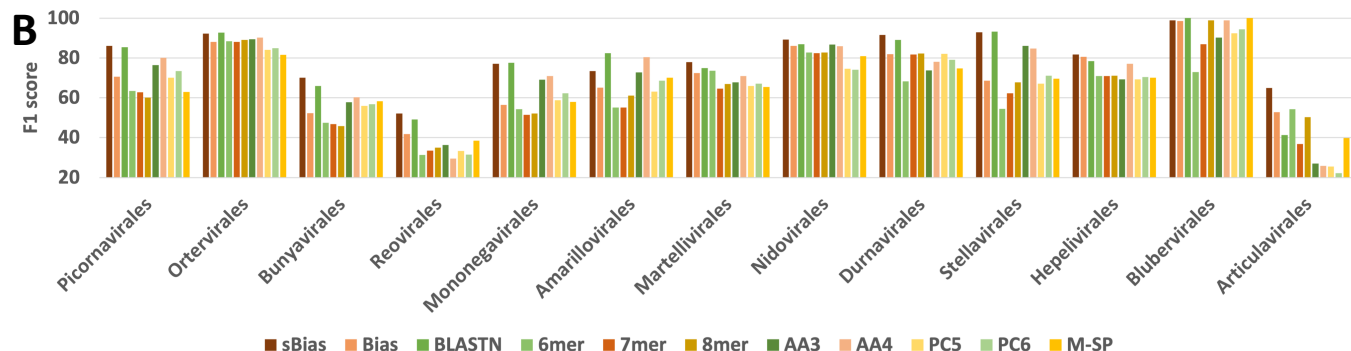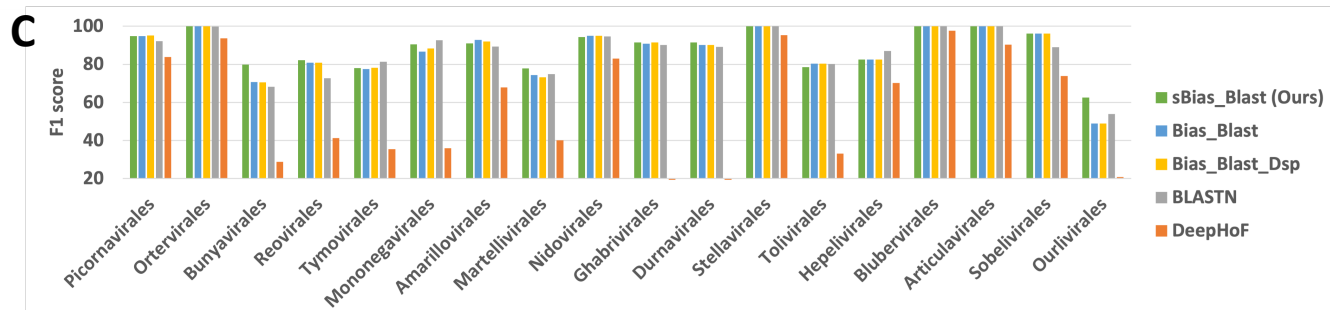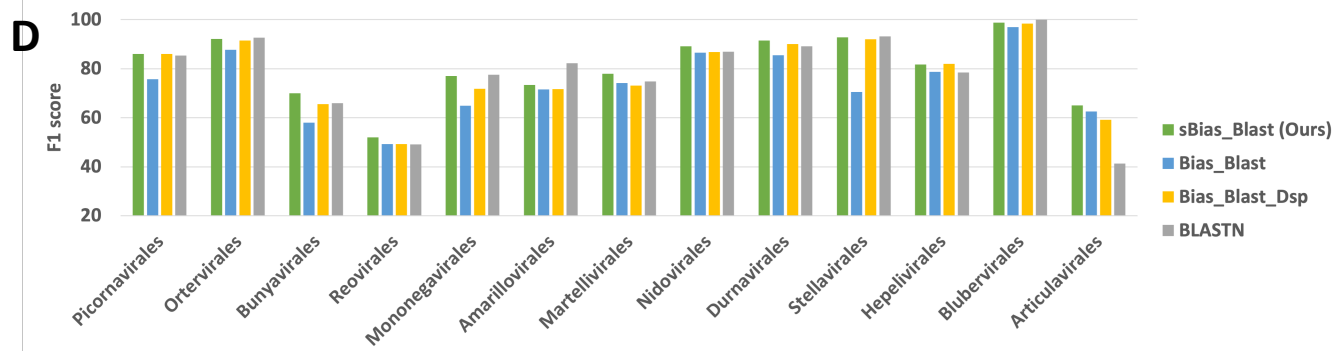

Supplement: giae059_Supplemental_Files [file giae059_supplemental_files.zip › Supplementary_Fig_S1.pdf]

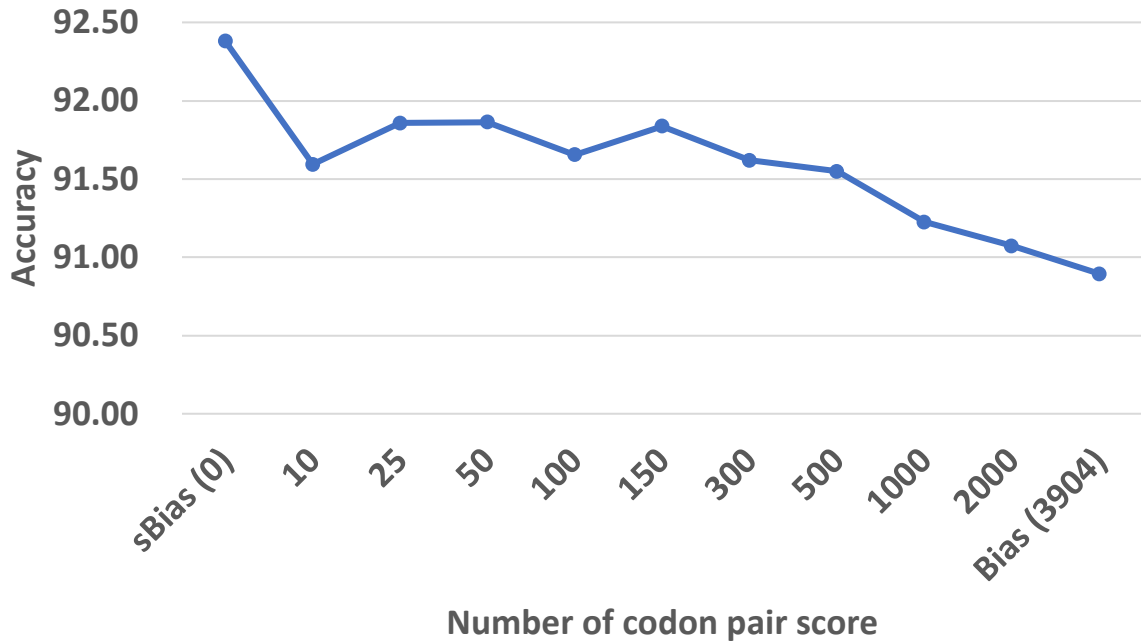

Supplement: giae059_Supplemental_Files [file giae059_supplemental_files.zip › Supplementary_Fig_S2.pdf]

**A**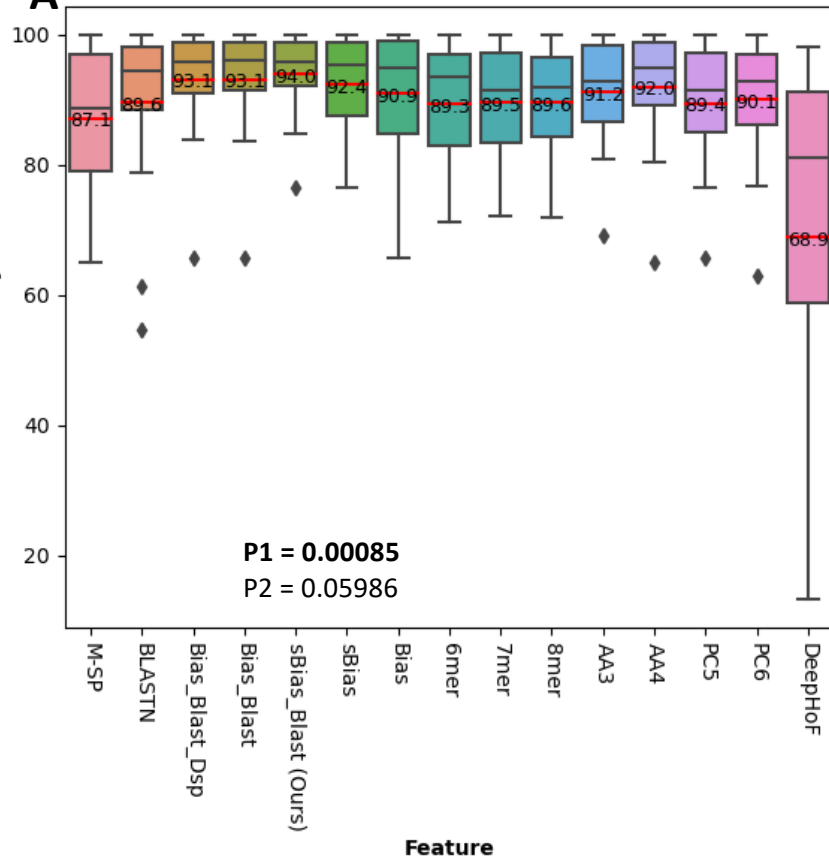**B**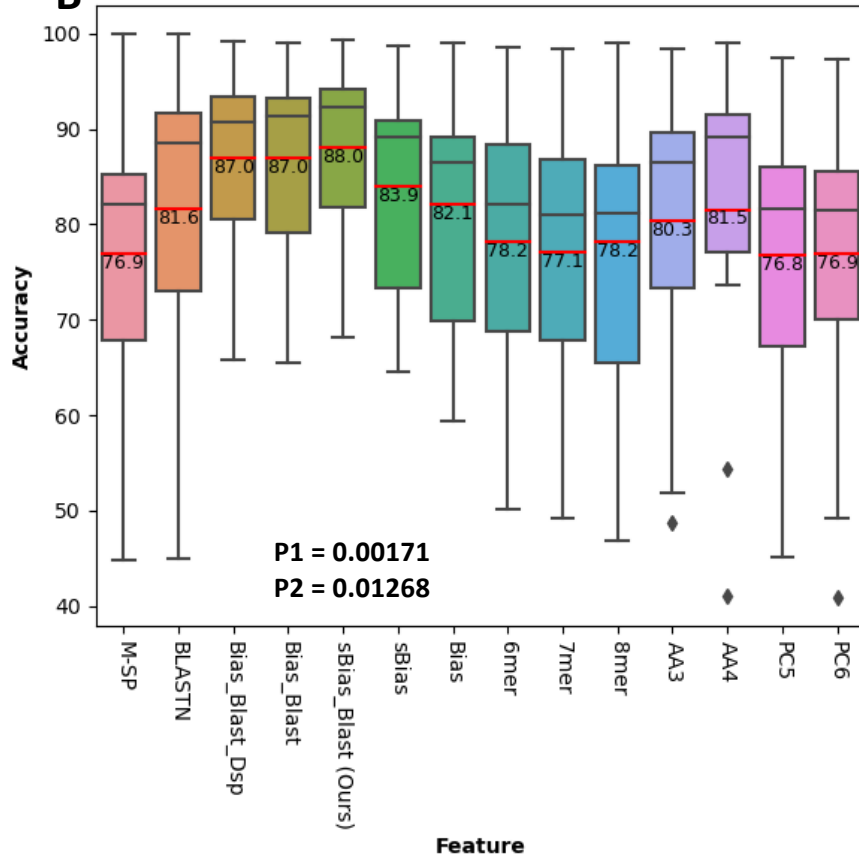

Supplement: giae059_Supplemental_Files [file giae059_supplemental_files.zip › Supplementary_Fig_S3.pdf]

Precision

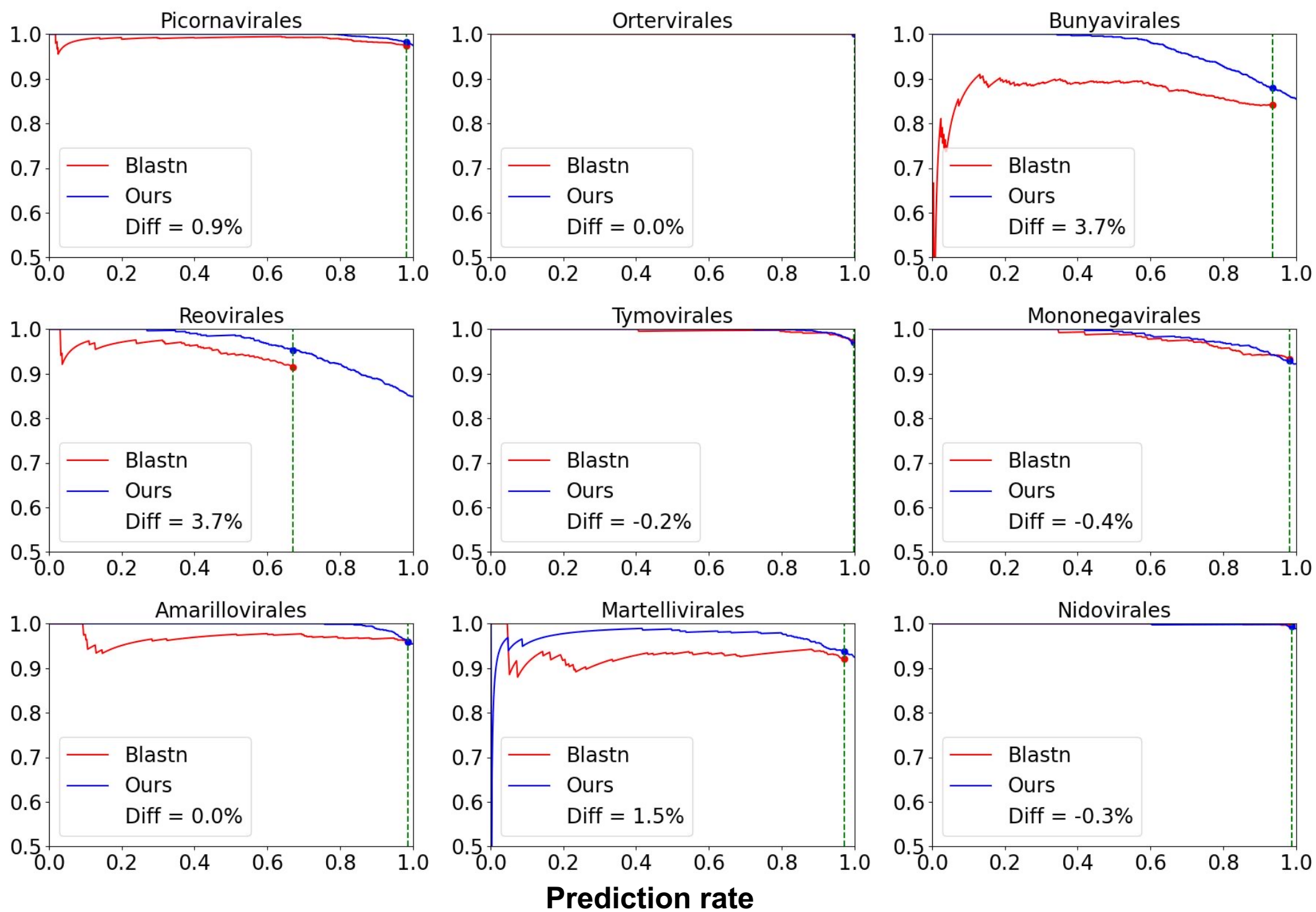

Supplement: giae059_Supplemental_Files [file giae059_supplemental_files.zip › Supplementary_Fig_S5.pdf]

Layer 1

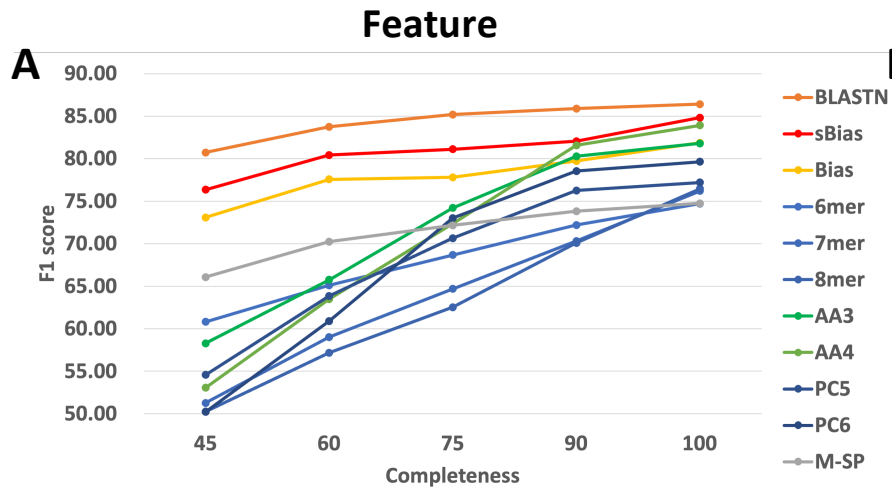

B

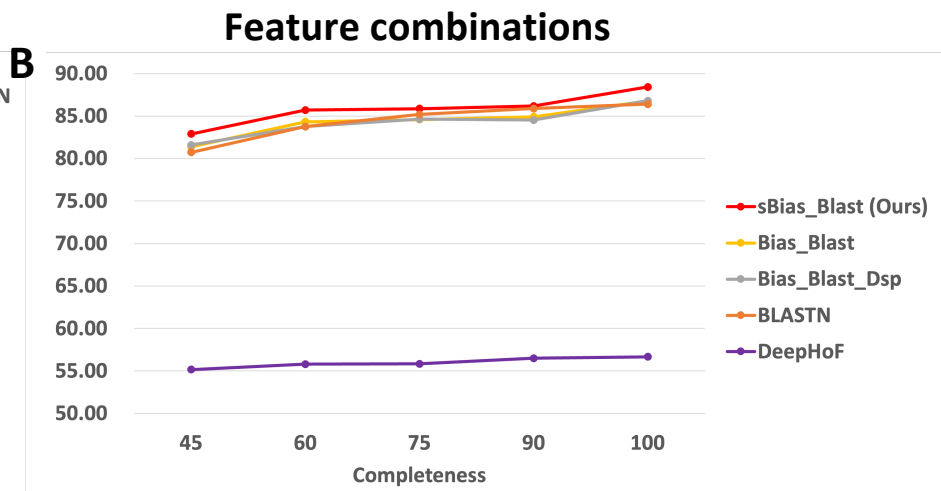

Layer 2

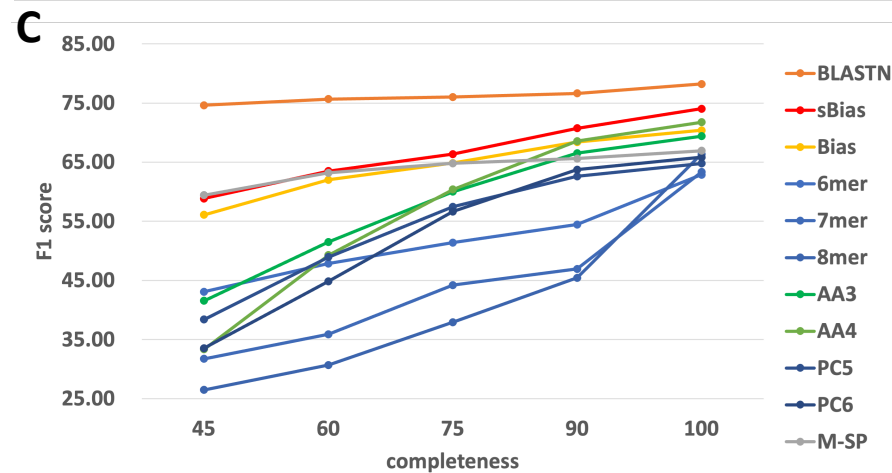

D

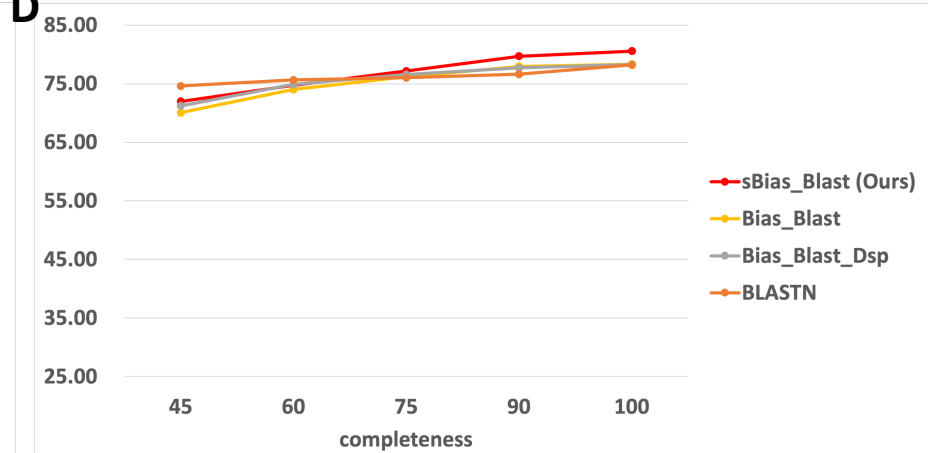

Supplement: giae059_Supplemental_Files [file giae059_supplemental_files.zip › Supplementary_Fig_S6.pdf]
