# Supplementary material for: RNAVirHost: a machine learning–based method for predicting hosts of RNA viruses through viral genomes
Source: Gigascience. 2024 Aug 22;13:giae059. doi: 10.1093/gigascience/giae059 (PMC11340644; doi:10.1093/gigascience/giae059)
Supplement: giae059_Response_to_Reviewer_Comments_Original_Submission [file giae059_response_to_reviewer_comments_original_submission.pdf]

Author's Response To Reviewer Comments

Dear Editor and Reviewers,
We would like to thank you for your careful reading of our paper and the valuable comments. We have addressed the comments of all reviewers, with their comments in italic and our responses in blue. The revised figures and descriptions were added to the main manuscript and the supplementary file. The changes in the main draft and the supplementary file are highlighted in red. We also submitted a response letter with refined figures and typesetting as a supplementary material for your convenience.

Before we provide the detailed responses, we would like to summarize the major improvements or modifications below.
1. We expanded the discussion on host prediction problem to better define the challenge. This clarifies the complexities and nuances involved in this area of research, while maintaining the reliability of the study.
2. We provided more details about the benchmark datasets, including data sources, taxonomic distributions of hosts and viruses, and data generation standards, to ensure fair and transparent comparisons.
3. We further evaluated the different strategies in the data imbalanced task using the F1 score as an additional metric, providing a more comprehensive assessment of performance.
4. We conducted an additional assessment on predicting hosts using fragmented viral contigs.
5. We carefully revised and polished the manuscript and figures to enhance clarity, readability, and overall presentation.
6. We changed the tool's name to better reflect the tool's function and input requirement.

We hope that the following point-to-point responses will be clear enough to convey our ideas and address the comments of the reviewers.

Response to Reviewer 1's comments
Chen et al. have developed a new tool to predict hosts for RNA viruses. Tools like VirHost are highly beneficial for understanding the interactions between viruses and their hosts. However, I believe the current manuscript has several limitations that need to be addressed before further assessment:
Thank you for your summary. We greatly appreciate your time in reviewing our manuscript. We have revised the manuscript accordingly. Our point-by-point responses are detailed below.

1) The authors should share their training and test datasets. There is not enough information about these datasets in the manuscript.
Thank you for your valuable feedback. Following this suggestion, we have expanded the "Data Collection" section to provide more details about the reference dataset, including its sources and filtering criteria.
In the experiments on "novel RNA viruses", we generate the "novel RNA viruses" by leaving a specific genus of reference viruses as the test set. This genus is not part of the training data, thus ensuring a rigorous test of our method. Then, we trained the model without including the specific genus and assessed its performance on that genus to mimic the situation where a novel query, particularly with an unknown genus label, is used as input. The leave-one-genus-out evaluations are conducted on all virus genera (448 virus genera). As all the test sequences were generated from the reference dataset, we got the genus labels from the annotation files of Virus-Host DB and NCBI GenBank. The information can be found in section "Performance on novel RNA viruses".
For the "recently identified viruses", we collected 20 publications that focused on identifying novel viruses on specific hosts and confirmed the viral infection by laboratory measures. While fifteen of them were published after 2023, we expanded the dataset by including five more works that were published before 2023 to cover different hosts as comprehensively as possible. We manually curated the dataset to ensure that the reference dataset does not include any new viral sequences presented in the 20 works. Besides, most of the works were designed on understudied hosts, for example, Hippocampus erectus (seahorse) as fishes, various fungi, Stellaria aquatica and Cnidium officinale as plant, and Apis mellifera (Western honey bee) as Invertebrate. More details can be accessed in section "Performance on recently identified viruses".
Furthermore, for reproducibility and further analysis, we have deposited all data used in the study in GigaDB, making it readily accessible to the research community. Upon publication, the GigaDB repository will be accessible through the link provided in section "Data availability".

2) They should compare their tool with similar tools/ pipelines for its prediction performance.
Thank you for this comment. We have compared our pipeline with related methods/tools/pipelines. In the section “related work”, we introduced a list of works that can be used to predict the hosts of metagenomic-assembled viruses instead of specific viruses or hosts. For those that can be used on RNA viruses, we have included them in the comparison. The corresponding publications for the benchmarked methods are: Bias [1], Bias_Blast [1], DSP [2], Bias_Blast_Dsp [2], all types of k-mer [3], and DeepHoF [4]. Except DeepHoF, other pipelines lack well-packaged software. Thus, we retrained/reimplemented their methods and renamed them based on their main features. The performance comparison with these tools can be found in Figures 2, 4, S1, and S6. More detailed information is provided in the second paragraph of “Assessment via cross-validation” section.
[1] Babayan, S. A., Orton, R. J., & Streicker, D. G. (2018). Predicting reservoir hosts and arthropod vectors from evolutionary signatures in RNA virus genomes. Science, 362(6414), 577-580.
[2] Lee, B., Smith, D. K., & Guan, Y. (2021). Alignment free sequence comparison methods and reservoir host prediction. Bioinformatics, 37(19), 3337-3342.
[3] Young, F., Rogers, S., & Robertson, D. L. (2020). Predicting host taxonomic information from viral genomes: A comparison of feature representations. PLoS computational biology, 16(5), e1007894.
[4] Guo, Q., Li, M., Wang, C., Guo, J., Jiang, X., Tan, J., ... & Zhu, H. (2021). Predicting hosts based on early SARS-CoV-2 samples and analyzing the 2020 pandemic. Scientific Reports, 11(1), 17422.

4) The manuscript could benefit from proofreading.
Thank you for your suggestions. We have carefully revised and polished the manuscript to enhance clarity.

5) I found the introduction to be unfocused and lacking enough coherence.
Thank you for your comment. Our introduction intends to provide a more comprehensive background for host prediction problem. To better convey the challenges of host prediction, we not only discussed the host range and definition of viruses but also compared different challenges of host prediction problems between DNA viruses and RNA viruses.
To improve the manuscript's readability and coherence, we have reorganized its structure and introduced a new subsection titled "Related Work." With the new changes, the organization of Introduction is: 1) introduction of RNA viruses, 2) introduction of the data for virus discovery and need for host identification, 3) problem formulation with a focus on the discussion of “hosts”, 4) a comprehensive discussion of the current methods for identifying the virus host. In the subsection “Related work”, we first acknowledged the experimental methods. Then, as there is a fast accumulation of prokaryotic virus host prediction tools, we briefly covered them and concluded why these tools cannot be applied to eukaryotic viruses. Then, we discussed the state-of-the-art RNA virus host prediction in two scenarios. The first scenario covers tools that predict the hosts for a targeted group of viruses, such as viruses in only a couple of families. The second scenario covers tools that predicted a specified set of hosts for a virus, such as deciding whether a virus can infect human.
We expect these modifications to highlight the focus of our work.

Response to Reviewer 2’s comments
The authors present a new tool for annotation of RNA viruses to generate host reservoirs for a viral genome of a known viral order. The paper is generally well written and this new concept for viral annotations would be of interest to the wider scientific community.
Thank you for providing the summary. We sincerely appreciate your time and comments. Our point-to-point responses are shown below.

Main comments:
I would recommend a change in the tool name (you can keep if you wish). There are many viral host prediction tools, and the name VirHost implies a general host prediction tool whereas it appears to be more specialized.
Thank you for this suggestion. To better abbreviate the function of our tool, we change the name to “RNAVirHost”, which further clarifies the scope of virus type and improves the conciseness. We supposed this name could be a better summary of our tool.

I would like a clearer definition of "natural-hosts" and "reservoir hosts", and what make a reservoir host annotation different to a host annotation. This should also be clearer in the abstract.

Thank you for this insightful comment. While viruses' hosts are clearly defined as cellular organisms that the viruses can infect and replicate within their cells, there are various conflicting definitions of "reservoir". Some studies highlighted that the infections in reservoirs are always nonpathogenic; some stressed that the reservoirs allow the persistence of the viruses; some suggested the uniqueness of the reservoir; others claimed that reservoirs could be a community involving various species [1,2,3]. Usually, the minimum set of host communities where the viruses can sustain can be accepted as "reservoirs". For example, wild aquatic birds are considered reservoirs for avian influenza viruses [4]. However, it is hard to state who is not part of the "reservoirs". In some cases, viruses can be sustained in a larger set of hosts. For instance, dogs and bats are individual sources of rabies viruses, which can be sustained in both communities solely [5].

By definition, the natural hosts comprise reservoir and secondary hosts. The secondary hosts are supposed to be infected in the spillover events, and the viruses show distinctive characteristics in secondary hosts, like higher pathogenicity and strong lethality [3,8]. Even though spillover events have shown their significant threat to human, they are relatively rare during the long co-evolutionary period with their reservoir hosts [6].

In the first draft, we followed Babayan [7] and defined the problem as a reservoir prediction problem. However, the definition of a reservoir is more stringent and complex than the definition a host. In this revision, we followed the usage of the "host" in the Virus-Host Database and NCBI GenBank. These widely used reference datasets use "hosts" rather than "reservoirs".

Therefore, given that the concept of "reservoir" involves various aspects of viruses, including replication, transmission, maintenance, pathogenicity, and virulence, and has not yet received a common-acknowledged definition, we decided to shift the name from "reservoir" to "host" in this context, which is expected to define the objective better and enhance the clarity. This modification aligns our research goals with the two data sources' definitions of viruses' hosts.

[1] Haydon, D. T., Cleaveland, S., Taylor, L. H., & Laurenson, M. K. (2002). Identifying reservoirs of infection: a conceptual and practical challenge. Emerging infectious diseases, 8(12), 1468-1473.

[2] Guth, S., Visher, E., Boots, M., & Brook, C. E. (2019). Host phylogenetic distance drives trends in virus virulence and transmissibility across the animal–human interface. Philosophical Transactions of the Royal Society B, 374(1782), 20190296.

[3] Brook, C. E., Rozins, C., Guth, S., & Boots, M. (2023). Reservoir host immunology and life history shape virulence evolution in zoonotic viruses. Plos Biology, 21(9), e3002268.

[4] Blagodatski, A., Trutneva, K., Glazova, O., Mityaeva, O., Shevkova, L., Kegeles, E., ... & Volchkov, P. (2021). Avian influenza in wild birds and poultry: dissemination pathways, monitoring methods, and virus ecology. Pathogens, 10(5), 630.

[5] Brunker, K., & Mollentze, N. (2018). Rabies virus. Trends in microbiology, 26(10), 886-887.

[6] Plowright, R. K., Parrish, C. R., McCallum, H., Hudson, P. J., Ko, A. I., Graham, A. L., & Lloyd-Smith, J. O. (2017). Pathways to zoonotic spillover. Nature Reviews Microbiology, 15(8), 502-510.

[7] Babayan, S. A., Orton, R. J., & Streicker, D. G. (2018). Predicting reservoir hosts and arthropod vectors from evolutionary signatures in RNA virus genomes. Science, 362(6414), 577-580.

[8] Mollentze, N., Streicker, D. G., Murcia, P. R., Hampson, K., & Biek, R. (2020). Virulence mismatches in index hosts shape the outcomes of cross-species transmission. Proceedings of the National Academy of Sciences, 117(46), 28859-28866.

The benchmarking datasets should be provided prior to publication. The authors also need to describe their approach for selecting genomes to include for the benchmarking datasets for 'novel' viruses and for the recently identified viruses.

Thank you for your valuable feedback. Following this suggestion, we have expanded the "Data Collection" section to provide more details about the reference dataset, including its sources and filtering criteria.

In the experiments on "novel RNA viruses", we generate the "novel RNA viruses" by leaving a specific genus of reference viruses as the test set. This genus is not part of the training data, thus ensuring a rigorous test of our method. Then, we trained the model without including the specific genus and assessed its performance on that genus to mimic the situation where a novel query, particularly with an unknown genus label, is used as input. The leave-one-genus-out evaluations are conducted on all virus genera (448 virus genera). As all the test sequences were generated from the reference dataset, we got the genus

labels from the annotation files of Virus-Host DB and NCBI GenBank. The information can be found in section “Performance on novel RNA viruses”.
For the “recently identified viruses”, we collected 20 works that focused on identifying novel viruses on specific hosts and confirmed the viral infection by laboratory measures. While fifteen of them were published after 2023, we expanded the dataset by including five more works that were published before 2023 to cover different hosts as comprehensively as possible. We manually curated the dataset to ensure that the reference dataset does not include any new viral sequences presented in the 20 works. Besides, most of the works were designed on understudied hosts, for example, Hippocampus erectus (seahorse) as fishes, various fungi, Stellaria aquatica and Cnidium officinale as plant, and Apis mellifera (Western honey bee) as Invertebrate. More details can be accessed in section “Performance on recently identified viruses”.
Furthermore, for reproducibility and further analysis, we have deposited all data used in the study in GigaDB, making it readily accessible to the research community. Upon publication, the GigaDB repository will be accessible through the link provided in section “Data availability”.

The authors could calculate F1 scores to highlight optimizing the trade-off between sensitivity and precision.
Thank you for your comment. We have further evaluated the different prediction methods using the F1 score as an additional metric. Our tool still showed superior performance in most experiments. The new figures can be found in Supplementary Figure S1.
In the cross-validation experiments, our tool showed the highest F1 scores across various virus orders and host layers. In Layer 1 (hosts’ kingdom or phylum level), we achieved the highest F1 score of 88.41%, outperforming the second-best strategy, Bias_Blast_Dsp (a method from [1]), by 1.6%. In Layer 2 (hosts’ class or order level), we also achieved the highest F1 score of 80.59%, outperforming the second-best strategy, Bias_Blast (a method from [2]), by 2.25%. The higher F1 score of our tool indicates its robustness against the imbalanced host labels. The result is shown below:

Fig. S1 Host prediction performance (F1 score) of different feature sets in Layer 1 (A) and Layer 2 (B). Host prediction performance (F1 score) of different feature combinations in Layer 1 (C) and Layer 2 (D). X-axis: Virus orders sorted by size (from largest to smallest). The performance comparison in Layer 2 considers the errors from both Layer 1 and Layer 2. Among the 18 orders, thirteen can be further classified in Layer 2. Therefore, we focused our performance evaluation solely on Layer 2 within these thirteen orders.
[1] Lee, B., Smith, D. K., & Guan, Y. (2021). Alignment free sequence comparison methods and reservoir host prediction. Bioinformatics, 37(19), 3337-3342.
[2] Babayan, S. A., Orton, R. J., & Streicker, D. G. (2018). Predicting reservoir hosts and arthropod vectors from evolutionary signatures in RNA virus genomes. Science, 362(6414), 577-580.

Minor comments:
-The concept of a reservoir instead of a host should be explained in the abstract.
Thank you for your comment. We added more discussion about hosts in Introduction and Abstract. More detailed information about this can be found in our response to the second major comment.

-Please check spelling of virus names. Flaviviridae is misspelled.
Thank for pointing this out. We have carefully checked the spelling of virus taxa.

-Code looks good, were you planning on releasing it as a bioconda or pypi package?
Thank you for your comment. Now, we have packaged and distributed the project in a more accessible way through Bioconda, PyPi, and GitHub.

-The color scale for fig3b is odd. Is there a better way to highlight the chordata bias for invertebrate?
Thank you for this valuable comment. We now modified Fig. 3(B) as shown below.

Fig. 3 (B) The confusion matrix of \tool's prediction in Layer 1. The values outside the brackets denote the percentage of the misclassified members, which were normalized to the sum of misclassified viruses of the corresponding groups (by row). The values in the brackets denote the percentage of the prediction labels, normalized to the corresponding groups' total number (by row). The viruses infecting bacteria are limited to specific virus orders, which exclusively infect bacteria. Hence, we do not visualize bacteria here.

Response to Reviewer 3’s comments
The authors present a machine learning-based tool named VirHost that can predict the reservoir hosts of RNA viruses. The motivation behind the development of the tool is clear and well-justified. The authors have thoroughly benchmarked the tool against existing tools such as DeepHoF based on different genomic features and machine learning architectures. The tool has also been tested on newly identified viruses whose hosts have been experimentally determined which shows the tool's potential. I appreciate the fact that the authors have identified and discussed the limitations of VirHost. Overall, I think this is a solid piece of work.
Thank you for providing the summary. We sincerely appreciate the time and effort you dedicated to reviewing our manuscript. Our point-to-point responses are shown below.

Comments for revisions in text
Metagenome assembly can be a challenging task, especially in recovering RNA viruses and genomes can be fragmented. In cases where the complete contiguous genomes are not recovered, especially for larger viruses, how will the length of the fragments (contigs) affect the prediction accuracy? The authors are welcome to discuss this aspect.
Thank you for this valuable comment. We have conducted an additional assessment on the fragmented viral sequences to evaluate the performance of the tools. Specifically, we followed the cross-validation setting and generated four groups of short contigs by cutting the test sequences with different length ratios, including 90%, 75%, 60%, 45%. We then evaluated the tools by their order-wise accuracy and F1 scores. Our results, presented in Fig. 4 and Fig. S6 as below, demonstrate superior performance compared to other strategies.
Among the simple feature sets, “sBias” and “Bias” consistently outperformed other alignment-free feature sets. Notably, even when the completeness of viral sequences was reduced to 45%, “sBias” maintained comparable accuracy compared to the alignment-based method, BLASTN. Among the feature combinations, our tool, combining “sBias” and BLASTN, demonstrated the highest accuracy and F1 scores in most cases. The new figures were added to the main manuscript and Supplementary File.

Fig. 4 Host prediction performance (order-wise accuracy) of different feature sets in Layer 1 (A, B) and Layer 2 (C, D). X-axis: the completeness of viral sequences. Y-axis: The order-wise accuracy of the corresponding feature set.

Fig. S6 Host prediction performance (order-wise F1 score) of different feature sets in Layer 1 (A, B) and Layer 2 (C, D). X-axis: the completeness of viral sequences. Y-axis: The order-wise F1 score of the corresponding feature set.

In Figure 2, do A and B have the same legend? It would be clearer to include separate legends for A and B.
Thank you for this comment. They do have the same legend. Following your suggestion, we added separate legends for A and B.

Minor comments
I noticed a couple of typos.
- Page 3: Although numerous RNA viruses "have" been found, the current...
- Page 4: To "represent" the "genomic features", we translate the query...
- Page 5: Considering that most RNA "viruses" are "shorter" than 50 kbp and...
- Page 5: A detailed description of the benchmarked "features" can be found in the...
- Page 7: We investigated host distribution of each virus family and visualized the "error rates" of virus...
- Page 7: ... implying that these wrong "predictions" are mainly from viruses with multiple hosts across phylum. - Page 8: The statistics "are" listed in Table 2.
- Page 8: ... which is a member of Alphaparti-tivirus, a genus that "infects" both "plants" and fungi.
- Page 9: With the increasing "availability of" viruses' host annotations...
I recommend the authors to proofread the final manuscript thoroughly.
Thank you for catching these. We have conducted additional rounds of proofreading and carefully revised and polished the manuscript with the assistance of Grammlary.

Comments for revisions in GitHub
Please add a .gitignore file so you can ignore the files and directories which are unnecessary to the git project (e.g. .idea).
Thank you for pointing this out. We have removed the unnecessary files.

With the current version of the code, I had to always either run virhost.py from the VirHost directory or give the full path to virhost.py. It would be more convenient if VirHost could be installed in the current Python environment so it can be called from anywhere. It might be worth looking into how to package and distribute Python projects.
It would be nice to put VirHost on Bioconda and PyPI so the users can easily install it.
Thank you for your comment. Now, we have packaged and distributed the project in a more accessible way through Bioconda, PyPi, and GitHub.

The authors mention "However, we recommend users to generate their own taxonomic classification result, which is expected to improve the prediction confidence.". It would be good to have more details on how to generate custom taxonomic classification results with recommended tools to use so that even someone who is not familiar with the available bioinformatics solutions can easily get started.
Thank you for your comment. The taxonomic classification file is necessary for the input requirement. We provide easy access for the users to get the order-level label through BLASTN. We aligned the query against the reference dataset and used the best hit strategy to assign the order-level virus label. While the order-level classification is not a hard task, this method can provide sufficiently accurate prediction.
Besides the default BLASTN program, we recommend two classification programs that can be applied to RNA viruses for users: PhaGCN2, CAT.
The resulting file includes two columns: the sequence ID and the viruses’ order labels (including 30 orders), as shown below. Now, we provide more detailed guidance in GitHub for users’ convenience.
