# Supplementary material for: RNAVirHost: a machine learning–based method for predicting hosts of RNA viruses through viral genomes
Source: Gigascience. 2024 Aug 22;13:giae059. doi: 10.1093/gigascience/giae059 (PMC11340644; doi:10.1093/gigascience/giae059)
Supplement: giae059_Reviewer_2_Report_Original_Submission — Michael Roach -- 4/10/2024 Reviewed [file giae059_reviewer_2_report_original_submission.pdf]

Reviewer Report

Title: RNAVirHost: a machine learning-based method for predicting hosts of RNA viruses through viral genomes

Version: Original Submission **Date:** 4/10/2024

Reviewer name: Michael Roach

Reviewer Comments to Author:

The authors present a new tool for annotation of RNA viruses to generate host reservoirs for a viral genome of a known viral order. The paper is generally well written and this new concept for viral annotations would be of interest to the wider scientific community.

Main comments:

I would recommend a change in the tool name (you can keep if you wish). There are many viral host prediction tools, and the name VirHost implies a general host prediction tool whereas it appears to be more specialised.

I would like a clearer definition of "natural-hosts" and "reservoir hosts", and what make a reservoir host annotation different to a host annotation. This should also be clearer in the abstract.

The benchmarking datasets should be provided prior to publication. The authors also need to describe their approach for selecting genomes to include for the benchmarking datasets for 'novel' viruses and for the recently identified viruses.

The authors could calculate F1 scores to highlight optimising the trade-off between sensitivity and precision.

Minor comments:

-The concept of a reservoir instead of a host should be explained in the abstract.

-Please check spelling of virus names. Flaviviridae is misspelled.

-Code looks good, were you planning on releasing it as a bioconda or pypi package?

-The color scale for fig3b is odd. Is there a better way to highlight the chordata bias for invertebrate?

Methods

Are the methods appropriate to the aims of the study, are they well described, and are necessary controls included? Choose an item.

Conclusions

Are the conclusions adequately supported by the data shown? Choose an item.

Reporting Standards

Does the manuscript adhere to the journal's guidelines on minimum standards of reporting? Choose an item.

Choose an item.

Statistics

Are you able to assess all statistics in the manuscript, including the appropriateness of statistical tests used? Choose an item.

Quality of Written English

Please indicate the quality of language in the manuscript: Choose an item.

Declaration of Competing Interests

Please complete a declaration of competing interests, considering the following questions:

- Have you in the past five years received reimbursements, fees, funding, or salary from an organisation that may in any way gain or lose financially from the publication of this manuscript, either now or in the future?
- Do you hold any stocks or shares in an organisation that may in any way gain or lose financially from the publication of this manuscript, either now or in the future?
- Do you hold or are you currently applying for any patents relating to the content of the manuscript?
- Have you received reimbursements, fees, funding, or salary from an organization that holds or has applied for patents relating to the content of the manuscript?
- Do you have any other financial competing interests?
- Do you have any non-financial competing interests in relation to this paper?

If you can answer no to all of the above, write 'I declare that I have no competing interests' below. If your reply is yes to any, please give details below.

I declare that I have no competing interests

I agree to the open peer review policy of the journal. I understand that my name will be included on my report to the authors and, if the manuscript is accepted for publication, my named report including any attachments I upload will be posted on the website along with the authors' responses. I agree for my report to be made available under an Open Access Creative Commons CC-BY license (http://creativecommons.org/licenses/by/4.0/). I understand that any comments which I do not wish to be included in my named report can be included as confidential comments to the editors, which will not be published.

Choose an item.

To further support our reviewers, we have joined with Publons, where you can gain additional credit to further highlight your hard work (see: https://publons.com/journal/530/gigascience). On publication of this paper, your review will be automatically added to Publons, you can then choose whether or not to claim your Publons credit. I understand this statement.

Yes Choose an item.
