# Supplementary material for: RNAVirHost: a machine learning–based method for predicting hosts of RNA viruses through viral genomes
Source: Gigascience. 2024 Aug 22;13:giae059. doi: 10.1093/gigascience/giae059 (PMC11340644; doi:10.1093/gigascience/giae059)
Supplement: giae059_Reviewer_3_Report_Original_Submission — Vijini Mallawaarachchi -- 4/12/2024 Reviewed [file giae059_reviewer_3_report_original_submission.pdf]

Reviewer Report

Title: RNAVirHost: a machine learning-based method for predicting hosts of RNA viruses through viral genomes

Version: Original Submission **Date:** 4/12/2024

Reviewer name: Vijini Mallawaarachchi

Reviewer Comments to Author:

The authors present a machine learning-based tool named VirHost that can predict the reservoir hosts of RNA viruses. The motivation behind the development of the tool is clear and well-justified. The authors have thoroughly benchmarked the tool against existing tools such as DeepHoF based on different genomic features and machine learning architectures. The tool has also been tested on newly identified viruses whose hosts have been experimentally determined which shows the tool's potential. I appreciate the fact that the authors have identified and discussed the limitations of VirHost. Overall, I think this is a solid piece of work.

Comments for revisions in text

Metagenome assembly can be a challenging task, especially in recovering RNA viruses and genomes can be fragmented. In cases where the complete contiguous genomes are not recovered, especially for larger viruses, how will the length of the fragments (contigs) affect the prediction accuracy? The authors are welcome to discuss this aspect.

In Figure 2, do A and B have the same legend? It would be more clear to include separate legends for A and B.

Minor comments

I noticed a couple of typos.

- Page 3: Although numerous RNA viruses "have" been found, the current...
- Page 4: To "represent" the "genomic features", we translate the query...
- Page 5: Considering that most RNA "viruses" are "shorter" than 50 kbp and...
- Page 5: A detailed description of the benchmarked "features" can be found in the...
- Page 7: We investigated host distribution of each virus family and visualized the "error rates" of virus...
- Page 7: ... implying that these wrong "predictions" are mainly from viruses with multiple hosts across phylum.
- Page 8: The statistics "are" listed in Table 2.
- Page 8: ... which is a member of Alphaparti-tivirus, a genus that "infects" both "plants" and fungi.
- Page 9: With the increasing "availability of" viruses' host annotations...

I recommend the authors to proofread the final manuscript thoroughly.

Comments for revisions in GitHub

Please add a .gitignore file so you can ignore the files and directories which are unnecessary to the git project (e.g. .idea).

With the current version of the code, I had to always either run virhost.py from the VirHost directory or give the full path to virhost.py. It would be more convenient if VirHost could be installed in the current

Python environment so it can be called from anywhere. It might be worth looking into how to package and distribute Python projects.
It would be nice to put VirHost on Bioconda and PyPI so the users can easily install it.
The authors mention "However, we recommend users to generate their own taxonomic classification result, which is expected to improve the prediction confidence.". It would be good to have more details on how to generate custom taxonomic classification results with recommended tools to use so that even someone who is not familiar with the available bioinformatics solutions can easily get started.

Methods

Are the methods appropriate to the aims of the study, are they well described, and are necessary controls included? Choose an item.

Conclusions

Are the conclusions adequately supported by the data shown? Choose an item.

Reporting Standards

Does the manuscript adhere to the journal's guidelines on minimum standards of reporting? Choose an item.

Choose an item.

Statistics

Are you able to assess all statistics in the manuscript, including the appropriateness of statistical tests used? Choose an item.

Quality of Written English

Please indicate the quality of language in the manuscript: Choose an item.

Declaration of Competing Interests

Please complete a declaration of competing interests, considering the following questions:

- Have you in the past five years received reimbursements, fees, funding, or salary from an organisation that may in any way gain or lose financially from the publication of this manuscript, either now or in the future?
- Do you hold any stocks or shares in an organisation that may in any way gain or lose financially from the publication of this manuscript, either now or in the future?
- Do you hold or are you currently applying for any patents relating to the content of the manuscript?

- Have you received reimbursements, fees, funding, or salary from an organization that holds or has applied for patents relating to the content of the manuscript?
- Do you have any other financial competing interests?
- Do you have any non-financial competing interests in relation to this paper?

If you can answer no to all of the above, write 'I declare that I have no competing interests' below. If your reply is yes to any, please give details below.

I declare that I have no competing interests.

I agree to the open peer review policy of the journal. I understand that my name will be included on my report to the authors and, if the manuscript is accepted for publication, my named report including any attachments I upload will be posted on the website along with the authors' responses. I agree for my report to be made available under an Open Access Creative Commons CC-BY license (http://creativecommons.org/licenses/by/4.0/). I understand that any comments which I do not wish to be included in my named report can be included as confidential comments to the editors, which will not be published.

Choose an item.

To further support our reviewers, we have joined with Publons, where you can gain additional credit to further highlight your hard work (see: https://publons.com/journal/530/gigascience). On publication of this paper, your review will be automatically added to Publons, you can then choose whether or not to claim your Publons credit. I understand this statement.

Yes Choose an item.
